# Influence of Achiral Phosphine Ligands on a Synergistic Organo‐ and Palladium‐Catalyzed Asymmetric Allylic Alkylation

**DOI:** 10.1002/chem.202202951

**Published:** 2022-10-27

**Authors:** David McLeod, Nicolaj Inunnguaq Jessen, Thanh V. Q. Nguyen, Marcus Espe, Jeremy David Erickson, Karl Anker Jørgensen, Limin Yang, K. N. Houk

**Affiliations:** ^1^ Department of Chemistry Aarhus University Langelandsgade 140 8000 Aarhus C Denmark; ^2^ College of Materials Chemistry and Chemical Engineering Hangzhou Normal University 311121 Hangzhou P. R. China; ^3^ Department of Chemistry and Biochemistry Department of Biochemical and Biomolecular Engineering University of California 90095 Los Angeles California USA

**Keywords:** allylic alkylation, asymmetric synthesis, DFT calculations, mechanism, synergistic catalysis

## Abstract

An unusual diastereodivergent stereoselective allylation reaction is presented. It consists of a palladium‐catalyzed allylation reaction of an organocatalytically generated amino isobenzofulvene, where the diastereoselectivity is controlled by the electronic properties of a monodentate, achiral ligand on palladium. One major diastereoisomer is formed using triarylphosphines substituted with neutral or electron‐donating substituents of the aryl group, while those with electron‐withdrawing substituents favor the other diastereoisomer. The diastereoselectivity correlates with the Taft inductive parameter of substituents on the triarylphosphine ligand on palladium. The synergistic reaction involves both a catalytic secondary amine catalyst for the indene‐aldehyde activation and the monodentate phosphine ligands on palladium, affording a highly enantioselective reaction with up to 98 % enantiomeric excess. Based on computational investigations, the role of the monodentate phosphine ligand on the diastereoselectivity is discussed.

## Graphical abstract

**Through the employment** of synergistic amino‐ and palladium catalysis, an allylic alkylation is disclosed where all four stereoisomers can be accessed as the major product. Importantly, this stereodivergence has been developed to employ the aminocatalyst as the sole chiral reagent. While the aminocatalyst dictates the enantioselectivity, diastereoselectivity is governed by the nature of an achiral, monodentate phosphine ligand on palladium.

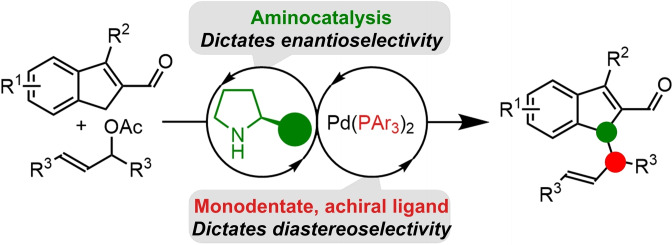

## Introduction

The allylation reaction is a fundamental and important transformation in organic synthesis.[^1^, ^2^] Moreover, few transition metal catalyzed reactions offer the versatility of palladium‐catalyzed asymmetric allylic alkylations.[^3^, ^4^] The robust nature of this chemistry, and the fact that palladium allyl complexes can be formed under mild conditions‐from relatively unreactive allylic acetate/carbonates‐renders these reactions ideal models for synergistic catalysis.^[5]^

While the concept of synergistic catalysis is not new, only in the last decade has it really been exploited to enable new chemistry not attainable with a single catalyst.[^6^, ^7^, ^8^, ^9^, ^10^] Among the first examples[^11^, ^12^, ^13^] of synergistic catalysis involving an organocatalytic cycle, the direct α‐allylic alkylation of unactivated aldehydes and cyclic ketones with allyl acetate was achieved through combinatorial enamine and palladium catalysis.^[14]^ In a seminal publication, Carreira et al. reported diastereodivergent synergistic allylation providing γ,δ‐unsaturated aldehydes bearing vicinal quaternary/tertiary stereocenters employing pseudoenantiomeric cinchona alkaloid derived aminocatalysts and chiral iridium complexes containing antipode phosphoramidite ligands‐each catalyst is able to exert a high degree of local stereocontrol independent of one another.^[15]^ This was followed by the allylic alkylation of linear aldehydes activated through secondary aminocatalysis.^[16]^ Following this approach, we reported an asymmetric diastereodivergent γ‐allylation of exocyclic α,β‐unsaturated aldehydes via a combination of organo‐ and transition metal catalysis.^[17]^

**Scheme 1:**
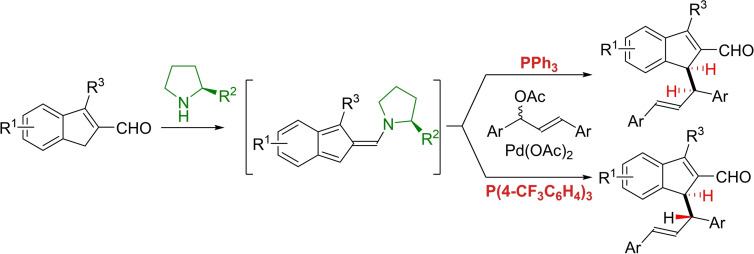
Synergistic catalytic strategy for the diastereodivergent allylation of indene‐2‐carbaldehydes.

The application of reactive conjugated polyene systems derived from (hetero)aromatic compounds has a long history in organic chemistry.^[18]^ Within the last decade, organocatalysis has emerged as a convenient approach to generating these reactive intermediates via *aminocatalytic dearomative* reactions.^[19]^ Recently, complementary *aminocatalytic aromative* reactions have been disclosed. These generate highly‐conjugated reactive systems, having for example 6π‐ or 10π‐electrons, that undergo stereoselective cycloadditions.[^20^, ^21^, ^22^, ^23^] Importantly, these strategies employ unorthodox reactive intermediates affording the potential for novel reaction pathways. In exploring this unique reactivity, we became interested in the potential of pairing two reactive intermediates—a palladium allyl species and an organocatalytically activated conjugated system‐through synergistic catalysis (Scheme 1).

In light of the considerable synthetic potential of reactive conjugated systems in exploring new reactivities and mechanisms, we here disclose a dual catalytic palladium‐allylation reaction of a transient amino isobenzofulvene 10π‐electron system. This reaction has been developed to be diastereodivergent, controlled by the electronic properties of the employed monodentate, achiral ligand, which, to the best of our knowledge, is unprecedented. To further our mechanistic understanding of this remarkable effect, the synthetic development of the reaction concept has been studied applying computational and experimental investigations.

## Results and Discussion

### Development of the archetypal reaction

The reaction between 3‐methyl‐1*H*‐indene‐2‐carbaldehyde **1 a** and diphenylallyl acetate **2 a** facilitated by diphenylprolinol‐based catalysts **3 a**‐**g** and Pd(OAc)_2_ with PPh_3_ was investigated. A number of organocatalysts were screened to improve the stereoselectivity of the reaction; salient results are shown in Table 1. Ether‐ or silyl ether substituted diphenylprolinol catalysts allowed for high conversion within 48 h, but with poor diastereoselectivity. Substitution of the silyl ether with a fluorine atom facilitated the reaction and greatly improved the diastereoselectivity. Generally, high enantiomeric excess is observed for the major diastereomer, while the minor diastereomer exhibits a lower degree of enantioselectivity; the mechanism by which this occurs is not trivial (see below). Addition of molecular sieves predictably halted the reaction by impeding cleavage of the catalyst, while addition of one equivalent of water also proved deleterious. Acid additives were likewise found to retard reactivity. For a presentation of full screening results, see Supporting Information.

**Table 1:** Initial screening of reaction conditions and amine screening.

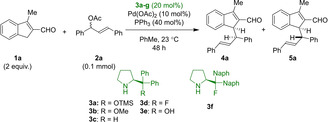						
Entry	Aminocatalyst [mol %]	Pd(OAc)_2_ [mol %]	PPh_3_ [mol %]	Conversion [%]^[a]^	d. r.^[b]^ **4 a** : **5 a**	*ee* ^[c]^ **4 a/5 a** [%]
1	**3 a**	10	40	>95	1.6 : 1	91/23
2	**3 b**	10	40	>95	1.4 : 1	89/25
3	**3 c**	10	40	>95	3 : 1	86/46
4	**3 d**	10	40	>95	8 : 1	98/55
5	**3 e**	10	40	trace	n. d.	n. d.
6	**3 f**	10	40	60	6 : 1	99/61
7	**3 a**	10	0	0	n. d.	n. d.
8	**3 a**	0	40	0	n. d.	n. d.
9	‐	10	40	12	1 : 1.4	n. d.

[a] Evaluated by crude ^1^H NMR based on relative integration of aldehyde signals. [b] Evaluated by relative integration of aldehyde signals on chromatographically pure material. [c] Evaluated by UPCC.

### Effect of ligand modification on stereochemistry

The ligands of chiral metal complexes influence the enantioselectivity of a reaction, but also often exert control over the diastereoselectivity—sometimes unexpectedly. An early example was reported by Shimizu et al. while studying the reaction of dimethyl sodiomalatonate with bicyclo[4.4.0]decane (±)‐2β‐acetoxy‐4aβ‐methyl‐2,3,4,4*a*,5,6‐hexahydronaphthalene using chiral bidentate ligands and a dimeric palladium precatalyst.^[24]^

In the current case, despite their broad application in asymmetric allylation reactions, we were surprised to observe that chiral bidentate ligands impeded reactivity, while simple phosphoramidite ligands facilitated allylation with poor diastereoselectivity‐attempts to use a chiral phosphoramidite failed to give any product (see Supporting Information). Due to the inability of prototypical chiral allylation ligands to efficaciously carry out the desired reaction, we turned to monodentate phosphines. Table 2 presents the influence of a series of monodentate phosphines on the diastereo‐ and enantioselectivity of the allylation reaction of 3‐methyl‐1*H*‐indene‐2‐carbaldehyde **1 a** with allyl acetate **2 a** catalyzed by (fluorodiphenylmethyl)‐pyrrolidine **3 d** ^[25]^ and Pd(OAc)_2_.

**Table 2:** Ligand screening.

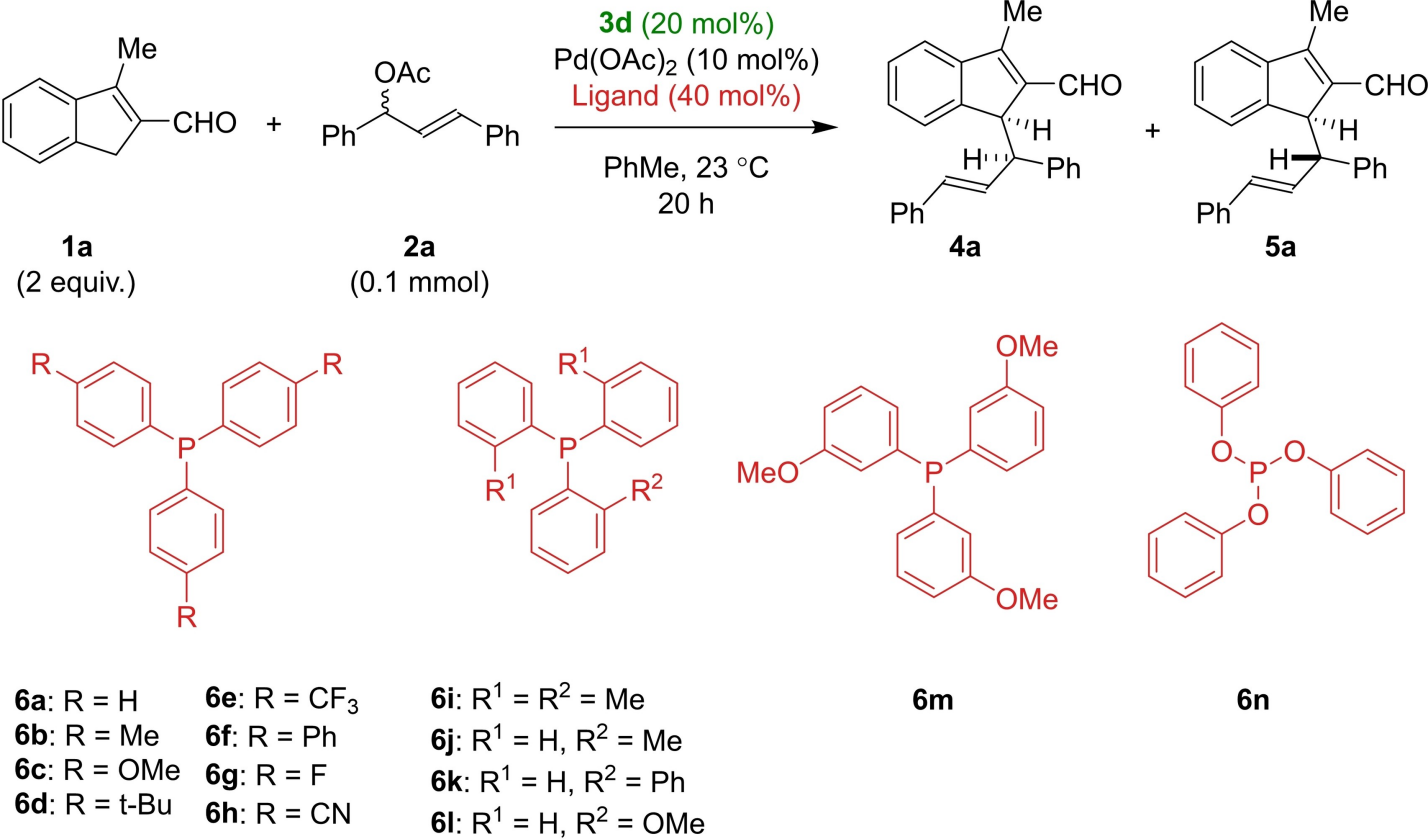				
Entry	Ligand	Conversion^[a]^ [%]	d. r.^[b]^ **4 a** : **5 a**	*ee* ^[c]^ **4 a/5 a** [%]
1	**6 a**	>95	8 : 1	91/58
2	**6 b**	43	5 : 1	90/57
3	**6 c**	38	2.3 : 1	73/8
4	**6 d**	45	8 : 1	87/44
5	**6 e**	>95	1 : 2	90/90
6	**6 f**	35	4.6 : 1	96/73
7	**6 g**	76	1 : 1	93/74
8	**6 h**	15	1 : 2.1	89/98
9	**6 i**	n.r.	n. d.	n. d.
10	**6 j**	70	6 : 1	98/54
11	**6 k**	n.r.	n. d.	n. d.
12	**6 l**	30	5 : 1	94/52
13	**6 m**	35	6 : 1	90/65
14	**6 n**	62	1 : 2	93/76

[a] Evaluated by crude ^1^H NMR based on relative integration of aldehyde signals. [b] Evaluated by relative integration of aldehyde signals on chromatographically pure material. [c] Evaluated by UPCC.

The results using palladium complexes with modified coordination spheres are significant from a number of perspectives. First, they indicate the reaction likely proceeds with a single ligand coordinated to the palladium allyl in the transition state. The sluggish reactivity—and outright lack of reactivity, in cases of rigid ligand scaffolds—observed when using bidentate phosphines compared to the almost uniform success of monodentate ligands may suggest that the ability of the phosphine ligand(s) to disassociate, leaving only a single phosphine ligand bound to the metal, is important. Significantly, a conspicuous electronic effect of the *achiral* monodentate phosphine ligand coordinated to palladium on the diastereoselectivity of the reaction is apparent (Table 2). Electron‐poor ligands **6 e**, **6 h** and **6 n** displayed a fundamental inversion in diastereoselectivity. Further, the ligand was able to modulate the reaction enantioselectivity (e. g. Table 2, entries 1 and 5). Whereas ligand electronics predominate, steric effects are not wholly absent, as evidenced by significant differences in conversion (Table 2, entries 10, 13). These observations, and the ineffectiveness of bidentate ligands, suggest that the reaction is quite sensitive to the steric bulk of the ligand.

Importantly, examples wherein asymmetric stereoinduction is reversed solely by tuning the electronic properties of the ligand exist but are very rare‐notably, these sparse reports all employ chiral ligands.[^26^, ^27^, ^28^] While systematically studying *C*_2_‐symmetric ligands on the prototypical reaction of 1,3‐diphenylallyl with a malonate nucleophile, RajanBabu et al. observed inversion of enantioselectivity by modulating the electronics of the same chiral ligand backbone. The reversal in stereoselectivity was rationalised, after comprehensive NMR studies, to be the result of nucleophilic attack at the allylic carbon, which is *trans* to the more deshielded and π‐accepting phosphorus atom. However, this does not provide a satisfactory explanation in the current case, as invocation of a (*syn*,*syn*)/(*syn*,*anti*) equilibrium[^26^, ^29^, ^30^] cannot be excluded (see below).

**Figure 1:**
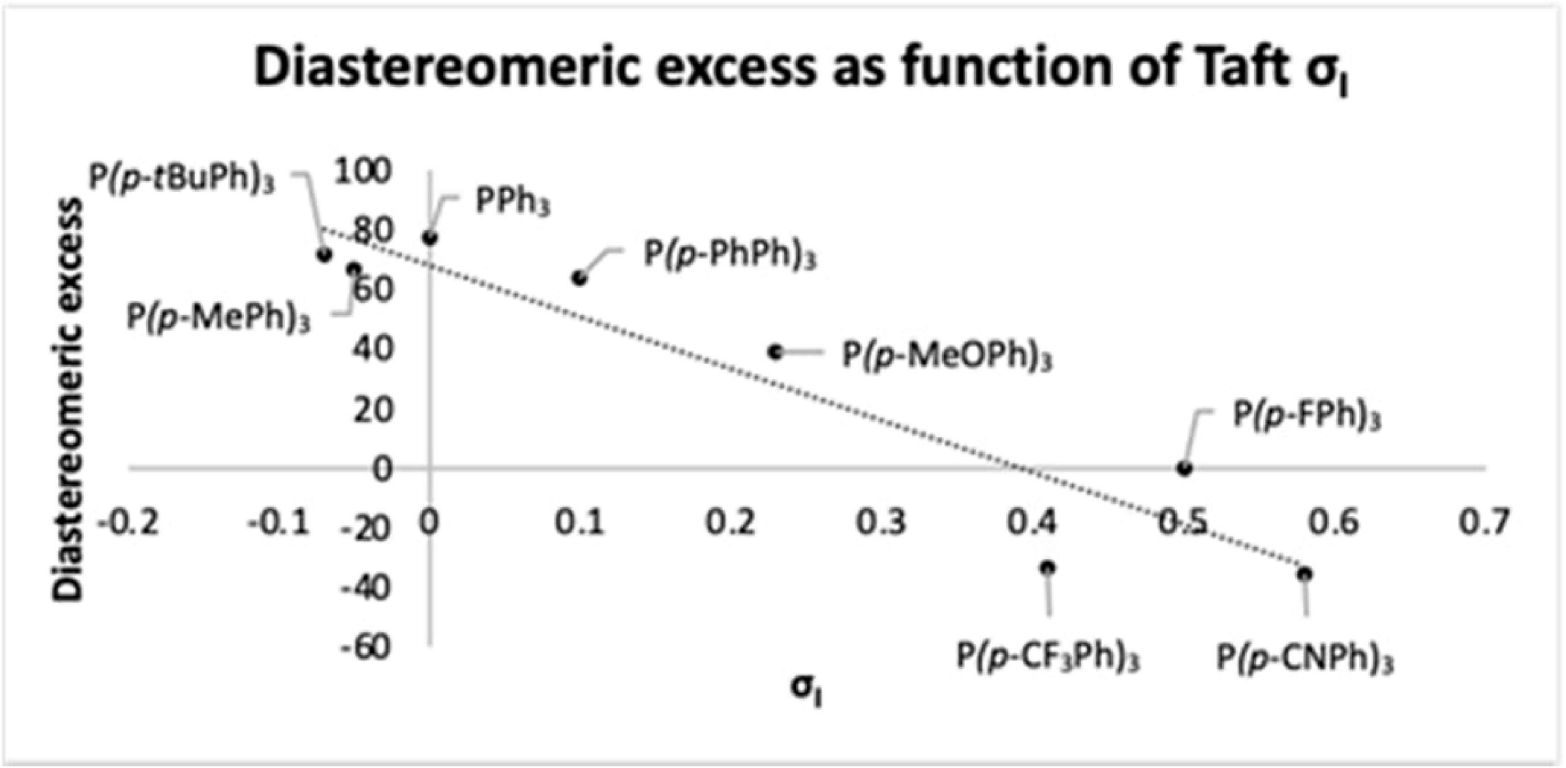
Influence of inductive effects (σ_I_) of phosphine ligands on the diastereoselectivity (toward **4 a**) of the allylation reaction.

We became keenly interested in the mechanistic origins of this phenomenon. Initial investigations applying steric parameters‐Tolman angle, percent volume buried‐to explain the observed changes in the diastereoselectivity proved futile. The electronic component of reactions is commonly described by Hammett values (σ_p_) which include both resonance (σ_R_) and inductive (σ_I_) terms.^[31]^ The equation, σ_p_=σ_R_+σ_I_, was first formulated by Taft and Lewis.^[32]^ Interestingly, in the current case, a good correlation between diastereoselectivity and the nature of the applied ligands was only possible by extracting the Taft linear inductive component (σ_I_) from the overall Hammett equation (for a comparison of *p*‐substituted triaryl phosphines, see Figure 1). To test the validity of this relationship, we synthesized *tris*(4‐cyanophenyl)phosphine **6 h**, which has an extreme σ_I_ value of +0.59;^[32]^ this ligand fit the trend line nicely, delivering **4 a** with the highest observed selectivity, but with low conversion.

### Diastereodivergence control

Despite advances, most enantioselective reactions which furnish compounds with multiple stereogenic centres are often limited to the synthesis of a select set of stereoisomers.^[33]^ While generally, the cognate enantiomer of a product generated through asymmetric catalysis may be accessed by employing the mirror‐image of the catalyst, most reactions are not amenable to modulating diastereoselectivity. Superseding the intrinsic diastereoselectivity of a reaction by means of *a single chiral catalyst* remains a great challenge since the spatial positions of both reaction partners needs to be controlled.

For the formation of molecules with multiple stereocenters, the mechanism(s) by which chirality is transferred in diastereodivergent processes is clearly of interest in order to facilitate exploration of new chemical space and reactions. In the limited number of methods which allow the preparation of all possible stereoisomers from a single set of precursors, stereodivergence is typically the result of significant modification to the structure of the catalyst or reaction conditions;^[34]^ so much so, in fact, that these strategies cannot be broadly applied.

The current reaction employs a single chiral organocatalyst in tandem with an *achiral* mesomeric palladium intermediate. While the organocatalyst governs the absolute sense of stereoinduction, the ligands on palladium exert a large degree of local stereocontrol simply through modulation of the electronic properties of *achiral* monodentate phosphine ligands coordinated to palladium‐a unique observation to the best of our knowledge. Though the enantiomer of **5 a** is accessible by switching to the antipode organocatalyst, the ability of inverting the relative stereochemistry on the allyl by switching between triphenylphosphine **6 a** and *tris*(4‐trifluoromethylphenyl)phosphine **6 e** allows an extra dimension of stereocontrol. Thus, all four stereoisomers are accessible.

### Improving the diastereoselectivity of the reaction using tris(4‐trifluoromethylphenyl)phosphine as ligand

Having established a set of conditions which favored the formation of diastereomer **4 a** (*S*,*R‐*configuration), we sought to improve the modest diastereoselectivity through a secondary screening of aminocatalysts (Table 3 and Supporting Information). Diphenylprolinol silyl ethers were found to be most effective, improving the diastereoselectivity with only a slight decrease in the observed enantioselectivity (Table 3, entries 1, 5–7). Catalyst **3 a** proved to be the most reactive and diastereoselective catalyst and was therefore chosen for investigating the scope and selectivity of this set of conditions. Traces of by‐product formation were present, although the yields were not impacted to any significant extent. In all cases, a marked preference for the formation of product diastereomer **5 a** was observed when using *tris*(4‐trifluoromethylphenyl)phosphine **6 e** as ligand.

**Table 3:** Optimization of the allylation reaction of **1 a** towards product **5 a**.

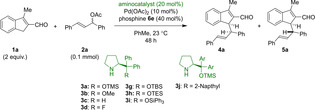				
Entry	Ligand	Conversion [%]^[a]^	d. r.^[b]^ **4 a** : **5 a**	*ee* ^[c]^ **4 a/5 a** [%]
1	**3 a**	>95	1 : 2.8	82/85
2	**3 b**	>95	1 : 1.8	79/77
3	**3 c**	>95	1 : 1.8	n. d.
4	**3 d**	>95	1 : 2	91/90
5	**3 g**	60	1 : 2.2	86/88
6	**3 h**	80	1 : 2.6	87/84
7	**3 i**	50	1 : 2.2	94/94
8	**3 j**	trace	1 : 1	n. d.

[a] Evaluated by crude ^1^H NMR based on relative integration of aldehyde signals. [b] Evaluated by relative integration of aldehyde signals on chromatographically pure material. [c] Evaluated by UPCC.

### Synergistic reactions of substituted indenes and substituent effects

With optimized reaction Conditions A and B for the formation of products **4** or **5**, respectively, the tolerance for employing substrates with various substituents was investigated. Gratifyingly, indene carbaldehydes carrying diverse substituents could be employed in the developed reaction, giving consistently high yields (Table 4). Among these, substrates carrying halogen, alkyl, or electron‐rich heteroatom substituents were found to be compatible, and all positions except C7 (adjacent to the functionalized indene center) were successfully modified. *Importantly, Conditions A in all cases rendered adduct **4** the major diastereoisomer, while Conditions B rendered adduct **5** the major diastereoisomer*. The reaction therefore is ubiquitously stereodivergent; the choice of achiral monodentate triarylphosphine ligand on palladium dictates the diastereoselectivity of the reaction, while the aminocatalyst‐as the only chiral additive‐controls the enantioselectivity of the reaction. The highest degree of diastereodivergency was obtained for 5‐methoxysubstituted adducts **4 g** (entry 13, 8 : 1 d. r. under Conditions A) and **5 g** (entry 14, 1 : 6.2 d. r. under Conditions B). In addition to the observed diastereodivergency, it was generally observed that the enantioselectivity, with which the diastereoisomers were formed, was found to be higher when diastereoselectivity favored it; adducts **4** were formed with higher enantioselectivity using Conditions A than Conditions B, and could typically be obtained with ≥90 % *ee*, while adducts **5** were formed with higher enantioselectivity using Conditions B than Conditions A. Interestingly, Conditions A and B in some cases produced opposite enantiomers of **5** (Table 4, entries 5/6, 9/10, 17/18, 19/20, 21/22, 23/24, 25/26). It is of note that while 3‐alkylsubstituted indene carbaldehydes were employed in enantioselective transformations, 3‐phenylindene carbaldehyde (not shown) was too reactive, as its functionalization proceeded rapidly even in the absence of an aminocatalyst.

**Table 4:** Scope of the allylation reaction toward products **4** or **5** using various indene carbaldehydes **1**.

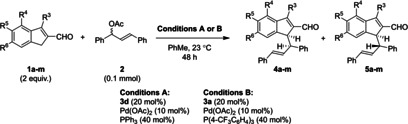									
Entry	R^3^	R^4^	R^5^	R^6^	Products	Conditions	Yield [%]^[a]^	d. r.^[b]^ **4 : 5**	*ee* ^[c]^ [%] **4/5**
1	**Me**	H	H	H	**4 a** : **5 a**	**A**	83	10 : 1	96/57
2						**B**	76	1 : 2.7	83/82
3	**Et**	H	H	H	**4 b** : **5 b**	**A**	79	4.2 : 1	83/68
4						**B**	69	1 : 2.2	94/87
5	**Cy**	H	H	H	**4 c** : **5 c**	**A**	67	2.4 : 1	90/−40
6						**B**	53	1 : 1.3	88/33
7	Me	**OMe**	H	H	**4 d** : **5 d**	**A**	76	2.9 : 1	92/0
8						**B**	71	1 : 2	69/75
9	Me	**F**	H	H	**4 e** : **5 e**	**A**	68	2.4 : 1	89/−36
10						**B**	69	1 : 2	81/63
11	Me	H	**Me**	H	**4 f** : **5 f**	**A**	85	7.4 : 1	98/66
12						**B**	73	1 : 4.7	87/94
13	Me	H	**OMe**	H	**4 g** : **5 g**	**A**	92	8 : 1	97/76
14						**B**	57	1 : 6.2	97/98
15	Me	H	H	**Me**	**4 h** : **5 h**	**A**	88	5.1 : 1	95/70
16						**B**	68	1 : 2.7	84/82
17	Me	H	H	**OMe**	**4 i** : **5 i**	**A**	94	3.6 : 1	93/−8
18						**B**	88	1 : 2.9	72/69
19	Me	H	H	**F**	**4 j** : **5 j**	**A**	95	2.3 : 1	94/−60
20						**B**	84	1 : 2.8	66/67
21	Me	H	H	**Cl**	**4 k** : **5 k**	**A**	78	2.4 : 1	92/−30
22						**B**	87	1 : 2.3	54/70
23	Me	H	H	**Br**	**4 l** : **5 l**	**A**	79	2.2 : 1	90/−14
24						**B**	78	1 : 2.5	58/71
25	Me	H	H	**SMe**	**4 m** : **5 m**	**A**	87	2.2 : 1	93/−19
26						**B**	84	1 : 3.1	69/69

[a] Isolated yield. [b] Evaluated by relative integration of aldehyde signals on chromatographically pure material. [c] Evaluated by UPCC.

### Synergistic reactions of the substituted allyl acetates and substituent effects

Subsequently, the reaction scope was investigated through employing various precursors for the catalytically generated allylpalladium component (Table 5). Application of various diarylallyl acetates underpinned the generality of the reaction diastereodivergency, as Conditions A in all cases produced **4** as the major diastereoisomer with ≥90 % *ee*, while Conditions B in all cases favored **5**, with 77–90 % *ee*.

**Table 5:** Scope of the allylation reaction toward products **4** or **5** using various allyl acetates **2**.

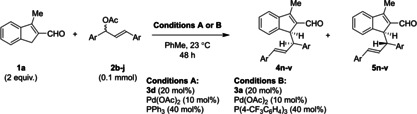						
Entry	Ar	Products	Conditions	Yield [%]^[a]^	d. r.^[b]^ **4 : 5**	*ee* ^[c]^ [%] **4 : 5**
1	4‐MeC_6_H_4_	**4 n** : **5 n**	**A**	83	10 : 1	96 : 57
2			**B**	76	1 : 2.7	83 : 82
3	4‐(OMe)C_6_H_4_	**4 o** : **5 o**	**A**	79	4.2 : 1	83 : 68
4			**B**	69	1 : 2.2	94 : 87
5	4‐FC_6_H_4_	**4 p** : **5 p**	**A**	67	2.4 : 1	90 : −40
6			**B**	53	1 : 1.3	88 : 33
7	4‐ClC_6_H_4_	**4 q** : **5 q**	**A**	76	2.9 : 1	92 : 0
8			**B**	71	1 : 2	69 : 75
9	4‐BrC_6_H_4_	**4 r** : **5 r**	**A**	68	2.4 : 1	89 : −36
10			**B**	69	1 : 2	81 : 63
11	4‐CF_3_C_6_H_4_	**4 s** : **5 s**	**A**	85	7.4 : 1	98 : 66
12			**B**	73	1 : 4.7	87 : 94
13	4‐NO_2_C_6_H_4_	**4 t** : **5 t**	**A**	92	8 : 1	97 : 76
14			**B**	57	1 : 6.2	97 : 98
15	3‐ClC_6_H_4_	**4 u** : **5 u**	**A**	88	5.1 : 1	95 : 70
16			**B**	68	1 : 2.7	84 : 82
17	3‐BrC_6_H_4_	**4 v** : **5 v**	**A**	94	3.6 : 1	93 : −8
18			**B**	88	1 : 2.9	72 : 69

[a] Isolated yield. [b] Evaluated by relative integration of aldehyde signals on chromatographically pure material. [c] Evaluated by UPCC.

Notably, the diastereoselectivity with which the developed allylation proceeds is often low. Across the 44 entries of Tables 4 and 5, the highest d. r. was obtained for **4 n** using Conditions A (10.5 : 1, Table 5, entry 1), while the lowest d. r. was obtained for **5 o** under Conditions B (1.2 : 1, Table 5, entry 4). These values indicate that only small energetic differences exist between the pathways toward each diastereoisomer, and it is remarkable that *no exceptions* have been found to the diastereodivergency of this reaction. Understanding these trends obtained using monodentate, achiral phosphine ligands, may assist in the future development of diastereodivergent reactions which can rely on the electronic nature of simple ligands, and therefore a rationale to the obtained results became our focus.

### Mechanistic considerations and frontier molecular orbital applications

In attempts to explore the unprecedented role of achiral monodentate phosphine ligands on palladium in controlling the diastereoselectivity of an enantioselective allylation reaction, the reaction mechanism for the formation of the two different diastereoisomers **4 a** and **5 a**, and the origins of diastereo‐ and enantioselectivities, were investigated computationally. The reaction between indene‐2‐carbaldehyde **1 a** and diphenylallyl acetate **2 a** in the presence of organocatalyst **3 a** and Pd(PPh_3_)_2_ was studied computationally using DFT (ωB97X‐D/6‐311++G(d,p)/SDD with SMD (toluene) solvation model on B3LYP/6‐31G(d)/Lanl2dz calculated geometries; see Supporting Information for details).[^35^, ^36^, ^37^, ^38^, ^39^, ^40^, ^41^, ^42^, ^43^] These conditions were chosen to most accurately describe the reaction using Conditions A (see above).

**Figure 2:**
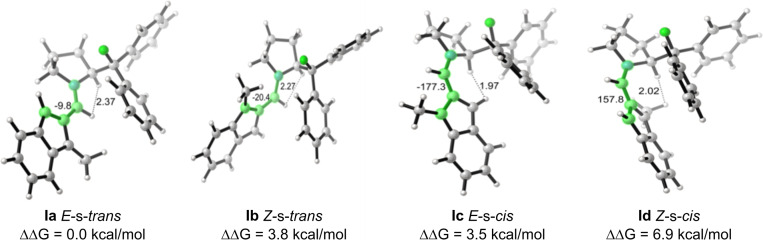
Four optimized conformations of the enamine intermediates **Ia**–**d**. Dihedral angle given in degrees and highlighted in green; distances given in Å.

First, indene‐2‐carbaldehyde **1 a** reacts with the secondary amine catalyst, diphenylprolinol‐based **3 a** (*S*‐configuration), forming the enamine isobenzofulvene intermediate **I**. As **3 a** is the sole enantioenriched species employed in the reaction setup, the geometry of the resulting enamine **I** is crucial to the stereochemical outcome of the reaction. The diphenylfluoromethyl group was calculated in all cases to be arranged with the C−F bond *anti*‐periplanar to the C−H bond at the chiral center of the catalyst. Therefore, only four unique enamines **I** were located, differentiated by configuration of the enamine olefin and conformation of the enamine C−N bond; E‐*s‐trans* **Ia**, Z‐*s‐trans* **Ib**, E‐*s‐cis* **Ic**, and Z‐*s‐cis* **Id**. We began our investigation by comparing the computed relative stabilities of different enamine intermediate geometries (Figure 2).

The most stable enamine intermediate was found to be the *E*‐s‐*trans* aminofulvene **Ia**. The instabilities of the remaining geometries can be attributed to steric repulsion and/or torsional strain. In the structure of *E*‐s‐*trans* aminofulvene **Ia**, the dihedral angle of the enamine (highlighted in green, Figure 2) is −10° and the distance between two potentially clashing hydrogens (Figure 2) is determined as 2.37 Å. For *Z*‐s‐*trans* aminofulvene **Ib**, a twisted dihedral angle of 20° destabilizes this configuration resulted in a relatively higher energy than **Ia** by 3.8 kcal/mol. Differently, in the structure of *E*‐s‐*cis* aminofulvene **Ic**, destabilization may be inferred from H−H steric repulsion with the distance of 1.97 Å, raising the relative energy by 3.5 kcal/mol. For the calculated structure of Z‐s‐*cis* aminofulvene **Id**, both a large dihedral angle of 22° and the H−H steric repulsion contribute to a further increased relative energy of 6.9 kcal/mol. The analysis of the organocatalytically generated amino isobenzofulvene geometry allowed for predicting which face of the 10π‐system was most accessible for the subsequent allylation reaction, determining the stereochemistry of the forming stereocenter on the indene core.

**Figure 3:**
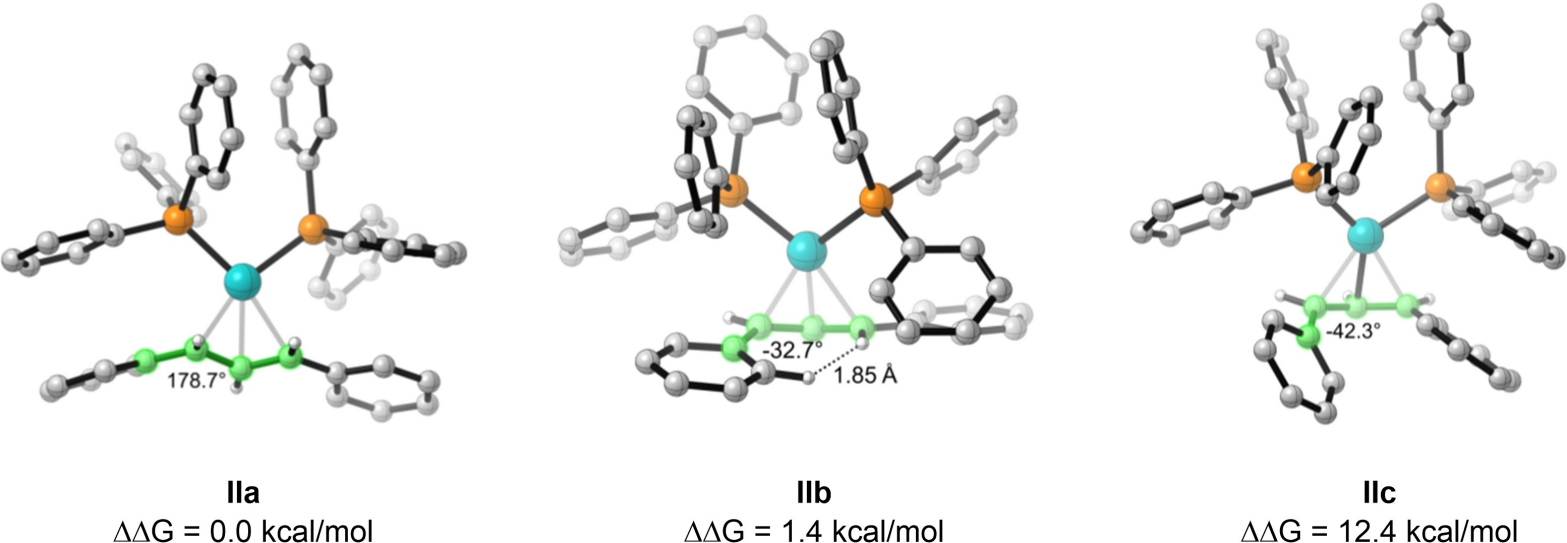
The three lowest‐energy conformations of the diphenylallyl‐Pd(PPh_3_)_2_ complex **II**.

The second stereocenter formed during the reaction is situated on the allyl fragment, and therefore the geometry of this species during the reaction is likewise important. The three lowest‐energy configurations of the diphenylallyl‐Pd(PPh_3_)_2_ complex **II** are shown in Figure 3. The relative free energies are 0.0, 1.4, and 12.4 kcal/mol, in favor of the *E*,*E*‐configured intermediate **IIa**. The differences in energy appear to arise from hydrogen‐hydrogen repulsions from H−H distances of 1.85 Å observed in the intermediate structure **IIb**, causing a distortion in dihedral angle (highlighted in green) to 33°, leading to a higher relative energy. Finally, for complex **IIc**, a large distortion of 42° (highlighted in green) causes a further increase in energy.

**Figure 4:**
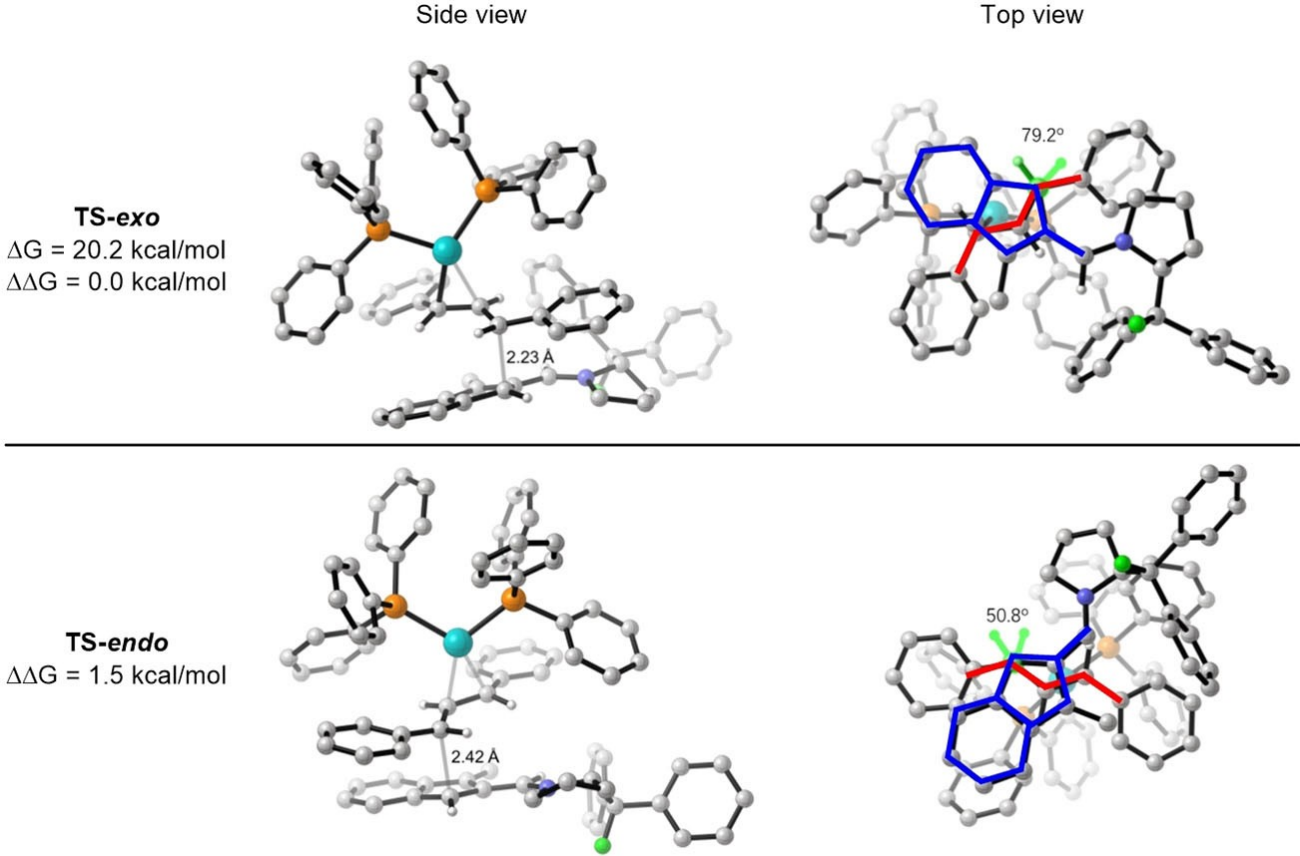
Transition state structures for the *endo‐* and *exo*‐pathways from *E*‐s‐*trans*‐**Ia** reacting with diphenylallyl‐Pd(PPh_3_)_2_ complex **IIa**. For the top view (right), indenyl and allyl moieties are highlighted in blue and red for clarity.

We next explored the transition state structures for the functionalization of indene‐2‐carbaldehyde between *E*‐s‐*trans* aminofulvene **Ia** and the *E*,*E*‐diphenylallyl‐Pd(PPh_3_)_2_ complex **IIa**. The π‐interaction between the allyl moiety and the incoming nucleophile of isobenzofulvene intermediate **Ia** plays an important role in the formation of transition state structures, and the most stable transition state structures of *exo‐* and *endo*‐pathways are shown in Figure 4. The lowest reaction barrier was calculated to be the formation of the *exo*‐product (via **TS‐*exo***, leading to the *S*,*S*‐product **4 a**) with a relative free energy of 20.2 kcal/mol compared to separated species. The **TS‐*exo*** is 1.5 kcal/mol lower in energy than **TS‐*endo*** toward *S*,*R*‐product **5 a**. This is in accordance with the experimental observations (12 : 1 calculated d. r. vs. 10 : 1 experimental d. r.). The energetic difference between these two transition state structures does not appear to be a result of steric clash, and therefore a rationale is proposed employing FMO theory, taking into account π‐π interactions between the isobenzofulvene and allyl moieties (see below).

**Figure 5:**
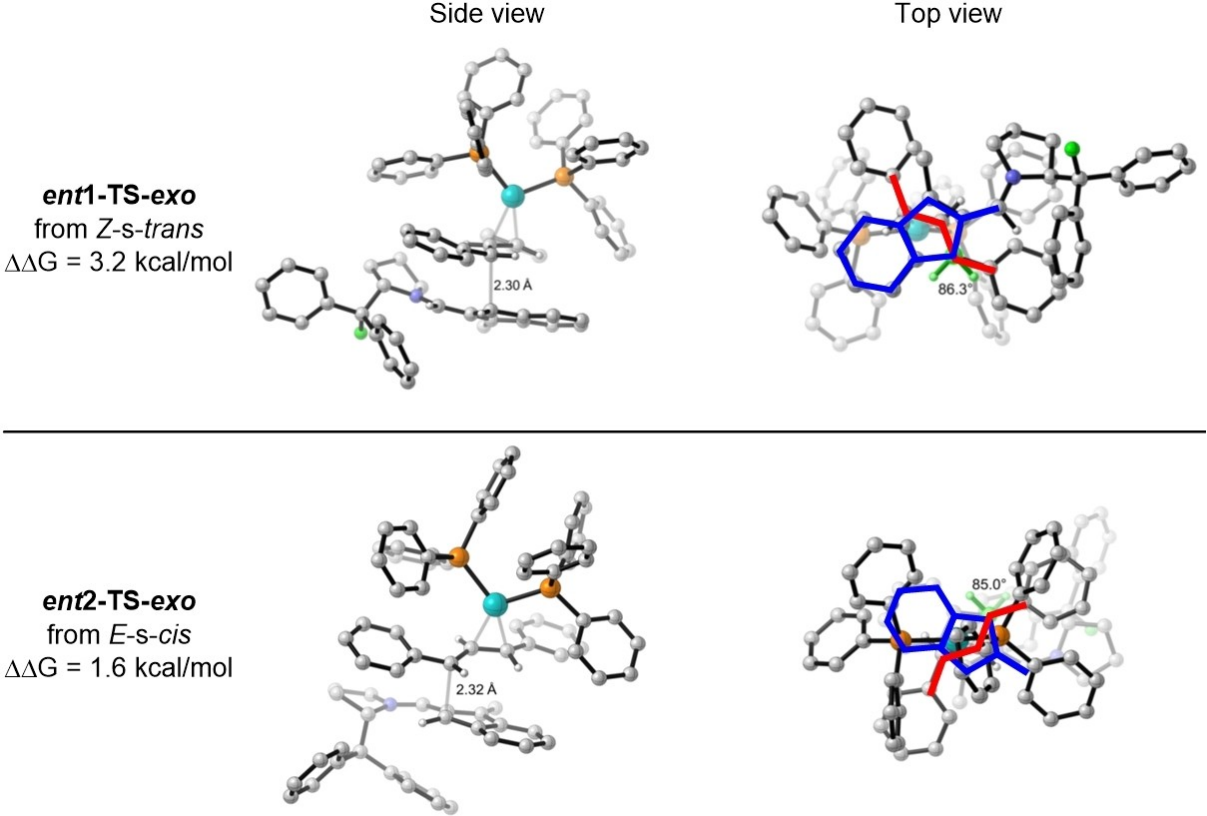
Transition state structures from the *exo*‐conformations from *Z*‐s‐*trans*‐**Ib** and *E*‐s‐*cis‐* **Ic** with *E*,*E*‐diphenylallyl‐Pd(PPh_3_)_2_ complex **IIa**.

In order to rationalize the high enantioselectivity of the allylation reaction under Conditions A, transition state structures which would lead to the formation of ***ent*** **‐4 a** were located. In these calculations, the intermediates of *Z*‐s‐*trans* aminofulvene **Ib** and *E*‐s‐*cis* aminofulvene **Ic** were considered, as they shield the opposite face of the indenyl moiety compared to the more stable **Ia**. Similarly, the π‐π interaction between the allyl moiety and the incoming nucleophile of isobenzofulvene intermediate **Ib** or **Ic** also plays an important role in the formation of the product arising from these enantiomeric pathways; the most stable transition state structures for the allylation step between **Ib** or **Ic** and **IIa** are shown in Figure 5. Of these, ***ent*** **1‐TS‐*exo*** from **Ib** was found to be 3.2 kcal/mol higher in energy than **TS‐exo**, while ***ent*** **2‐TS‐*exo*** from **Ic** was found to be only 1.6 kcal/mol higher in energy than **TS‐exo**. The latter energetic difference is in accordance with the experimental observations (88 % calculated *ee* vs. 96 % experimental *ee*).

**Figure 6:**
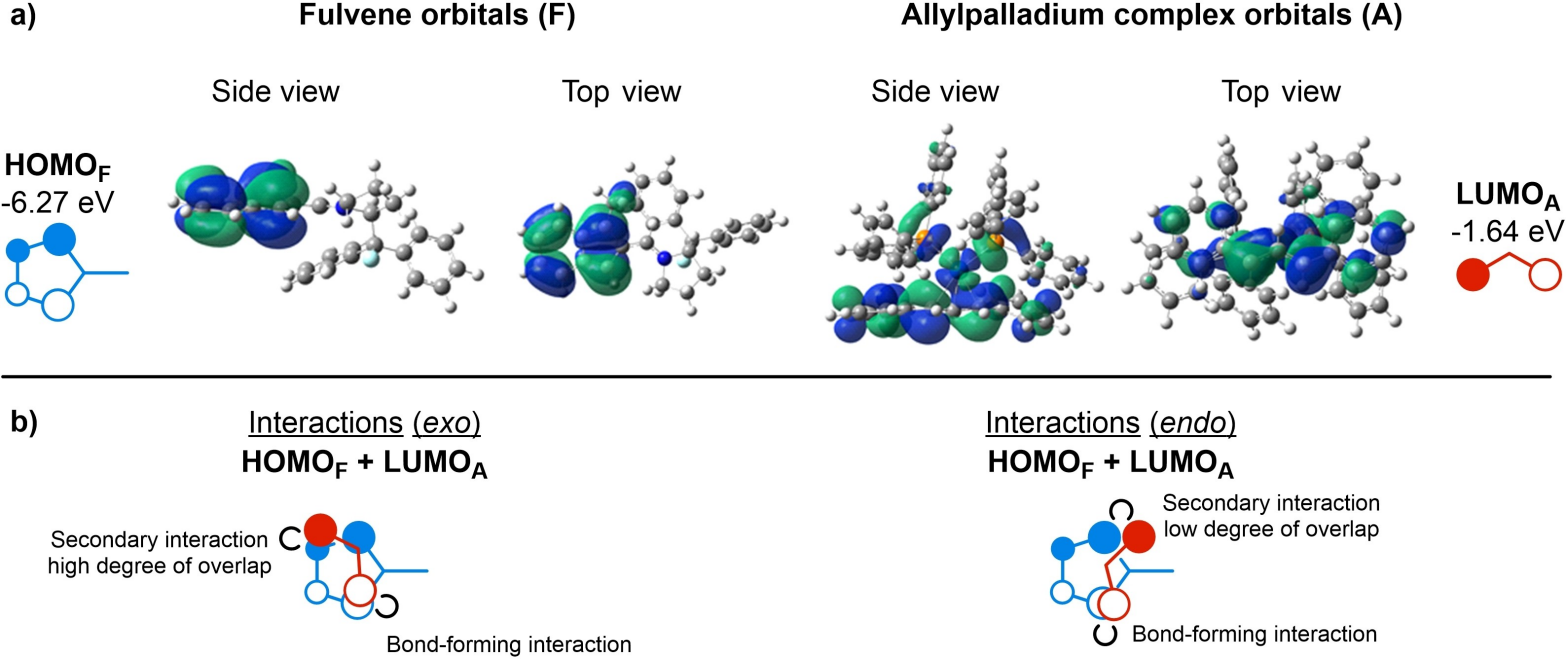
a) HOMOs of fulvene and LUMO of allyl cation. b) FMO interactions for the interactions leading to the two different diastereoisomers **4** and **5**, respectively, based on an *exo*‐ and *endo*‐approach, respectively, for electron‐rich and electron‐poor phosphine ligands.

With convincing calculated geometries for the formation of **4** and **5** under Conditions A in hand, a rationale for the observed inversion in diastereoselectivity upon phosphine ligand switch was sought. While efforts to compute the key transition state for the allylation reaction under Conditions B were not fruitful, two plausible explanations of stereoselectivity are proposed: inductively electron‐withdrawing substituents on a phosphine ligand (such as the CF_3_‐substituents of **6 e** employed under Conditions B) should cause the phosphine moiety to become a poor electron donor, and thus a poor ligand for coordination to palladium. Therefore, a change in ligand stoichiometry‐for example, two phosphine ligands or one phosphine ligand and one acetate ligand‐could entail a change in steric bulk of the allylpalladium species, leading to a change in the energetic barriers in favor of an *endo*‐pathway to produce **5** as the major diastereoisomer. However, the gradual change in the preference of **5** over **4** using phosphine ligands substituted with increasingly electron‐poor substituents suggests a more subtle change in the reaction course. The energies of the allylpalladium FMOs change based on the electron density provided by the ligated palladium species. Based on this, we propose that changes in secondary π‐orbital interactions between the two π‐components are important for the reaction diastereoselectivity (Figure 6).

The FMO analysis commenced by calculating the HOMO_F_ of the amino isobenzofulvene **Ia**, and the LUMO_A_ of allylpalladium complex **IIa^[^** ^44]^ (Figure 6a). Considering these FMOs and the geometries computed (Figure 4), the *exo*‐pathway toward adduct **4** entails two positive interactions between HOMO_F_ and LUMO_A_, at the nuclei between which bond formation takes place as well as a secondary interaction (Figure 6b). These interactions in **TS‐*exo*** appear to be highly important to the observed preference for the *exo*‐pathway toward **4** upon usage of PPh_3_ as ligand, as no steric preference for this pathway is apparent (Figure 4). In **TS‐*endo*** toward **5**, these FMO interactions are weaker due to much poorer overlap, meaning that secondary orbital overlap must contribute less to the stabilization of this TS (Figure 4). It is surmised that the higher electrophilicity of the allylpalladium species ligated by *tris*(4‐trifluoromethylphenyl)phosphine **6 e** leads to a lack of necessity for secondary orbital interactions or organized TS, so that selectivity decreases and a small preference for TSs leading to **5** emerges. Thus, the *exo*‐pathway may be favored upon usage of PPh_3_ due to secondary orbital interactions, but using ligands such as **6 e** may cause the lowering of the energetic barrier associated with **TS‐*endo*** to a degree where an inversion in diastereoselectivity is observed.

## Conclusion

An unprecedented synergistic amino‐ and palladium catalyzed, diastereodivergent allylic alkylation reaction of indene‐2‐carbaldehydes reacting with diarylallyl acetates has been developed in which the electronic properties of the employed monodentate, achiral phosphine ligand have been found to be crucial to the diastereoselectivity. Herein, enantioselectivity is dictated by the sole chiral species, a secondary aminocatalyst, while diastereoselectivity is governed by the electronic properties of the substituents on the phosphine ligand on palladium. A correlation between phosphine ligand substituents and Taft σ_I_ values suggested inductive effects to be highly important to the diastereomeric outcome of the reaction; inductively withdrawing substituents promotes the formation of the *endo*‐product, in contrast to unsubstituted triphenylphosphine. Two conditions have been optimized to give opposite diastereoisomers, showing the generality of the diastereodivergence. The reaction concept has been demonstrated for 44 reactions, with up to 98 % *ee* for each diastereoisomer, and in all cases the diastereoselectivity was controlled through the choice of phosphine ligand. In order to understand the mechanism, and to rationalize the unprecedented effect of the achiral, monodentate phosphine ligands on the diastereoselectivity, computational studies were performed. For the system employing triphenylphosphine as ligand on palladium, computational studies were in agreement with the observed enantio‐ and diastereoselectivity. An FMO analysis provided a basis for discussing the role of the achiral ligand on the diastereoselectivity. We believe that understanding how the electronic nature of simple ligands influence reactive intermediates may prove valuable in the future development of stereocontrolled reactions.

## Conflict of interest

The authors declare no conflict of interest.

1

## Supporting information

As a service to our authors and readers, this journal provides supporting information supplied by the authors. Such materials are peer reviewed and may be re‐organized for online delivery, but are not copy‐edited or typeset. Technical support issues arising from supporting information (other than missing files) should be addressed to the authors.

Supporting Information

## Data Availability

The data that support the findings of this study are available in the supplementary material of this article.
