# Supplementary material for: Influence of Achiral Phosphine Ligands on a Synergistic Organo‐ and Palladium‐Catalyzed Asymmetric Allylic Alkylation
Source: Chemistry. 2022 Oct 27;28(71):e202202951. doi: 10.1002/chem.202202951 (PMC10091967; doi:10.1002/chem.202202951)
Supplement: Supplementary file 1 — Supporting Information [file CHEM-28-0-s001.pdf]

Chemistry–A European Journal

Supporting Information

Influence of Achiral Phosphine Ligands on a Synergistic Organo- and Palladium-Catalyzed Asymmetric Allylic Alkylation

David McLeod, Nicolaj Inunnguaq Jessen, Thanh V. Q. Nguyen, Marcus Espe, Jeremy David Erickson, Karl Anker Jørgensen,* Limin Yang,* and K. N. Houk*

This document is separated into four parts:

Part 1: Experimental Procedures and Characterization Data S2
Part 2: NMR Spectra S50
Part 3: UPCC Spectra S124
Part 4: Computational data S171

Part 1: Experimental Procedures and Characterization Data

Table of Contents

General considerations S3

Table S1. Selected catalyst screening experiments S4

Table S2. Selected ligand screening experiments S5

Table S3. Selected organocatalyst screening experiments with tris(*p*-trifluoromethylphenyl)phosphine S6

Experimental procedures S7

- Synthesis and characterisation of substrates S7
- Representative general procedure I for the preparation of indene-2-carbaldehydes **1a,f**: S7
- Representative general procedure II for the preparation of indene-2-carbaldehydes **1b-e,h-m**: S7
- Representative general procedure III for the preparation of diarylallyl acetates **2a-j**: S13

Procedures for diastereodivergent allylation and characterization of products S17

- Preparation of allylation racemates S17
- General procedures for the diastereodivergent allylation of indene-2-carbaldehydes........ S17

General considerations

Reactions were carried out under nitrogen or argon atmosphere with dry solvents using anhydrous conditions unless otherwise stated. Tetrahydrofuran (THF), was distilled from Na^0 with benzophenone as an indicator. Toluene (PhMe) was dried over molecular sieves (4Å) and then degassed by four sequential freeze-pump-thaw cycles. All fine chemicals were obtained from Sigma-Aldrich, Alfa-Aesar or TCI, and used without further purification unless otherwise stated. Reactions were monitored by thin layer chromatography (TLC) on silica gel pre-coated aluminum-backed sheets (0.2 mm, Merck silica gel 60 F_{254}). Visualization was accomplished by irradiation with UV light at 254 nm and/or 2,4-dintrophenylhydrazine (2,4-DNP) stain. 2,4-DNP stain: to 95% EtOH (100 mL) were added conc. sulfuric acid (30 mL), 2,4-dintrophenylhydrazine (6 g) and water (40 mL). Yields refer to chromatographically and spectroscopically (^{1}H-NMR) homogeneous materials, unless otherwise stated. NMR spectra were recorded on a Varian AS400 MHz spectrometer and calibrated using residual undeuterated solvent as an internal reference ($CHCl_3$ @ δ 7.26 ppm ^{1}H NMR, δ 77.16 ppm ^{13}C NMR). Chemical shifts (δ) for ^{19}F NMR are reported in ppm relative to C_6H_5F as external reference calibrated to -115.3 ppm ($CDCl_3$). The following abbreviations are used to indicate the multiplicity in NMR spectra: s, singlet; d, doublet; t, triplet; q, quartet; m, multiplet; bs, broad signal. ^{13}C NMR and ^{19}F NMR spectra were acquired in broad band decoupled mode. Signal assignments were accomplished *via* analysis of HMBC, HSQC, COSY, and NOESY experiments where necessary. Diastereomeric ratios (dr) were determined from the relative integration of the ^{1}H spectra for the aldehyde and methyl protons of chromatographically pure material. Enantiomeric excess (ee) were determined by Ultraperformance Convergence Chromatography UPCC analysis employing a chiral stationary phase column specified in the individual experiment, by comparing the samples with the appropriate racemic mixtures. Mass spectra were recorded on a Bruker Maxis Impact mass spectrometer using electrospray ionization (ESI+). Optical rotations were determined with a Bellingham+Stanley ADP440+ polarimeter at 589 nm and 25 °C. Data are reported as follows: [α]λ temp, concentration (c; g/100 mL), and solvents. All melting points are corrected.

Table S1. Selected catalyst screening experiments

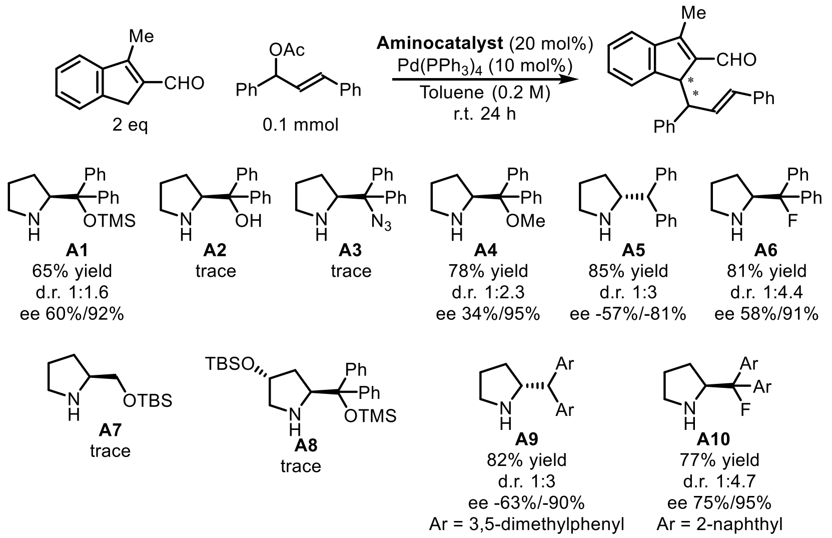

Table S2. Selected ligand screening experiments

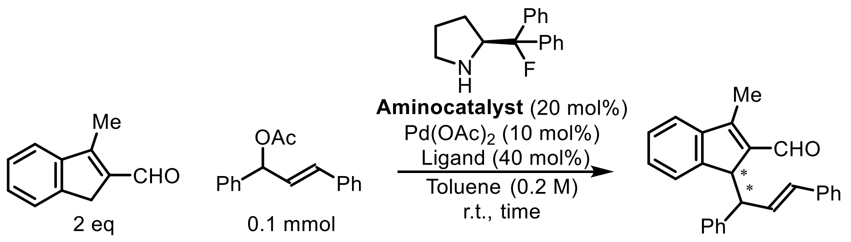

Ligand	time (h)	Conversion	d.r.	*ee*(%)
-	nr	nr	nr	nr
PCy_3	20	nr	nr	nr
PEt_3	20	55	45/55	13/15
dppf	20	30	1/3.0	32/88
dppe	68	15	1/1.0	3/50
(*S*)-BINAP	20	nr	nr	nr
$P(3,5\text{-}CF_3Ph)_3$	nr	nr	nr	nr
$P(C_6F_5)_3$	nr	nr	nr	nr
$P(Naphth)_3$	nr	nr	nr	nr

Table S3. Selected organocatalyst screening experiments with tris(*p*-trifluoromethylphenyl)phosphine

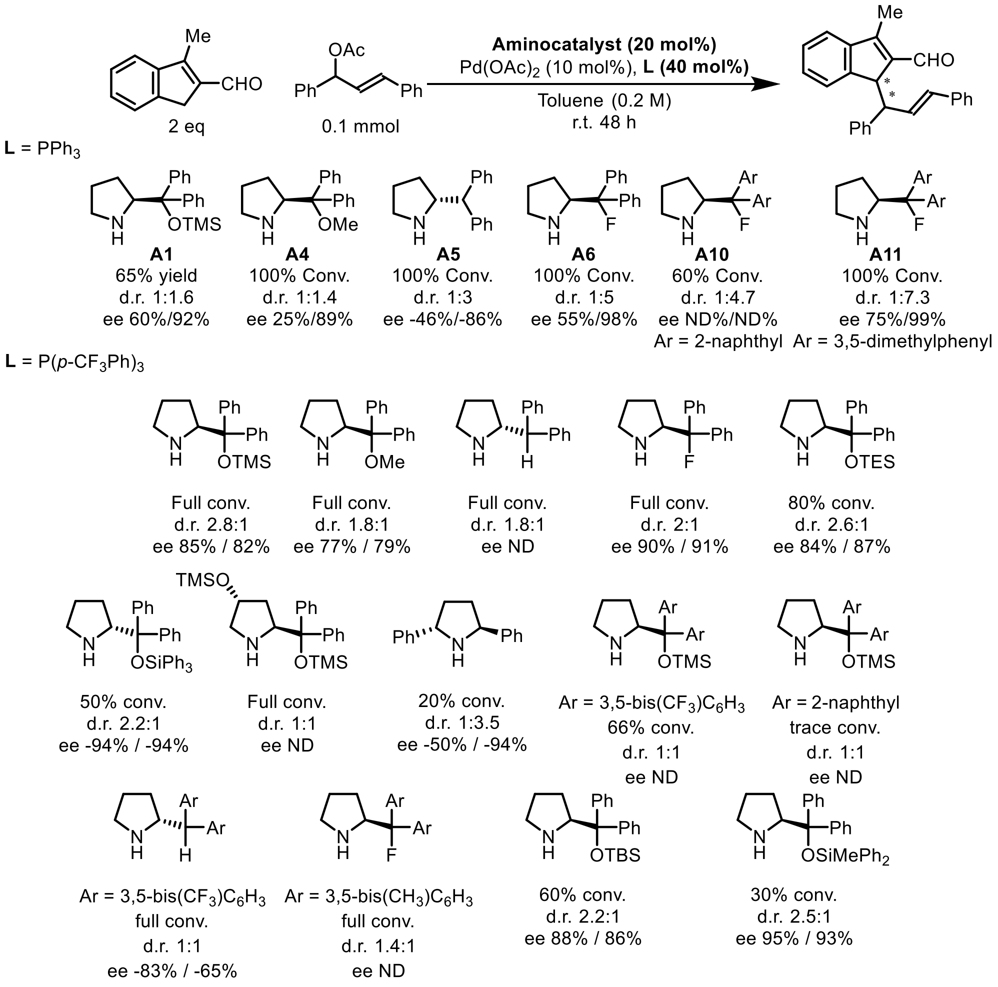

Experimental procedures

Synthesis and characterisation of substrates

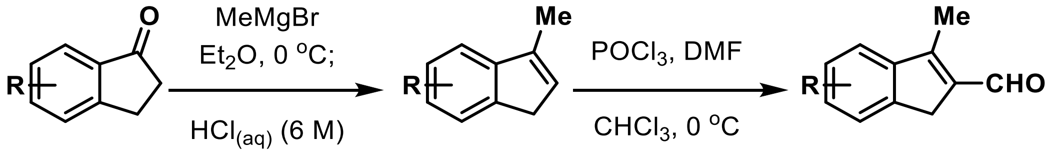

Representative general procedure I for the preparation of indene-2-carbaldehydes 1a,f:

Step 1. To the 1-indanone derivative (10 mmol, 1 equiv.) in Et_2O (100 mL) at 0 °C was added methylmagnesium bromide (3.0 *M* in Et_2O, 6.7 mL, 20 mmol, 2 equiv.) dropwise. The reaction was stirred for 15 min at this temperature following addition and the cooling bath removed. Upon warming to ambient temperature, TLC analysis indicated complete consumption of starting material. The reaction mixture was again cooled to 0 °C and HCl (6 M in water, 80 mL) was slowly added dropwise. The resulting biphasic mixture was vigorously stirred overnight prior to being transferred to a separatory funnel. The layers were separated and the aqueous layer was extracted with Et_2O (3 x 50 mL). The combined organic layers were then dried over $MgSO_4$, filtered and concentrated under reduced pressure. The resulting crude mixture was then purified by column chromatography (silica gel, pentane) to afford the corresponding indene (50 to 75% yields).

Step 2. To DMF (1.1 mmol, 1.1 equiv.) at 0 °C was added $POCl_3$ (1.1 mmol, 1.1 equiv.) dropwise. A colourless crystalline solid formed quickly, the ice-bath was removed following the addition and the reaction was allowed to reach rt and diluted with dry $CHCl_3$ (10 mL). The flask was then re-cooled to 0 °C and the indene (1 mmol, 1 equiv.) in 10 mL dry $CHCl_3$ was added dropwise. The now-coloured reaction mixture was then allowed to reach rt and stirred overnight. The resulting red solution was quenched with a saturated aqueous solution of NaOAc (30 mL), and the resulting biphasic mixture heated to 70 °C with vigorous stirring for 30 min. The mixture was transferred to a separatory funnel and the organic layer collected. The aqueous phase was then extracted with Et_2O (2 x 30 mL) and the combined organic layers washed with brine prior to being dried over Na_2SO_4 and concentrated under reduced pressure. The residue was then purified by column chromatography (see entries for specific conditions) to give the corresponding indene-2-carbaldehyde.

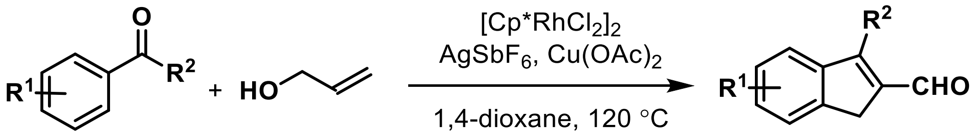

Representative general procedure II for the preparation of indene-2-carbaldehydes 1b-e,h-m:

(Synthesis modified from ref. 1) A 10-mL screw-cap vial was successively charged with ketone (1 mmol, if solid), $[RhCp^*Cl_2]_2$ (0.025 mmol), $Cu(OAc)_2$ (2.2 mmol) and $AgSbF_6$ (0.1 mmol). To this vial was added a solution of allyl alcohol (2 mmol) in dioxane (5 mL) (if the ketone is a liquid, it was pre-mixed with allyl alcohol). The vial was briefly flushed with argon, capped and placed in a hot plate pre-heated to 120 ºC with vigorous stirring for 24 h. Then it was cooled and filtered

through a short plug of silica (washed with EtOAc). After the removal of solvent and volatiles, the crude mixture was purified using column chromatography (see entries for specific conditions) to afford the desired aldehydes (conversions were typically 30-50% with isolated yields between 20-40%). Note: typically, 3-4 identical reactions (1 mmol each) were set up in parallel and purified together.

3-Methyl-1*H*-indene-2-carbaldehyde (**1a**)

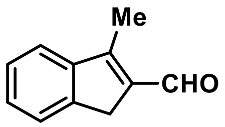

1-Indanone (5.29 g, 40.0 mmol) was subjected to general procedure I. The crude aldehyde was purified by column chromatography (silica gel, pentane/CH_2Cl_2, 1:4 to 0:5 v/v) to afford aldehyde **1a** (2.28 g, 14.4 mmol, 36% yield over two steps).

Physical State: orange solid.

Analytical data were in accordance with the literature.[1]

3-Ethyl-1*H*-indene-2-carbaldehyde (**1b**)

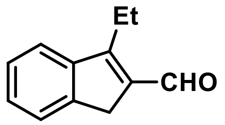

Propiophenone (130 mg, 1.0 mmol) was subjected to general procedure II, the crude product was purified by column chromatography (silica gel, pentane/Et_2O, 9:1 v/v) to afford 3-ethyl-1*H*-indene-2-carbaldehyde **1b** (53 mg, 0.31 mmol, 31% yield).

Physical State: yellow solid.

R*f* = 0.38 (pentane:Et_2O, 9:1 v/v; 2,4-DNP).

^{1}H-NMR [$CDCl_3$, 400 MHz] δ: 10.22 (s, 1H), 7.61 (m, 1H), 7.54 (m, 1H), 7.40 (m, 2H), 3.63 (s, 2H), 3.05 (q, *J* = 7.6 Hz, 2H), 1.36 (t, *J* = 7.6 Hz, 3H).

^{13}C-NMR [$CDCl_3$, 100 MHz] δ: 187.2, 162.3, 144.9, 143.7, 138.8, 129.1, 127.0, 124.9, 122.0, 35.8, 18.8, 15.1.

HRESI MS (*m*/*z*): $(M+H)^+$ calcd. for $C_{12}H_{13}O$, 173.0961; found 173.0969.

3-Cyclopropyl-1*H*-indene-2-carbaldehyde (**1c**)

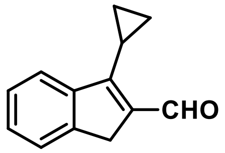

Cyclopropyl(phenyl)methanone (150 mg, 1.0 mmol) was subjected to general procedure II, the crude product was purified by column chromatography (silica gel, pentane/Et_2O, 17:3 v/v) to afford 3-cyclopropyl-1*H*-indene-2-carbaldehyde **1c** (88 mg, 0.48 mmol, 48% yield).

Physical State: yellow solid.

R*f* = 0.26 (pentane:Et_2O, 9:1 v/v; 2,4-DNP).

^{1}H-NMR [$CDCl_3$, 400 MHz] δ: 10.40 (s, 1H), 7.64 (m, 1H), 7.50 (m, 1H), 7.37 (m, 2H), 3.60 (m 2H), 2.20 (m, 1H), 1.19 (m, 2H), 1.07 (m, 2H).

[1] Z. Shi, M. Boultadakis-Arapinis, F. Glorius, *Chem. Commun.* **2013**, *49*, 6489-6491.

^{13}**C-NMR** [$CDCl_3$, 100 MHz] δ: 187.8, 160.5, 144.6, 144.0, 140.9, 128.8, 126.9, 124.8, 123.1, 35.8, 8.7, 7.1.

HRESI MS (*m*/*z*): $(M+H)^+$ calcd. for $C_{13}H_{13}O$, 185.0961; found 185.0965.

4-Methoxy-3-methyl-1*H*-indene-2-carbaldehyde (**1d**)

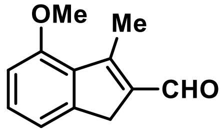

2'-Methoxyacetophenone (150 mg, 1.0 mmol) was subjected to general procedure II, the crude product was purified by column chromatography (silica gel, pentane/Et_2O, 17:3 v/v) to afford 4-methoxy-3-methyl-1*H*-indene-2-carbaldehyde **1d** (23 mg, 0.13 mmol, 13% yield).

Physical State: yellow solid.

$\mathbf{R}_f$ **=** 0.48 (pentane:Et_2O, 4:1 v/v; 2,4-DNP).

^{1}**H-NMR** [$CDCl_3$, 400 MHz] δ: 10.15 (s, 1H), 7.34 (t, J = 7.9 Hz, 1H), 7.10 (d, J = 7.4 Hz, 1H), 6.82 (d, J = 8.2 Hz, 1H), 3.90 (s, 3H), 3.59 (q, J = 2.1 Hz, 2H), 2.71 (t, J = 2.2 Hz, 3H).

^{13}**C-NMR** [$CDCl_3$, 100 MHz] δ: 187.0, 157.9, 157.5, 147.1, 138.2, 132.7, 130.7, 117.3, 109.0, 55.5, 36.1, 14.3.

HRESI MS (*m*/*z*): $(M+H)^+$ calcd. for $C_{12}H_{13}O_2$, 189.0910; found 189.0914.

4-Fluoro-3-methyl-1*H*-indene-2-carbaldehyde (**1e**)

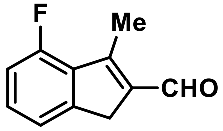

2'-Fluoroacetophenone (140 mg, 1.0 mmol) was subjected to general procedure II, the crude product was purified by column chromatography (silica gel, pentane/Et_2O, 9:1 v/v) to afford 4-fluoro-3-methyl-1*H*-indene-2-carbaldehyde **1e** (71 mg, 0.40 mmol, 40% yield).

Physical State: yellow solid.

$\mathbf{R}_f$ **=** 0.32 (pentane:Et_2O, 9:1 v/v; 2,4-DNP).

^{1}**H-NMR** [$CDCl_3$, 400 MHz] δ: 10.18 (s, 1H), 7.33 (m, J = 4.9 Hz, 1H), 7.27 (m, 1H), 7.00 (m, J = 10.7 Hz, 1H), 3.63 (q, J = 2.1 Hz, 2H), 2.68 (d, J = 2.0 Hz, 3H).

^{13}**C-NMR** [$CDCl_3$, 100 MHz] δ: 186.8, 159.3 (d, $^{1}J_{C\text{-}F}$ = 255 Hz), 154.1 (d, $^{3}J_{C\text{-}F}$ = 4 Hz), 147.0 (d, $^{3}J_{C\text{-}F}$ = 4 Hz), 139.4 (d, $^{4}J_{C\text{-}F}$ = 2 Hz), 131.7 (d, $^{2}J_{C\text{-}F}$ = 12 Hz), 130.6 (d, $^{3}J_{C\text{-}F}$ = 7 Hz), 120.6 (d, $^{3}J_{C\text{-}F}$ = 4 Hz), 114.1 (d, $^{2}J_{C\text{-}F}$ = 20 Hz), 36.3, 13.2 (d, $^{4}J_{C\text{-}F}$ = 4 Hz).

^{19}**F-NMR** [$CDCl_3$, 376 MHz] δ: -119.5.

HRESI MS (*m*/*z*): $(M+H)^+$ calcd. for $C_{11}H_{10}FO$, 177.0710; found 177.0709.

3,5-Dimethyl-1*H*-indene-2-carbaldehyde (**1f**)

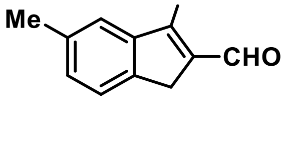

6-Methyl-1-indanone (1.83 g, 12.5 mmol) was subjected to general procedure I. The crude aldehyde was purified by column chromatography (silica gel, pentane/CH_2Cl_2, 1:4 to 0:5 v/v) to afford aldehyde **1f** (700 mg, 4.06 mmol, 32% yield over two steps).

Physical State: orange solid.

$\mathbf{R_f}$ **=** 0.40 (pentane:Et_2O, 1:1 v/v; 2,4-DNP).

^{1}H-NMR [$CDCl_3$, 400 MHz] δ: 10.23 (s, 1H), 7.43 (d, *J* = 7.6 Hz, 1H), 7.40 (s, 1H), 7.25 (dd, *J* = 7.6, 1.6 Hz, 1H), 3.60 (q, *J* = 2.0 Hz, 2H), 2.56 (t, *J* = 2.3 Hz, 3H), 2.46 (s, 3H).

^{13}C-NMR [$CDCl_3$, 100 MHz] δ: 187.3, 156.2, 145.1, 141.7, 139.9, 136.8, 130.3, 124.4, 122.3, 35.5, 21.6, 11.0.

HRESI MS (*m/z*): $(M+H)^+$ calcd. for $C_{12}H_{13}O$, 173.0961; found 173.0961.

3,6-Dimethyl-1*H*-indene-2-carbaldehyde (**1h**)

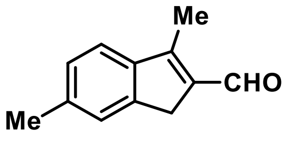

4'-Methylacetophenone (134 mg, 1.00 mmol) was subjected to general procedure II. The crude aldehyde was purified by column chromatography (silica gel, pentane/CH_2Cl_2, 1:4 to 0:5 v/v) to afford aldehyde **1h** (36.7 mg, 0.213 mmol, 21% yield).

Physical State: yellow solid.

Analytical data were in accordance with the literature.[1]

6-Methoxy-3-methyl-1*H*-indene-2-carbaldehyde (**1i**)

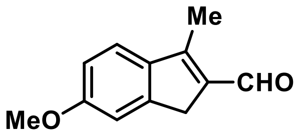

4'-Methoxyacetophenone (150 mg, 1.0 mmol) was subjected to general procedure II, the crude product was purified by column chromatography (silica gel, pentane/Et_2O, 19:1 to 4:1 v/v) to afford 6-methoxy-3-methyl-1*H*-indene-2-carbaldehyde **1i** (42 mg, 0.42 mmol, 22% yield).

Physical State: yellow solid.

$\mathbf{R_f}$ **=** 0.42 (pentane:Et_2O, 3:1 v/v; 2,4-DNP).

^{1}H-NMR [$CDCl_3$, 400 MHz] δ: 10.15 (s, 1H), 7.50 (d, *J* = 8.6 Hz, 1H), 7.09 (d, *J* = 2.3 Hz, 1H), 6.96 (dd, *J* = 8.6, 2.2 Hz, 1H), 3.88 (s, 3H), 3.62 (q, *J* = 1.8 Hz, 2H), 2.54 (t, *J* = 2.2 Hz, 3H).

^{13}C-NMR [$CDCl_3$, 100 MHz] δ: 186.6, 161.5, 156.6, 147.0, 138.0, 137.9, 122.8, 113.8, 110.0, 55.7, 35.8, 11.0.

HRESI MS (*m/z*): $(M+H)^+$ calcd. for $C_{12}H_{13}O_2$, 189.0910; found 189.0912.

6-Fluoro-3-methyl-1*H*-indene-2-carbaldehyde (**1j**)

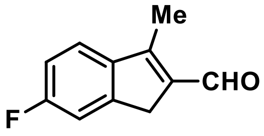

4'-Fluoroacetophenone (140 mg, 1.01 mmol) was subjected to general procedure II. The crude aldehyde was purified by column chromatography (silica gel, pentane/CH_2Cl_2, 1:4 to 0:5 v/v) to afford aldehyde **1j** (72.3 mg, 0.410 mmol, 41% yield).

Physical State: orange solid.

Analytical data were in accordance with the literature.[1]

6-Chloro-3-methyl-1*H*-indene-2-carbaldehyde (**1k**)

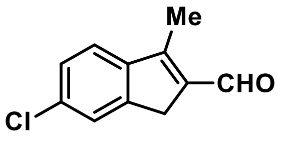

4’-Chloroacetophenone (155 mg, 1.00 mmol) was subjected to general procedure II. The crude aldehyde was purified by column chromatography (silica gel, pentane/CH_2Cl_2, 1:4 to 0:5 v/v) to afford aldehyde **1k** (57.1 mg, 0.296 mmol, 30% yield).

Physical State: orange solid.

Analytical data were in accordance with the literature.[1]

6-Bromo-3-methyl-1*H*-indene-2-carbaldehyde (**1l**)

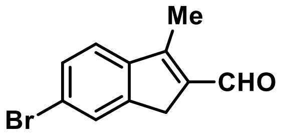

4’-Bromoacetophenone (199 mg, 1.00 mmol) was subjected to general procedure II. The crude aldehyde was purified by column chromatography (silica gel, pentane/CH_2Cl_2, 1:4 to 0:5 v/v) to afford aldehyde **1l** (75.0 g, 0.316 mmol, 32% yield).

Physical State: orange solid.

Analytical data were in accordance with the literature.[1]

3-Methyl-6-(methylthio)-1*H*-indene-2-carbaldehyde (**1m**)

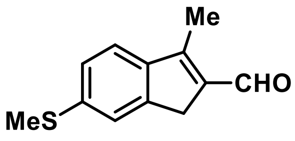

4’-(Methylthio)acetophenone (230 mg, 1.4 mmol) was subjected to general procedure II, the crude product was purified by column chromatography (silica gel, pentane/Et_2O, 19:1 to 7:3 v/v) to afford 3-methyl-6-(methylthio)-1*H*-indene-2-carbaldehyde **1m** (90 mg, 0.44 mmol, 31% yield).

Physical State: orange solid.

R*f* = 0.33 (pentane:Et_2O, 3:1 v/v; 2,4-DNP).

^{1}H-NMR [$CDCl_3$, 400 MHz] δ: 10.18 (s, 1H), 7.47 (d, J = 8.1 Hz, 1H), 7.39 (s, 1H), 7.26 (dd, J = 8.1, 1.5 Hz, 1H), 3.69-3.54 (q, J = 2.3 Hz, 2H), 2.60-2.48 (m, 6H).

^{13}C-NMR [$CDCl_3$, 100 MHz] δ: 187.0, 155.9, 145.3, 142.0, 140.9, 138.7, 125.0, 122.0, 121.9, 35.6, 15.8, 11.0.

HRESI MS (*m/z*): $(M+H)^+$ calcd. for $C_{12}H_{13}OS$, 205.0682; found 205.0683.

5-Methoxy-3-methyl-1*H*-indene-2-carbaldehyde (**1g**)

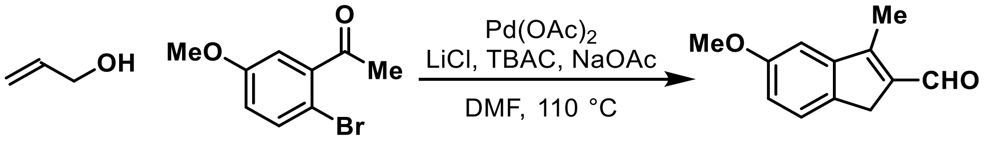

Synthesis of indene **1g**: To a 200-mL flask were weighed 2-bromoketone (2.3 g, 10 mmol), $Pd(OAc)_2$ (0.11 g, 0.50 mmol), LiCl (0.85 g, 20 mmol), TBAC (5.6 g, 20 mmol) and NaOAc (2.1 g, 25 mmol). To the mixture was added a solution of allyl alcohol (1.0 mL, 15 mmol) in DMF (100

mL). The resulting mixture was stirred at 110 ºC overnight, cooled down and separated in Et_2O (100 mL) and water (100 mL). The organic phase was collected, washed with water (2 x 50 mL) and brine (2 x 50 mL), dried over Na_2SO_4, and concentrated under reduced pressure. The crude residue was purified by column chromatography (silica gel, pentane/CH_2Cl_2, 1:1 to 0:10 v/v) to afford the indene-2-carbaldehyde **1g** (260 mg, 1.4 mmol, 14% yield).

Physical State: orange solid.

R_f = 0.31 (pentane:Et_2O, 3:1 v/v; 2,4-DNP).

^{1}H-NMR [$CDCl_3$, 400 MHz] δ: 10.23 (s, 1H), 7.43 (d, *J* = 8.2 Hz, 1H), 7.09 (d, *J* = 2.4 Hz, 1H), 7.00 (dd, *J* = 8.3, 2.3 Hz, 1H), 3.88 (s, 3H), 3.58 (q, *J* = 2.2 Hz, 2H), 2.55 (t, *J* = 2.1 Hz, 3H).

^{13}C-NMR [$CDCl_3$, 100 MHz] δ: 187.2, 159.4, 155.9, 146.2, 140.9, 136.7, 125.3, 116.0, 106.5, 55.8, 35.2, 11.0.

HRESI MS (*m/z*): $(M+H)^+$ calcd. for $C_{12}H_{13}O_2$, 189.0910; found 189.0913.

Representative general procedure III for the preparation of diarylallyl acetates 2a-j:

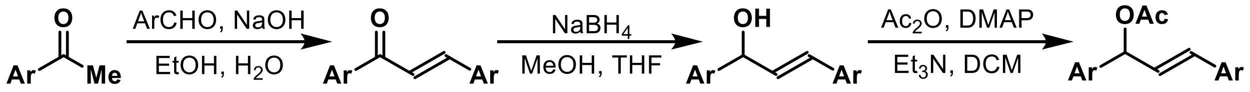

Step 1: To a solution of solution of aryl ketone (15 mmol, 1 equiv.) and aryl aldehyde (15 mmol, 1 equiv.) in EtOH (15 mL, 1 mL $mmol^{-1}$) was added NaOH (10 wt% in H_2O, 6 mL, 1 equiv.). The resulting mixture was stirred overnight (generally 16-18 h) and the precipitate formed was collected by vacuum filtration, washed with water (2 x 20 mL), MeOH (1 x 20 mL) and dried under vacuum. The solid chalcone was then used without further purification.

Step 2: A solution of the chalcone (6 mmol, 1 equiv.) in THF (8 mL, 0.75 mL $mmol^{-1}$) and MeOH (8 mL, 0.75 mL $mmol^{-1}$) was cooled to 0 °C and $NaBH_4$ (151.3, 4 equiv.) was added in one portion. The reaction mixture was stirred while slowly warmed up to rt (generally 1-2 h), over which time the reaction became colourless, indicating the consumption of the chalcone. After crude ^{1}H-NMR indicated the full consumption of the starting material, the mixture was diluted with Et_2O (50 mL) and the organic layer washed with water (2 x 30 mL) and brine (1 x 30 mL). The combined organic extracts were then dried over Na_2SO_4 and concentrated under reduced pressure to afford the crude allyl alcohol, which was used without further purification.

Step 3: A solution of the previous allyl alcohol (ca. 6 mmol), DMAP (0.6 mmol, 10 mol%) and triethylamine (60 mmol, 10 equiv.) in CH_2Cl_2 (12 mL, 0.5 mL $mmol^{-1}$) was cooled to 0 °C and acetic anhydride (12 mmol, 2 equiv.) was added dropwise. The reaction was continued stirring in the ice bath for ca. 1 h, when crude ^{1}H-NMR indicated the full conversion of the alcohol to the allyl acetate. The mixture was diluted with Et_2O (50 mL) and washed exhaustively with water (5 x 30 mL), a saturated solution of $NaHCO_3$ (2 x 30 mL) and brine (3 x 30 mL). The organic phase was then dried over Na_2SO_4 and concentrated under reduced pressure. The allyl acetates thus obtained were pure enough for use in subsequent reactions.

(*E*)-1,3-Diphenylallyl acetate (**2a**)

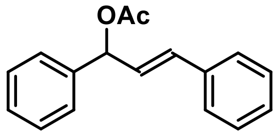

Starting from the commercially available *trans*-1,3-diphenyl-2-propen-1-ol (6.31 g, 30 mmol) was subjected to the general procedure III (Step 3) to afford (*E*)-1,3-diphenylallyl acetate (**2a**) (6.60 g, 26.2 mmol, 87% yield). Analytical data were in accordance with the literature.[2]

(*E*)-1,3-bis(4-Methylphenyl)allyl acetate (**2b**)

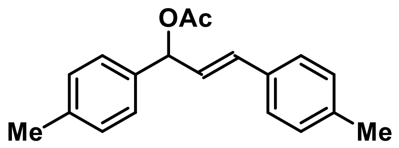

4'-Methylacetophenone (537 mg, 4.0 mmol) and 4-methylbenzaldehyde (0.47 mL, 4 mmol) were subjected to the general procedure III to afford (*E*)-1,3-bis(4-methylphenyl)allyl acetate (**2b**) (771 mg, 2.75 mmol, 69% yield, over 3 steps).

Physical State: colourless oil

[2] Y. Su, N. Jiao, *Org. Lett.* **2009**, *11*, 2980-2983.

Analytical data were in accordance with the literature.[3]

(E)-1,3-bis(4-Methoxyphenyl)allyl acetate (**2c**)

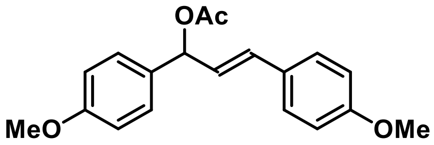

4'-Methoxyacetophenone (1.50 g, 10 mmol) was subjected to the general procedure III to afford (E)-1,3-bis(4-methoxyphenyl)allyl acetate (**2c**) (1.76 g, 5.631 mmol, 56% yield, over 3 steps).

Physical State: colourless oil.

R$_f$ = 0.69 (pentane: Et_2O, 3:1 v/v; $KMnO_4$).

^{1}H-NMR [$CDCl_3$, 400 MHz] δ: 7.35 (d, J = 8.6 Hz, 3H), 7.32 (d, J = 8.7 Hz, 3H), 6.91 (d, J = 8.8 Hz, 2H), 6.85 (d, J = 8.8 Hz, 2H), 6.56 (dd, J = 15.9, 1.2 Hz, 1H), 6.23 (dd, J = 15.8, 6.8 Hz, 1H), 3.82 (s, 6H), 3.81 (s, 6H), 2.12 (s, 3H).

^{13}C-NMR [$CDCl_3$, 100 MHz] δ: 170.3, 159.6, 159.5, 132.0, 131.7, 129.1, 128.6, 128.0, 125.5, 114.1 (2C), 76.2, 55.4, 55.4, 21.6.

HRESI MS (*m/z*): $(M\text{-}OAc)^+$ calcd. for $C_{17}H_{17}O_2$, 253.1223; found 253.1230.

(E)-1,3-bis(4-Fluorophenyl)allyl acetate (**2d**)

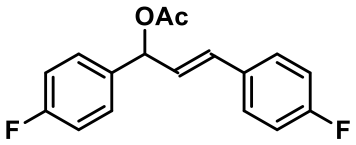

Starting from the commercially available 4,4'-difluorochalcone (488 mg, 2 mmol) was subjected to the general procedure III (Steps 2 and 3) to afford (E)-1,3-bis(4-fluorophenyl)allyl acetate (**2d**) (542 mg, 1.88 mmol, 94% yield, over 2 steps).

Physical State: colourless oil.

R$_f$ = 0.40 (pentane:CH_2Cl_2, 1:1 v/v; $KMnO_4$).

^{1}H-NMR [$CDCl_3$, 400 MHz] δ: 7.42-7.30 (m, 4H), 7.11-6.96 (m, 4H), 6.58 (d, J = 15.9 Hz), 6.40 (d, J = 6.8 Hz), 6.24 (dd, J = 15.9, 6.8 Hz), 2.13 (s, 3H).

^{13}C-NMR [$CDCl_3$, 100 MHz] δ: 170.0, 162.6 (d, J = 247.7 Hz), 162.5 (d, J = 246.9 Hz), 135.0 (d, J = 3.2 Hz), 132.1 (d, J = 3.3 Hz), 131.5, 128.8 (d, J = 8.4 Hz, 2C), 128.2 (d, J = 8.1 Hz, 2C), 127.0 (d, J = 2.0 Hz), 115.5 (d, J = 21.6 Hz, 4C), 73.3, 21.3.

^{19}F-NMR [$CDCl_3$, 376 MHz] δ: -113.4, -113.7.

HRESI MS (*m/z*): $(M\text{-}OAc)^+$ calcd. for $C_{15}H_{11}F_2$, 229.0823; found 229.0824.

(E)-1,3-bis(4-Chlorophenyl)allyl acetate (**2e**)

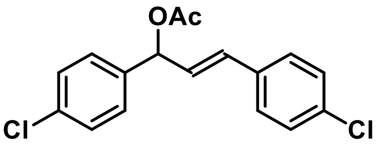

4'-Chloroacetophenone (1.54 g, 10.0 mmol) was subjected to the general procedure III to afford (E)-1,3-bis(4-chlorophenyl)allyl acetate (**2e**) (1.70 g, 5.29 mmol, 53% yield, over 3 steps).

Physical State: colourless oil.

[3] N. Kinoshita, T. Kawabata, K. Tsubaki, M. Bando, K. Fuji, *Tetrahedron*, **2006**, *62*, 1756-1763.

R_f = 0.27 (pentane: Et_2O, 3:1 v/v; $KMnO_4$).

^{1}H-NMR [$CDCl_3$, 400 MHz] δ: 7.35 (d, *J* = 3.0 Hz, 2H), 7.34 (d, *J* = 3.1 Hz, 2H), 7.29 (d, *J* = 3.1 Hz, 2H), 7.28 (d, *J* = 3.0 Hz, 2H), 6.56 (dd, *J* = 15.8, 1.2 Hz, 1H), 6.38 (dd, *J* = 6.6, 1.2 Hz, 1H), 6.27 (dd, *J* = 15.8, 6.7 Hz, 1H), 2.13 (s, 3H).

^{13}C-NMR [$CDCl_3$, 100 MHz] δ: 170.0, 137.6, 134.6, 134.2, 134.0, 131.8, 129.0, 128.9, 128.6, 128.0, 127.8, 75.4, 21.4.

HRESI MS (*m/z*): $(M\text{-}OAc)^+$ calcd. for $C_{15}H_{11}Cl_2$, 261.0232; found 261.0231.

(*E*)-1,3-bis(4-Bromophenyl)allyl acetate (**2f**)

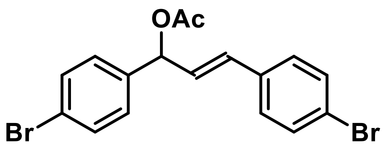

Prepared through the use of general procedure III. For step 1, 4'-bromoacetophenone (1.99 g, 10 mmol) and 4-bromobenzaldehyde (1850 mg, 10 mmol) afforded 4,4'-dibromochalcone (3.03 g, 8.27 mmol, 83% yield). For steps 2 and 3, 4,4'-dibromochalcone (1.00 g, 2,73 mmol) afforded (*E*)-1,3-bis(4-bromophenyl)allyl acetate (**2f**) (600 mg, 1.46 mmol, 53% over 2 steps).

Physical State: colourless oil.

Analytical data were in accordance with the literature.[4]

(*E*)-1,3-bis(4-(Trifluoromethyl)phenyl)allyl acetate (**2g**)

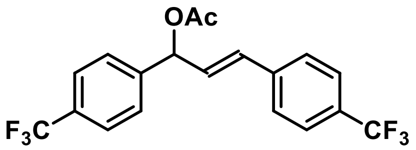

4'-(Trifluoromethyl)acetophenone (1.03 g, 2.91 mmol) was subjected to the general procedure III to afford (*E*)-1,3-bis(4-(trifluoromethyl)phenyl)allyl acetate (**2g**) (843 mg, 2.17 mmol, 75% yield, over 3 steps).

Physical State: yellow oil.

R_f = 0.60 (pentane:Et_2O, 4:1 v/v; $KMnO_4$).

^{1}H-NMR [$CDCl_3$, 400 MHz] δ: 7.65 (d, *J* = 8.2 Hz, 2H), 7.57 (d, *J* = 8.3 Hz, 2H), 7.53 (d, *J* = 8.2 Hz, 2H), 7.47 (d, *J* = 8.2 Hz, 2H), 6.68 (d, *J* = 15.8 Hz, 1H), 6.48 (d, *J* = 6.7 Hz, 1H), 6.40 (dd, *J* = 15.8, 6.6 Hz, 1H), 2.17 (s, 3H).

^{13}C-NMR [$CDCl_3$, 100 MHz] δ: 169.94, 142.84 (q, *J* = 1.0 Hz), 139.42 (q, *J* = 1.2 Hz), 131.89, 130.69 (q, *J* = 32.5 Hz), 130.25 (q, *J* = 32.5 Hz), 129.44, 127.45, 127.06, 125.89 (q, *J* = 3.8 Hz), 125.76 (q, *J* = 3.8 Hz), 124.17 (q, *J* = 271.9 Hz), 124.08 (q, *J* = 272.2 Hz), 75.24, 21.33.

^{19}F-NMR [$CDCl_3$, 376 MHz] δ: -62.62, -62.65.

HRESI MS (*m/z*): $(M\text{-}OAc)^+$ calcd. for $C_{17}H_{11}F_6$, 329.0759; found 329.0761.

[4] B. Lu, B. Feng, H. Ye, J.-R. Chen, W.-J. Xiao, *Org. Lett.* **2018**, *20*, 3473-3476.

(*E*)-1,3-bis(4-Nitrophenyl)allyl acetate (**2h**)

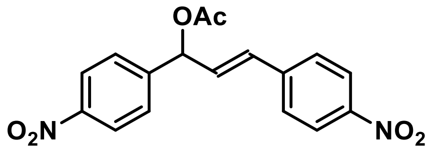

4’-Nitroacetophenone (1.24 g, 7.5 mmol) and 4-nitrobenzaldehyde (1.13 g, 7.5 mmol) were subjected to the general procedure III to afford (*E*)-1,3-bis(4-nitrophenyl)allyl acetate (**2h**) (420 mg, 1.23 mmol, 16% yield, over 3 steps).

Physical State: yellow solid.

Analytical data were in accordance with the literature.[3]

(*E*)-1,3-bis(3-Chlorophenyl)allyl acetate (**2i**)

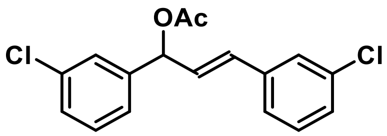

3’-Chloroacetophenone (1.55 g, 10.0 mmol) was subjected to the general procedure III to afford (*E*)-1,3-bis(3-chlorophenyl)allyl acetate (**2i**) (1.85 g, 5.77 mmol, 58% yield, over 3 steps).

Physical State: colourless oil.

R_f = 0.60 (pentane:CH_2Cl_2, 1:4 v/v).

^{1}H-NMR [$CDCl_3$, 400 MHz] δ: 7.38-7.28 (m, 8H), 6.57 (dd, *J* = 15.8, 1.0 Hz, 1H), 6.39 (dd, *J* = 6.7, 0.9 Hz, 1H), 6.31 (dd, *J* = 15.7, 6.7 Hz, 1H), 2.14 (s, 3H).

^{13}C-NMR [$CDCl_3$, 100 MHz] δ: 170.0, 141.1, 137.9, 134.7 (2C), 131.8, 130.1, 130.0, 128.6, 128.5, 128.3, 127.2, 126.7, 125.3, 125.2, 75.3, 21.4.

HRESI MS (*m/z*): $(M+Na)^+$ calcd. for $C_{17}H_{14}Cl_2NaO_2$, 343.0263; found 343.0265.

(*E*)-1,3-bis(3-Bromophenyl)allyl acetate (**2j**)

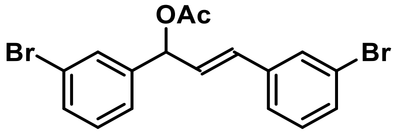

3’-Bromoacetophenone (1.59 g, 8.01 mmol) was subjected to the general procedure III to afford (*E*)-1,3-bis(3-bromophenyl)allyl acetate (**2j**) (2.30 g, 5.62 mmol, 56% yield, over 3 steps).

Physical State: colourless oil.

R_f = 0.57 (pentane:CH_2Cl_2, 1:4 v/v).

^{1}H-NMR [$CDCl_3$, 400 MHz] δ: 7.55 (dt, *J* = 3.4, 1.8 Hz, 2H), 7.47 (ddd, *J* = 7.8, 2.0, 1.2 Hz, 1H), 7.39 (ddd, *J* = 7.8, 1.9, 1.1 Hz, 1H), 7.34-7.24 (m, 3H), 7.21-7.17 (m, 1H), 6.57 (d, *J* = 15.7 Hz, 1H), 6.38 (d, *J* = 6.8 Hz, 1H), 6.30 (dd, *J* = 15.7, 6.7 Hz, 1H), 2.16 (s, 3H).

^{13}C-NMR [$CDCl_3$, 100 MHz] δ: 170.0, 141.3, 138.2, 131.6, 131.5, 131.2, 130.4, 130.3, 130.1, 129.6, 128.5, 125.8, 125.6, 122.9, 122.9, 75.2, 21.4.

HRESI MS (*m/z*): $(M-OAc)^+$ calcd. for $C_{15}H_{11}Br_2$, 348.9222; found 348.9235.

Procedures for diastereodivergent allylation and characterization of products

Preparation of allylation racemates

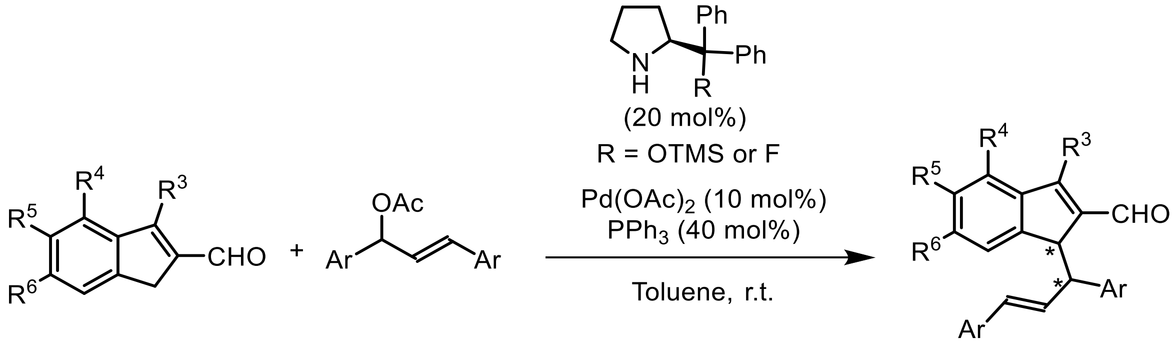

Scheme 3. Preparation of racemic products

The racemic allylation products were prepared using either racemic α,α-diphenylprolinol trimethylsilyl ether catalyst (prepared from weighing equimolar amounts of (*R*)- and (*S*)-catalyst) or 2-(fluorodiphenylmethyl)pyrrolidine (prepared from weighing equimolar amounts of (*R*)- and (*S*)-catalyst) with triphenylphosphine as the ligand on palladium.

Procedure: To an oven-dried vial were weighed indene-2-carbaldehyde (0.2 mmol, 2 equiv.), diarylallyl acetate (0.1 mmol, 1 equiv.), either (*S*)-α,α-diphenylprolinol trimethylsilyl ether (0.01 mmol, 10 mol%) and (*R*)-α,α-diphenylprolinol trimethylsilyl ether (0.01 mmol, 10 mol%), or (*S*)-2-(fluorodiphenylmethyl)pyrrolidine (0.01 mmol, 10 mol%) and (*R*)-2-(fluorodiphenylmethyl)pyrrolidine (0.01 mmol, 10 mol%), and triphenylphosphine (0.04 mmol, 40 mol%). A stock solution of $Pd(OAc)_2$ (0.5 mL, 0.02 *M* in dry/degassed PhMe) was then added and the reaction vial sparged with argon. The reaction was left to stir for 24 h at rt (~22 °C) prior to purification by column chromatography.

General procedures for the diastereodivergent allylation of indene-2-carbaldehydes

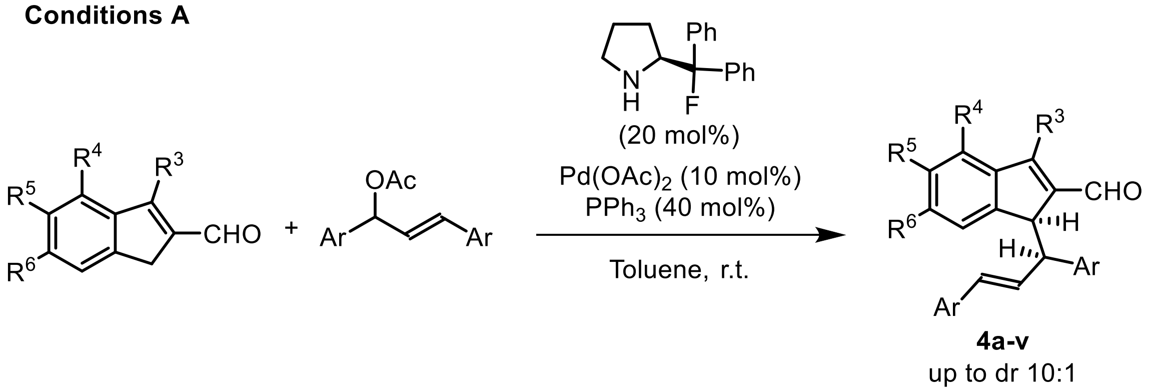

Conditions A for the allylation of indene-2-carbaldehydes: To an oven-dried vial were weighed indene-2-carbaldehyde (0.2 mmol, 2 equiv.), diarylallyl acetate (0.1 mmol, 1 equiv.), (*S*)-2-(fluorodiphenylmethyl)pyrrolidine (0.02 mmol, 20 mol%), and triphenylphosphine (0.04 mmol, 40 mol%). A stock solution of $Pd(OAc)_2$ (0.5 mL, 0.02 M in dry/degassed PhMe) was then added

and the reaction vial sparged with argon. The reaction was left to stir for 48 h at rt (~22 °C) at which point starting material was consumed (as monitored by ^{1}H-NMR) prior to purification by column chromatography.

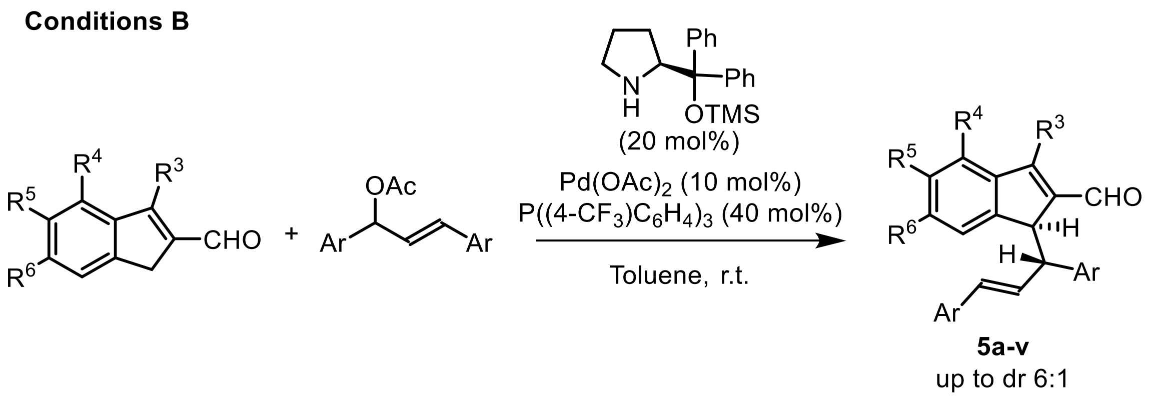

Conditions B for the allylation of indene-2-carbaldehydes: To an oven-dried vial were weighed indene-2-carbaldehyde (0.2 mmol, 2 equiv.), diarylallyl acetate (0.1 mmol, 1 equiv.), (*S*)-α,α-diphenylprolinol trimethylsilyl ether (0.02 mmol, 20 mol%), and triphenylphosphine (0.04 mmol, 40 mol%). A stock solution of $Pd(OAc)_2$ (0.5 mL, 0.02 M in dry/degassed PhMe) was then added and the reaction vial sparged with argon. The reaction was left to stir for 48 h at rt (~22 °C) at which point starting material was consumed (as monitored by ^{1}H-NMR) prior to purification by column chromatography.

(*S*)-1-((*S*,*E*)-1,3-Diphenylallyl)-3-methyl-1*H*-indene-2-carbaldehyde (**4a**)

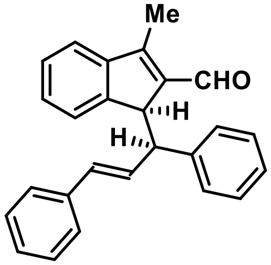

3-Methyl-1*H*-indene-2-carbaldehyde **1a** (31.6 mg, 0.2 mmol) and allyl acetate **2a** (25.2 mg, 0.1 mmol) were subjected to conditions A, the crude product was purified by column chromatography (silica gel, pentane/CH_2Cl_2, 1:1 to 1:4 v/v) to afford **4a** (29.1 mg, 0.083 mmol, 83% yield, d.r. (**4**:**5**) = 10:1, ee = 96%/57%).

Physical State: yellow oil.

R_f = 0.26 (pentane:CH_2Cl_2, 4:6 v/v; 2,4-DNP).

[α]D= +14.3° ($CHCl_3$, *c* 0.33).

^{1}H-NMR [$CDCl_3$, 400 MHz] δ: 10.13 (s, 1 H), 7.72-7.66 (m, 1H), 7.60 – 6.99 (m, 11 H), 6.91 (dd, *J* = 15.9, 7.3 Hz, 1H), 6.68 (m, 2H), 6.57 (d, *J* = 15.2 Hz, 1H) 4.69 (t, *J* = 5.8 Hz, 1H), 4.38 (dd, *J* = 5.2, 1.9 Hz, 1H), 2.31 (d, *J* = 1.9 Hz, 3H).

^{13}C-NMR [$CDCl_3$, 100 MHz] δ: 187.5, 157.1, 146.9, 144.7, 141.0, 139.5, 137.5, 133.1, 131.0, 129.1, 128.7 (2C), 128.2 (2C), 127.7 (2C), 127.6, 127.4, 126.6, 126.4 (2C), 125.4, 122.0, 53.6, 49.8, 10.6.

For data corresponding to the minor diastereomer, see: **5a**

HRESI MS (*m*/*z*): $(M+Na)^+$ calcd. for $C_{26}H_{22}NaO$, 373.1568; found 373.1565.

UPCC: Daicel Chiralpak IC, CO_2/*i*PrOH = 85/15, 3 mL/min, 40 °C, 254 nm, for major diastereomer t_R (major) = 3.80 min; t_R (minor) = 4.83 min, e.r. = 98:2, for minor diastereomer t_R (major) = 3.31 min; t_R (minor) = 4.12 min, e.r. = 78:22.

(*S*)-1-((*R*,*E*)-1,3-Diphenylallyl)-3-methyl-1*H*-indene-2-carbaldehyde (**5a**)

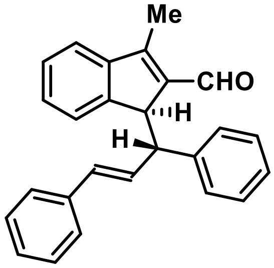

3-Methyl-1*H*-indene-2-carbaldehyde **1a** (31.6 mg, 0.2 mmol) and allyl acetate **2a** (25.2 mg, 0.1 mmol) were subjected to conditions B, the crude product was purified by column chromatography (silica gel, pentane/CH_2Cl_2, 1:1 to 1:4 v/v) to afford **5a** (26.7 mg, 0.076 mmol, 76% yield, d.r. (**4**:**5**) = 1:2.7, ee = 83%/82%).

Physical State: yellow oil.

R*f* = 0.3 (pentane:CH_2Cl_2, 1:1 v/v; 2,4-DNP).

^{1}H-NMR [$CDCl_3$, 400 MHz] δ: 10.23 (s, 1H), 7.54 (d, *J* = 7.6 Hz, 2H) 7.50-6.95 (m, 11H), 6.62-6.54 (m, 1H), 6.35 (d, *J* = 15.7 Hz, 1H), 5.64 (dd, *J* = 15.7, 10.0 Hz, 1H), 4.83 (dd, *J* = 10.0, 3.9 Hz, 1H), 4.46-4.30 (m, 1H), 2.49 (d, *J* = 1.8 Hz, 3H).

^{13}C-NMR [$CDCl_3$, 100 MHz] δ: 187.2, 156.9, 145.9, 144.5, 143.3, 141.4, 137.3, 132.9, 128.8, 128.4 (2C), 128.3 (2C), 128.2 (2C), 127.4, 127.1, 126.5, 126.2 (2C), 126.0, 125.3, 121.7, 53.4, 47.4, 10.7.

For data corresponding to the minor diastereomer, see: **4a**

HRESI MS (*m/z*): $(M+Na)^+$ calcd. for $C_{26}H_{22}NaO$, 373.1568; found 373.1572.

UPCC: Daicel Chiralpak IC, CO_2/*i*PrOH = 85/15, 3 mL/min, 40 °C, 254 nm, for major diastereomer t_R (major) = 3.29 min; t_R (minor) = 4.08 min, e.r. = 92:8, for minor diastereomer t_R (major) = 3.76 min; t_R (minor) = 4.81 min, e.r. = 91:9.

(*S*)-1-((*S*,*E*)-1,3-Diphenylallyl)-3-ethyl-1*H*-indene-2-carbaldehyde (**4b**)

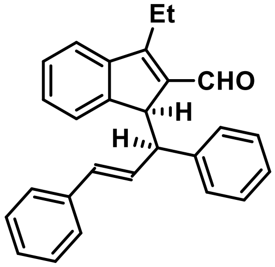

3-Ethyl-1*H*-indene-2-carbaldehyde **1b** (34.4 mg, 0.2 mmol) and allyl acetate **2a** (25.2 mg, 0.1 mmol) were subjected to conditions A, the crude product was purified by column chromatography (silica gel, pentane/CH_2Cl_2, 1:1 to 1:4 v/v) to afford **4b** (28.7 mg, 0.079 mmol, 79% yield, d.r. (**4**:**5**) = 4.2:1, ee = 83%/68%).

Physical State: yellow oil.

R*f* = 0.31 (pentane:CH_2Cl_2, 4:6 v/v; 2,4-DNP).

^{1}H-NMR [$CDCl_3$, 400 MHz] δ: 10.07 (s, 1H), 7.79-7.65 (m, 1H), 7.59-6.82 (m, 11H), 6.65-6.49 (m, 3H), 7.59-6.82 (m, 1H), 4.73-4.68 (m, 1H), 4.32 (d, *J* = 5.2 Hz, 1H), 2.88 – 2.78 (m, 1H), 2.71-2.59 (m, 1H), 0.84 (t, *J* = 7.6 Hz, 3H).

^{13}C-NMR [$CDCl_3$, 100 MHz] δ: 187.3, 163.5, 147.2, 143.9, 140.2, 139.2, 137.6, 132.9, 131.1, 128.9, 128.7 (2C), 128.1 (2C), 127.6 (2C), 127.5, 127.4, 126.7, 126.4 (2C), 125.6, 122.1, 53.5, 49.7, 18.3, 14.6.

For data corresponding to the minor diastereomer, see: **5b**

HRESI MS (*m/z*): $(M+Na)^+$ calcd. for $C_{27}H_{24}NaO$, 387.1725; found 387.1723.

UPCC: Daicel Chiralpak ID, CO_2/*i*PrOH = 99/1 to 60/40 over 4 min, 3 mL/min, 40 °C, 254 nm, for major diastereomer t_R (major) = 4.82 min; t_R (minor) = 5.34 min, e.r. = 92:8, for minor diastereomer t_R (major) = 4.54 min; t_R (minor) = 5.15 min, e.r. = 84:16.

(*S*)-1-((*R*,*E*)-1,3-Diphenylallyl)-3-ethyl-1*H*-indene-2-carbaldehyde (**5b**)

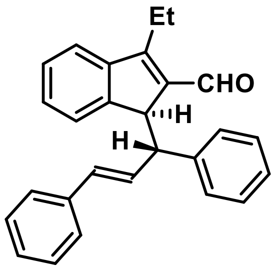

3-Ethyl-1*H*-indene-2-carbaldehyde **1b** (34.4 mg, 0.2 mmol) and allyl acetate **2a** (25.2 mg, 0.1 mmol) were subjected to conditions B, the crude product was purified by column chromatography (silica gel, pentane/CH_2Cl_2, 1:1 to 1:4 v/v) to afford **5b** (25.3 mg, 0.069 mmol, 69% yield, d.r. (**4**:**5**) = 1:2.2, ee = 94%/87%).

Physical State: yellow oil.

$\mathbf{R}_f$ = 0.31 (pentane:CH_2Cl_2, 4:6 v/v; 2,4-DNP).

^{1}H-NMR [$CDCl_3$, 400 MHz] δ: 10.22 (s, 1H), 7.65-6.81 (m, 13H), 6.67-6.49 (m, 1H), 6.35 (d, *J* = 15.7 Hz, 1H), 5.65 (dd, *J* = 15.7, 10.1 Hz, 1H), 4.83 (dd, *J* = 10.0, 4.0 Hz, 1H), 4.37 (d, *J* = 3.8 Hz, 1H), 3.03 (dq, *J* = 15.3, 7.6 Hz, 1H), 2.96-2.86 (m, 1H), 1.21 (t, *J* = 7.6 Hz, 3H).

^{13}C-NMR [$CDCl_3$, 100 MHz] δ: 187.1, 163.4, 146.5, 143.6, 143.3, 140.8, 137.3, 133.0, 128.7, 128.5 (4C), 128.4 (2C), 127.5, 127.5, 127.3, 126.6, 126.3 (2C), 126.0, 125.6, 122.0, 53.4, 47.7, 18.5, 15.4.

For data corresponding to the minor diastereomer, see: **4b**

HRESI MS (*m/z*): $(M+Na)^+$ calcd. for $C_{27}H_{24}NaO$, 387.1725; found 387.1723.

UPCC: Daicel Chiralpak ID, CO_2/*i*PrOH = 99/1 to 60/40 over 4 min, 3 mL/min, 40 °C, 254 nm, for major diastereomer t_R (major) = 4.55 min; t_R (minor) = 5.17 min, e.r. = 93:7, for minor diastereomer t_R (major) = 4.84 min; t_R (minor) = 5.37 min, e.r. = 97:3.

(*S*)-1-((*S*,*E*)-1,3-Diphenylallyl)-3-cyclopropyl-1*H*-indene-2-carbaldehyde (**4c**)

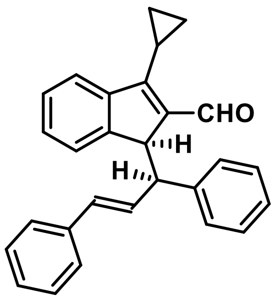

3-Cyclopropyl-1*H*-indene-2-carbaldehyde **1c** (36.8 mg, 0.2 mmol) and allyl acetate **2a** (25.2 mg, 0.1 mmol) were subjected to conditions A, the crude product was purified by column chromatography (silica gel, pentane/CH_2Cl_2, 1:1 to 1:4 v/v) to afford **4c** (25.3 mg, 0.067 mmol, 67% yield, d.r. (**4**:**5**) = 2.4:1, ee = 90%/-40%).

Physical State: yellow oil.

$\mathbf{R}_f$ = 0.31 (pentane:CH_2Cl_2, 4:6 v/v; 2,4-DNP).

^{1}H-NMR [$CDCl_3$, 400 MHz] δ: 10.28 (s, 1H), 7.70-7.66 (m, 1H), 7.54-7.50 (m, 1H), 7.44 (d, *J* = 7.3 Hz, 2H), 7.41-7.14 (m, 5H), 7.04 (t, *J* = 7.3 Hz, 1H), 6.94 (t, *J* = 7.5 Hz, 2H), 6.85 (dd, *J* = 15.9, 7.4 Hz, 1H), 6.58 (d, *J* = 15.7 Hz, 1H), 6.53-6.49 (m, 2H), 4.64 (t, *J* = 5.7 Hz, 1H), 4.31 (dd, *J* = 5.2, 2.1 Hz, 1H), 1.82-1.73 (m, 1H), 1.00 – 0.85 (m, 2H), 0.49 (td, *J* = 9.7, 5.5 Hz, 1H), 0.20 (td, *J* = 9.6, 5.7 Hz, 1H).

^{13}C-NMR [$CDCl_3$, 100 MHz] δ: 188.5, 161.5, 146.7, 144.8, 142.8, 139.2, 137.6, 132.8, 131.2, 128.7 (2C), 128.6, 128.1 (2C), 127.6, 127.4 (2C), 126.7, 126.4 (2C), 125.4, 123.2, 53.4, 50.0, 7.5, 6.9, 5.7.

For data corresponding to the minor diastereomer, see: **5c**

HRESI MS (*m/z*): $(M+Na)^+$ calcd. for $C_{28}H_{24}NaO$, 399.1719; found 399.1734.

UPCC: Daicel Chiralpak IB, CO_2/*i*PrOH = 99/1 to 60/40 over 4 min, 3 mL/min, 40 °C, 254 nm, for major diastereomer t_R (major) = 4.79 min; t_R (minor) = 5.24 min, e.r. = 95:5, for minor diastereomer t_R (major) = 5.13 min; t_R (minor) = 4.59 min, e.r. = 70:30.

(*S*)-1-((*R*,*E*)-1,3-Diphenylallyl)-3-cyclopropyl-1*H*-indene-2-carbaldehyde (**5c**)

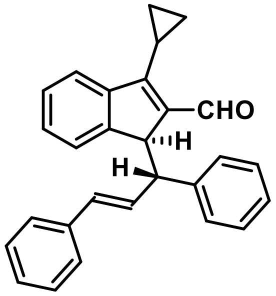

3-Cyclopropyl-1*H*-indene-2-carbaldehyde **1c** (36.8 mg, 0.2 mmol) and allyl acetate **2a** (25.2 mg, 0.1 mmol) were subjected to conditions B, the crude product was purified by column chromatography (silica gel, pentane/CH_2Cl_2, 1:1 to 1:4 v/v) to afford **5c** (19.7 mg, 0.052 mmol, 52% yield, d.r. (**4**:**5**) = 1:1.3, ee = 88%/33%).

Physical State: yellow oil.

R*f* = 0.31 (pentane:CH_2Cl_2, 4:6 v/v; 2,4-DNP).

^{1}H-NMR [$CDCl_3$, 400 MHz] δ: 10.43 (s, 1H), 7.64 (d, *J* = 7.7 Hz, 1H), 7.42-7.12 (m, 10H), 7.08 (d, *J* = 7.0 Hz, 2H), 6.56-6.52 (m, 1H), 6.32 (d, *J* = 15.7 Hz, 1H), 5.62 (dd, *J* = 15.7, 10.1 Hz, 1H), 4.77 (dd, *J* = 10.1, 3.9 Hz, 1H), 4.34 (dd, *J* = 3.4, 1.9 Hz, 1H), 2.09-2.00 (m, 1H), 1.15-1.03 (m, 2H), 0.90-0.84 (m, 1H), 0.80-0.72 (m, 1H).

^{13}C-NMR [$CDCl_3$, 100 MHz] δ: 188.2, 161.6, 146.1, 144.2, 143.2, 143.2, 137.4, 132.9, 128.5 (3C), 128.4 (2C), 128.4 (2C), 127.5, 127.4, 127.3, 126.6, 126.2 (2C), 126.1, 125.4, 123.1, 53.3, 47.9, 8.1, 7.1, 6.7.

For data corresponding to the minor diastereomer, see: **4c**

HRESI MS (*m/z*): $(M+Na)^+$ calcd. for $C_{28}H_{24}NaO$, 399.1719; found 399.1734.

UPCC: Daicel Chiralpak IB, CO_2/*i*PrOH = 99/1 to 60/40 over 4 min, 3 mL/min, 40 °C, 254 nm, for major diastereomer t_R (major) = 4.57 min; t_R (minor) = 5.15 min, e.r. = 67:33, for minor diastereomer t_R (major) = 4.76 min; t_R (minor) = 5.26 min, e.r. = 94:6.

(*S*)-1-((*S*,*E*)-1,3-Diphenylallyl)-4-methoxy-3-methyl-1*H*-indene-2-carbaldehyde (**4d**)

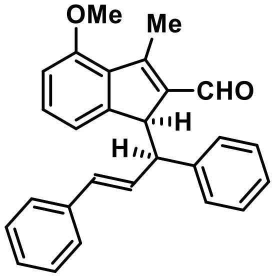

4-Methoxy-3-methyl-1*H*-indene-2-carbaldehyde **1c** (37.6 mg, 0.2 mmol) and allyl acetate **2a** (25.2 mg, 0.1 mmol) were subjected to conditions A, the crude product was purified by column chromatography (silica gel, pentane/CH_2Cl_2, 1:1 to 1:4 v/v) to afford **4d** (29.0 mg, 0.076 mmol, 76% yield, d.r. = 2.9:1, ee = 92%/0%).

Physical State: orange oil.

R*f* = 0.53 (pentane:Et_2O, 3:1 v/v; 2,4-DNP).

^{1}H-NMR [$CDCl_3$, 400 MHz] δ: 10.04 (s, 1H), 7.50-6.98 (m, 11H), 6.84 (m, 2H), 6.69 (m, 1H), 6.53 (d, *J* = 16.0 Hz, 1H), 4.67 (m, 1H), 4.30 (m, 1H), 3.84 (s, 3H), 2.45 (d, *J* = 2.0 Hz, 3H).

^{13}C-NMR [$CDCl_3$, 100 MHz] δ: 187.2, 158.9, 157.4, 149.5, 139.6, 137.7, 133.3, 132.4, 130.9, 130.5, 128.7 (2C), 128.3 (2C), 127.7 (2C), 127.3, 126.6, 126.4, 126.4 (2C), 118.0, 109.7, 55.4, 53.7, 49.8, 13.9.

For data corresponding to the minor diastereomer, see: **5d**

HRESI MS (*m/z*): $(M+Na)^+$ calcd. for $C_{27}H_{24}NaO_2$, 403.1669; found 403.1681.

UPCC: Daicel Chiralpak IC, CO_2/*i*PrOH = 85/15, 3 mL/min, 40 °C, 254 nm, for major diastereomer t_R (major) = 5.75 min; t_R (minor) = 7.70 min, e.r. = 96:4, for minor diastereomer t_R (major) = 6.40 min; t_R (minor) = 4.98 min, e.r. = 50:50.

(*S*)-1-((*R*,*E*)-1,3-Diphenylallyl)-4-methoxy-3-methyl-1*H*-indene-2-carbaldehyde (**5d**)

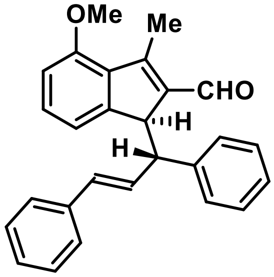

4-Methoxy-3-methyl-1*H*-indene-2-carbaldehyde **1c** (37.6 mg, 0.2 mmol) and allyl acetate **2a** (25.2 mg, 0.1 mmol) were subjected to conditions B, the crude product was purified by column chromatography (silica gel, pentane/CH_2Cl_2, 1:1 to 1:4 v/v) to afford **5d** (27.1 mg, 0.071 mmol, 71% yield, d.r. (**4**:**5**) = 1:2, ee = 69%/75%).

Physical State: orange oil.

R*f* = 0.53 (pentane:Et_2O, 3:1 v/v; 2,4-DNP).

^{1}H-NMR [$CDCl_3$, 400 MHz] δ: 10.17 (s, 1H), 7.65-6.75 (m, 10H), 6.35 (d, *J* = 15.8 Hz, 2H), 6.15 (d, *J* = 7.6 Hz, 2H), 5.70 (dd, *J* = 15.7, 10.2 Hz, 1H), 4.83 (m, 1H), 4.30 (m, 1H), 3.89 (s, 3H), 2.65 (d, *J* = 2.0 Hz, 3H).

^{13}C-NMR [$CDCl_3$, 100 MHz] δ: 187.0, 158.8, 157.4, 148.7, 143.6, 140.2, 137.6, 133.1, 132.3, 130.3, 128.5 (2C), 128.5 (2C), 128.4 (2C), 127.3, 126.5, 126.5 (2C), 126.4, 118.0, 109.7, 55.4, 53.6, 47.7, 14.2.

For data corresponding to the minor diastereomer, see: **4d**

HRESI MS (*m/z*): $(M+Na)^+$ calcd. for $C_{27}H_{24}NaO_2$, 403.1669; found 403.1667.

UPCC: Daicel Chiralpak IC, CO_2/*i*PrOH = 85/15, 3 mL/min, 40 °C, 254 nm, for major diastereomer t_R (major) = 5.07 min; t_R (minor) = 6.52 min, e.r. = 88:12, for minor diastereomer t_R (major) = 5.86 min; t_R (minor) = 7.85 min, e.r. = 85:15.

(*S*)-1-((*S*,*E*)-1,3-Diphenylallyl)-4-fluoro-3-methyl-1*H*-indene-2-carbaldehyde (**4e**)

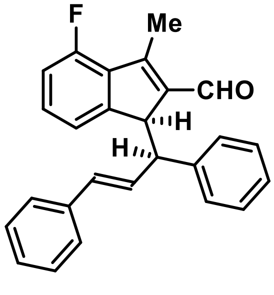

4-Fluoro-3-methyl-1*H*-indene-2-carbaldehyde **1e** (35.2 mg, 0.2 mmol) and allyl acetate **2a** (25.2 mg, 0.1 mmol) were subjected to conditions A, the crude product was purified by column chromatography (silica gel, pentane/CH_2Cl_2, 1:1 to 1:4 v/v) to afford **4e** (25.0 mg, 0.068 mmol, 68% yield, d.r. (**4**:**5**) = 2.4:1, ee = 89%/-36%).

Physical State: yellow oil.

R*f* = 0.72 (pentane:Et_2O, 3:1 v/v; 2,4-DNP).

^{1}H-NMR [$CDCl_3$, 400 MHz] δ: 10.07 (s, 1H), 7.46-6.97 (m, 11H), 6.83 (dd, *J* = 15.9, 7.0 Hz, 1H), 6.65 (d, *J* = 7.4 Hz, 2H), 6.54 (d, *J* = 15.7 Hz, 1H), 4.65 (t, *J* = 6.0 Hz, 1H), 4.37 (m, 1H), 2.42 (m, 3H).

^{13}C-NMR [$CDCl_3$, 100 MHz] δ: 187.2, 159.3 (d, *J* = 255 Hz), 155.2 (d, *J* = 4.1 Hz), 149.6 (d, *J* = 4.0 Hz), 141.0 (d, *J* = 1.5 Hz), 139.1, 137.4, 132.7, 131.6 (d, *J* = 12.3 Hz), 131.3, 130.5 (d, *J* = 7.5 Hz), 128.8 (2C), 128.1 (2C), 127.8 (2C), 127.5, 126.9, 126.4 (2C), 121.4 (d, *J* = 3.4 Hz), 114.8 (d, *J* = 20.4 Hz), 54.3, 49.7, 13.0 (d, *J* = 5.0 Hz).

^{19}F-NMR [$CDCl_3$, 376 MHz] δ: -118.9.

For data corresponding to the minor diastereomer, see: **5e**

HRESI MS (*m*/*z*): $(M+Na)^+$ calcd. for $C_{26}H_{21}FNaO$, 391.1469; found 391.1480.

UPCC: Daicel Chiralpak IC, CO_2/*i*PrOH = 85/15, 3 mL/min, 40 °C, 254 nm, for major diastereomer t_R (major) = 2.61 min; t_R (minor) = 3.35 min, e.r. = 94:6, for minor diastereomer t_R (major) = 2.87 min; t_R (minor) = 2.36 min, e.r. = 68:32.

(*S*)-1-((*R*,*E*)-1,3-Diphenylallyl)-4-fluoro-3-methyl-1*H*-indene-2-carbaldehyde (**5e**)

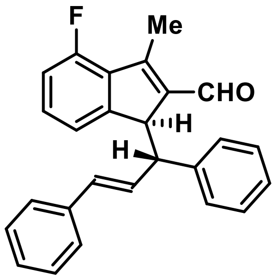

4-Fluoro-3-methyl-1*H*-indene-2-carbaldehyde **1e** (35.2 mg, 0.2 mmol) and allyl acetate **2a** (25.2 mg, 0.1 mmol) were subjected to conditions B, the crude product was purified by column chromatography (silica gel, pentane/CH_2Cl_2, 1:1 to 1:4 v/v) to afford **5e** (25.4 mg, 0.069 mmol, 69% yield, d.r. (**4**:**5**) = 1:2, ee = 81%/63%).

Physical State: yellow oil.

R_f = 0.72 (pentane:Et_2O, 3:1 v/v; 2,4-DNP).

^{1}H-NMR [$CDCl_3$, 400 MHz] δ: 10.20 (s, 1H), 7.50-6.97 (m, 12H), 6.40-6.29 (m, 2H), 5.67 (dd, *J* = 15.7, 10.1 Hz, 1H), 4.83 (dd, *J* = 10.1, 3.9 Hz, 1H), 4.38 (m, 1H), 2.64 (m, 3H).

^{13}C-NMR [$CDCl_3$, 100 MHz] δ: 187.0, 159.3 (d, *J* = 255 Hz), 155.1 (d, *J* = 4.1 Hz), 148.7 (d, *J* = 4.1 Hz), 143.1, 141.5 (d, *J* = 1.5 Hz), 137.3, 133.4, 131.5 (d, *J* = 11.9 Hz), 130.4 (d, *J* = 7.4 Hz),128.6 (2C), 128.5 (2C), 128.3 (2C), 127.5, 126.8, 126.4 (2C), 125.6, 121.4 (d, *J* = 3.4 Hz), 114.8 (d, *J* = 20.4 Hz), 54.2, 47.6, 13.2 (d, *J* = 5.1 Hz).

^{19}F-NMR [$CDCl_3$, 376 MHz] δ: -119.0.

For data corresponding to the minor diastereomer, see: **4e**

HRESI MS (*m*/*z*): $(M+Na)^+$ calcd. for $C_{26}H_{21}FNaO$, 391.1469; found 391.1471.

UPCC: Daicel Chiralpak IC, CO_2/*i*PrOH = 85/15, 3 mL/min, 40 °C, 254 nm, for major diastereomer t_R (major) = 2.38 min; t_R (minor) = 2.91 min, e.r. = 81:19, for minor diastereomer t_R (major) = 2.64 min; t_R (minor) = 3.39 min, e.r. = 91:9.

(S)-1-((S,E)-1,3-Diphenylallyl)-3,5-dimethyl-1H-indene-2-carbaldehyde (**4f**)

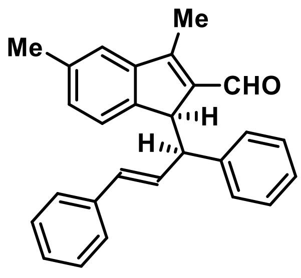

3,5-Dimethyl-1H-indene-2-carbaldehyde **1f** (34.4 mg, 0.2 mmol) and allyl acetate **2a** (25.2 mg, 0.1 mmol) were subjected to conditions A, the crude product was purified by column chromatography (silica gel, pentane/CH_2Cl_2, 1:1 to 1:4 v/v) to afford **4f** (31.2 mg, 0.085 mmol, 85% yield, d.r. (**4**:**5**) = 7.4:1, ee = 98%/56%).

Physical State: yellow oil.

R_f = 0.61 (pentane:Et_2O, 3:1 v/v; 2,4-DNP).

$[\alpha]_D$= +18.2° ($CHCl_3$, c 0.47).

^{1}H-NMR [$CDCl_3$, 400 MHz] δ: 10.08 (s, 1H), 7.52 (m, 1H), 7.58-6.96 (m, 10H), 6.87 (dd, J = 15.8, 7.4 Hz, 1H), 6.67 (m, 2H), 6.53 (d, J = 15.8, 1H), 4.63 (m, 1H), 4.31 (m, 1H), 2.44 (s, 3H), 2.27 (d, J = 2.0 Hz, 3H).

^{13}C-NMR [$CDCl_3$, 100 MHz] δ: 187.5, 157.2, 144.9, 144.1, 141.3, 139.7, 137.6, 137.4, 133.3, 130.9, 130.1, 128.7 (2C), 128.2 (2C), 127.7 (2C), 127.4, 126.6, 126.4 (2C), 125.1, 122.5, 53.2, 49.9, 21.6, 10.6.

For data corresponding to the minor diastereomer, see: **5f**

HRESI MS (*m*/*z*): $(M+Na)^+$ calcd. for $C_{27}H_{24}NaO$, 387.1719; found 387.1728.

UPCC: Daicel Chiralpak IC, CO_2/iPrOH = 85/15, 3 mL/min, 40 °C, 254 nm, for major diastereomer t_R (major) = 4.34 min; t_R (minor) = 5.53 min, e.r. = 99:1, for minor diastereomer t_R (major) = 3.71 min; t_R (minor) = 4.74 min, e.r. = 78:22.

(S)-1-((R,E)-1,3-Diphenylallyl)-3,5-dimethyl-1H-indene-2-carbaldehyde (**5f**)

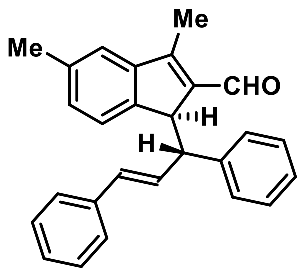

3,5-Dimethyl-1H-indene-2-carbaldehyde **1f** (34.4 mg, 0.2 mmol) and allyl acetate **2a** (25.2 mg, 0.1 mmol) were subjected to conditions B, the crude product was purified by column chromatography (silica gel, pentane/CH_2Cl_2, 1:1 to 1:4 v/v) to afford **5f** (26.6 mg, 0.073 mmol, 73% yield, d.r. (**4**:**5**) = 1:4.7, ee = 87%/94%).

Physical State: yellow oil.

R_f = 0.61 (pentane:Et_2O, 3:1 v/v; 2,4-DNP).

^{1}H-NMR [$CDCl_3$, 400 MHz] δ: 10.21 (s, 1H), 7.47-6.97 (m, 12H), 6.47-6.30 (m, 2H), 5.65 (dd, J = 15.9, 10.0 Hz, 1H), 4.81 (dd, J = 10.1, 4.2 Hz, 1H), 4.32 (m, 1H), 2.47 (d, J = 1.9 Hz, 3H), 2.42 (s, 3H).

^{13}C-NMR [$CDCl_3$, 100 MHz] δ: 187.3, 157.1, 144.9, 143.6, 143.2, 141.9, 137.5, 137.3, 132.9, 129.9, 128.5 (2C), 128.4 (2C), 128.4 (2C), 127.3, 126.6, 126.4 (2C), 126.3, 125.1, 122.4, 53.2, 47.6, 21.6, 10.8.

For data corresponding to the minor diastereomer, see: **4f**

HRESI MS (*m*/*z*): $(M+Na)^+$ calcd. for $C_{27}H_{24}NaO$, 387.1719; found 387.1721.

UPCC: Daicel Chiralpak IC, CO_2/*i*PrOH = 85/15, 3 mL/min, 40 °C, 254 nm, for major diastereomer t_R (major) = 3.75 min; t_R (minor) = 4.81 min, e.r. = 97:3, for minor diastereomer t_R (major) = 4.40 min; t_R (minor) = 5.61 min, e.r. = 94:6.

(*S*)-1-((*S*,*E*)-1,3-Diphenylallyl)-5-methoxy-3-methyl-1*H*-indene-2-carbaldehyde (**4g**)

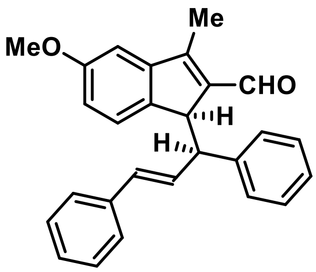

5-Methoxy-3-methyl-1*H*-indene-2-carbaldehyde **1g** (37.6 mg, 0.2 mmol) and allyl acetate **2a** (25.2 mg, 0.1 mmol) were subjected to conditions A, the crude product was purified by column chromatography (silica gel, pentane/CH_2Cl_2, 1:1 to 1:4 v/v) to afford **4g** (34.9 mg, 0.092 mmol, 92% yield, d.r. (**4**:**5**) = 8:1, ee = 97%/76%).

Physical State: yellow oil.

R_f = 0.42 (pentane:Et_2O, 3:1 v/v; 2,4-DNP).

$[\alpha]_D$= +2.8° ($CHCl_3$, *c* 0.25).

^{1}H-NMR [$CDCl_3$, 400 MHz] δ: 10.07 (s, 1H), 7.52 (d, *J* = 7.7 Hz, 1H), 7.45-6.73 (m, 11H), 6.67 (m, 2H), 6.53 (d, *J* = 15.4, 1H), 4.61 (t, *J* = 5.7, 1H), 4.28 (m, 1H), 3.86 (s, 3H), 2.25 (d, *J* = 1.9 Hz, 3H).

^{13}C-NMR [$CDCl_3$, 100 MHz] δ: 187.4, 159.7, 156.8, 142.7, 139.6, 139.1, 137.6, 133.3, 130.9, 128.7 (2C), 128.2 (2C), 127.7 (2C), 127.4, 126.6, 126.4 (2C), 125.9, 115.4, 106.7, 55.6, 52.9, 49.9, 21.6, 10.6.

For data corresponding to the minor diastereomer, see: **5g**

HRESI MS (*m/z*): $(M+Na)^+$ calcd. for $C_{27}H_{24}NaO_2$, 403.1669; found 403.1686.

UPCC: Daicel Chiralpak IC, CO_2/*i*PrOH = 85/15, 3 mL/min, 40 °C, 254 nm, for major diastereomer t_R (major) = 4.82 min; t_R (minor) = 6.19 min, e.r. = 98:2, for minor diastereomer t_R (major) = 4.25 min; t_R (minor) = 5.37 min, e.r. = 88:12.

(*S*)-1-((*R*,*E*)-1,3-Diphenylallyl)-5-methoxy-3-methyl-1*H*-indene-2-carbaldehyde (**5g**)

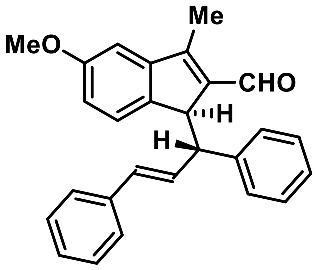

5-Methoxy-3-methyl-1*H*-indene-2-carbaldehyde **1g** (37.6 mg, 0.2 mmol) and allyl acetate **2a** (25.2 mg, 0.1 mmol) were subjected to conditions B, the crude product was purified by column chromatography (silica gel, pentane/CH_2Cl_2, 1:1 to 1:4 v/v) to afford **5g** (21.7 mg, 0.057 mmol, 57% yield, d.r. (**4**:**5**) = 1:6.2, ee = 97%/98%).

Physical State: yellow oil.

R_f = 0.42 (pentane:Et_2O, 3:1 v/v; 2,4-DNP).

$[\alpha]_D$= -12.4° ($CHCl_3$, *c* 0.15).

^{1}H-NMR [$CDCl_3$, 400 MHz] δ: 10.20 (s, 1H), 7.46-6.90 (m, 12H), 6.42 (d, *J* = 8.7 Hz, 1H), 6.33 (d, *J* = 15.7 Hz, 1H), 5.65 (dd, *J* = 15.6, 10.1 Hz, 1H), 4.79 (dd, *J* = 10.1, 4.0 Hz, 1H), 4.29 (m, 1H), 3.85 (s, 3H), 2.46 (d, *J* = 2.1 Hz, 3H).

^{13}C-NMR [$CDCl_3$, 100 MHz] δ: 187.2, 159.6, 156.7, 146.1, 143.6, 142.7, 138.3, 137.5, 133.0, 128.5 (2C), 128.5 (2C), 128.3 (2C), 127.3, 126.6, 126.3 (2C), 126.3, 126.0, 115.1, 122.4, 55.6, 52.9, 47.6, 10.8.

For data corresponding to the minor diastereomer, see: **4g**

HRESI MS (*m/z*): $(M+H)^+$ calcd. for $C_{27}H_{25}O_2$, 381.1849; found: 381.1848.

UPCC: Daicel Chiralpak IC, CO_2/*i*PrOH = 85/15, 3 mL/min, 40 °C, 254 nm, for major diastereomer t_R (major) = 4.26 min; t_R (minor) = 5.40 min, e.r. = 99:1, for minor diastereomer t_R (major) = 4.86 min; t_R (minor) = 6.23 min, e.r. = 98:2.

(*S*)-1-((*S*,*E*)-1,3-Diphenylallyl)-3,6-dimethyl-1*H*-indene-2-carbaldehyde (**4h**)

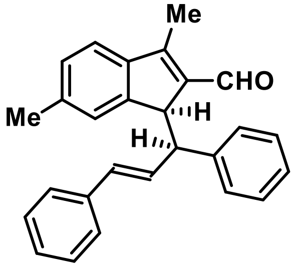

3,6-Dimethyl-1*H*-indene-2-carbaldehyde **1h** (34.4 mg, 0.2 mmol) and allyl acetate **2a** (25.2 mg, 0.1 mmol) were subjected to conditions A, the crude product was purified by column chromatography (silica gel, pentane/CH_2Cl_2, 1:1 to 1:4 v/v) to afford **4h** (32.0 mg, 0.088 mmol, 88% yield, d.r. (**4**:**5**) = 5.1:1, ee = 95%/70%).

Physical State: yellow oil.

R*f* = 0.32 (pentane:CH_2Cl_2, 4:6 v/v; 2,4-DNP).

^{1}H-NMR [$CDCl_3$, 400 MHz] δ: 10.05 (s, 1H), 7.51-6.91 (m, 11H), 6.85 (dd, *J* = 15.9, 7.5 Hz, 1H), 6.70-6.60 (m, 2H), 6.54 (d, *J* = 15.9 Hz, 1H), 4.68-4.53 (m, 1H), 4.38-4.19 (m, 1H), 2.51-2.36 (m, 3H), 2.32-2.16 (m, 3H).

^{13}C-NMR [$CDCl_3$, 100 MHz] δ: 187.2, 157.2, 147.2, 142.1, 140.3, 139.6, 139.3, 137.5, 133.1, 130.8, 128.6 (2C), 128.4, 128.0 (2C), 127.5 (2C), 127.2, 126.4, 126.2 (2C), 126.0, 121.6, 53.3, 49.8, 22.1, 10.5.

For data corresponding to the minor diastereomer, see: **5h**

HRESI MS (*m/z*): $(M+H)^+$ calcd. for $C_{27}H_{25}O$, 365.1900; found 365.1905.

UPCC: Daicel Chiralpak IC, CO_2/*i*PrOH = 85/15, 3 mL/min, 40 °C, 254 nm, for major diastereomer t_R (major) = 8.84 min; t_R (minor) = 11.33 min, e.r. = 98:2, for minor diastereomer t_R (major) = 7.29 min; t_R (minor) = 10.23 min, e.r. = 85:15.

(*S*)-1-((*R*,*E*)-1,3-Diphenylallyl)3,6-dimethyl-1*H*-indene-2-carbaldehyde (**5h**)

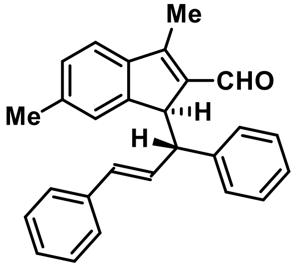

3,5-Dimethyl-1*H*-indene-2-carbaldehyde **1h** (34.4 mg, 0.2 mmol) and allyl acetate **2a** (25.2 mg, 0.1 mmol) were subjected to conditions B, the crude product was purified by column chromatography (silica gel, pentane/CH_2Cl_2, 1:1 to 1:4 v/v) to afford **5h** (24.7 mg, 0.068 mmol, 68% yield, d.r. (**4**:**5**) = 1:2.7, ee = 84%/82%).

Physical State: yellow oil.

R*f* = 0.32 (pentane:CH_2Cl_2, 4:6 v/v; 2,4-DNP).

^{1}H-NMR [$CDCl_3$, 400 MHz] δ: 10.19 (s, 1H), 7.51-6.95 (m, 12H), 6.45-6.23 (m, 2H), 5.65 (dd, *J* = 15.7, 10.0 Hz, 1H), 4.82 (dd, *J* = 10.0, 3.9 Hz, 1H), 4.39-4.19 (m, 1H) 2.51-2.39 (m, 3H), 2.29-2.23 (m, 3H).

^{13}C-NMR [$CDCl_3$, 100 MHz] δ: 187.0, 157.1, 146.3, 143.4, 142.0, 140.8, 139.1, 137.4, 132.8, 128.3 (2C), 128.3 (3C), 128.2 (2C), 127.5, 127.1, 126.5, 126.2, 126.2 (2C), 121.4, 53.2, 47.5, 21.9, 10.7.

For data corresponding to the minor diastereomer, see: **4h**

HRESI MS (*m*/*z*): $(M+Na)^+$ calcd. for $C_{27}H_{24}NaO$, 387.1719; found 387.1721.

UPCC: Daicel Chiralpak IC, CO_2/*i*PrOH = 85/15, 3 mL/min, 40 °C, 254 nm, for major diastereomer t_R (major) = 7.29 min; t_R (minor) = 10.24 min, e.r. = 91:9, for minor diastereomer t_R (major) = 8.87 min; t_R (minor) = 11.32 min, e.r. = 92:8.

(*S*)-1-((*S*,*E*)-1,3-Diphenylallyl)-6-methoxy-3-methyl-1*H*-indene-2-carbaldehyde (**4i**)

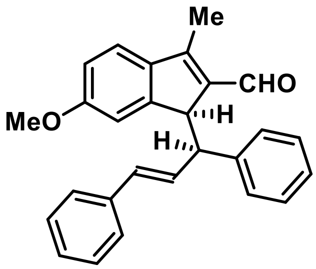

6-Methoxy-3-methyl-1*H*-indene-2-carbaldehyde **1i** (37.6 mg, 0.2 mmol) and allyl acetate **2a** (25.2 mg, 0.1 mmol) were subjected to conditions A, the crude product was purified by column chromatography (silica gel, pentane/CH_2Cl_2, 3:7) to afford **4i** (35.6 mg, 0.094 mmol, 94% yield, d.r. (**4**:**5**) = 3.6:1, ee = 93%/-8%).

Physical State: yellow oil.

R_f = 0.35 (pentane:CH_2Cl_2, 3:7 v/v; 2,4-DNP).

^{1}H-NMR [$CDCl_3$, 400 MHz] δ: 9.99 (s, 1H), 7.70-6.97 (m, 10H), 6.96-6.80 (m, 2H), 6.75-6.62 (m, 2H), 6.51 (d, *J* = 15.9 Hz, 1H), 4.60 (t, *J* = 6.3 Hz, 1H), 4.35-4.21 (m, 1H), 3.77 (s, 3H), 2.25 (d, *J* = 1.8 Hz, 3H).

^{13}C-NMR [$CDCl_3$, 100 MHz] δ: 186.6, 160.8, 157.2, 149.2, 139.5, 139.5, 137.6, 137.4, 133.0, 130.8, 128.6 (2C), 128.1 (2C), 127.6 (2C), 127.2, 126.5, 126.2 (2C), 122.8, 113.9, 110.8, 55.4, 53.2, 49.8, 10.5.

For data corresponding to the minor diastereomer, see: **5i**

HRESI MS (*m*/*z*): $(M+H)^+$ calcd. for $C_{27}H_{25}O_2$, 381.1849; found 381.1852.

UPCC: Daicel Chiralpak IC, CO_2/*i*PrOH = 85/15, 3 mL/min, 40 °C, 254 nm, for major diastereomer t_R (major) = 13.65 min; t_R (minor) = 16.65 min, e.r. = 96:4, for minor diastereomer t_R (major) = 14.93 min; t_R (minor) = 11.69 min, e.r. = 54:46.

(*S*)-1-((*R*,*E*)-1,3-Diphenylallyl)-6-methoxy-3-methyl-1*H*-indene-2-carbaldehyde (**5i**)

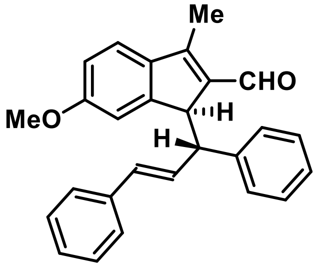

6-Methoxy-3-methyl-1*H*-indene-2-carbaldehyde **1i** (37.6 mg, 0.2 mmol) and allyl acetate **2a** (25.2 mg, 0.1 mmol) were subjected to conditions B, the crude product was purified by column chromatography (silica gel, pentane/CH_2Cl_2, 3:7) to afford **5i** (33.4 mg, 0.088 mmol, 88% yield, d.r. (**4**:**5**) = 1:2.9, ee = 72%/69%).

Physical State: yellow oil.

R*f* = 0.35 (pentane:CH_2Cl_2, 3:7 v/v; 2,4-DNP).

^{1}H-NMR [$CDCl_3$, 400 MHz] δ: 10.13 (s, 1H), 7.60-6.97 (m, 11H), 6.97-6.82 (m, 1H), 6.37 (d, *J* = 15.7 Hz, 1H), 6.00 (d, *J* = 2.3 Hz, 1H), 5.63 (dd, *J* = 15.7, 10.0 Hz, 1H), 4.87 (dd, *J* = 10.0, 4.2 Hz, 1H), 4.36-4.23 (m, 1H), 3.58 (s, 3H), 2.45 (d, *J* = 2.0 Hz, 3H).

^{13}C-NMR [$CDCl_3$, 100 MHz] δ: 186.4, 160.6, 157.2, 148.4, 143.4, 139.9, 137.4, 137.3, 132.9, 128.40 (2C), 128.35 (2C), 128.3 (2C), 127.1, 126.5, 126.2 (2C), 125.9, 122.6, 114.5, 110.2, 55.2, 53.3, 47.4, 10.7.

For data corresponding to the minor diastereomer, see: **4i**

HRESI MS (*m*/*z*): $(M+H)^+$ calcd. for $C_{27}H_{25}O_2$, 381.1849; found 381.1853.

UPCC: Daicel Chiralpak IC, CO_2/*i*PrOH = 85/15, 3 mL/min, 40 °C, 254 nm, for major diastereomer t_R (major) = 11.58 min; t_R (minor) = 14.84 min, e.r. = 85:15, for minor diastereomer t_R (major) = 13.57 min; t_R (minor) = 16.55 min, e.r. = 86:14.

(*S*)-1-((*S*,*E*)-1,3-Diphenylallyl)-6-fluoro-3-methyl-1*H*-indene-2-carbaldehyde (**4j**)

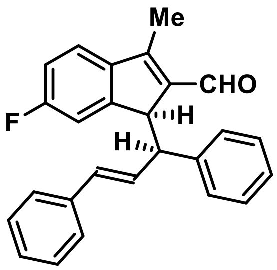

6-Fluoro-3-methyl-1*H*-indene-2-carbaldehyde **1j** (35.2 mg, 0.2 mmol) and allyl acetate **2a** (25.2 mg, 0.1 mmol) were subjected to conditions A, the crude product was purified by column chromatography (silica gel, pentane/CH_2Cl_2, 1:1 to 1:4 v/v) to afford **4j** (34.9 mg, 0.095 mmol, 95% yield, d.r. (**4**:**5**) = 2.3:1, ee = 94%/-60%).

Physical State: yellow oil.

R*f* = 0.53 (pentane:Et_2O, 3:1 v/v; 2,4-DNP).

^{1}H-NMR [$CDCl_3$, 400 MHz] δ: 10.06 (s, 1H), 7.51-6.98 (m, 11H), 6.83 (dd, *J* = 16.0, 7.2 Hz, 1H), 6.70 (d, *J* = 7.2 Hz, 2H), 6.57 (d, *J* = 15.9 Hz, 1H), 4.63 (t, *J* = 6.1 Hz, 1H), 4.34 (m, 1H), 2.28 (d, *J* = 2.0 Hz, 3H).

^{13}C-NMR [$CDCl_3$, 100 MHz] δ: 187.1, 163.5 (d, *J* = 250 Hz), 156.1 (d, *J* = 1.1 Hz), 149.4 (d, *J* = 9.1 Hz), 141.1 (d, *J* = 3.7 Hz), 140.8 (d, *J* = 1.9 Hz), 139.3, 137.3, 132.4, 131.5, 128.8 (2C), 128.1 (2C), 127.8 (2C), 127.6, 126.8, 126.4 (2C), 123.1 (d, *J* = 9.4 Hz), 115.1 (d, *J* = 23.6 Hz), 113.0 (d, *J* = 23.7 Hz), 53.7 (d, *J* = 2.5 Hz), 49.7, 10.7.

^{19}F-NMR [$CDCl_3$, 376 MHz] δ: -110.2.

For data corresponding to the minor diastereomer, see: **5j**

HRESI MS (*m*/*z*): $(M+Na)^+$ calcd. for $C_{26}H_{21}FNaO$, 391.1469; found 391.1485.

UPCC: Daicel Chiralpak IC, CO_2/*i*PrOH = 85/15, 3 mL/min, 40 °C, 254 nm, for major diastereomer t_R (major) = 4.68 min; t_R (minor) = 6.30 min, e.r. = 97:3, for minor diastereomer t_R (major) = 5.62 min; t_R (minor) = 4.32 min, e.r. = 80:20.

(*S*)-1-((*R*,*E*)-1,3-Diphenylallyl)-6-fluoro-3-methyl-1*H*-indene-2-carbaldehyde (**5j**)

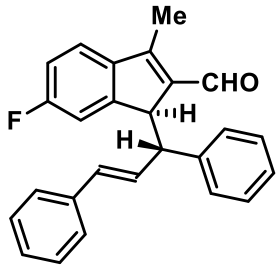

6-Fluoro-3-methyl-1*H*-indene-2-carbaldehyde **1j** (35.2 mg, 0.2 mmol) and allyl acetate **2a** (25.2 mg, 0.1 mmol) were subjected to conditions B, the crude product was purified by column chromatography (silica gel, pentane/CH_2Cl_2, 1:1 to 1:4 v/v) to afford **5j** (32.9 mg, 0.083 mmol, 83% yield, d.r. (**4**:**5**) = 1:2.8, ee = 66%/67%).

Physical State: yellow oil.

R*f* = 0.53 (pentane:Et_2O, 3:1 v/v; 2,4-DNP).

^{1}H-NMR [$CDCl_3$, 400 MHz] δ: 10.19 (s, 1H), 7.63-6.98 (m, 12H), 6.37 (d, *J* = 15.7 Hz, 1H), 6.24 (dd, *J* = 9.1, 2.2 Hz, 1H), 5.64 (dd, *J* = 15.7, 10.1 Hz, 1H), 4.85 (dd, *J* = 10.0, 4.0 Hz, 1H), 4.34 (broad s, 1H), 2.47 (d, *J* = 1.9 Hz, 3H).

^{13}C-NMR [$CDCl_3$, 100 MHz] δ: 186.8, 163.4 (d, *J* = 249.2 Hz), 156.0 (d, *J* = 1.0 Hz), 148.6 (d, *J* = 9.1 Hz), 142.9, 141.6 (d, *J* = 3.7 Hz), 140.7 (d, *J* = 2.1 Hz), 137.3, 133.4, 128.7 (2C), 128.6 (2C), 128.3 (2C), 127.4, 126.9, 126.4 (2C), 125.6, 123.0 (d, *J* = 9.4 Hz), 115.0 (d, *J* = 23.6 Hz), 113.1 (d, *J* = 23.9 Hz), 53.7 (d, *J* = 2.4 Hz), 47.4, 10.9.

^{19}F-NMR [$CDCl_3$, 376 MHz] δ: -110.3.

For data corresponding to the minor diastereomer, see: **4j**

HRESI MS (*m/z*): $(M+Na)^+$ calcd. for $C_{26}H_{21}FNaO$, 391.1469; found 391.1484.

UPCC: Daicel Chiralpak IC, CO_2/*i*PrOH = 85/15, 3 mL/min, 40 °C, 254 nm, for major diastereomer t_R (major) = 4.29 min; t_R (minor) = 5.61 min, e.r. = 83:17, for minor diastereomer t_R (major) = 4.67 min; t_R (minor) = 6.29 min, e.r. = 83:17.

(*S*)-1-((*S*,*E*)-1,3-Diphenylallyl)-6-chloro-3-methyl-1*H*-indene-2-carbaldehyde (**4k**)

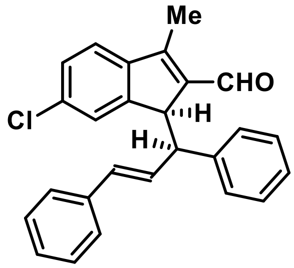

6-Chloro-3-methyl-1*H*-indene-2-carbaldehyde **1k** (38.5 mg, 0.2 mmol) and allyl acetate **2a** (25.2 mg, 0.1 mmol) were subjected to conditions A, the crude product was purified by column chromatography (silica gel, pentane/CH_2Cl_2, 1:1 to 1:4 v/v) to afford **4k** (30.3 mg, 0.079 mmol, 78% yield, d.r. (**4**:**5**) = 2.4:1, ee = 92%/-30%).

Physical State: yellow oil.

R*f* = 0.32 (pentane:CH_2Cl_2, 4:6 v/v; 2,4-DNP).

^{1}H-NMR [$CDCl_3$, 400 MHz] δ: 10.08 (s, 1H), 7.59 (d, *J* = 2.2 Hz, 1H), 7.49-7.30 (m, 5H), 7.30-7.10 (m, 2H), 7.10-7.01 (m, 3H), 6.82 (dd, *J* = 15.9, 7.5 Hz, 1H), 6.76-6.66 (m, 2H), 6.58 (d, *J* = 15.9 Hz, 1H), 4.61 (t, *J* = 6.1 Hz, 1H), 4.33 (d, *J* = 3.3 Hz, 1H), 2.27 (d, *J* = 1.9 Hz, 3H).

^{13}C-NMR [$CDCl_3$, 100 MHz] δ: 187.4, 155.8, 148.5, 143.2, 141.3, 139.3, 137.3, 135.1, 132.3, 131.5, 128.8 (2C), 128.1 (2C), 128.1, 127.9 (2C), 127.6, 126.8, 126.4 (2C), 125.7, 122.8, 53.8, 49.8, 10.6.

For data corresponding to the minor diastereomer, see: **5k**

HRESI MS (*m/z*): $(M+H)^+$ calcd. for $C_{26}H_{22}ClO$, 385.1354; found 385.1374.

UPCC: Daicel Chiralpak IC, CO_2/*i*PrOH = 90/10, 2 mL/min, 40 °C, 309 nm, for major diastereomer t_R (major) = 9.82 min; t_R (minor) = 13.18 min, e.r. = 96:4, for minor diastereomer t_R (major) = 12.26 min; t_R (minor) = 9.06 min, e.r. = 65:35.

(*S*)-1-((*R*,*E*)-1,3-Diphenylallyl)-6-chloro-3-methyl-1*H*-indene-2-carbaldehyde (**5k**)

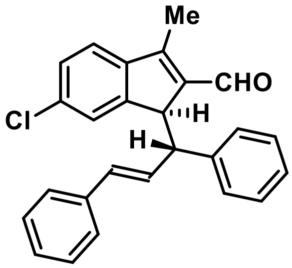

6-Chloro-3-methyl-1*H*-indene-2-carbaldehyde **1k** (38.3 mg, 0.2 mmol) and allyl acetate **2a** (25.2 mg, 0.1 mmol) were subjected to conditions B, the crude product was purified by column chromatography (silica gel, pentane/CH_2Cl_2, 1:1 to 1:4 v/v) to afford **5k** (33.4 mg, 0.087 mmol, 87% yield, d.r. (**4**:**5**) = 1:2.3, ee = 54%/70%).

Physical State: yellow oil.

R*f* = 0.32 (pentane:CH_2Cl_2, 4:6 v/v; 2,4-DNP).

^{1}H-NMR [$CDCl_3$, 400 MHz] δ: 10.21 (s, 1H), 7.49-7.30 (m, 8H), 7.30-7.10 (m, 4H), 6.49 (d, *J* = 1.3 Hz, 1H), 6.37 (d, *J* = 15.7 Hz, 1H), 5.65 (dd, *J* = 15.7, 10.1 Hz, 1H), 4.83 (dd, *J* = 10.1, 4.0 Hz, 1H), 4.33 (d, *J* = 1.6 Hz, 1H), 2.47 (d, *J* = 1.8 Hz, 3H).

^{13}C-NMR [$CDCl_3$, 100 MHz] δ: 187.1, 155.8, 147.6, 143.2, 142.9, 141.8, 137.2, 135.0, 133.3, 128.6 (2C), 128.5 (2C), 128.2 (2C), 128.0, 127.4, 127.0, 126.4 (2C), 125.9, 125.5, 122.7, 53.7, 47.5, 10.8.

For data corresponding to the minor diastereomer, see: **4k**

HRESI MS (*m/z*): $(M+H)^+$ calcd. for $C_{26}H_{22}ClO$, 385.1354; found 385.1374.

UPCC: Daicel Chiralpak IC, CO_2/*i*PrOH = 90/10, 2 mL/min, 40 °C, 309 nm, for major diastereomer t_R (major) = 8.98 min; t_R (minor) = 12.24 min, e.r. = 85:15, for minor diastereomer t_R (major) = 9.82 min; t_R (minor) = 13.16 min, e.r. = 77:23.

(*S*)-1-((*S*,*E*)-1,3-Diphenylallyl)-6-bromo-3-methyl-1*H*-indene-2-carbaldehyde (**4l**)

6-Bromo-3-methyl-1*H*-indene-2-carbaldehyde **1l** (47.2 mg, 0.2 mmol) and allyl acetate **2a** (25.2 mg, 0.1 mmol) were subjected to conditions A, the crude product was purified by column chromatography (silica gel, pentane/CH_2Cl_2, 1:1 to 1:4 v/v) to afford **4l** (34.1 mg, 0.079 mmol, 79% yield, d.r. = 2.2:1, ee = 90%/-14%).

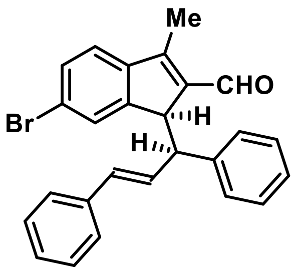

Physical State: yellow oil.

R*f* = 0.34 (pentane:CH_2Cl_2, 4:6 v/v; 2,4-DNP).

^{1}H-NMR [$CDCl_3$, 400 MHz] δ: 10.08 (s, 1H), 7.74 (d, *J* = 1.4 Hz, 1H), 7.5--7.49 (m, 1H), 7.46-7.39 (m, 3H), 7.39-7.32 (m, 1H), 7.30-7.11 (m, 2H), 7.10-6.99 (m, 3H),

6.80 (dd, J = 15.9, 7.5 Hz, 1H), 6.73-6.66 (m, 2H), 6.61 (dd, J = 9.4, 1.8 Hz, 1H), 4.58 (t, J = 6.1 Hz, 1H), 4.31 (d, J = 3.3 Hz, 1H), 2.27 (d, J = 1.9 Hz, 3H).

^{13}C-NMR [$CDCl_3$, 100 MHz] δ: 187.4, 155.7, 148.7, 143.6, 141.3, 139.4, 137.4, 132.4, 131.6, 130.9, 128.8 (2C), 128.7, 128.1 (2C), 127.9 (2C), 127.6, 126.9, 126.4 (2C), 123.4, 123.1, 53.8, 49.9, 10.6.

For data corresponding to the minor diastereomer, see: **5l**

HRESI MS (*m/z*): $(M+H)^+$ calcd. for $C_{26}H_{22}BrO$, 429.0849; found 429.0869.

UPCC: Daicel Chiralpak ID, CO_2/*i*PrOH = 99/1 to 60/40 over 4 min, 3 mL/min, 40 °C, 254 nm, for major diastereomer t_R (major) = 4.31 min; t_R (minor) = 4.86 min, e.r. = 95:5, for minor diastereomer t_R (major) = 4.68 min; t_R (minor) = 4.22 min, e.r. = 57:43.

(*S*)-1-((*R*,*E*)-1,3-Diphenylallyl)-6-bromo-3-methyl-1*H*-indene-2-carbaldehyde (**5l**)

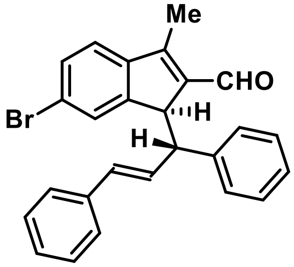

3-Methyl-1*H*-indene-2-carbaldehyde **1l** (47.4 mg, 0.2 mmol) and allyl acetate **2a** (25.2 mg, 0.1 mmol) were subjected to conditions B, the crude product was purified by column chromatography (silica gel, pentane/CH_2Cl_2, 1:1 to 1:4 v/v) to afford **5l** (33.6 mg, 0.078 mmol, 78% yield, d.r. (**4**:**5**) = 1:2.5, ee = 58%/71%).

Physical State: yellow oil.

R*f* = 0.34 (pentane:CH_2Cl_2, 4:6 v/v; 2,4-DNP).

^{1}H-NMR [$CDCl_3$, 400 MHz] δ: 10.21 (s, 1H), 7.55-7.49 (m, 1H), 7.46-7.39 (m, 3H), 7.39-7.32 (m, 4H), 7.30-7.11 (m, 4H), 6.56 (s, 1H), 6.36 (d, J = 15.7 Hz, 1H), 5.64 (dd, J = 15.7, 10.1 Hz, 1H), 4.82 (dd, J = 10.1, 4.1 Hz, 1H), 4.31 (d, J = 3.3 Hz, 1H), 2.46 (d, J = 1.9 Hz, 3H).

^{13}C-NMR [$CDCl_3$, 100 MHz] δ: 187.1, 155.7, 147.8, 143.6, 142.9, 141.7, 137.3, 133.3, 130.9, 128.9, 128.6 (2C), 128.5 (2C), 128.3 (2C), 127.4, 127.0, 126.4 (2C), 125.6, 123.4, 122.9, 53.8, 47.6, 10.7.

For data corresponding to the minor diastereomer, see: **4l**

HRESI MS (*m/z*): $(M+H)^+$ calcd. for $C_{26}H_{22}BrO$, 429.0849; found 429.0843

UPCC: Daicel Chiralpak ID, CO_2/*i*PrOH = 99/1 to 60/40 over 4 min, 3 mL/min, 40 °C, 254 nm, for major diastereomer t_R (major) = 4.21 min; t_R (minor) = 4.68 min, e.r. = 85:15, for minor diastereomer t_R (major) = 4.32 min; t_R (minor) = 4.84 min, e.r. = 79:21.

(*S*)-1-((*S*,*E*)-1,3-Diphenylallyl)-3-methyl-6-(methylthio)-1*H*-indene-2-carbaldehyde (**4m**)

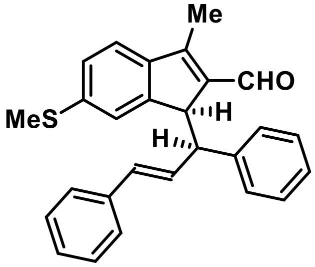

3-Methyl-1*H*-indene-2-carbaldehyde **1m** (40.8 mg, 0.2 mmol) and allyl acetate **2a** (25.2 mg, 0.1 mmol) were subjected to conditions A, the crude product was purified by column chromatography (silica gel, pentane/CH_2Cl_2, 1:1 to 1:4 v/v) to afford **4m** (34.4 mg, 0.087 mmol, 87% yield, d.r. (**4**:**5**) = 2.2:1, ee = 93%/-19%).

Physical State: yellow oil.

R*f* = 0.18 (pentane:CH_2Cl_2, 4:6 v/v; 2,4-DNP).

^{1}H-NMR [$CDCl_3$, 400 MHz] δ: 10.04 (s, 1H), 7.49--7.11 (m, 8H), 7.10-7.02 (m, 3H), 6.85 (dd, J = 15.9, 7.3 Hz, 1H), 6.69 (d, J = 6.8 Hz, 2H), 6.49 (d, J = 15.9 Hz, 1H), 4.55 (t, J = 6.2 Hz, 1H), 4.32 (d, J = 3.8 Hz, 1H), 2.37 (s, 3H), 2.26 (d, J = 1.7 Hz, 3H).

^{13}C-NMR [$CDCl_3$, 100 MHz] δ: 187.2, 156.7, 147.9, 141.7, 140.6, 140.3, 139.7, 137.5, 133.2, 131.0, 128.7 (2C), 128.2 (2C), 127.9 (2C), 127.5, 126.8, 126.3 (2C), 125.6, 122.2, 122.0, 53.3, 50.1, 15.5, 10.6.

For data corresponding to the minor diastereomer, see: **5m**

HRESI MS (*m/z*): $(M+H)^+$ calcd. for $C_{27}H_{25}OS$, 397.1621; found 397.1640.

UPCC: Daicel Chiralpak IC, CO_2/*i*PrOH = 85/15, 3 mL/min, 40 °C, 254 nm, for major diastereomer t_R (major) = 6.67 min; t_R (minor) = 8.11 min, e.r. = 97:3, for minor diastereomer t_R (major) = 7.29 min; t_R (minor) = 5.99 min, e.r. = 60:40.

(*S*)-1-((*R*,*E*)-1,3-Diphenylallyl)-3-methyl-6-(methylthio)-1*H*-indene-2-carbaldehyde (**5m**)

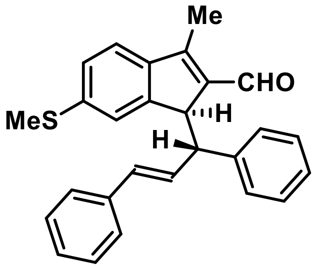

3-Methyl-1*H*-indene-2-carbaldehyde **1m** (40.7 mg, 0.2 mmol) and allyl acetate **2a** (25.2 mg, 0.1 mmol) were subjected to conditions B, the crude product was purified by column chromatography (silica gel, pentane/CH_2Cl_2, 1:1 to 1:4 v/v) to afford **5m** (33.6 mg, 0.085 mmol, 85% yield, d.r. (**4**:**5**) = 1:3.1, ee = 69%/69%).

Physical State: yellow oil.

R*f* = 0.18 (pentane:CH_2Cl_2, 4:6 v/v; 2,4-DNP).

^{1}H-NMR [$CDCl_3$, 400 MHz] δ: 10.18 (s, 1H), 7.49-7.11 (m, 12H), 6.38 (d, J = 15.7 Hz, 1H), 6.26 (s, 1H), 5.62 (dd, J = 15.7, 10.1 Hz, 1H), 4.87 (dd, J = 10.1, 4.3 Hz, 1H), 4.32 (d, J = 2.1 Hz, 1H), 2.46 (d, J = 1.8 Hz, 3H), 2.21 (s, 3H).

^{13}C-NMR [$CDCl_3$, 100 MHz] δ: 187.0, 156.8, 146.9, 143.5, 141.5, 140.5, 140.5, 137.4, 133.2, 128.5 (2C), 128.5 (2C), 128.5 (2C), 127.3, 126.7, 126.3 (2C), 125.8, 125.5, 121.8, 121.7, 53.4, 47.5, 15.0, 10.8.

For data corresponding to the minor diastereomer, see: **4m**

HRESI MS (*m/z*): $(M+Na)^+$ calcd. for $C_{27}H_{24}NaOS$, 419.1446; found 419.1464.

UPCC: Daicel Chiralpak IC, CO_2/*i*PrOH = 85/15, 3 mL/min, 40 °C, 254 nm, for major diastereomer t_R (major) = 6.03 min; t_R (minor) = 7.32 min, e.r. = 84:16, for minor diastereomer t_R (major) = 6.71 min; t_R (minor) = 8.15 min, e.r. = 85:15.

(*S*)-1-((*S*,*E*)-1,3-bis(4-Methylphenyl)allyl)-3-methyl-1*H*-indene-2-carbaldehyde (**4n**)

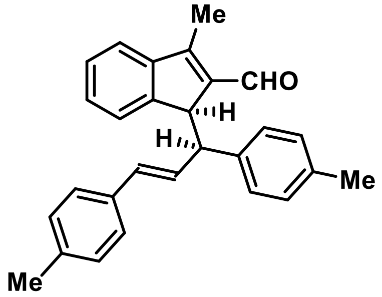

3-Methyl-1*H*-indene-2-carbaldehyde **1a** (31.6 mg, 0.2 mmol) and allyl acetate **2b** (28.0 mg, 0.1 mmol) were subjected to conditions A, the crude product was purified by column chromatography (silica gel, pentane/CH_2Cl_2, 1:1 v/v) to afford **4n** (32.6 mg, 0.086 mmol, 86% yield, d.r. (**4**:**5**) = 10.5:1, ee = 96%/41%).

Physical State: yellow oil.

R_f = 0.3 (pentane:CH_2Cl_2, 1:1 v/v; 2,4-DNP).

$[\alpha]_D$= +11.6° ($CHCl_3$, *c* 0.25).

^{1}H-NMR [$CDCl_3$, 400 MHz] δ: 10.09 (s, 1H), 7.69-7.58 (m, 1H), 7.49-7.05 (m, 7H), 6.88-6.67 (m, 3H), 6.58-6.37 (m, 3H), 4.60 (t, *J* = 6.2 Hz, 1H), 4.39-4.22 (m, 1H), 2.35 (s, 3H), 2.30 (d, *J* = 1.9 Hz, 3H), 2.19 (s, 3H).

^{13}C-NMR [$CDCl_3$, 100 MHz] δ: 187.4, 156.8, 146.9, 144.6, 141.0, 137.0, 136.5, 135.9, 134.7, 132.2, 130.5, 129.3 (2C), 128.8, 128.2 (2C), 127.9 (2C), 127.4, 126.1 (2C), 125.3, 121.8, 53.6, 49.2, 21.2, 20.9, 10.5.

For data corresponding to the minor diastereomer, see: **5n**

HRESI MS (*m*/*z*): $(M+H)^+$ calcd. for $C_{28}H_{27}O$, 379.2056; found 379.2053.

UPCC: Daicel Chiralpak ID, CO_2/*i*PrOH = 99/1 to 80/20 over 12 min, 3 mL/min, 40 °C, 254 nm, for major diastereomer t_R (major) = 8.59 min; t_R (minor) = 9.47 min, e.r. = 98:2, for minor diastereomer t_R (major) = 8.25 min; t_R (minor) = 10.56 min, e.r. = 71:29.

(*S*)-1-((*R*,*E*)-1,3-bis(4-Methylphenyl)allyl)-3-methyl-1*H*-indene-2-carbaldehyde (**5n**)

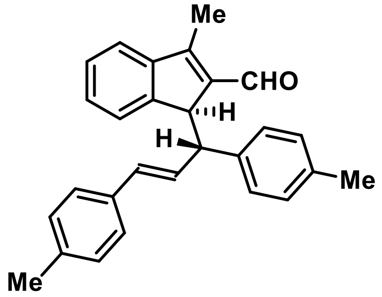

3-Methyl-1*H*-indene-2-carbaldehyde **1a** (31.6 mg, 0.2 mmol) and allyl acetate **2b** (28.0 mg, 0.1 mmol) were subjected to conditions B, the crude product was purified by column chromatography (silica gel, pentane/CH_2Cl_2, 1:1) to afford **5n** (26.0 mg, 0.069 mmol, 69% yield, d.r. (**4**:**5**) = 1:2.6, ee = 83%/83%).

Physical State: yellow oil.

R_f = 0.3 (pentane:CH_2Cl_2, 1:1 v/v; 2,4-DNP).

^{1}H-NMR [$CDCl_3$, 400 MHz] δ: 10.21 (s, 1H), 7.60-7.49 (d, *J* = 7.6 Hz, 1H), 7.46-7.29 (m, 3H), 7.24-7.11 (m, 2H), 7.05-6.95 (m, 3H), 6.85-6.75 (m, 1H), 6.62 (d, *J* = 7.6 Hz, 1H), 6.55-6.44 (m, 1H), 6.29 (d, *J* = 15.7 Hz, 1H), 5.56 (dd, *J* = 15.7, 10.0 Hz, 1H), 4.75 (dd, *J* = 10, 3.8 Hz, 1H), 4.38-4.28 (m, 1H), 2.48 (d, *J* = 1.8 Hz, 3H), 2.39 (s, 3H), 2.27 (s, 3H).

^{13}C-NMR [$CDCl_3$, 100 MHz] δ: 187.2, 156.7, 146.1, 144.5, 141.5, 140.4, 136.9, 135.9, 134.6, 132.5, 129.01 (2C), 128.99 (2C), 128.7, 128.1 (2C), 127.3, 126.1 (2C), 125.3, 125.1, 121.6, 53.5, 47.1, 21.10, 21.1, 10.08.

HRESI MS (*m*/*z*): $(M+H)^+$ calcd. for $C_{28}H_{27}O$, 379.2056; found 379.2054.

For data corresponding to the minor diastereomer, see: **4n**

UPCC: Daicel Chiralpak ID, CO_2/*i*PrOH = 99/1 to 80/20 over 12 min, 40 °C, 254 nm, for major diastereomer t_R (major) = 8.63 min; t_R (minor) = 11.45 min, e.r. = 92:8, for minor diastereomer t_R (major) = 9.10 min; t_R (minor) = 10.04 min, e.r. = 91:9.

(*S*)-1-((*S*,*E*)-1,3-bis(4-Methoxyphenyl)allyl)-3-methyl-1*H*-indene-2-carbaldehyde (**4o**)

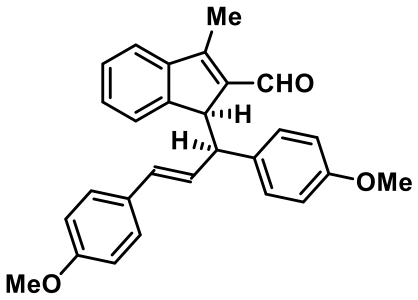

3-Methyl-1*H*-indene-2-carbaldehyde **1a** (31.6 mg, 0.2 mmol) and allyl acetate **2c** (31.2 mg, 0.1 mmol) were subjected to conditions A, the crude product was purified by column chromatography (silica gel, pentane/CH_2Cl_2, 1:1 to 0:10 v/v) to afford **4o** (31.4 mg, 0.076 mmol, 76% yield, d.r. (**4**:**5**) = 8.7:1, ee = 96%/47%).

Physical State: yellow oil.

R_f = 0.26 (CH_2Cl_2; 2,4-DNP).

$[\alpha]_D$= +19.9° ($CHCl_3$, *c* 0.65).

^{1}H-NMR [$CDCl_3$, 400 MHz] δ: 10.10 (s, 1H), 7.69-7.64 (m, 1H), 7.48-7.31 (m, 5H), 6.88 (d, *J* = 8.7 Hz, 2H), 6.71 (dd, *J* = 15.9, 7.1 Hz, 1H), 6.58-6.51 (m, 4H), 6.45 (d, *J* = 15.9 Hz, 1H), 4.66-4.54 (m, 1H), 4.30 (dd, *J* = 5.0, 1.9 Hz, 1H), 3.82 (s, 3H), 3.68 (s, 3H), 2.30 (d, *J* = 1.8 Hz, 3H).

^{13}C-NMR [$CDCl_3$, 100 MHz] δ: 187.6, 159.1, 158.2, 157.0, 147.1, 144.8, 141.2, 131.8, 131.3, 130.5, 130.2, 129.1 (2C), 129.0, 127.6, 127.5 (2C), 125.4, 122.0, 114.1 (2C), 113.0 (2C), 55.4, 55.2, 53.8, 48.9, 10.7.

For data corresponding to the minor diastereomer, see: **5o**

HRESI MS (*m/z*): $(M+Na)^+$ calcd. for $C_{28}H_{26}NaO_3$, 433.1774; found 433.1777.

UPCC: Daicel Chiralpak ID, CO_2/*i*PrOH = 99/1 to 60/40 over 4 min, 3 mL/min, 40 °C, 254 nm, for major diastereomer t_R (major) = 5.16 min; t_R (minor) = 5.41 min, e.r. = 98:2, for minor diastereomer t_R (major) = 4.89 min; t_R (minor) = 6.31 min, e.r. = 73:27.

(*S*)-1-((*R*,*E*)-1,3-bis(4-Methoxyphenyl)allyl)-3-methyl-1*H*-indene-2-carbaldehyde (**5o**)

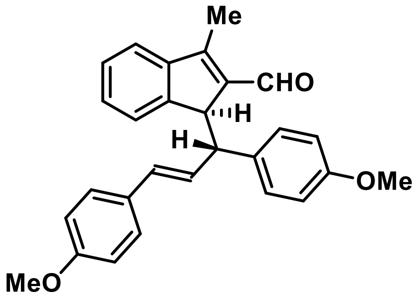

3-Methyl-1*H*-indene-2-carbaldehyde **1a** (31.6 mg, 0.2 mmol) and allyl acetate **2c** (31.2 mg, 0.1 mmol) were subjected to conditions B, the crude product was purified by column chromatography (silica gel, pentane/CH_2Cl_2, 1:1 to 0:10 v/v) to afford **5o** (35.4 mg, 0.086 mmol, 86% yield, d.r. (**4**:**5**) = 1:1.2, ee = 90%/77%).

Physical State: yellow oil.

R_f = 0.26 (CH_2Cl_2; 2,4-DNP).

^{1}H-NMR [$CDCl_3$, 400 MHz] δ: 10.22 (s, 1H), 7.53 (d, *J* = 7.6 Hz, 1H), 7.46-7.31 (m, 5H), 7.21 (t, *J* = 7.5 Hz, 1H), 7.03 (d, *J* = 8.7 Hz, 2H), 6.74 (d, *J* = 8.7 Hz, 2H), 6.61 (d, *J* = 7.5 Hz, 1H), 6.28 (d, *J* = 15.7 Hz, 1H), 5.46 (dd, *J* = 15.7, 10.0 Hz, 1H), 4.74 (dd, *J* = 10.0, 3.9 Hz, 1H), 4.31 (broad s, 1H), 3.85 (s, 3H), 3.75 (s, 3H), 2.48 (d, *J* = 1.8 Hz, 3H).

^{13}C-NMR [$CDCl_3$, 100 MHz] δ: 187.3, 159.0, 158.3, 156.9, 146.3, 144.7, 141.7, 135.7, 132.2, 130.3, 129.3 (2C), 128.8, 127.5, 127.4 (2C), 125.4, 124.2, 121.8, 113.9 (2C), 113.8 (2C), 55.5, 55.4, 53.8, 46.9, 10.8.

HRESI MS (*m*/*z*): $(M+Na)^+$ calcd. for $C_{28}H_{26}NaO_3$, 433.1774; found 433.1780.

For data corresponding to the minor diastereomer, see: **4o**

UPCC: Daicel Chiralpak ID, CO_2/*i*PrOH = 99/1 to 60/40 over 4 min, 40 °C, 254 nm, for major diastereomer t_R (major) = 4.88 min; t_R (minor) = 6.30 min, e.r. = 88:12, for minor diastereomer t_R (major) = 5.16 min; t_R (minor) = 5.40 min, e.r. = 95:5.

(*S*)-1-((*S*,*E*)-1,3-bis(4-Fluorophenyl)allyl)-3-methyl-1*H*-indene-2-carbaldehyde (**4p**)

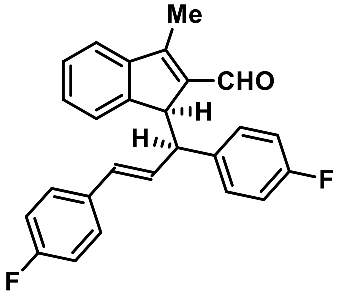

3-Methyl-1*H*-indene-2-carbaldehyde **1a** (31.6 mg, 0.2 mmol) and allyl acetate **2d** (28.8 mg, 0.1 mmol) were subjected to conditions A, the crude product was purified by column chromatography (silica gel, pentane/CH_2Cl_2, 1:1 v/v) to afford **4p** (29.8 mg, 0.077 mmol, 77% yield, d.r. (**4**:**5**) = 5.7:1, ee = 97%/28%).

Physical State: yellow oil.

R*f* = 0.3 (pentane:CH_2Cl_2, 1:1 v/v; 2,4-DNP).

^{1}H-NMR [$CDCl_3$, 400 MHz] δ: 10.10 (s, 1H), 7.86-7.13 (m, 7H), 7.11-6.93 (m, 2H), 6.82-6.51 (m, 4H), 6.46 (d, *J* = 16.0 Hz, 1H), 4.72-4.58 (m, 1H), 4.41-4.19 (m, 1H), 2.30 (d, *J* = 1.9 Hz, 3H).

^{13}C-NMR [$CDCl_3$, 100 MHz] δ: 187.3, 163.2 (d, *J* = 246 Hz), 160.5 (d, *J* = 244 Hz), 157.2, 146.4, 144.6, 140.7, 134.9 (d, *J* = 3.2 Hz), 133.4 (d, *J* = 3.2), 132.5 (d, *J* = 2.0 Hz), 129.9, 129.4 (d, J = 7.7 Hz, 2C), 129.0, 127.7 (d, *J* = 7.8 Hz, 2C) 127.7, 125.1, 122.0, 115.5 (d, *J* = 22 Hz, 2C), 114.4 (d, *J* = 21 Hz, 2C), 53.4, 48.6, 10.5.

^{19}F-NMR [$CDCl_3$, 376 MHz] δ: -114.8, -116.3.

For data corresponding to the minor diastereomer, see: **5p**

HRESI MS (*m*/*z*): $(M+H)^+$ calcd. for $C_{26}H_{21}F_2O$, 387.1555; found 387.1559.

UPCC: Daicel Chiralpak IB, CO_2/*i*PrOH = 99/1 to 80:20 over 12 min, 3 mL/min, 40 °C, 254 nm, for major diastereomer t_R (major) = 5.63 min; t_R (minor) = 6.46 min, e.r. = 99:1, for minor diastereomer t_R (major) = 5.29 min; t_R (minor) = 6.20 min, e.r. = 64:36.

(*S*)-1-((*R*,*E*)-1,3-bis(4-Fluorophenyl)allyl)-3-methyl-1*H*-indene-2-carbaldehyde (**5p**)

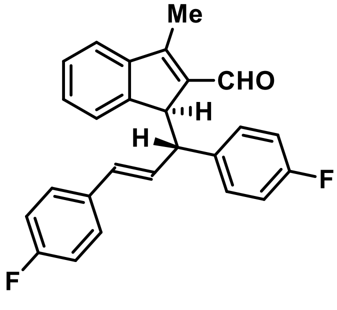

3-Methyl-1*H*-indene-2-carbaldehyde **1a** (31.6 mg, 0.2 mmol) and allyl acetate **2d** (28.8 mg, 0.1 mmol) were subjected to conditions B, the crude product was purified by column chromatography (silica gel, pentane/CH_2Cl_2, 1:1 to 0:10 v/v) to afford **5p** (24.1 mg, 0.062 mmol, 62% yield, d.r. (**4**:**5**) = 1:1.9, ee = 82%/81%).

Physical State: yellow oil.

R*f* = 0.32 (pentane:CH_2Cl_2, 4:6 v/v; 2,4-DNP).

^{1}H-NMR [$CDCl_3$, 400 MHz] δ: 10.23 (s, 1H), 7.55 (d, *J* = 7.6 Hz, 1H), 7.47-7.30 (m, 3H), 7.23 (t, *J* = 7.3 Hz, 1H), 7.11-6.99 (m, 4H), 6.89 (t, *J* = 8.7 Hz, 2H), 6.61 (d, *J* = 7.6 Hz, 1H), 6.30 (d, *J* = 15.7 Hz, 1H), 5.54 (dd, *J* = 15.7, 9.9 Hz, 1H), 4.77 (dd, *J* = 9.9, 4.0 Hz, 1H), 4.39-4.20 (m, 1H), 2.49 (d, *J* = 2.0 Hz, 3H).

^{13}C-NMR [$CDCl_3$, 100 MHz] δ: 187.3, 162.2 (d, *J* = 246.6 Hz), 161.7 (d, *J* = 244.9 Hz), 157.2, 145.9, 144.6, 141.4, 139.0 (d, *J* = 3.2 Hz), 133.4 (d, *J* = 3.3 Hz), 132.0, 129.7 (d, *J* = 7.8 Hz, 2C), 129.1, 127.8 (d, *J* = 8.2 Hz, 2C), 127.7, 125.7 (d, *J* = 2.1 Hz) 125.2, 122.0, 115.5 (d, *J* = 14.5 Hz, 2C), 115.2 (d, *J* = 14.1 Hz, 2C), 53.6, 47.0, 10.8.

^{19}F-NMR [$CDCl_3$, 376 MHz] δ: -114.8, -116.4.

For data corresponding to the minor diastereomer, see: **4p**

HRESI MS (*m/z*): $(M+H)^+$ calcd. for $C_{26}H_{21}F_2O$, 387.1560; found 387.1562.

UPCC: Daicel Chiralpak IB, CO_2/*i*PrOH = 99/1 to 80:20 over 12 min, 3 mL/min, 40 °C, 254 nm, for major diastereomer t_R (major) = 5.28 min; t_R (minor) = 6.18 min, e.r. = 90:10, for minor diastereomer t_R (major) = 5.68 min; t_R (minor) = 6.43 min, e.r. = 91:9.

(*S*)-1-((*S*,*E*)-1,3-bis(4-Chlorophenyl)allyl)-3-methyl-1*H*-indene-2-carbaldehyde (**4q**)

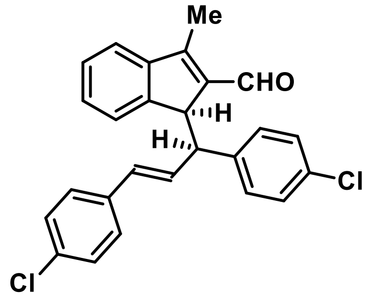

3-Methyl-1*H*-indene-2-carbaldehyde **1a** (31.6 mg, 0.2 mmol) and allyl acetate **2e** (32.1 mg, 0.1 mmol) were subjected to conditions A, the crude product was purified by column chromatography (silica gel, pentane/CH_2Cl_2, 1:1 to 0:10 v/v) to afford **4q** (34.1 mg, 0.081 mmol, 81% yield, d.r. (**4**:**5**) = 6.3:1, ee = 98%/10%).

Physical State: yellow oil.

R_f = 0.37 (pentane:CH_2Cl_2, 4:6 v/v; 2,4-DNP).

$[\alpha]_D$= +17.8° ($CHCl_3$, *c* 0.72).

^{1}H-NMR [$CDCl_3$, 400 MHz] δ: 10.10 (s, 1 H), 7.70-7.27 (m, 8H), 6.95 (dd, *J* = 6.3, 4.6 Hz, 2H), 6.81 (dd, *J* = 15.9, 7.0 Hz, 1H), 6.54 (d, *J* = 8.5 Hz, 2H), 6.43 (dd, *J* = 15.9, 1.1 Hz, 1H), 4.71-4.66 (m, 1H), 4.30 (dd, *J* = 5.0, 1.9 Hz, 1H), 2.31 (d, *J* = 1.91 Hz, 3H).

^{13}C-NMR [$CDCl_3$, 100 MHz] δ: 187.5, 157.6, 146.4, 144.7, 140.6, 137.7, 135.8, 133.2, 133.2, 132.4, 130.1, 129.5 (2C), 129.3, 128.9 (2C), 127.9 (2C), 127.9, 127.6 (2C), 125.2, 122.2, 53.3, 48.9, 10.7.

For data corresponding to the minor diastereomer, see: **5q**

HRESI MS (*m/z*): $(M+H)^+$ calcd. for $C_{26}H_{21}Cl_2O$, 419.0964; found 419.0966.

UPCC: Daicel Chiralpak IB, CO_2/*i*PrOH = 99/1 to 60:40 over 4 min, 3 mL/min, 40 °C, 254 nm, for major diastereomer t_R (major) = 3.99 min; t_R (minor) = 4.29 min, e.r. = 99:1, for minor diastereomer t_R (major) = 3.85 min; t_R (minor) = 4.23 min, e.r. = 55:45.

(*S*)-1-((*R*,*E*)-1,3-bis(4-Chlorophenyl)allyl)-3-methyl-1*H*-indene-2-carbaldehyde (**5q**)

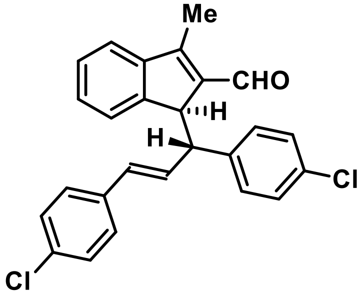

3-Methyl-1*H*-indene-2-carbaldehyde **1a** (31.5 mg, 0.2 mmol) and allyl acetate **2e** (32.1 mg, 0.1 mmol) were subjected to conditions B, the crude product was purified by column chromatography (silica gel, pentane/CH_2Cl_2, 1:1 to 0:10 v/v) to afford **5q** (30.5 mg, 0.073 mmol, 73% yield, d.r. (**4**:**5**) = 1:1.9, ee = 82%/85%).

Physical State: yellow oil.

R_f = 0.37 (pentane:CH_2Cl_2, 4:6 v/v; 2,4-DNP).

^{1}H-NMR [$CDCl_3$, 400 MHz] δ: 10.22 (s, 1H), 7.55 (d, *J* = 7.6 Hz, 1H), 7.48-7.28 (m, 5H), 7.23 (dd, *J* = 7.5, 0.8 Hz, 1H), 7.17 (d, *J* = 8.5 Hz, 2H), 7.01 (d, J = 8.5 Hz, 2H), 6.62 (d, *J* = 7.6 Hz, 1H), 6.29 (d, *J* = 15.7 Hz, 1H), 5.59 (dd, *J* = 15.7, 10.0 Hz, 1H), 4.77 (dd, *J* = 9.9, 3.8 Hz, 1H), 4.29 (broad s, 1H), 2.49 (d, *J* = 1.9 Hz, 3H).

^{13}C-NMR [$CDCl_3$, 100 MHz] δ: 187.2, 157.3, 145.7, 144.6, 141.7, 141.3, 135.7, 133.1, 132.5, 132.2, 129.7 (2C), 129.2, 128.7 (2C), 128.6 (2C), 127.8, 127.5 (2C), 126.5, 125.2, 122.0, 53.4, 47.2, 10.8.

For data corresponding to the minor diastereomer, see: **4q**

HRESI MS (*m*/*z*): $(M+H)^+$ calcd. for $C_{26}H_{21}Cl_2O$, 419.0964; found 419.0964.

UPCC: Daicel Chiralpak ID, CO_2/*i*PrOH = 90/10, 3 mL/min, 40 °C, 254 nm, for major diastereomer t_R (major) = 3.88 min; t_R (minor) = 4.26 min, e.r. = 92:8, for minor diastereomer t_R (major) = 4.04 min; t_R (minor) = 4.32 min, e.r. = 91:9.

(*S*)-1-((*S*,*E*)-1,3-bis(4-Bromophenyl)allyl)-3-methyl-1*H*-indene-2-carbaldehyde (**4r**)

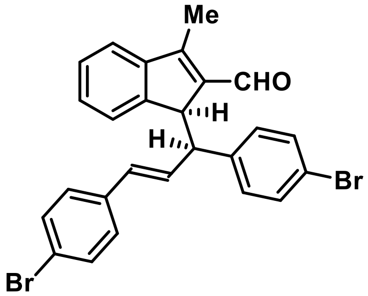

3-Methyl-1*H*-indene-2-carbaldehyde **1a** (31.6 mg, 0.2 mmol) and allyl acetate **2f** (41.1 mg, 0.1 mmol) were subjected to conditions A, the crude product was purified by column chromatography (silica gel, pentane/CH_2Cl_2, 1:1 to 0:10 v/v) to afford **4r** (41.2 mg, 0.081 mmol, 81% yield, d.r. (**4**:**5**) = 5.7:1, ee = 97%/-6%).

Physical State: yellow oil.

R_f = 0.37 (pentane:CH_2Cl_2, 4:6 v/v; 2,4-DNP).

^{1}H-NMR [$CDCl_3$, 400 MHz] δ: 10.12 (s, 1 H), 7.68-7.60 (m, 1H), 7.55-7.25 (m, 7H), 7.14 (d, *J* = 8.3 Hz, 2H), 6.85 (dd, *J* = 15.9, 7.0 Hz, 1H), 6.51 (d, *J* = 8.3 Hz, 2H), 6.44 (d, *J* = 15.9 Hz, 1H), 4.69 (t, *J* = 6.0 Hz, 1H), 4.41-4.26 (m, 1H), 2.35 (d, *J* = 1.4 Hz, 3H).

^{13}C-NMR [$CDCl_3$, 100 MHz] δ: 187.4, 157.5, 146.2, 144.6, 140.5, 138.1, 136.1, 133.2, 131.8 (2C), 130.8 (2C), 130.1, 129.8, 129.2 (2C), 127.8 (2C), 125.1, 122.2, 121.2, 120.6, 53.1, 48.9, 10.6.

For data corresponding to the minor diastereomer, see: **5r**

HRESI MS (*m*/*z*): $(M+H)^+$ calcd. for $C_{26}H_{21}Br_2O$, 506.9954; found 506.9953.

UPCC: Daicel Chiralpak IB, CO_2/CH_2Cl_2 = 99/1 to 60/40 over 4 min, 3 mL/min, 40 °C, 254 nm, for major diastereomer t_R (major) = 6.29 min; t_R (minor) = 6.74 min, e.r. = 99:1, for minor diastereomer t_R (major) = 6.63 min; t_R (minor) = 6.00 min, e.r. = 53:47.

(*S*)-1-((*R*,*E*)-1,3-bis(4-Bromophenyl)allyl)-3-methyl-1*H*-indene-2-carbaldehyde (**5r**)

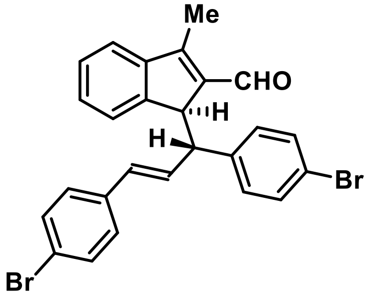

3-Methyl-1*H*-indene-2-carbaldehyde **1a** (31.6 mg, 0.2 mmol) and allyl acetate **2f** (41.0 mg, 0.1 mmol) were subjected to conditions B, the crude product was purified by column chromatography (silica gel, pentane/CH_2Cl_2, 1:1 to 0:10 v/v) to afford **5r** (35.3 mg, 0.070 mmol, 70% yield, d.r. = 1:1.9, ee = 90%/90%).

Physical State: yellow oil.

R*f* = 0.37 (pentane:CH_2Cl_2, 4:6 v/v; 2,4-DNP).

^{1}H-NMR [$CDCl_3$, 400 MHz] δ: 10.22 (s, 1H), 7.57-7.20 (m, 9H), 6.95 (d, *J* = 8.5 Hz, 2H), 6.63 (d, *J* = 7.6 Hz, 1H), 6.27 (d, *J* = 15.7 Hz, 1H), 5.60 (dd, *J* = 15.7, 10.0 Hz, 1H), 4.75 (dd, *J* = 9.9, 3.8 Hz, 1H), 4.29 (broad s, 1H), 2.48 (d, *J* = 1.9 Hz, 3H).

^{13}C-NMR [$CDCl_3$, 100 MHz] δ: 187.2, 157.3, 145.7, 144.6, 142.2, 141.2, 136.1, 132.3, 131.7 (2C), 131.6 (2C), 130.1 (2C), 129.2, 127.9 (2C), 127.8, 126.5, 125.2, 122.1, 121.3, 120.6, 53.3, 47.3, 10.8.

For data corresponding to the minor diastereomer, see: **4r**

HRESI MS (*m/z*): $(M+H)^+$ calcd. for $C_{26}H_{21}Br_2O$, 506.9954; found 506.9950.

UPCC: Daicel Chiralpak IB, CO_2/CH_2Cl_2 = 99/1 to 60/40 over 4 min, 3 mL/min, 40 °C, 254 nm, for major diastereomer t_R (major) = 5.93 min; t_R (minor) = 6.58 min, e.r. = 95:5, for minor diastereomer t_R (major) = 6.25 min; t_R (minor) = 6.69 min, e.r. = 95:5.

(*S*)-1-((*S*,*E*)-1,3-bis(4-(Trifluoromethyl)phenyl)allyl)-3-methyl-1*H*-indene-2-carbaldehyde (**4s**)

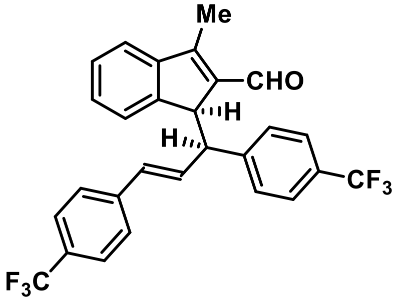

3-Methyl-1*H*-indene-2-carbaldehyde **1a** (31.6 mg, 0.2 mmol) and allyl acetate **2g** (38.8 mg, 0.1 mmol) were subjected to conditions A, the crude product was purified by column chromatography (silica gel, pentane/CH_2Cl_2, 1:1 to 0:10 v/v) to afford **4s** (40.0 mg, 0.082 mmol, 82% yield, d.r. (**4**:**5**) = 3.2:1, ee = 97%/-15%).

Physical State: yellow oil.

R*f* = 0.47 (pentane:CH_2Cl_2, 4:6 v/v; 2,4-DNP).

^{1}H-NMR [$CDCl_3$, 400 MHz] δ: 10.10 (s, 1H), 7.67-7.40 (m, 8H), 7.31-7.24 (m, 2H), 6.96 (dd, *J* = 16.0, 7.1 Hz, 1H), 6.74 (d, *J* = 8.1 Hz, 2H), 6.52 (dd, *J* = 16.0, 1.4 Hz, 1H), 4.86-4.74 (m, 1H), 4.36 (dd, *J* = 5.3, 2.1 Hz, 1H), 2.31 (d, *J* = 2.0 Hz, 3H).

^{13}C-NMR [$CDCl_3$, 126 MHz] δ: 187.5, 157.8, 146.1, 144.7, 143.1 (q, *J* = 1.3 Hz), 140.6 (q, *J* = 1.3 Hz), 140.4, 134.9, 130.4, 129.5 (q, *J* = 32.4 Hz, 2C), 129.4, 129.1 (q, *J* = 32.3 Hz, 2C), 128.5,

128.1, 126.6, 125.8 (q, J = 3.9 Hz, 2C), 125.1, 124.8 (q, J = 3.8 Hz, 2C), 124.3 (q, J = 271.9 Hz), 124.2 (q, J = 271.9 Hz), 122.4, 53.1, 49.4, 10.7.

^{19}F-NMR [$CDCl_3$, 376 MHz] δ: -62.4, -62.5.

For data corresponding to the minor diastereomer, see: **5s**

HRESI MS (*m/z*): $(M+H)^+$ calcd. for $C_{28}H_{21}F_6O$, 487.1491; found 487.1499.

UPCC: Daicel Chiralpak IA, CO_2/*i*PrOH = 99/1 to 60/40 over 8 min, 3 mL/min, 40 °C, 254 nm, for major diastereomer t_R (major) = 3.46 min; t_R (minor) = 3.87 min, e.r. = 98:2, for minor diastereomer t_R (major) = 3.79 min; t_R (minor) = 3.25 min, e.r. = 58:42.

(*S*)-1-((*R*,*E*)-1,3-bis(4-(Trifluoromethyl)phenyl)allyl)-3-methyl-1*H*-indene-2-carbaldehyde (**5s**)

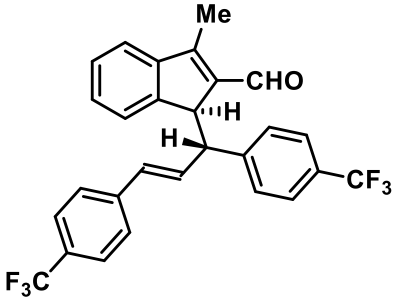

3-Methyl-1*H*-indene-2-carbaldehyde **1a** (31.6 mg, 0.2 mmol) and allyl acetate **2g** (38.8 mg, 0.1 mmol) were subjected to conditions B, the crude product was purified by column chromatography (silica gel, pentane/CH_2Cl_2, 1:1 to 0:10 v/v) to afford **5s** (31.3 mg, 0.064 mmol, 64% yield, d.r. (**4**:**5**) = 1:1.7, ee = 82%/80%).

Physical State: yellow oil.

R_f = 0.47 (pentane:CH_2Cl_2, 4:6 v/v; 2,4-DNP).

^{1}H-NMR [$CDCl_3$, 400 MHz] δ: 10.24 (s, 1H), 7.69-7.37 (m, 8H), 7.31-7.23 (m, 1H), 7.19 (d, J = 8.1 Hz, 2H), 6.60 (d, J = 7.6 Hz, 1H), 6.40 (d, J = 15.8 Hz, 1H), 5.76 (dd, J = 15.8, 10.0 Hz, 1H), 4.90 (dd, J = 9.9, 3.5 Hz, 1H), 4.35 (broad s, 1H), 2.50 (d, J = 1.9 Hz, 3H).

^{13}C-NMR [$CDCl_3$, 100 MHz] δ: 187.3, 157.6, 147.1 (q, J = 1.2 Hz), 145.4, 144.6, 141.0, 140.4 (q, J = 1.4 Hz), 132.5, 129.4 (q, J = 32.3 Hz, 2C), 129.3, 129.1 (q, J = 32.5 Hz, 2C), 128.7, 128.1, 128.0, 126.5, 125.6 (q, J = 3.8 Hz, 2C), 125.5 (q, J = 3.7 Hz, 2C), 125.1, 124.4 (q, J = 272.0 Hz), 124.0 (q, J = 272.0 Hz), 122.2, 53.1, 47.6, 10.8.

^{19}F-NMR [$CDCl_3$, 376 MHz] δ: -62.3, -62.5.

For data corresponding to the minor diastereomer, see: **4s**

HRESI MS (*m/z*): $(M+H)^+$ calcd. for $C_{28}H_{21}F_6O$, 487.1491; found 487.1499.

UPCC: Daicel Chiralpak IA, CO_2/*i*PrOH = 99/1 to 60/40 over 8 min, 3 mL/min, 40 °C, 254 nm, for major diastereomer t_R (major) = 3.24 min; t_R (minor) = 3.79 min, e.r. = 90:10, for minor diastereomer t_R (major) = 3.45 min; t_R (minor) = 3.88 min, e.r. = 91:9.

(S)-1-((S,E)-1,3-bis(4-Nitrophenyl)allyl)-3-methyl-1H-indene-2-carbaldehyde (**4t**)

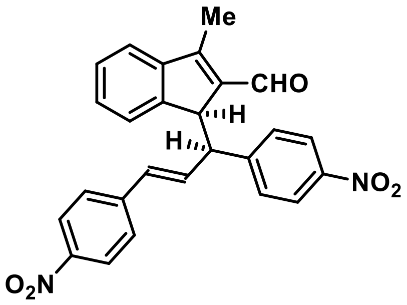

3-Methyl-1H-indene-2-carbaldehyde **1a** (31.6 mg, 0.2 mmol) and allyl acetate **2h** (34.2 mg, 0.1 mmol) were subjected to conditions A, the crude product was purified by column chromatography (silica gel, pentane/CH_2Cl_2, 1:1 to 0:10 v/v) to afford **4t** (33.4 mg, 0.076 mmol, 76% yield, d.r. (**4**:**5**) = 1.9:1, ee = 90%/-19%).

Physical State: yellow oil.

R_f = 0.43 (CH_2Cl_2; 2,4-DNP).

^{1}H-NMR [$CDCl_3$, 400 MHz] δ: 10.11 (s, 1H), 8.22 (d, J = 8.7 Hz, 2H), 7.87 (d, J = 8.8 Hz, 2H), 7.65-7.40 (m, 5H), 7.25 (m, 1H), 7.05 (dd, J = 16.0, 7.1 Hz, 1H), 6.80 (t, J = 9.0 Hz, 2H), 6.56 (d, J = 15.9 Hz, 1H), 4.95-4.89 (m, 1H), 4.38 (dd, J = 4.8, 2.0 Hz, 1H), 2.32 (d, J = 1.9 Hz, 3H).

^{13}C-NMR [$CDCl_3$, 100 MHz] δ: 187.4, 158.1, 147.2, 147.0, 146.3, 145.5, 144.6, 143.3, 139.9, 136.4, 130.3, 129.7, 129.0 (2C), 128.4, 127.0 (2C), 125.0, 124.3 (2C), 123.1 (2C), 122.6, 52.9, 49.4, 10.7.

For data corresponding to the minor diastereomer, see: **5t**

HRESI MS (*m*/*z*): $(M+H)^+$ calcd. for $C_{26}H_{21}N_2O_5$, 441.1445; found 441.1446.

UPCC: Daicel Chiralpak IB, CO_2/*i*PrOH = 99/1 to 60/40 over 4 min, 3 mL/min, 40 °C, 254 nm, for major diastereomer t_R (major) = 4.92 min; t_R (minor) = 5.60 min, e.r. = 95:5, for minor diastereomer t_R (major) = 5.42 min; t_R (minor) = 4.82 min, e.r. = 59:41.

(S)-1-((R,E)-1,3-bis(4-Nitrophenyl)allyl)-3-methyl-1H-indene-2-carbaldehyde (**5t**)

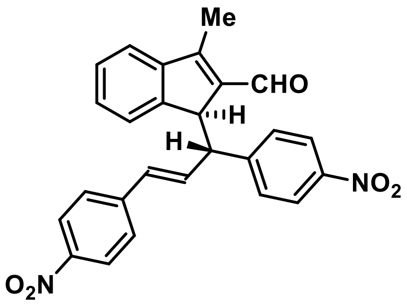

3-Methyl-1H-indene-2-carbaldehyde **1a** (31.6 mg, 0.2 mmol) and allyl acetate **2h** (34.2 mg, 0.1 mmol) were subjected to conditions B, the crude product was purified by column chromatography (silica gel, pentane/CH_2Cl_2, 1:1 to 0:10 v/v) to afford **5t** (29.7 mg, 0.067 mmol, 67% yield, d.r. (**4**:**5**) = 1:1.8, ee = 72%/80%).

Physical State: yellow oil.

R_f = 0.43 (CH_2Cl_2; 2,4-DNP).

^{1}H-NMR [$CDCl_3$, 400 MHz] δ: 10.24 (s, 1H), 8.23 (d, J = 8.6 Hz, 2H), 8.08 (d, J = 8.8 Hz, 2H), 7.65-7.41 (m, 4H), 7.32-7.27 (m, 3H), 6.64 (d, J = 7.5 Hz, 1H), 6.45 (d, J = 15.8 Hz, 1H), 5.94 (dd, J = 15.8, 9.8 Hz, 1H), 4.98-4.89 (m, 1H), 4.34 (broad s, 1H), 2.50 (d, J = 1.9 Hz, 3H).

^{13}C-NMR [$CDCl_3$, 100 MHz] δ: 187.2, 158.0, 150.2, 147.1, 147.0, 145.0, 144.5, 143.1, 140.5, 132.2, 130.3, 129.6, 129.2 (2C), 128.3, 126.9 (2C), 124.8, 124.1 (2C), 123.8 (2C), 122.4, 53.0, 48.0, 10.9.

For data corresponding to the minor diastereomer, see: **4t**

HRESI MS (*m*/*z*): $(M+H)^+$ calcd. for $C_{26}H_{21}N_2O_5$, 441.1445; found 441.1451.

UPCC: Daicel Chiralpak IB, CO_2/*i*PrOH = 99/1 to 60/40 over 4 min, 3 mL/min, 40 °C, 254 nm, for major diastereomer t_R (major) = 4.81 min; t_R (minor) = 5.43 min, e.r. = 90:10, for minor diastereomer t_R (major) = 4.91 min; t_R (minor) = 5.59 min, e.r. = 86:14.

(*S*)-1-((*S*,*E*)-1,3-bis(3-Chlorophenyl)allyl)-3-methyl-1*H*-indene-2-carbaldehyde (**4u**)

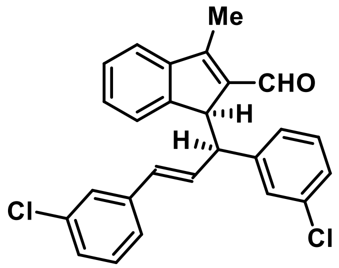

3-Methyl-1*H*-indene-2-carbaldehyde **1a** (31.2 mg, 0.2 mmol) and allyl acetate **2i** (32.1 mg, 0.1 mmol) were subjected to conditions A, the crude product was purified by column chromatography (silica gel, pentane/CH_2Cl_2, 1:1 to 0:10 v/v) to afford **4u** (33.1 mg, 0.079 mmol, 79% yield, d.r. (**4**:**5**) = 3.7:1, ee = 95%/68%).

Physical State: yellow solid.

R*f* = 0.36 (pentane:CH_2Cl_2, 4:6 v/v; 2,4-DNP).

^{1}H-NMR [$CDCl_3$, 400 MHz] δ: 10.11 (s, 1H), 7.64-7.57 (m, 1H), 7.48-7.19 (m, 7H), 7.06-7.00 (m, 1H), 6.92 (t, *J* = 7.9 Hz, 1H), 6.83 (dd, *J* = 15.9, 7.2 Hz, 1H), 6.58 (t, *J* = 1.7 Hz, 1H), 6.51 (d, *J* = 7.7 Hz, 1H), 6.44 (dd, *J* = 15.9, 1.2 Hz, 1H), 4.68-4.60 (m, 1H), 4.31 (d, *J* = 3.1 Hz, 1H), 2.31 (d, *J* = 2.0 Hz, 3H).

^{13}C-NMR [$CDCl_3$, 100 MHz] δ: 187.4, 157.5, 146.3, 144.7, 141.3, 140.6, 139.2, 134.8, 133.9, 133.5, 130.3, 130.0, 129.4, 129.0, 128.2, 128.0, 127.6, 127.0, 126.4, 126.3, 125.2, 124.7, 122.2, 53.2, 49.4, 10.6.

For data corresponding to the minor diastereomer, see: **5u**

HRESI MS (*m/z*): $(M+H)^+$ calcd. for $C_{26}H_{21}Cl_2O$, 419.0964; found 419.0964.

UPCC: Daicel Chiralpak ID, CO_2/*i*PrOH = 99/1 to 60/40 over 6.5 min, 2 mL/min, 40 °C, 309 nm, for major diastereomer t_R (major) = 5.69min; t_R (minor) = 6.64 min, e.r. = 98:2, for minor diastereomer t_R (major) = 5.56 min; t_R (minor) = 5.96 min, e.r. = 84:16.

(*S*)-1-((*R*,*E*)-1,3-bis(3-Chlorophenyl)allyl)-3-methyl-1*H*-indene-2-carbaldehyde (**5u**)

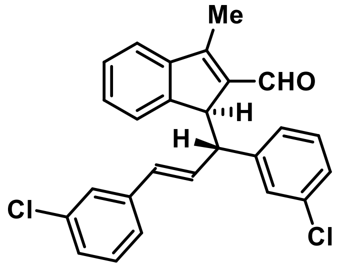

3-Methyl-1*H*-indene-2-carbaldehyde **1a** (31.2 mg, 0.2 mmol) and allyl acetate **2i** (32.1 mg, 0.1 mmol) were subjected to conditions B, the crude product was purified by column chromatography (silica gel, pentane/CH_2Cl_2, 1:1 to 0:10 v/v) to afford **5u** (32.6 mg, 0.078 mmol, 78% yield, d.r. (**4**:**5**) = 1:2.4, ee = 82%/86%).

Physical State: yellow solid.

R*f* = 0.36 (pentane:CH_2Cl_2, 4:6 v/v; 2,4-DNP).

^{1}H-NMR [$CDCl_3$, 400 MHz] δ: 10.15 (s, 1H), 7.51-7.46 (m, 1H), 7.40-7.13 (m, 6H), 7.09-7.03 (m, 2H), 6.98 (d, *J* = 5.6 Hz, 1H), 6.91-6.86 (m, 1H), 6.52 (d, *J* = 7.6 Hz, 1H), 6.21 (d, *J* = 15.7 Hz, 1H), 5.51 (dd, *J* = 15.7, 10.0 Hz, 1H), 4.72 (dd, *J* = 10.0, 3.9 Hz, 1H), 4.23 (s, 1H), 2.42 (d, *J* = 1.9 Hz, 3H).

^{13}C-NMR [$CDCl_3$, 100 MHz] δ: 187.2, 157.4, 145.5, 145.3, 144.6, 141.1, 138.9, 134.5, 134.5, 132.4, 129.8, 129.8, 129.2, 128.4, 127.9, 127.5, 127.0, 126.9, 126.5, 126.2, 125.2, 124.7, 122.1, 53.2, 47.3, 10.9.

For data corresponding to the minor diastereomer, see: **4u**

HRESI MS (*m/z*): $(M+H)^+$ calcd. for $C_{26}H_{21}Cl_2O$, 419.0964; found 419.0964.

UPCC: Daicel Chiralpak ID, CO_2/*i*PrOH = 99/1 to 60/40 over 6.5 min, 2 mL/min, 40 °C, 309 nm, for major diastereomer t_R (major) = 5.54 min; t_R (minor) = 5.95 min, e.r. = 93:7, for minor diastereomer t_R (major) = 5.69 min; t_R (minor) = 6.63 min, e.r. = 91:9.

(*S*)-1-((*S*,*E*)-1,3-bis(3-Bromophenyl)allyl)-3-methyl-1*H*-indene-2-carbaldehyde (**4v**)

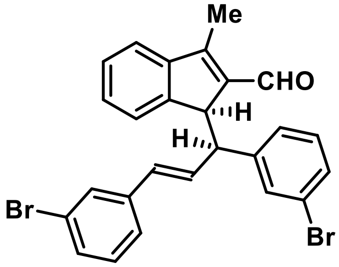

3-Methyl-1*H*-indene-2-carbaldehyde **1a** (31.6 mg, 0.2 mmol) and allyl acetate **2j** (41.0 mg, 0.1 mmol) were subjected to conditions A, the crude product was purified by column chromatography (silica gel, pentane/CH_2Cl_2, 1:1 to 0:10 v/v) to afford **4v** (43.2 mg, 0.085 mmol, 85% yield, d.r. (**4**:**5**) = 3.4:1, ee = 95%/64%).

Physical State: yellow solid.

R_f = 0.40 (pentane:CH_2Cl_2, 4:6 v/v; 2,4-DNP).

^{1}H-NMR [$CDCl_3$, 400 MHz] δ: 10.10 (s, 1H), 7.63-7.52 (m, 2H), 7.50-7.16 (m, 7H), 6.91-6.77 (m, 2H), 6.75-6.71 (m, 1H), 6.55 (d, *J* = 7.8 Hz, 1H), 6.43 (d, *J* = 15.9 Hz, 1H), 4.62 (t, *J* = 5.6 Hz, 1H), 4.31 (d, *J* = 3.0 Hz, 1H), 2.32 (d, *J* = 1.9 Hz, 3H).

^{13}C-NMR [$CDCl_3$, 100 MHz] δ: 187.4, 157.5, 146.2, 144.7, 141.5, 140.6, 139.4, 133.9, 131.2, 130.5, 130.3, 130.2, 129.9, 129.4, 129.3, 129.2, 128.0, 126.8, 125.2, 125.1, 123.0, 122.2, 121.8, 53.2, 49.4, 10.7.

For data corresponding to the minor diastereomer, see: **5v**

HRESI MS (*m/z*): $(M+H)^+$ calcd. for $C_{26}H_{21}Br_2O$, 506.9954; found 506.9953.

UPCC: Daicel Chiralpak ID, CO_2/*i*PrOH = 99/1 to 60/40 over 4 min, 3 mL/min, 40 °C, 309 nm, for major diastereomer t_R (major) = 6.73 min; t_R (minor) = 7.77 min, e.r. = 98:2, for minor diastereomer t_R (major) = 6.53 min; t_R (minor) = 7.00 min, e.r. = 82:18.

(*S*)-1-((*R*,*E*)-1,3-bis(3-Bromophenyl)allyl)-3-methyl-1*H*-indene-2-carbaldehyde (**5v**)

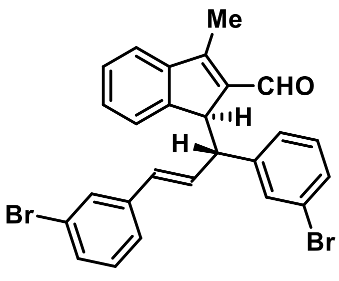

3-Methyl-1*H*-indene-2-carbaldehyde **1a** (31.6 mg, 0.2 mmol) and allyl acetate **2j** (41.0 mg, 0.1 mmol) were subjected to conditions B, the crude product was purified by column chromatography (silica gel, pentane/CH_2Cl_2, 1:1 to 0:10 v/v) to afford **5v** (40.9 mg, 0.081 mmol, 81% yield, d.r. (**4**:**5**) = 1:2.6, ee = 81%/88%).

Physical State: yellow solid.

R_f = 0.40 (pentane:CH_2Cl_2, 4:6 v/v; 2,4-DNP).

^{1}H-NMR [$CDCl_3$, 400 MHz] δ: 10.22 (s, 1H), 7.63-7.52 (m, 2H), 7.50-7.16 (m, 7H), 7.07 (t, *J* = 7.8 Hz, 1H), 7.00 (d, *J* = 7.7 Hz, 1H), 6.60 (d, *J* = 7.6 Hz, 1H), 6.27 (d, *J* = 15.7 Hz, 1H), 5.58 (dd,

J = 15.7, 10.0 Hz, 1H), 4.80 (dd, *J* = 10.0, 3.9 Hz, 1H), 4.31 (d, *J* = 3.0 Hz, 1H), 2.50 (d, *J* = 1.9 Hz, 3H).

^{13}C-NMR [$CDCl_3$, 100 MHz] δ: 187.2, 157.4, 145.5, 145.5, 144.6, 141.1, 139.2, 132.3, 131.4, 130.4, 130.1 (2C), 129.9, 129.2, 129.2, 127.9, 127.0, 126.9, 125.2, 125.1, 122.8, 122.8, 122.1, 53.2, 47.3, 10.8.

For data corresponding to the minor diastereomer, see: **4v**

HRESI MS (*m/z*): $(M+H)^+$ calcd. for $C_{26}H_{21}Br_2O$, 506.9954; found 506.9947.

UPCC: Daicel Chiralpak ID, CO_2/*i*PrOH = 99/1 to 60/40 over 4 min, 3 mL/min, 40 °C, 309 nm, for major diastereomer t_R (major) = 6.27 min; t_R (minor) = 6.70 min, e.r. = 94:6, for minor diastereomer t_R (major) = 6.45 min; t_R (minor) = 7.47 min, e.r. = 90:10.

X-Ray crystal structure

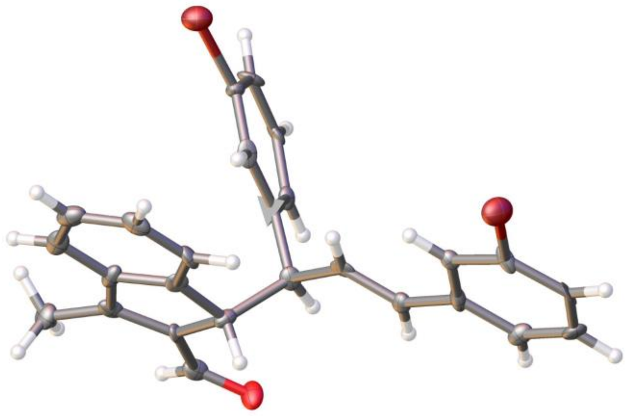

Figure S1. Crystal data for **4v**: $C_{26}H_{20}Br_2O$, M = 508.24, monoclinic, Space group P 21 (no. 4), a = 8.3719(6) Å, b = 14.0717(9) Å, c = 8.8443(6) Å, β = 95.022(2)°, Flack parameter = 0.04, V = 1037.92(12) Å3, T = 100 K, Z = 2, dc = 1.626 g cm-3, μ(Mo Kα, λ = 0.71073 Å) = 3.92 mm-1, 24480 reflections collected, 4385 unique [Rint = 0.0437], which were used in all calculations. Refinement on F2, final R(F) = 0.1219, Rw(F2) = 0.2369. CCDC deposition number 1976145.

Table S4. Pertinent crystallographic parameters of **4v**.

Item	Value
Molecular formula	$C_{26}H_{20}Br_2O$
Formula weight	508.24
Crystal system	monoclinic
Space Group	$P\ 2_1$
a (Å)	8.3719
b (Å)	14.0717
c (Å)	8.8443
α (°)	90
β (°)	95.022
γ (°)	90
Volume (Å^3)	1037.92
Z	2
T (K)	100
ρ (g cm^{-1})	1.626
λ (Å)	0.71073
μ (mm^{-1})	3.92
# measured refl	24480
# unique refl	4385
R_{int}	0.0437
# parameters	251
R(F^2), all refl	0.1219
R_w(F^2), all refl	0.2369
Goodness of fit	1.294

ECD spectrum of 5v

The mixture of stereoisomers obtained from the reaction of **1a** and **2j** (Scheme S1) was separated by chiral SCF chromatography.[5] The major enantiomer of **5v** was analyzed *via* electronic circular dichroism to ascertain its stereochemistry. The calculated ECD spectrum of **4v** (structure confirmed by X-ray crystallography, Figure S1) is shown in Figure S2. The calculated ECD spectrum of one enantiomer of **5v**, wherein the stereocenter on the indene is inverted, is shown in Figure S3. The experimental ECD spectrum (Figure S6) is opposite to Figure S3, therefore it is the allylic stereocentre which is inverted.

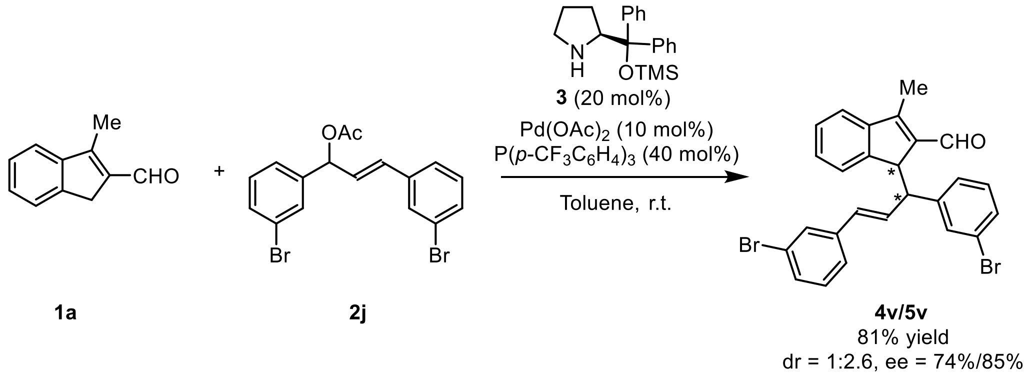

Scheme S1. The reaction of indene-2-carbaldehyde **1a** with bis(3-bromophenyl)allyl acetate **2j** catalyzed by trimethylsilyl diphenylprolinol **3a** and $Pd(OAc)_2$ affording **4v**/**5v** (d.r. = 2.6:1).

First, conformational analysis of each structure was performed using metadynamic sampling in extended tight binding Conformer–Rotamer Ensemble Sampling Tool (xtb CREST) program package. All further DFT calculations were computed using the Gaussian 16 package. Lowest energy conformers of up to 5 kcal/mol (21 conformers each of **4v** and **5v**) were then optimized with the M06-2X method and 6-31G(d,p) basis set in the gas phase and re-optimized with vibrational analysis using the same method but larger basis set of 6-311+G(2d,p) in CH_2Cl_2(SMD solvation model). The free energies were corrected using Truhlar's quasiharmonic approximation for frequencies below 100 cm^{-1}.

TD-DFT excited-state calculation of each conformer (with a population of at least 2% based on free energies) was performed using the same method with the large basis set and solvation in CH_2Cl_2 based on the optimized structure. The ECD spectra were then generated based on rotatory strengths resulting from the TD-DFT calculation using GaussView 6.0.16. Boltzmann averaging of all spectra led to the ECD spectra shown in Figure S2 and S3.

---

[5] The mixture of stereoisomers was separated by Lotus Separations LLC at Princeton University.

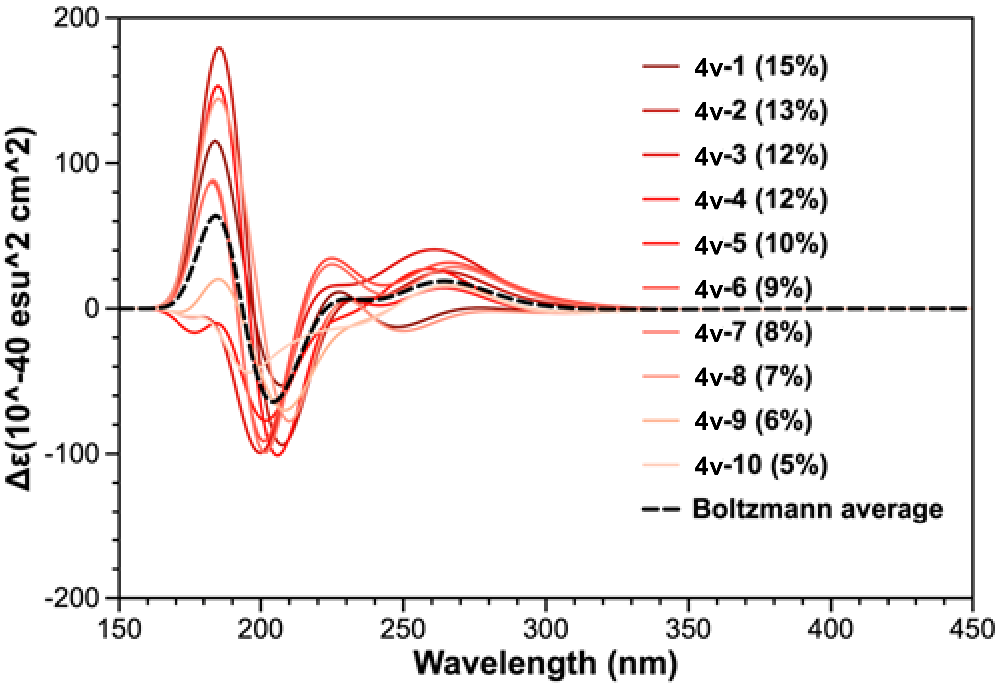

Figure S2. Calculated ECD spectra of all conformers and Boltzmann average of structure **4v** using M06-2X/6-311+G(2d,p) method in SMD solvation model (CH_2Cl_2).

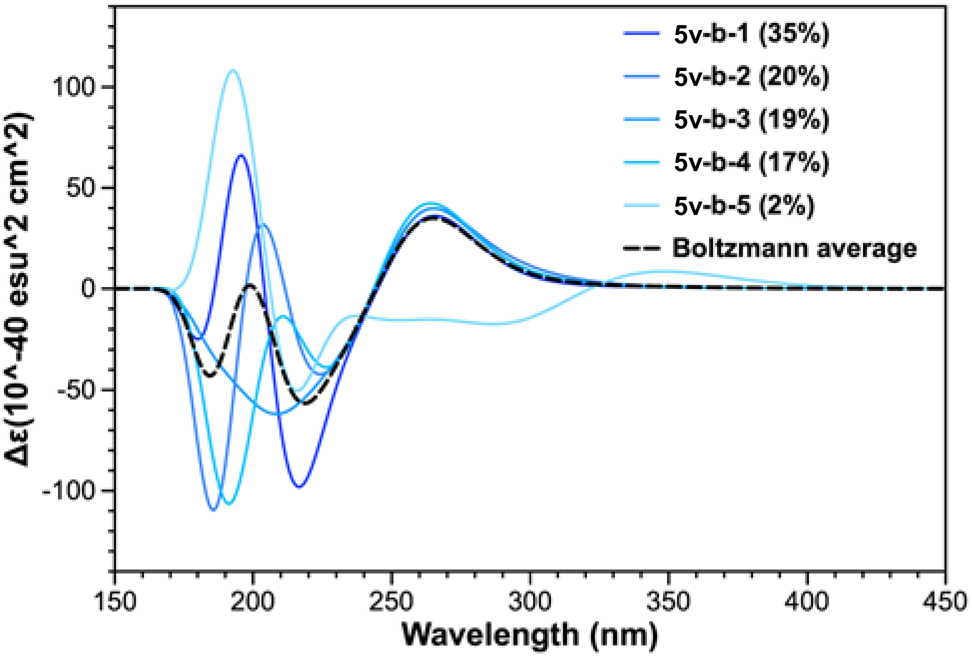

Figure S3. Calculated ECD spectra of all conformers and Boltzmann average of structure **5v** using M06-2X/6-311+G(2d,p) method in SMD solvation model (CH_2Cl_2).

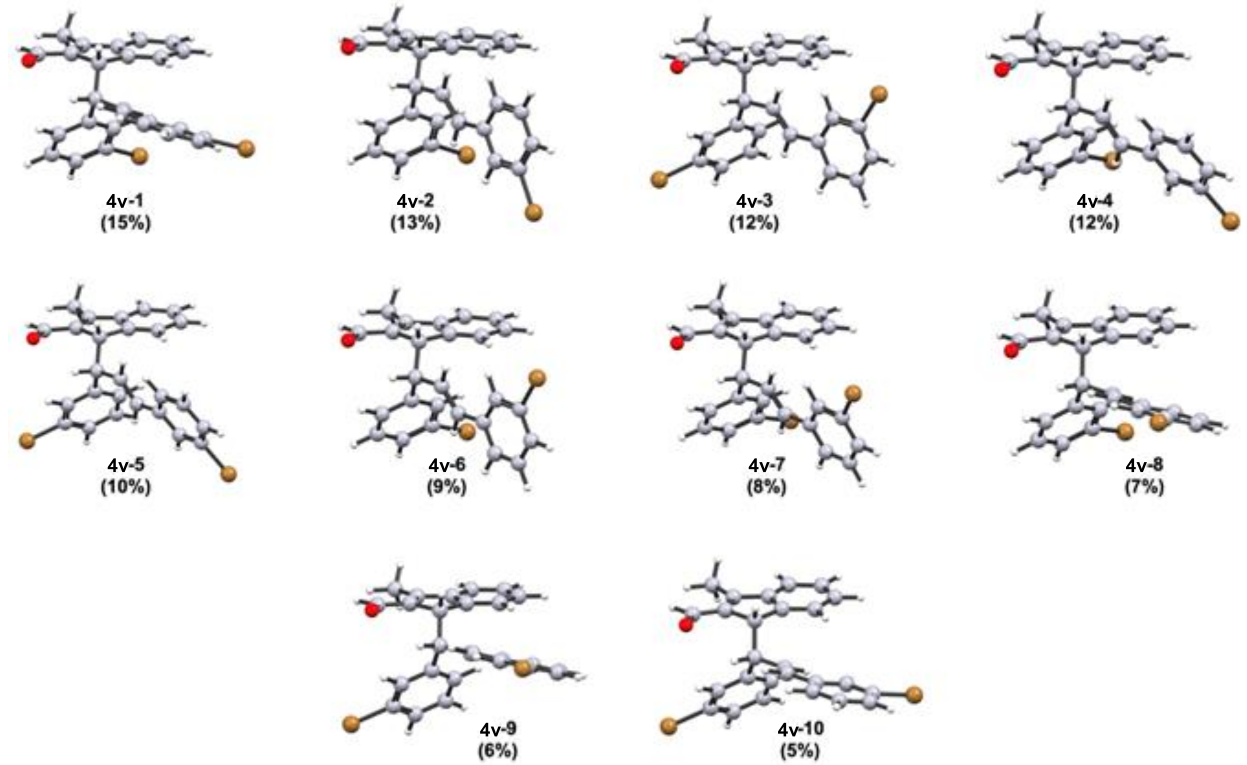

Figure S4. Lowest energy conformers of **4v** optimized using M06-2X/6-311+G(2d,p) method in SMD solvation model (CH_2Cl_2) and their corresponding populations.

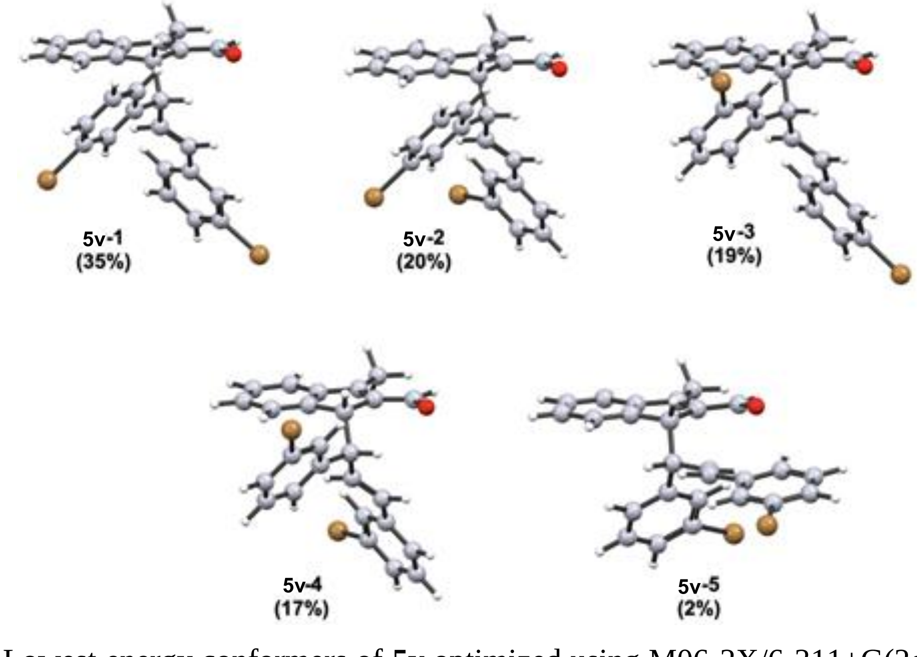

Figure S5. Lowest energy conformers of **5v** optimized using M06-2X/6-311+G(2d,p) method in SMD solvation model (CH_2Cl_2) and their corresponding populations.

Table S5. The calculated electronic and thermodynamic properties and Boltzmann distribution of all conformers of **5v** at M06-2X/6-311+G(2d,p) method in SMD solvation model (CH_2Cl_2). Temperature used was 298 K.

Conformer	Etot (kcal/mol)	G (kJ/mol)	ΔG	-ΔG/RT	exp^(-ΔG/RT)	mole fraction	pop (%)
1	-6226.254	-16346164.7	0.0	0.00	1.00	0.3496	35.0
2	-6226.255	-16346163.4	1.3	-0.54	0.58	0.2041	20.4
3	-6226.254	-16346163.2	1.5	-0.61	0.54	0.1903	19.0
4	-6226.255	-16346162.9	1.8	-0.73	0.48	0.1680	16.8
5	-6226.252	-16346158.1	6.6	-2.66	0.07	0.0244	2.4

Table S6. The calculated electronic and thermodynamic properties and Boltzmann distribution of all conformers of **4v** at M06-2X/6-311+G(2d,p) method in SMD solvation model (CH_2Cl_2). Temperature used was 298 K.

Conformer	Etot (kcal/mol)	G (kJ/mol)	ΔG	-ΔG/RT	exp^(-ΔG/RT)	mole fraction	pop (%)
1	-6226.2547	-16346163.9	0.0	0.00	1.00	0.1479	14.8
2	-6226.2550	-16346163.6	0.2	-0.10	0.91	0.1343	13.4
3	-6226.2546	-16346163.4	0.5	-0.19	0.83	0.1221	12.2
4	-6226.2549	-16346163.2	0.6	-0.25	0.78	0.1149	11.5
5	-6226.2544	-16346163.0	0.9	-0.36	0.70	0.1032	10.3
6	-6226.2551	-16346162.7	1.1	-0.45	0.64	0.0942	9.4
7	-6226.2551	-16346162.2	1.6	-0.66	0.52	0.0764	7.6
8	-6226.2547	-16346162.0	1.9	-0.76	0.47	0.0694	6.9
9	-6226.2543	-16346161.5	2.4	-0.97	0.38	0.0560	5.6
10	-6226.2544	-16346161.2	2.6	-1.06	0.35	0.0511	5.1

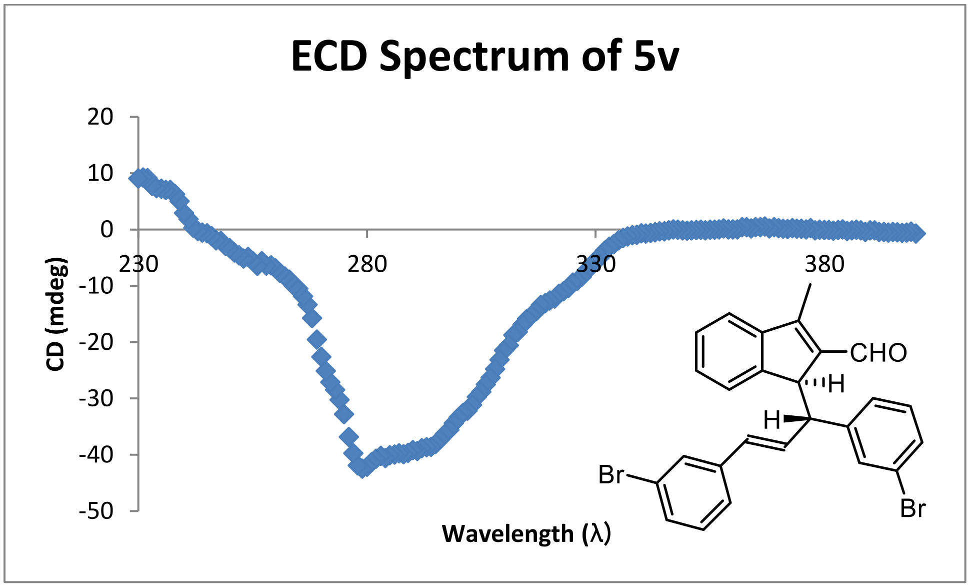

Figure S6. Experimental ECD spectrum structure **5v** (CH_2Cl_2). The spectrum was recorded between 230-400 nm as the solvent obscures measurement below 230 nm.

Part 2: NMR Spectra

Table of Contents

Compound **1b** ^{1}H NMR S54
Compound **1b** ^{13}C NMR S54
Compound **1c** ^{1}H NMR S55
Compound **1c** ^{13}C NMR S55
Compound **1d** ^{1}H NMR S56
Compound **1d** ^{13}C NMR S56
Compound **1e** ^{1}H NMR S57
Compound **1e** ^{13}C NMR S57
Compound **1e** ^{19}F NMR S58
Compound **1f** ^{1}H NMR S59
Compound **1f** ^{13}C NMR S59
Compound **1g** 1H NMR S60
Compound **1g** ^{13}C NMR S60
Compound **1i** ^{1}H NMR S61
Compound **1i** ^{13}C NMR S61
Compound **1m** ^{1}H NMR S62
Compound **1m** ^{13}C NMR S62
Compound **2c** ^{1}H NMR S63
Compound **2c** ^{13}C NMR S63
Compound **2d** ^{1}H NMR S64
Compound **2d** ^{13}C NMR S64
Compound **2d** ^{19}F NMR S66
Compound **2e** ^{1}H NMR S67
Compound **2e** ^{13}C NMR S67
Compound **2g** ^{1}H NMR S68
Compound **2g** ^{13}C NMR S68
Compound **2g** ^{19}F NMR S69
Compound **2i** ^{1}H NMR S70
Compound **2i** ^{13}C NMR S70
Compound **2j** ^{1}H NMR S71
Compound **2j** ^{13}C NMR S71
Compound **4a** ^{1}H NMR S72
Compound **4a** ^{13}C NMR S72
Compound **5a** ^{1}H NMR S73
Compound **5a** ^{13}C NMR S73
Compound **4b** ^{1}H NMR S74
Compound **4b** ^{13}C NMR S74
Compound **5b** ^{1}H NMR S75

Compound **5b** ^{13}C NMR S75
Compound **4c** ^{1}H NMR S76
Compound **4c** ^{13}C NMR S76
Compound **5c** ^{1}H NMR S77
Compound **5c** ^{13}C NMR S77
Compound **4d** ^{1}H NMR S78
Compound **4d** ^{13}C NMR S78
Compound **5d** ^{1}H NMR S79
Compound **5d** ^{13}C NMR S79
Compound **4e** ^{1}H NMR S80
Compound **4e** ^{13}C NMR S80
Compound **4e** ^{19}F NMR S81
Compound **5e** ^{1}H NMR S82
Compound **5e** ^{13}C NMR S82
Compound **5e** ^{19}F NMR S83
Compound **4f** ^{1}H NMR S84
Compound **4f** ^{13}C NMR S84
Compound **5f** ^{1}H NMR S85
Compound **5f** ^{13}C NMR S85
Compound **4g** ^{1}H NMR S86
Compound **4g** ^{13}C NMR S86
Compound **5g** ^{1}H NMR S87
Compound **5g** ^{13}C NMR S87
Compound **4h** ^{1}H NMR S88
Compound **4h** ^{13}C NMR S88
Compound **5h** ^{1}H NMR S89
Compound **5h** ^{13}C NMR S89
Compound **4i** ^{1}H NMR S90
Compound **4i** ^{13}C NMR S90
Compound **5i** ^{1}H NMR S91
Compound **5i** ^{13}C NMR S91
Compound **4j** ^{1}H NMR S92
Compound **4j** ^{13}C NMR S92
Compound **4j** ^{19}F NMR S93
Compound **5j** ^{1}H NMR S94
Compound **5j** ^{13}C NMR S94
Compound **5j** ^{19}F NMR S95
Compound **4k** ^{1}H NMR S96
Compound **4k** ^{13}C NMR S96
Compound **5k** ^{1}H NMR S97

Compound **5k** ^{13}C NMR S97
Compound **4l** ^{1}H NMR S98
Compound **4l** ^{13}C NMR S98
Compound **5l** ^{1}H NMR S99
Compound **4l** ^{13}C NMR S99
Compound **4m** ^{1}H NMR S100
Compound **4m** ^{13}C NMR S100
Compound **5m** ^{1}H NMR S101
Compound **5m** ^{13}C NMR S101
Compound **4n** ^{1}H NMR S102
Compound **4n** ^{13}C NMR S102
Compound **5n** ^{1}H NMR S103
Compound **5n** ^{13}C NMR S103
Compound **4o** ^{1}H NMR S104
Compound **4o** ^{13}C NMR S104
Compound **5o** ^{1}H NMR S105
Compound **5o** ^{13}C NMR S105
Compound **4p** ^{1}H NMR S106
Compound **4p** ^{13}C NMR S106
Compound **4p** ^{19}F NMR S107
Compound **5p** ^{1}H NMR S108
Compound **5p** ^{13}C NMR S108
Compound **5p** ^{19}F NMR S109
Compound **4q** ^{1}H NMR S110
Compound **4q** ^{13}C NMR S110
Compound **5q** ^{1}H NMR S111
Compound **5q** ^{13}C NMR S111
Compound **4r** ^{1}H NMR S112
Compound **4r** ^{13}C NMR S112
Compound **5r** ^{1}H NMR S113
Compound **5r** ^{13}C NMR S113
Compound **4s** ^{1}H NMR S114
Compound **4s** ^{13}C NMR S114
Compound **4s** ^{19}F NMR S115
Compound **5s** ^{1}H NMR S116
Compound **5s** ^{13}C NMR S116
Compound **5s** ^{19}F NMR S117
Compound **4t** ^{1}H NMR S118
Compound **4t** ^{13}C NMR S118
Compound **5t** ^{1}H NMR S119

Compound **5t** ^{13}C NMR S119
Compound **4u** ^{1}H NMR S120
Compound **4u** ^{13}C NMR S120
Compound **5u** ^{1}H NMR S121
Compound **5u** ^{13}C NMR S121
Compound **4v** ^{1}H NMR S122
Compound **4v** ^{13}C NMR S122
Compound **5v** ^{1}H NMR S123
Compound **5v** ^{13}C NMR S123

Compound **1b** ^{1}H NMR

(400 MHz, $CDCl_3$)

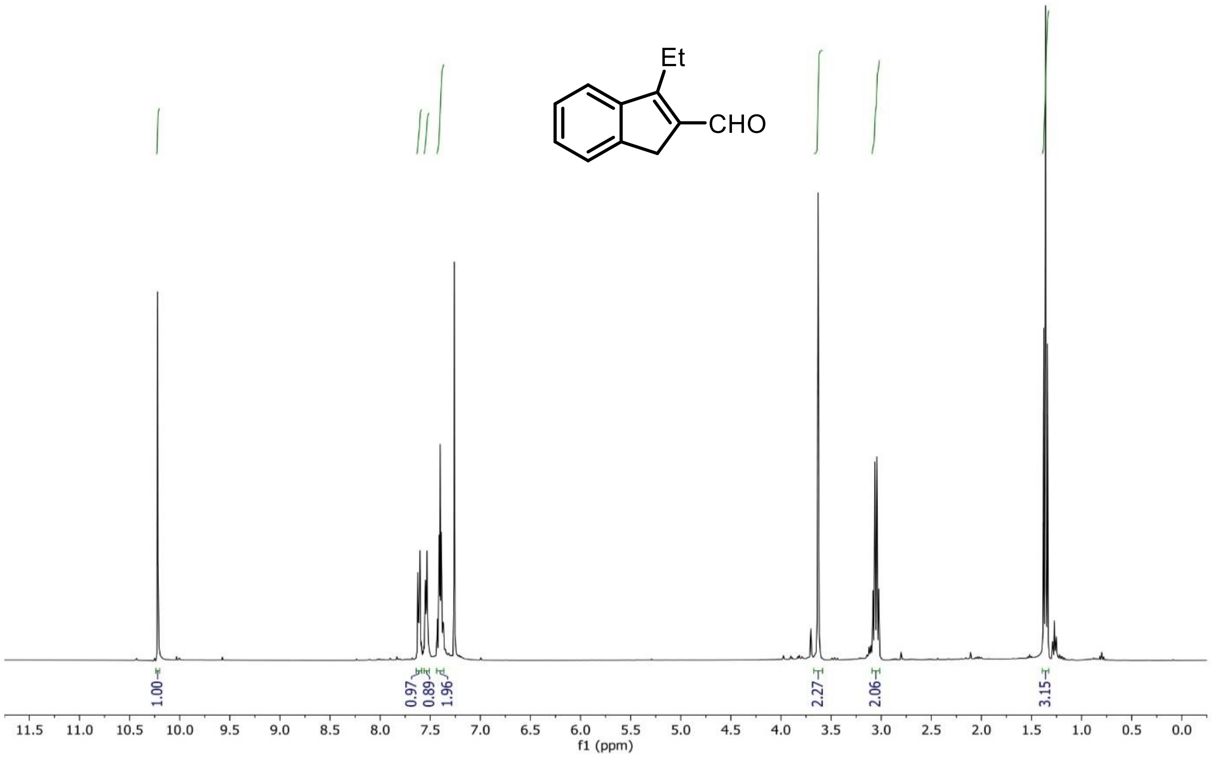

Compound **1b** ^{13}C NMR

(100 MHz, $CDCl_3$)

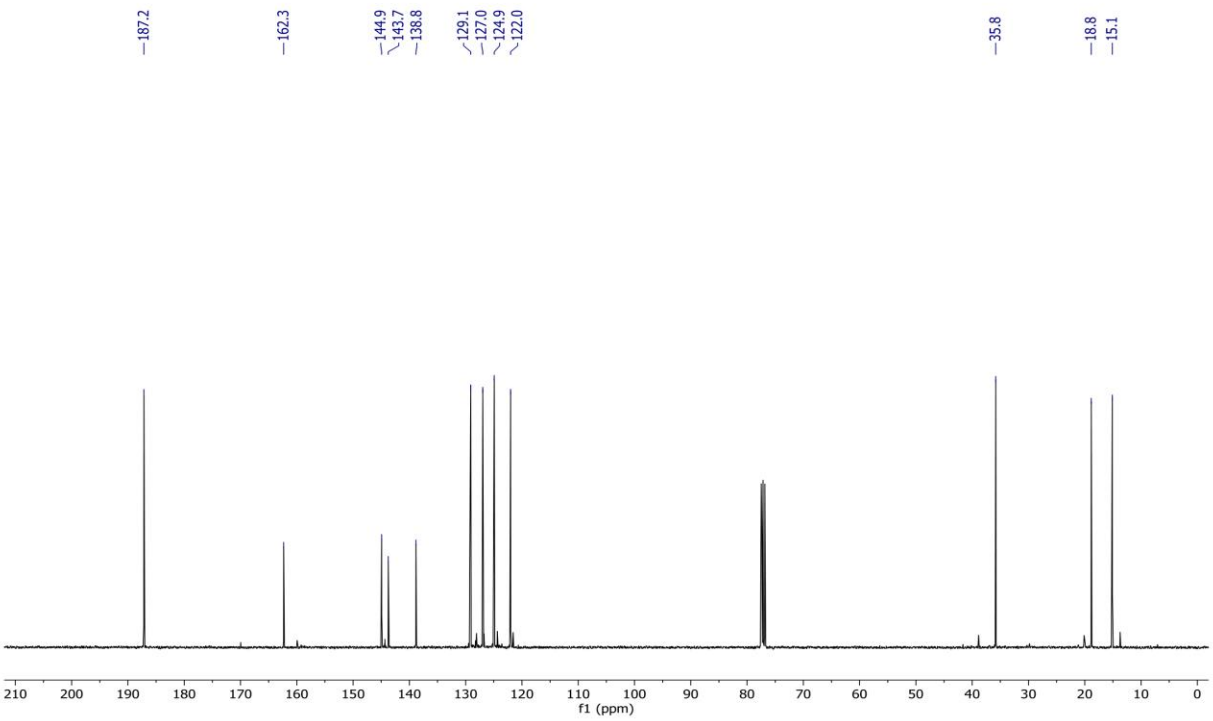

Compound **1c** ^{1}H NMR

(400 MHz, $CDCl_3$)

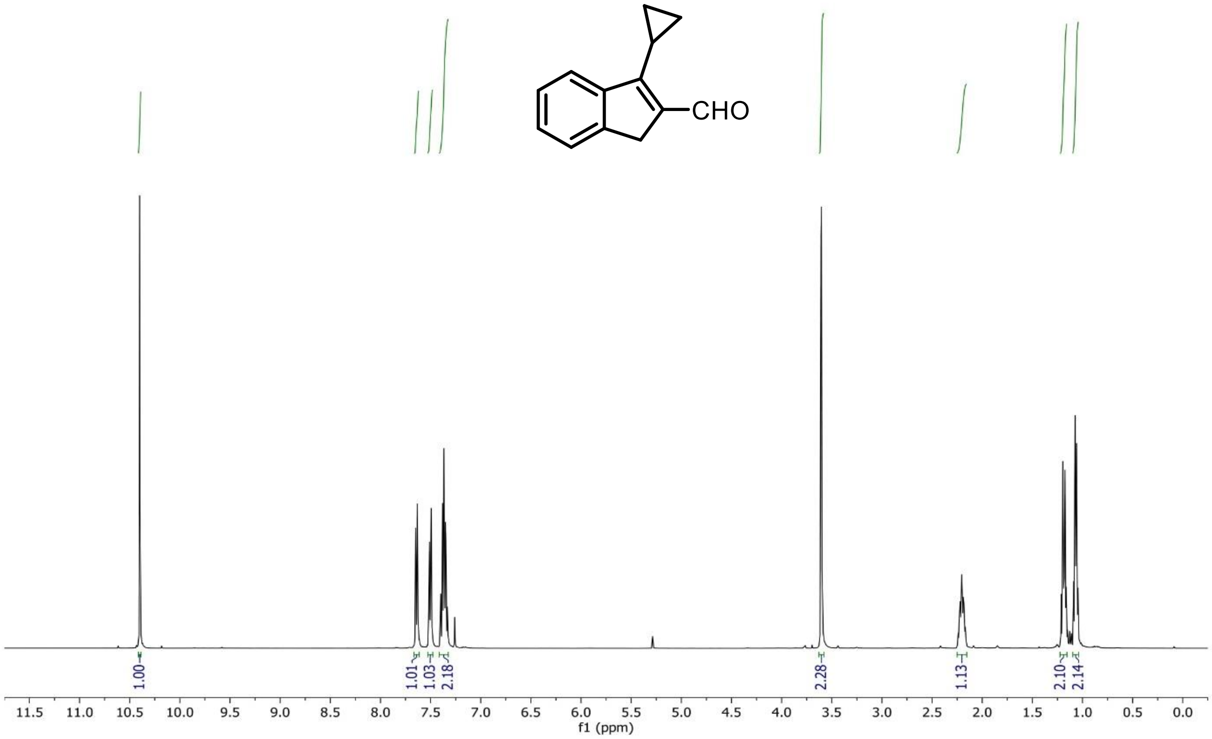

Compound **1c** ^{13}C NMR

(100 MHz, $CDCl_3$)

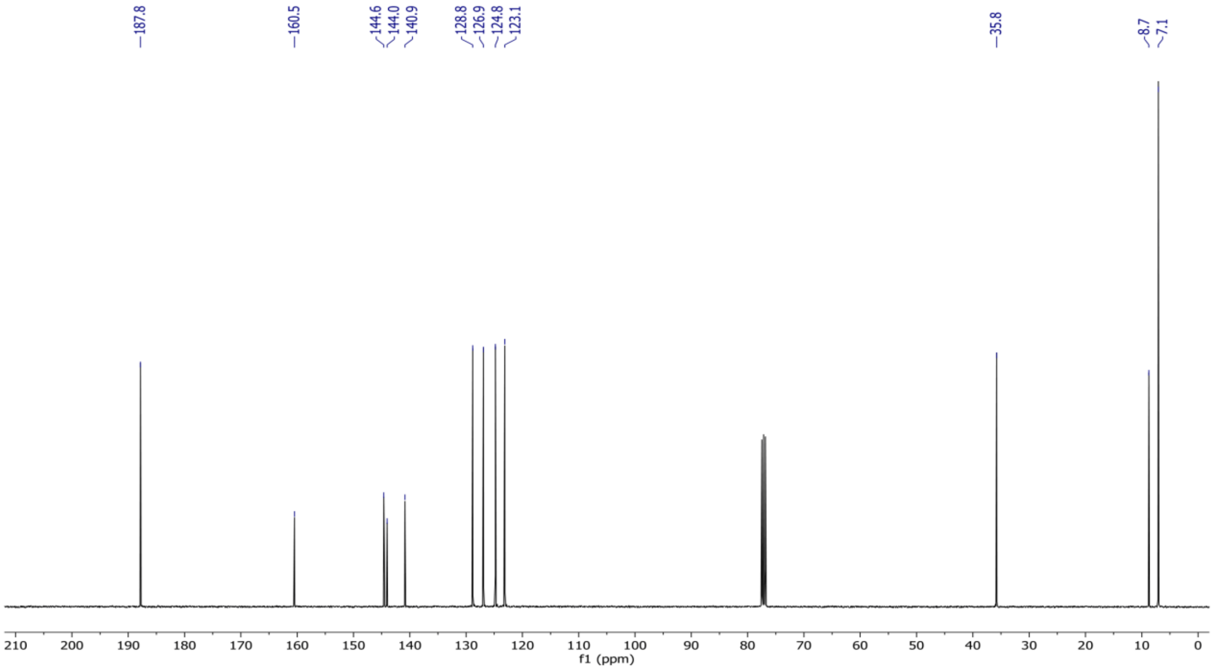

Compound **1d** ^{1}H NMR

(400 MHz, $CDCl_3$)

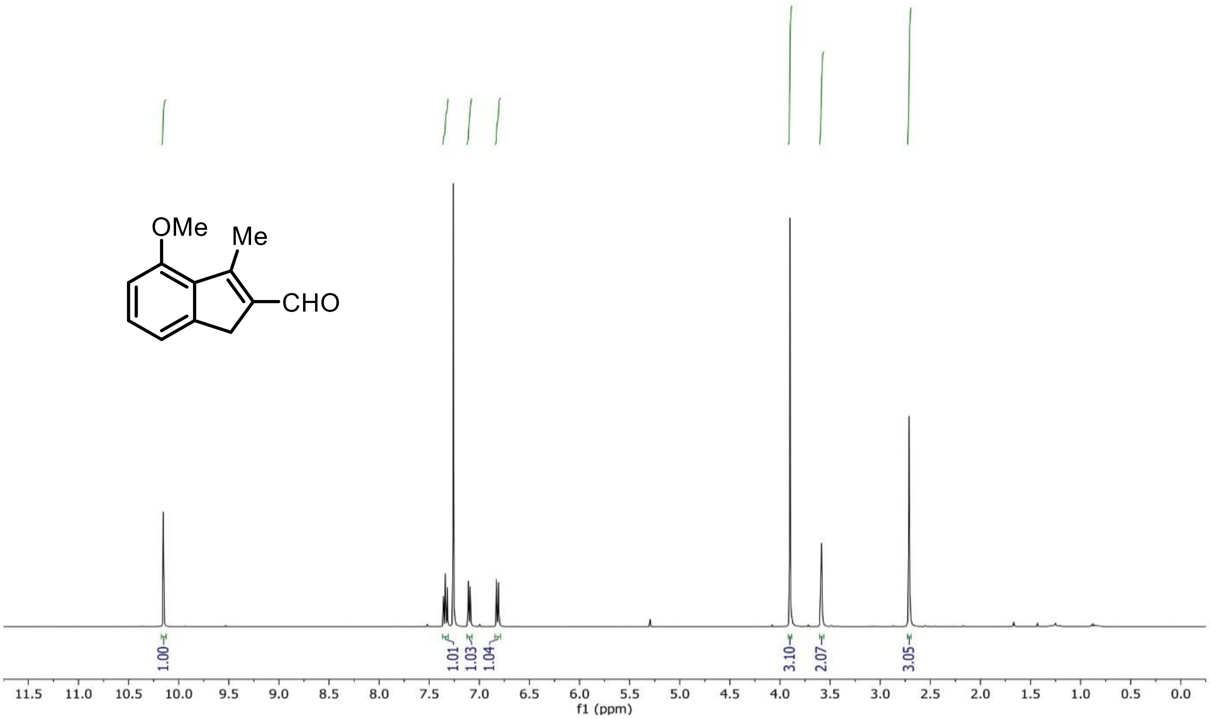

Compound **1d** ^{13}C NMR

(100 MHz, $CDCl_3$)

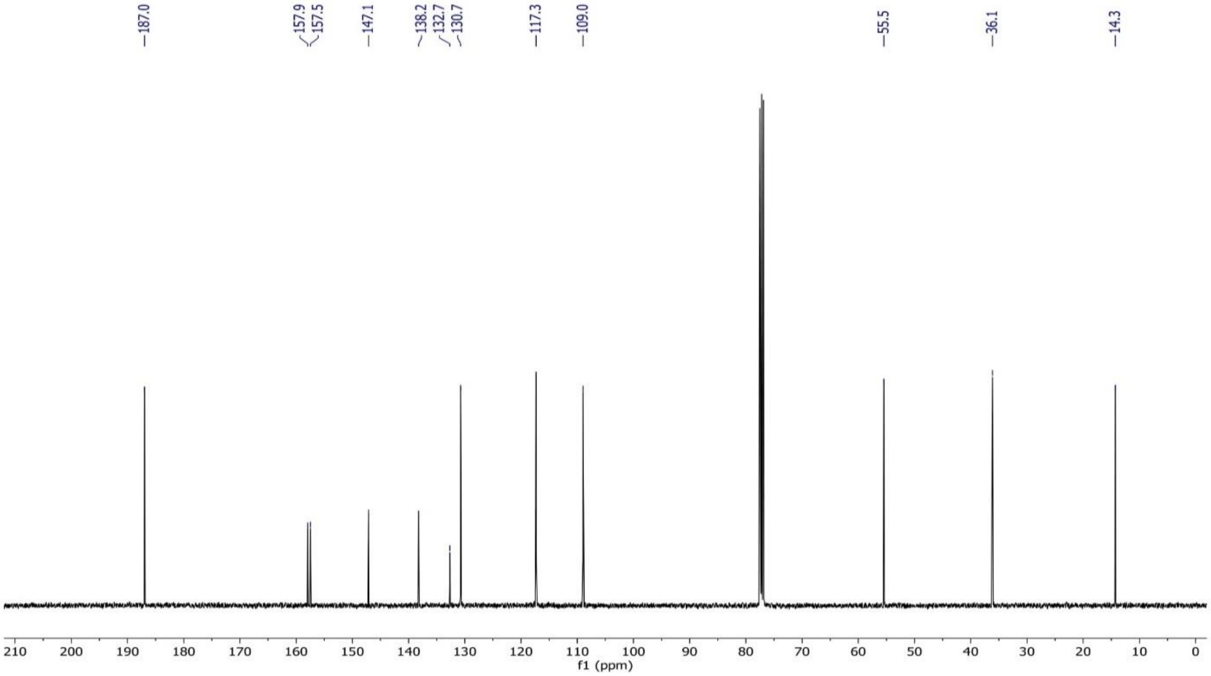

Compound **1e** ^{1}H NMR

(400 MHz, $CDCl_3$)

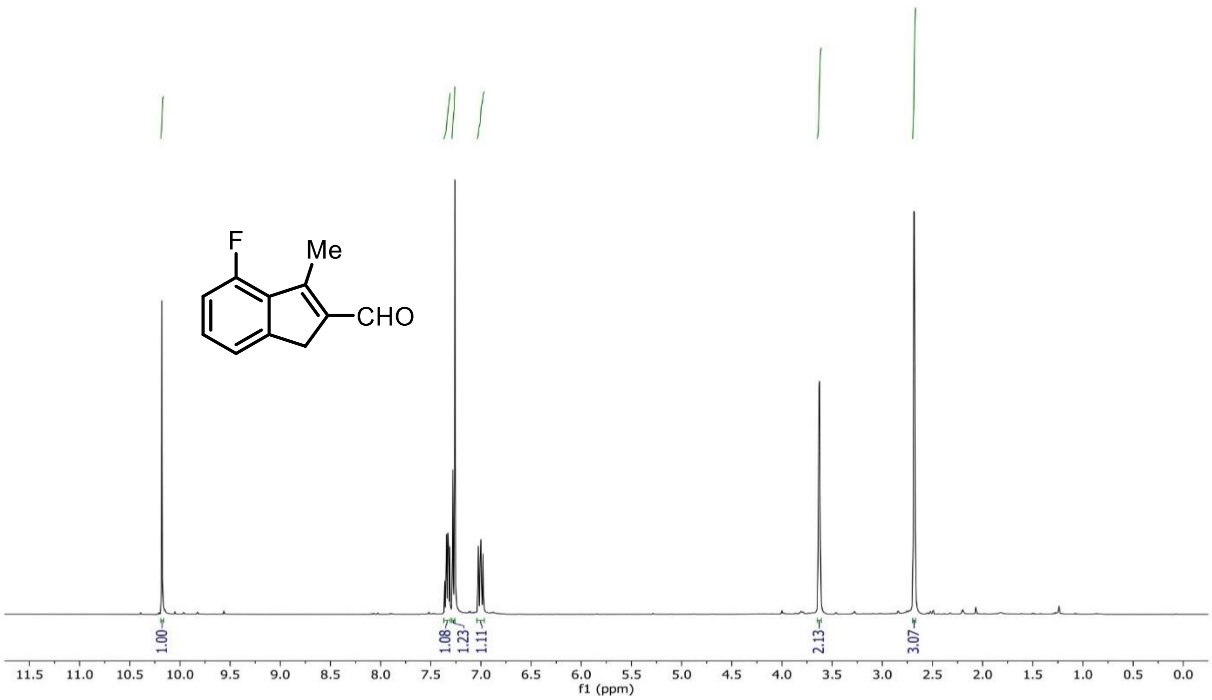

Compound **1e** ^{13}C NMR

(100 MHz, $CDCl_3$)

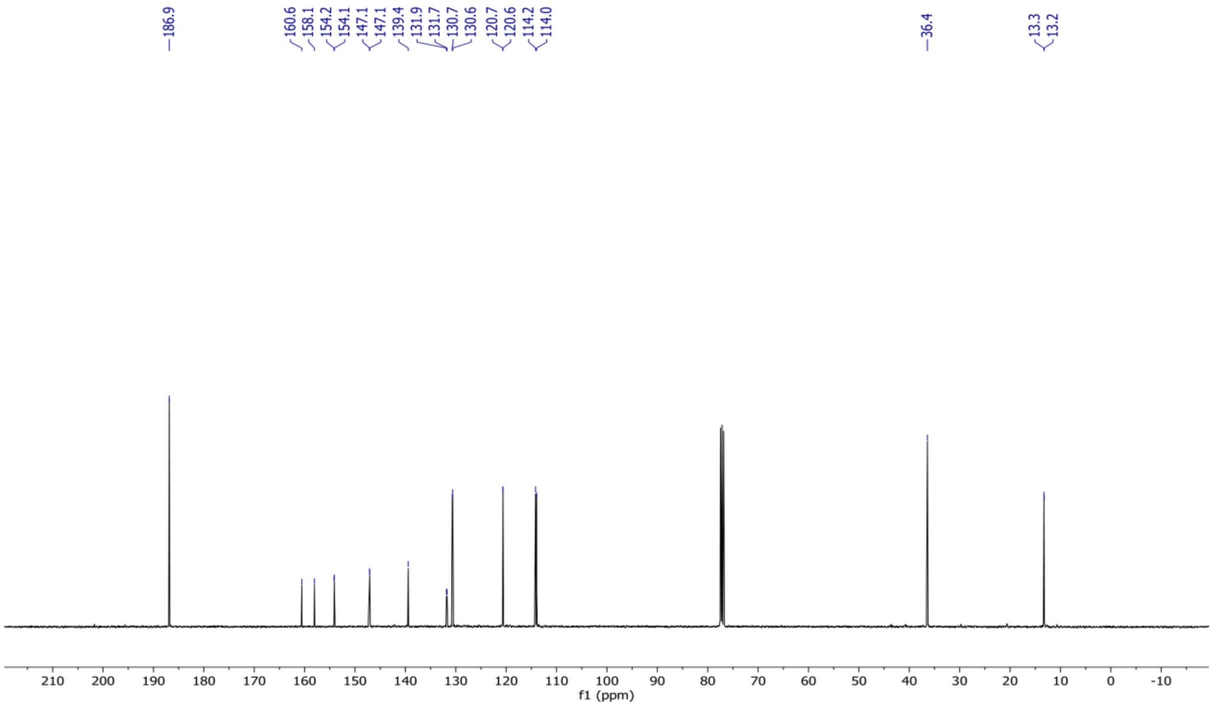

Compound **1e** ^{19}F NMR

(375 MHz, $CDCl_3$)

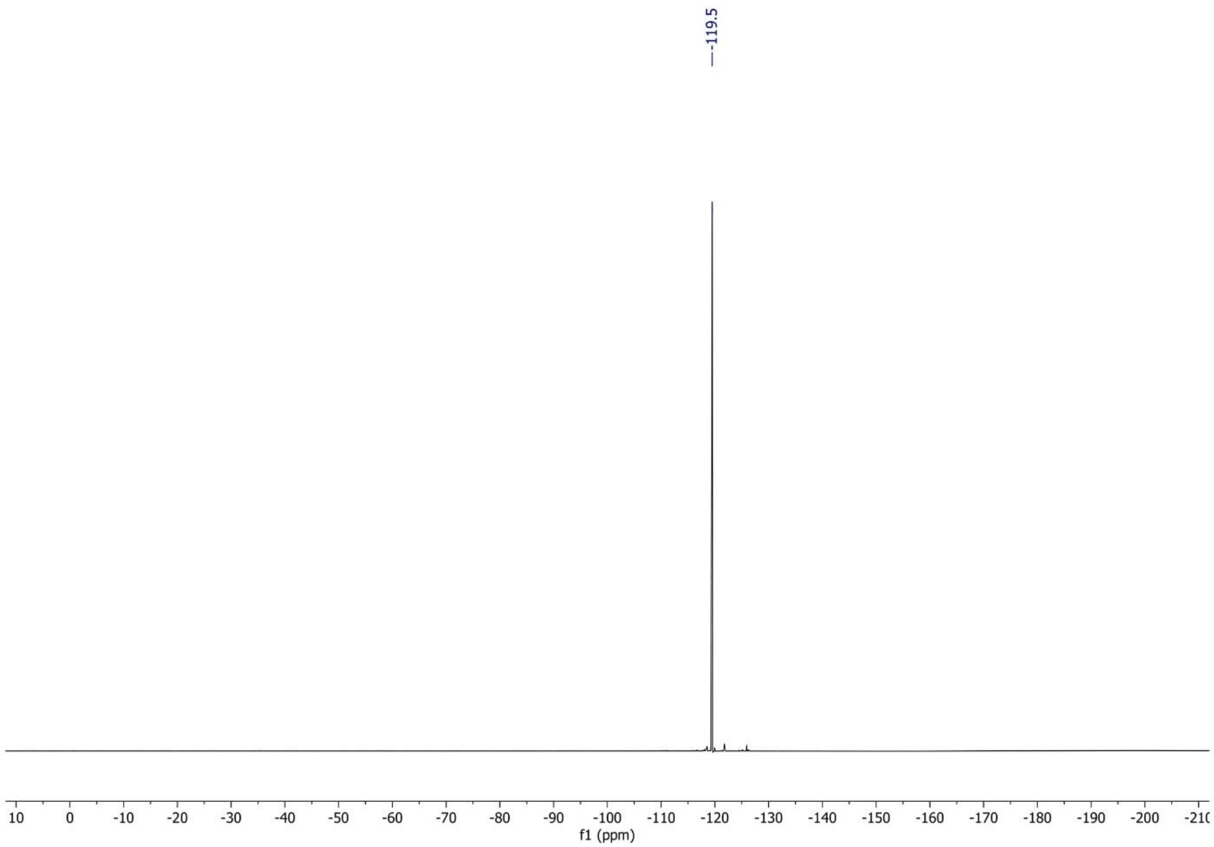

Compound **1f** ^{1}H NMR

(400 MHz, $CDCl_3$)

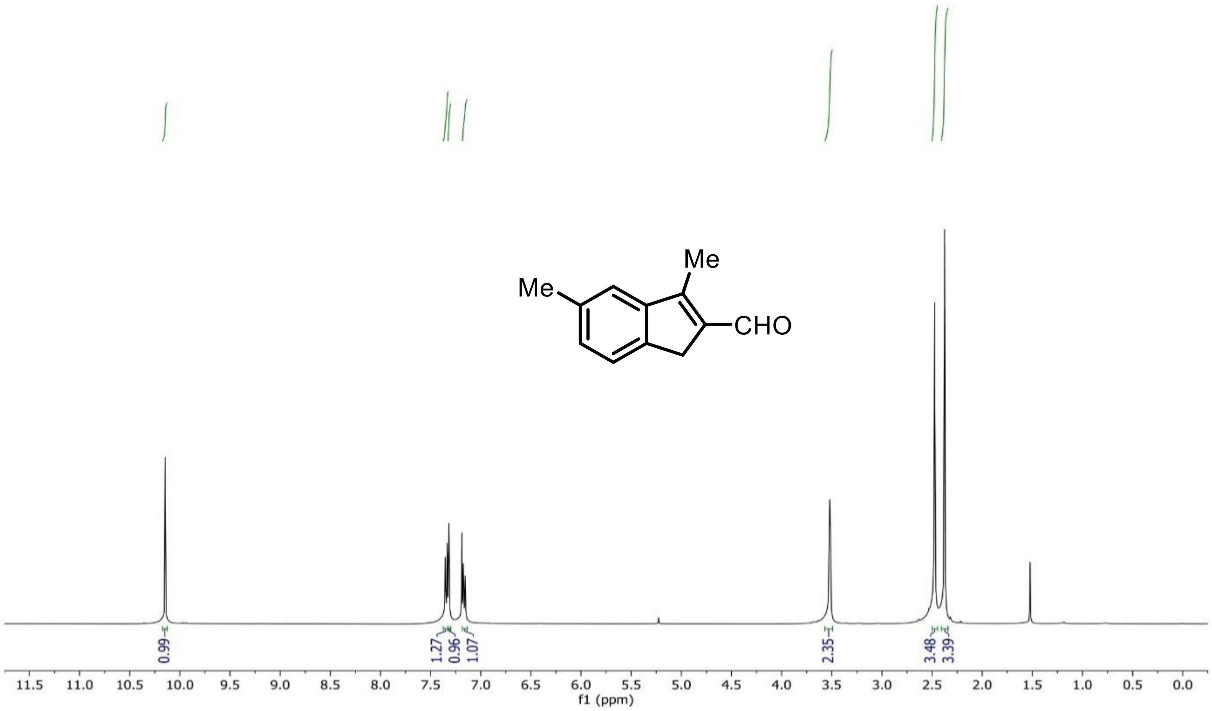

Compound **1f** ^{13}C NMR

(100 MHz, $CDCl_3$)

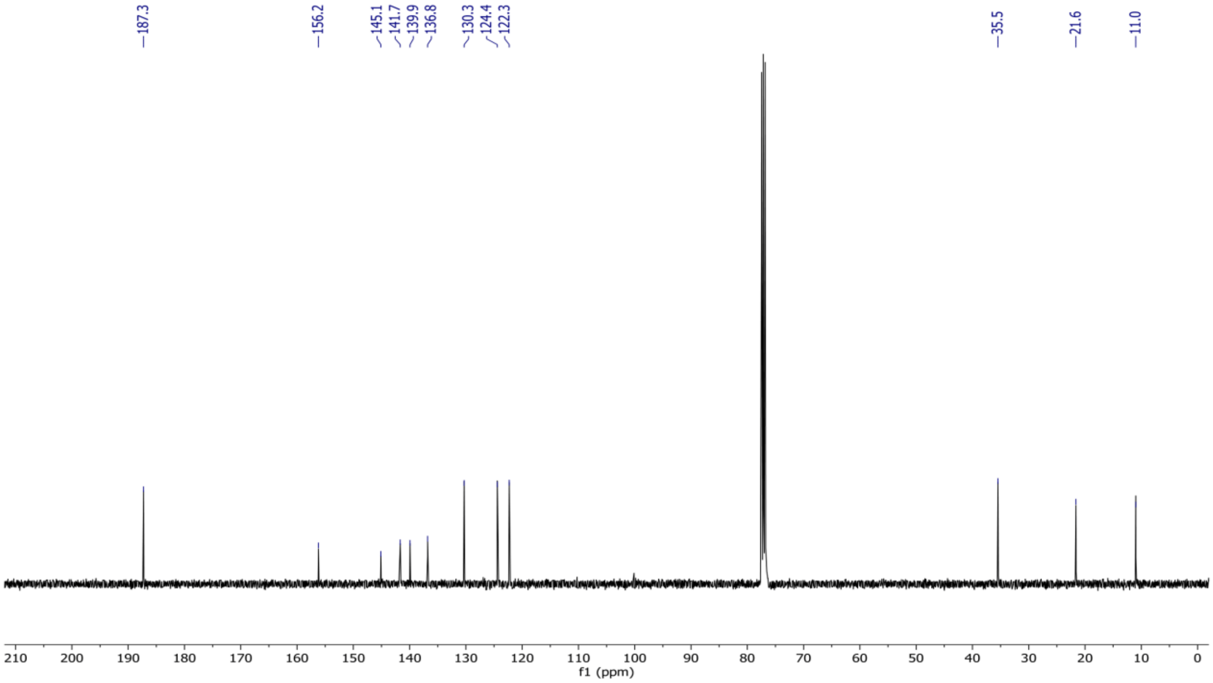

Compound **1g** 1H NMR

(400 MHz, $CDCl_3$)

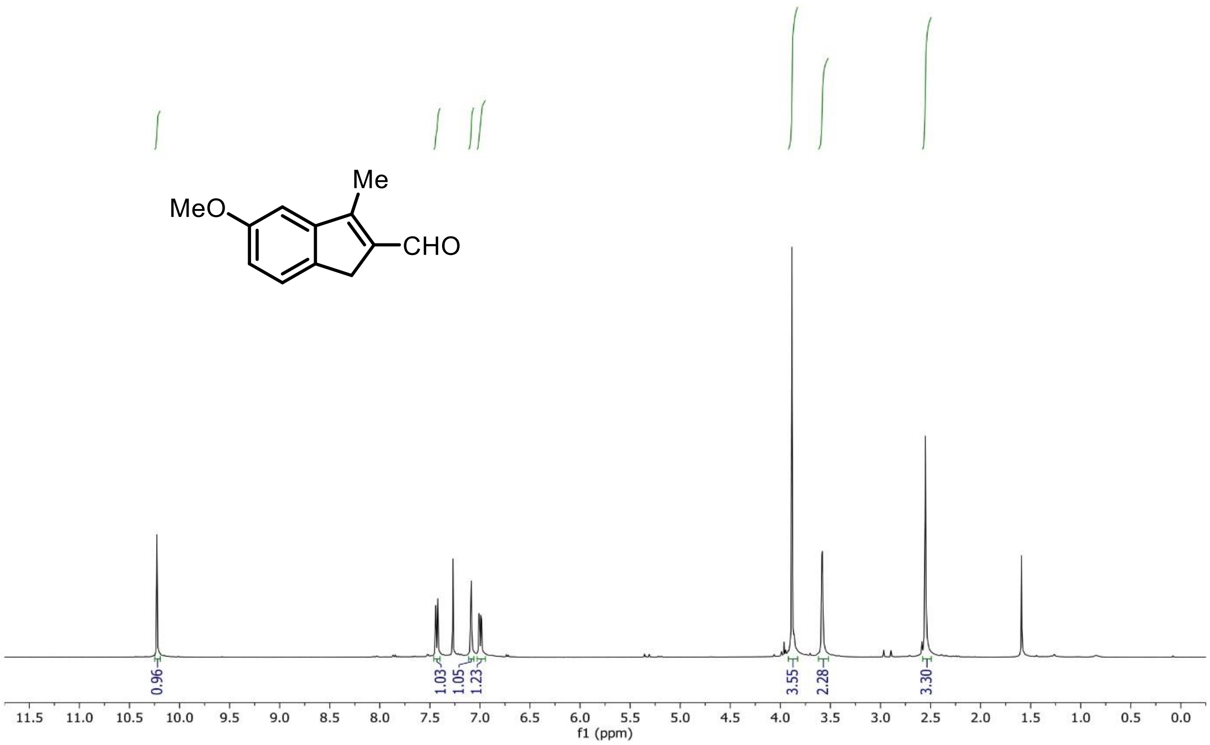

Compound **1g** ^{13}C NMR

(100 MHz, $CDCl_3$)

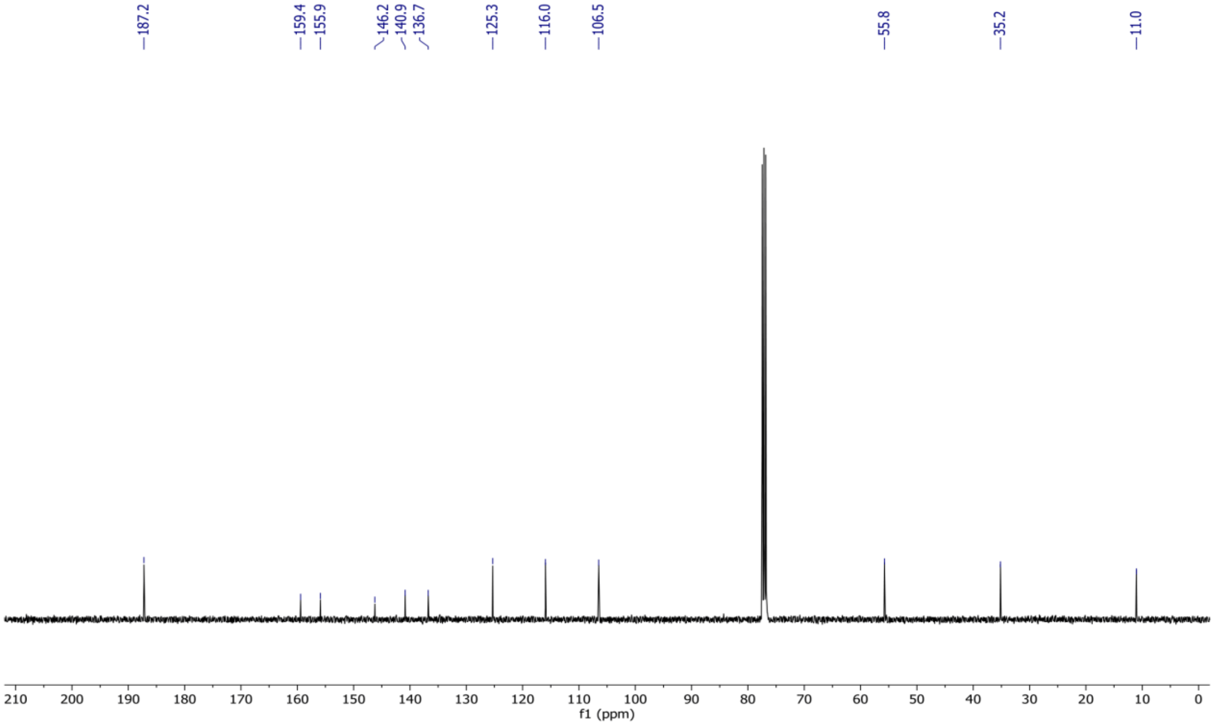

Compound **1i** ^{1}H NMR

(400 MHz, $CDCl_3$)

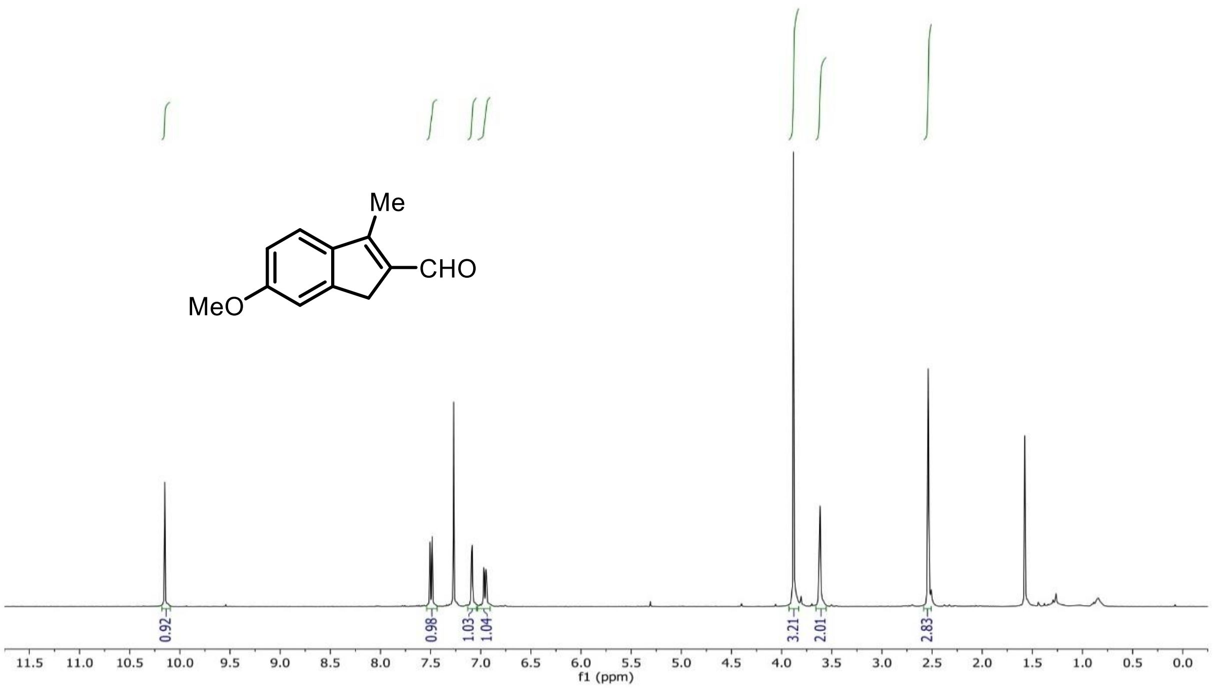

Compound **1i** ^{13}C NMR

(100 MHz, $CDCl_3$)

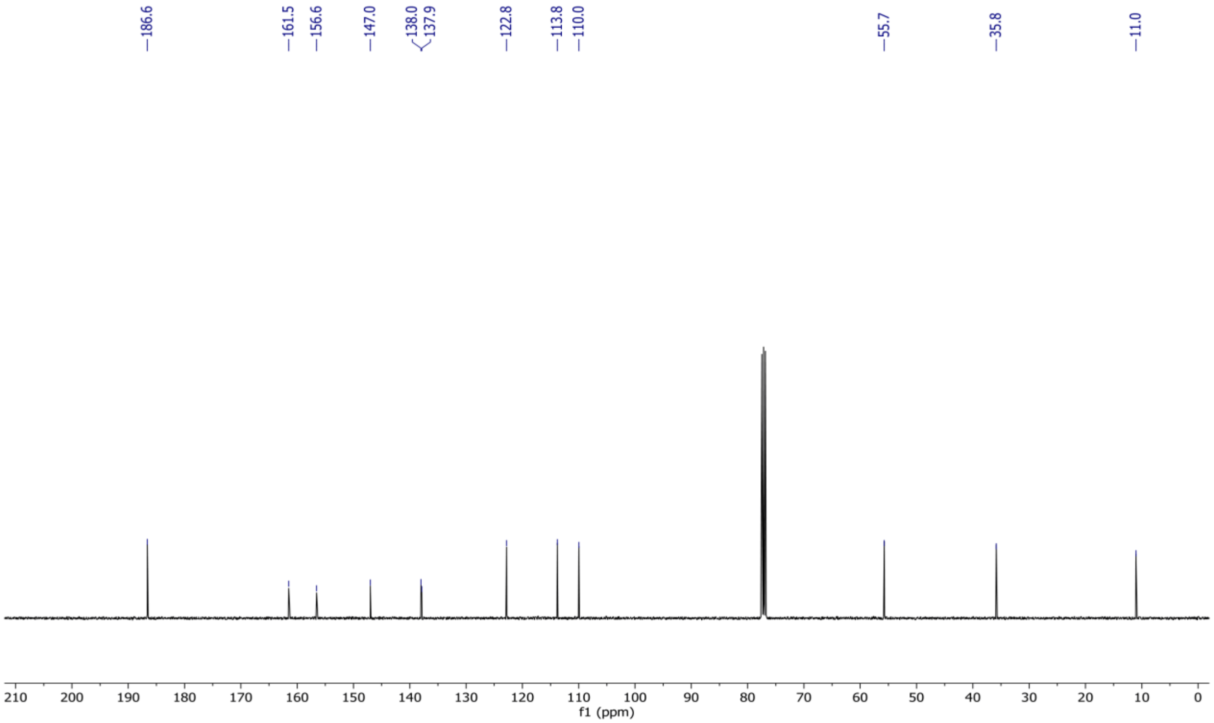

Compound **1m** ^{1}H NMR

(400 MHz, $CDCl_3$)

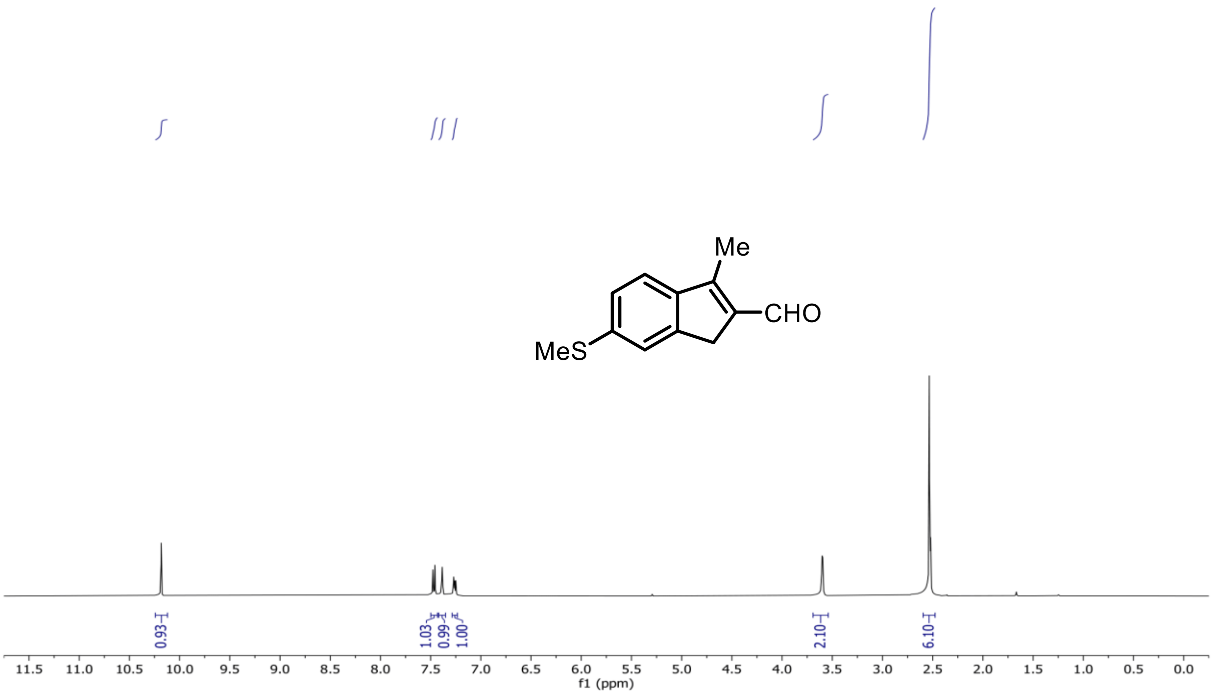

Compound **1m** ^{13}C NMR

(100 MHz, $CDCl_3$)

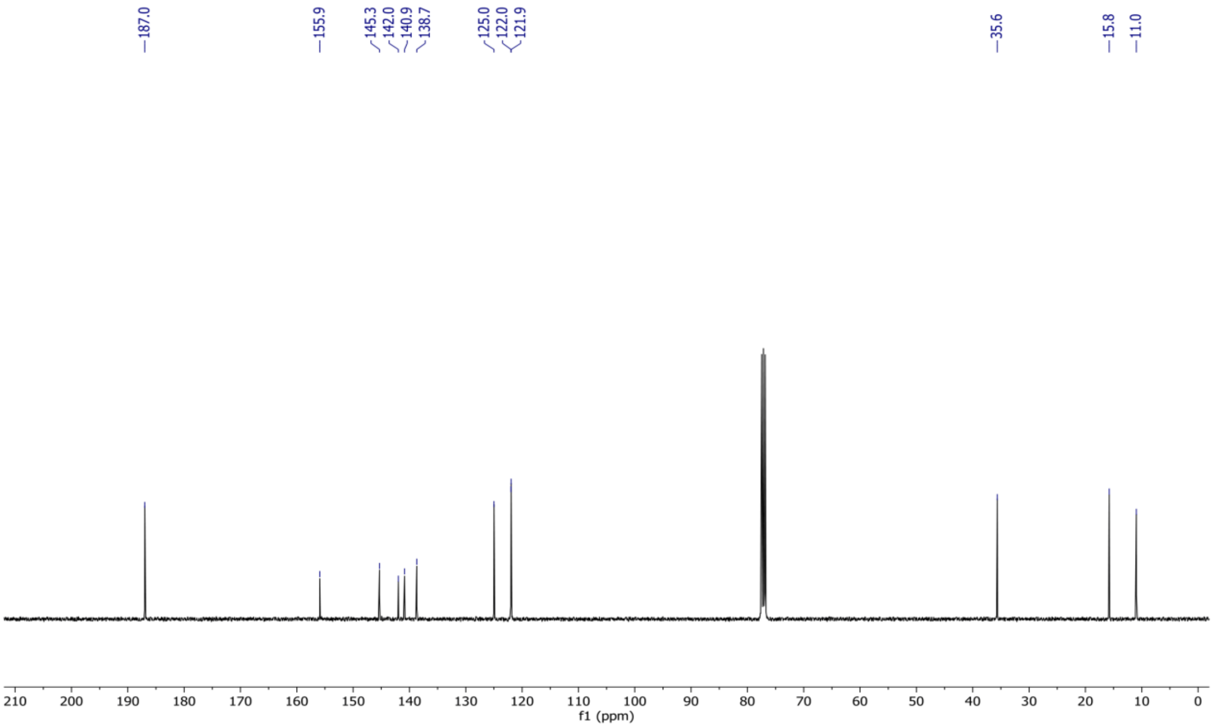

Compound **2c** ^{1}H NMR

(400 MHz, $CDCl_3$)

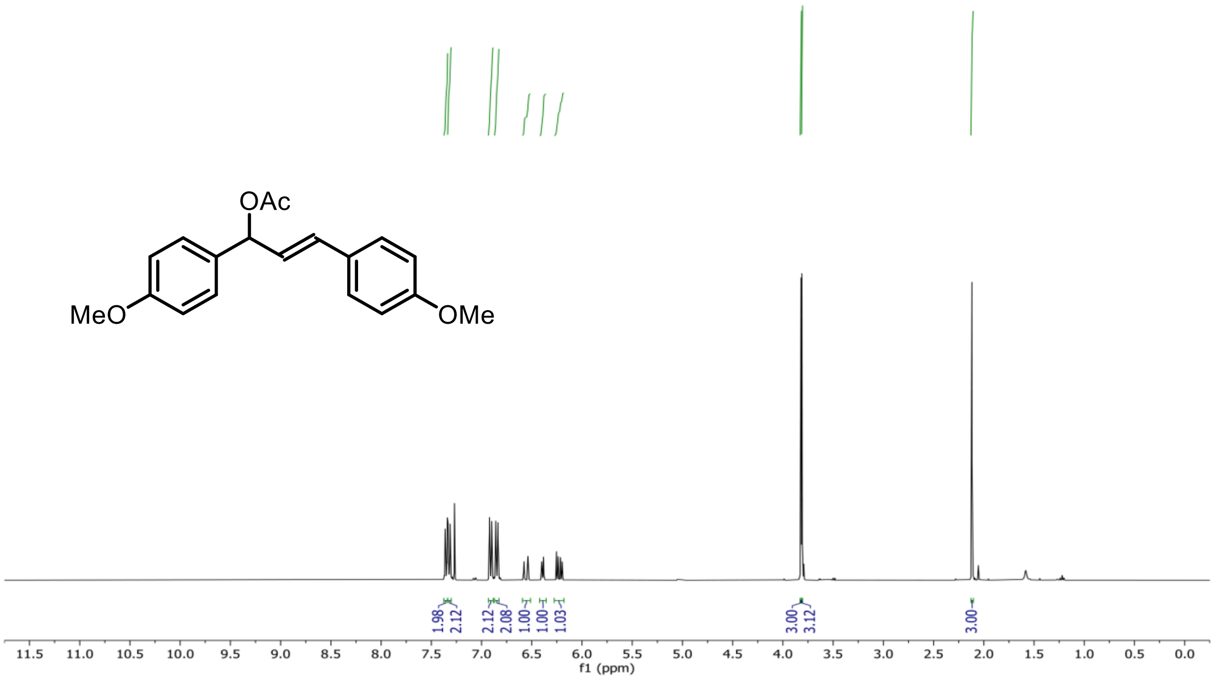

Compound **2c** ^{13}C NMR

(100 MHz, $CDCl_3$)

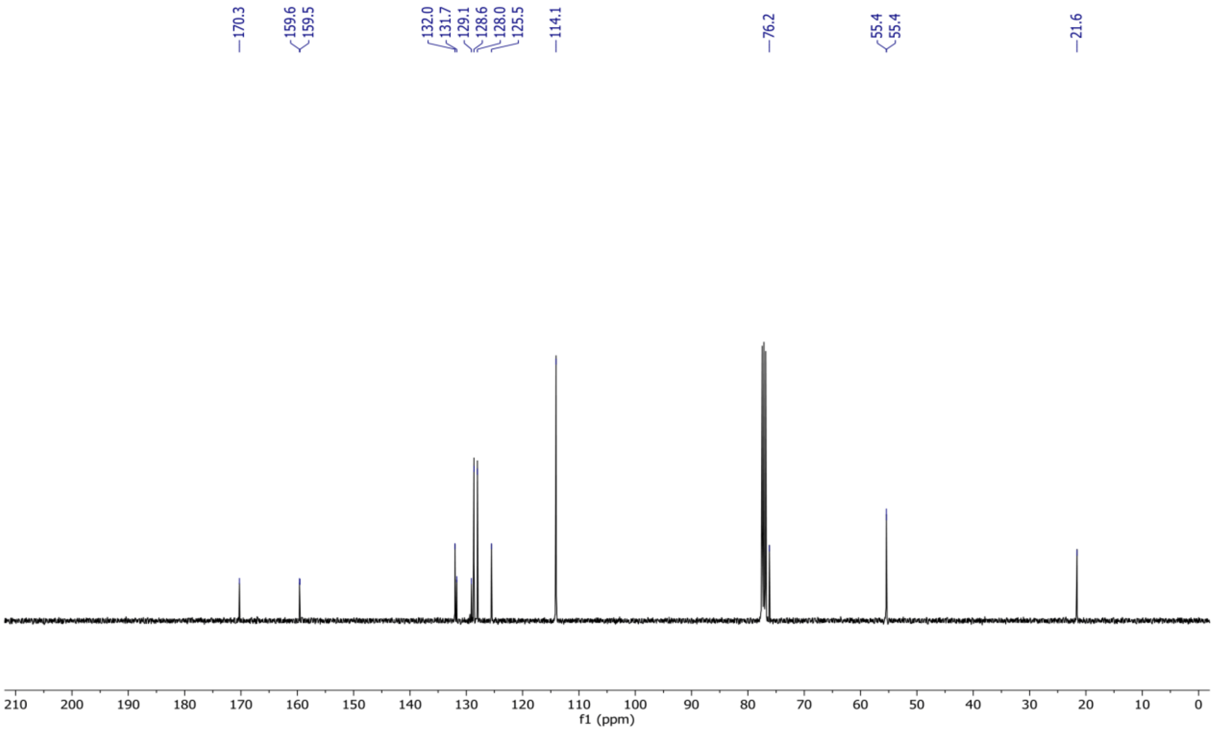

Compound **2d** ^{1}H NMR

(400 MHz, $CDCl_3$)

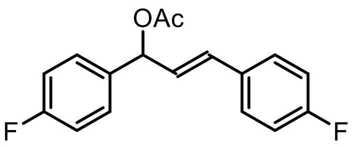

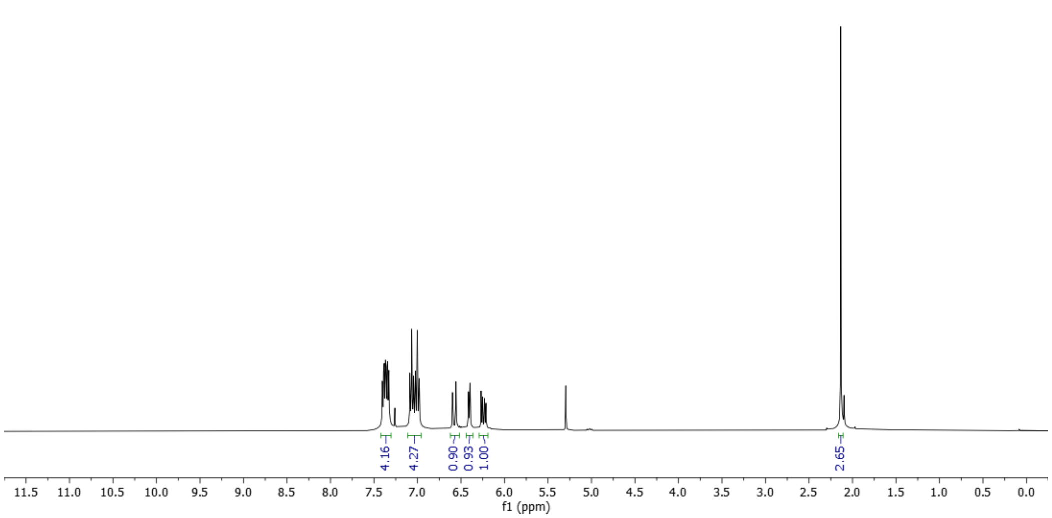

Compound **2d** ^{13}C NMR

(100 MHz, $CDCl_3$)

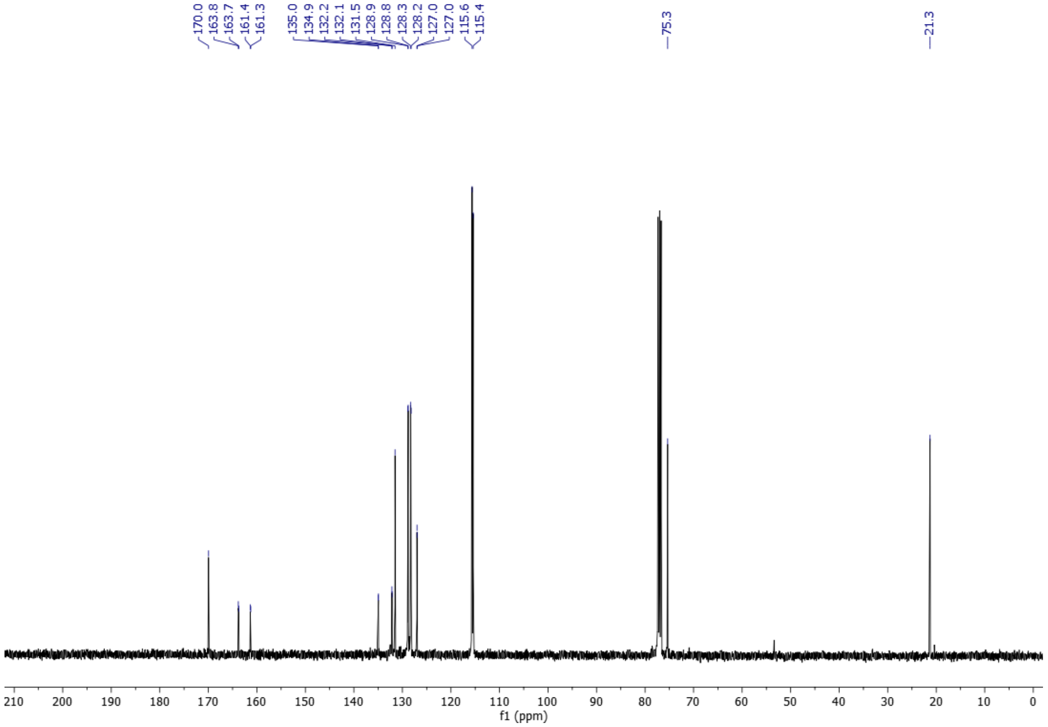

Compound **2d** ^{19}F NMR

(375 MHz, $CDCl_3$)

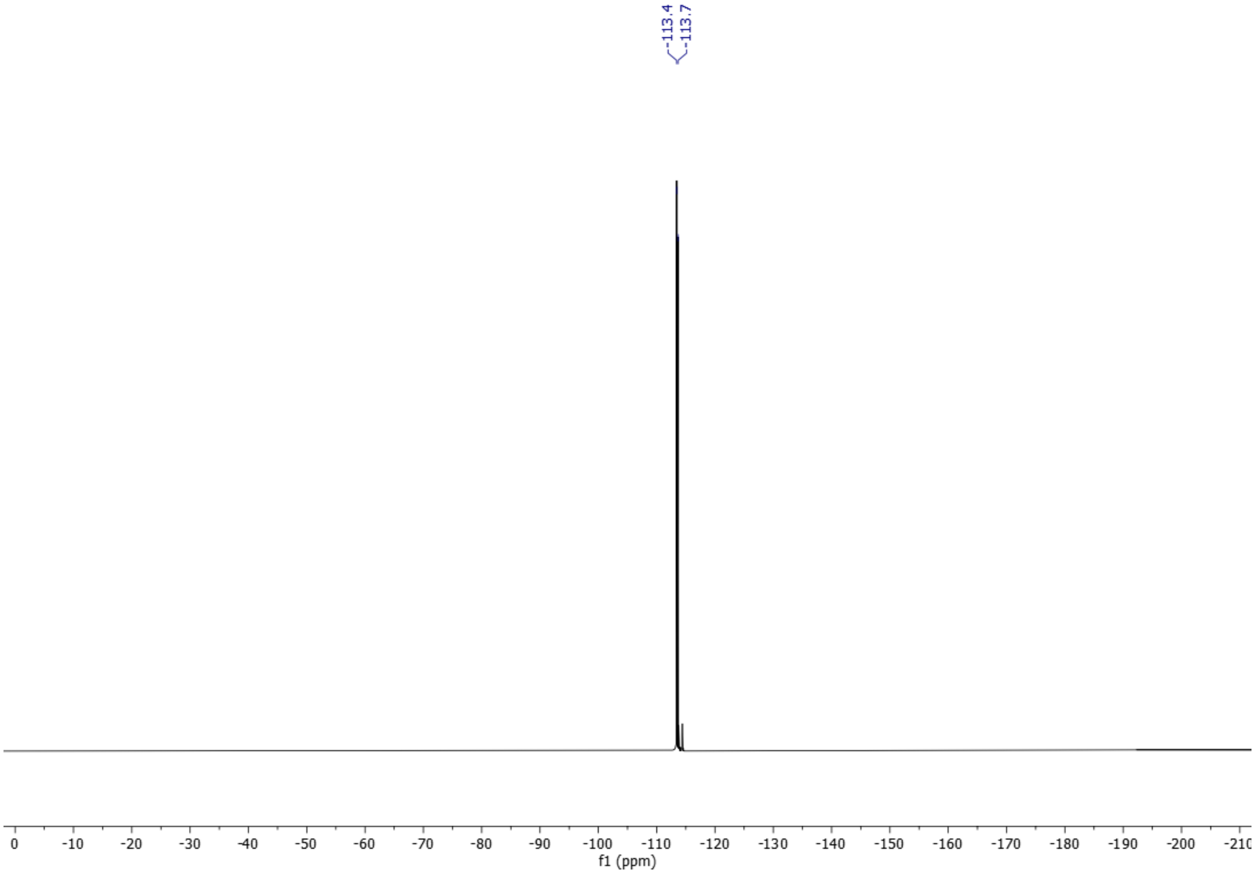

Compound **2e** ^{1}H NMR

(400 MHz, $CDCl_3$)

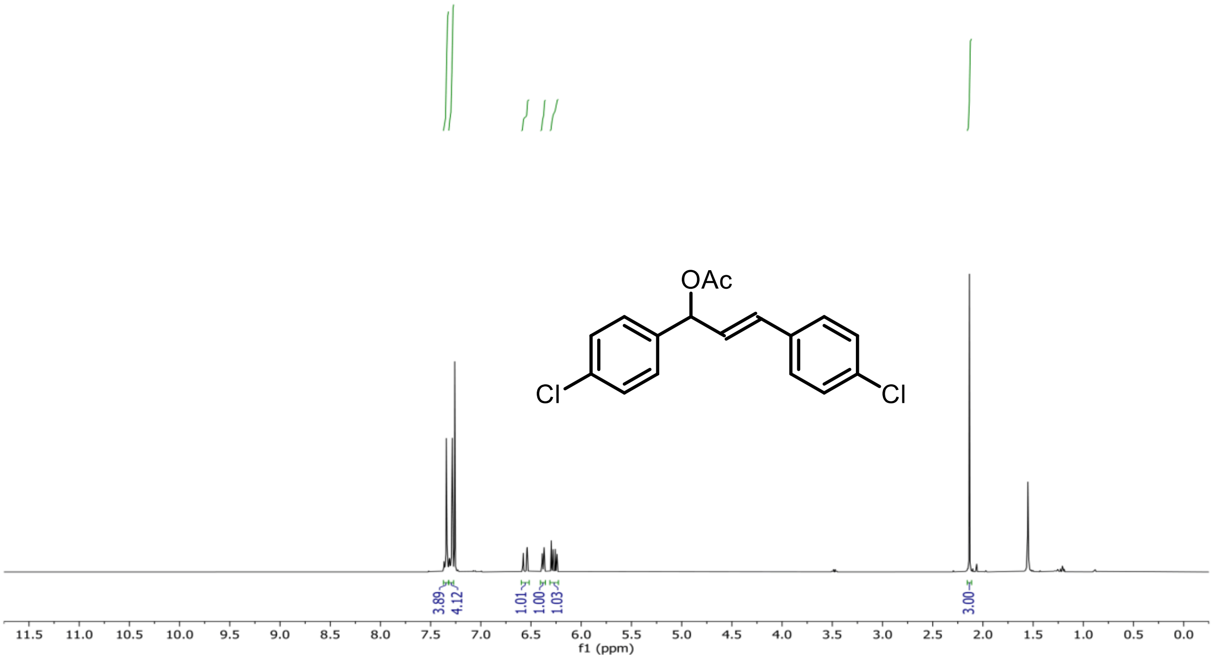

Compound **2e** ^{13}C NMR

(100 MHz, $CDCl_3$)

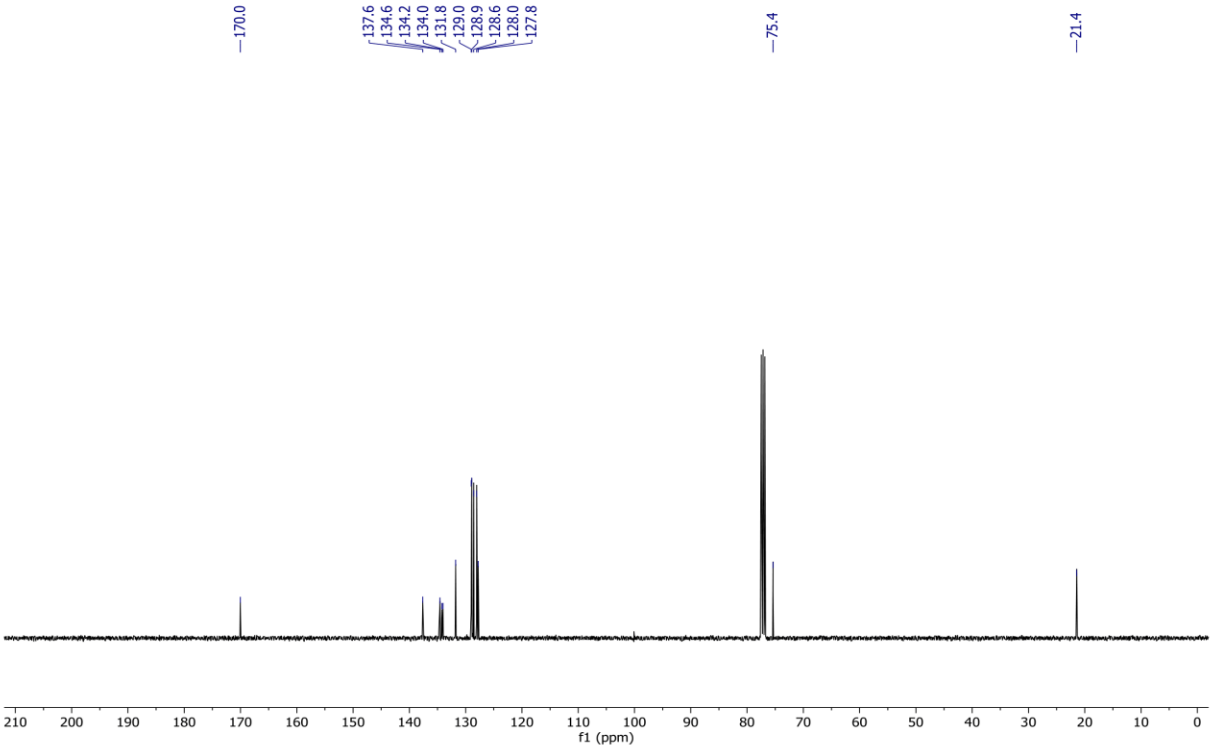

Compound **2g** ^{1}H NMR

(400 MHz, $CDCl_3$)

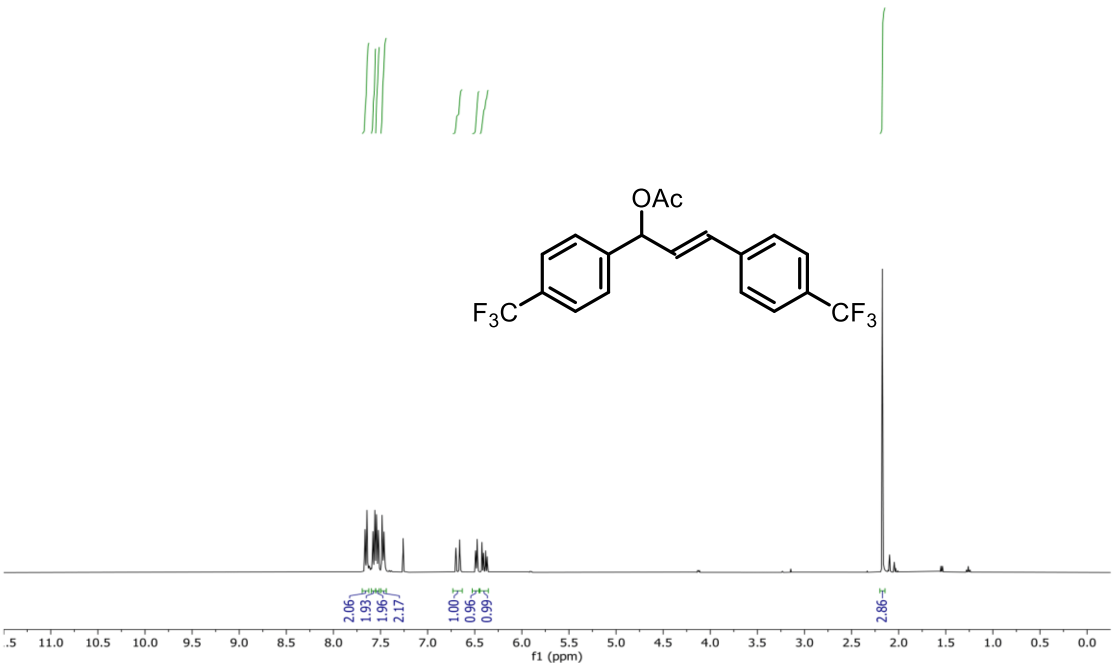

Compound **2g** ^{13}C NMR

(100 MHz, $CDCl_3$)

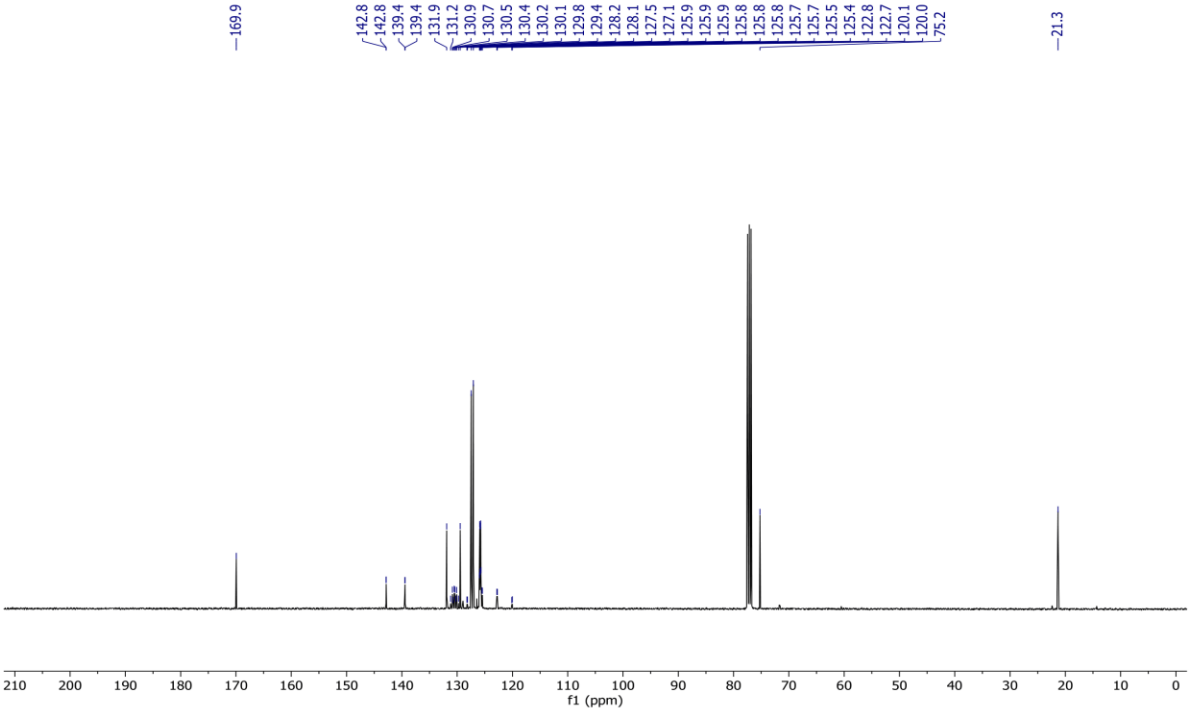

Compound **2g** ^{19}F NMR

(375 MHz, $CDCl_3$)

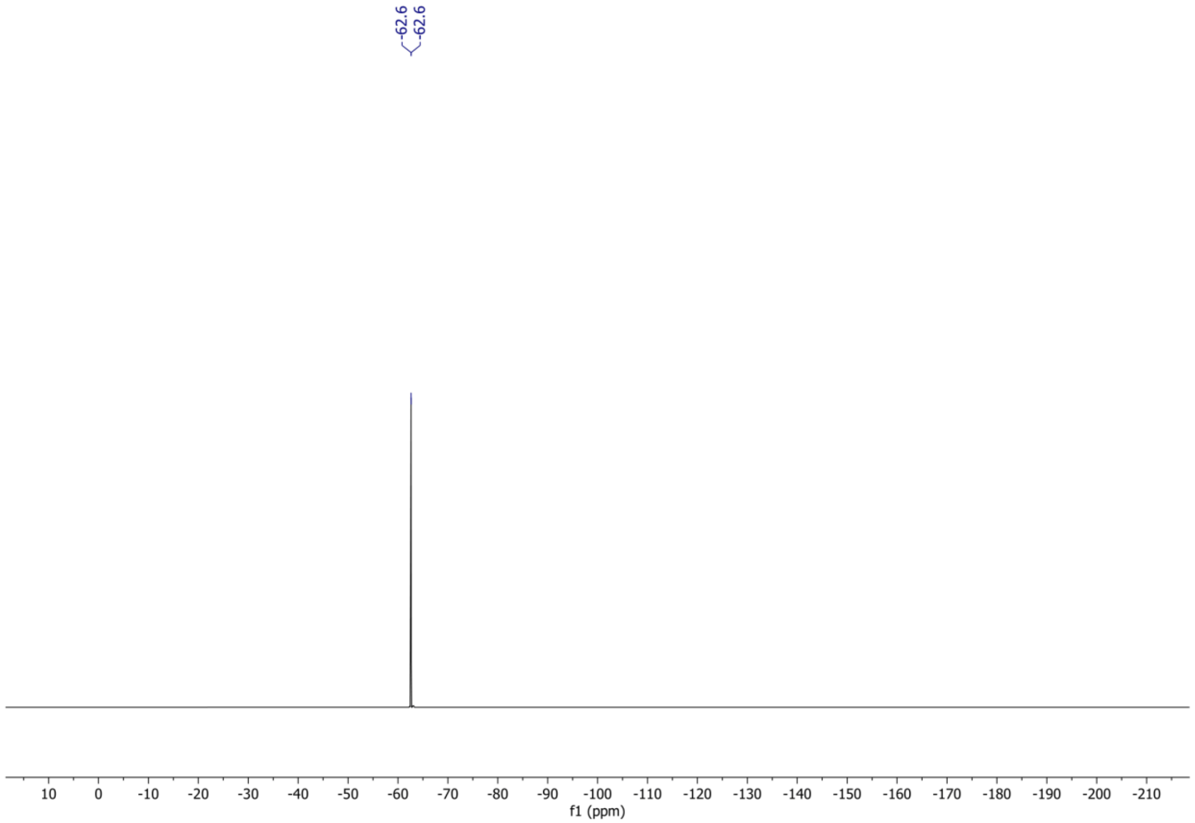

Compound **2i** ^{1}H NMR

(400 MHz, $CDCl_3$)

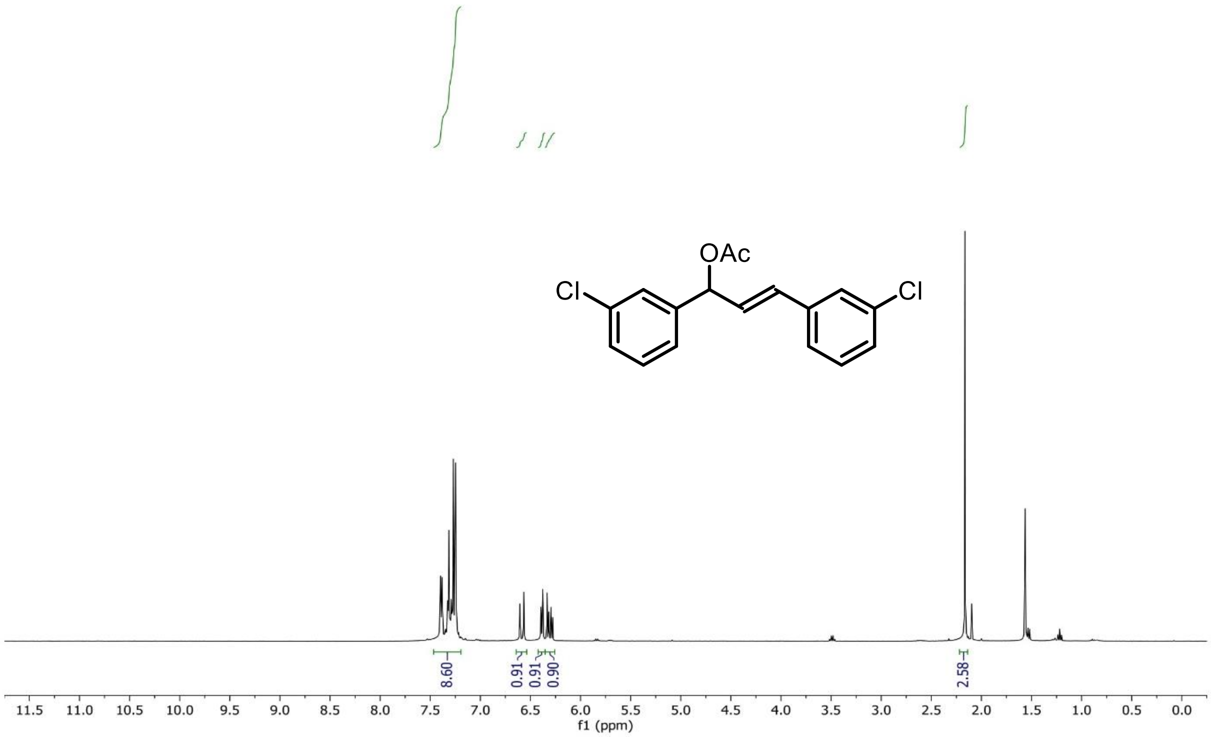

Compound **2i** ^{13}C NMR

(100 MHz, $CDCl_3$)

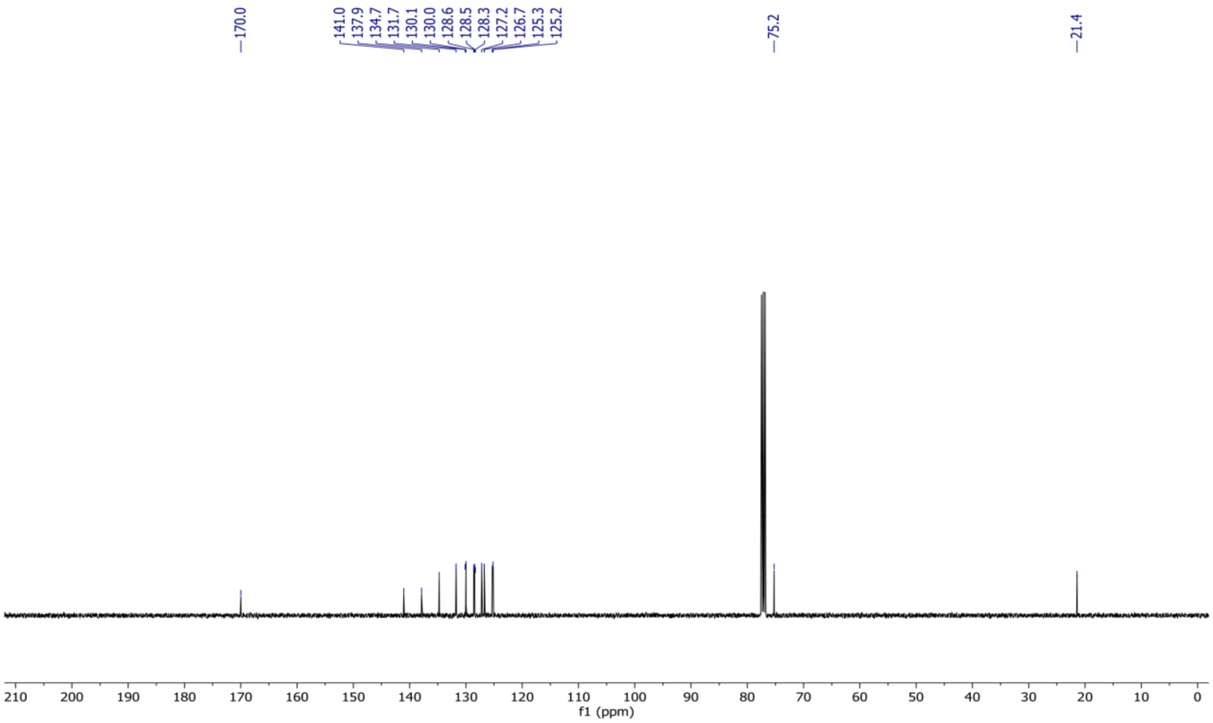

Compound **2j** ^{1}H NMR

(400 MHz, $CDCl_3$)

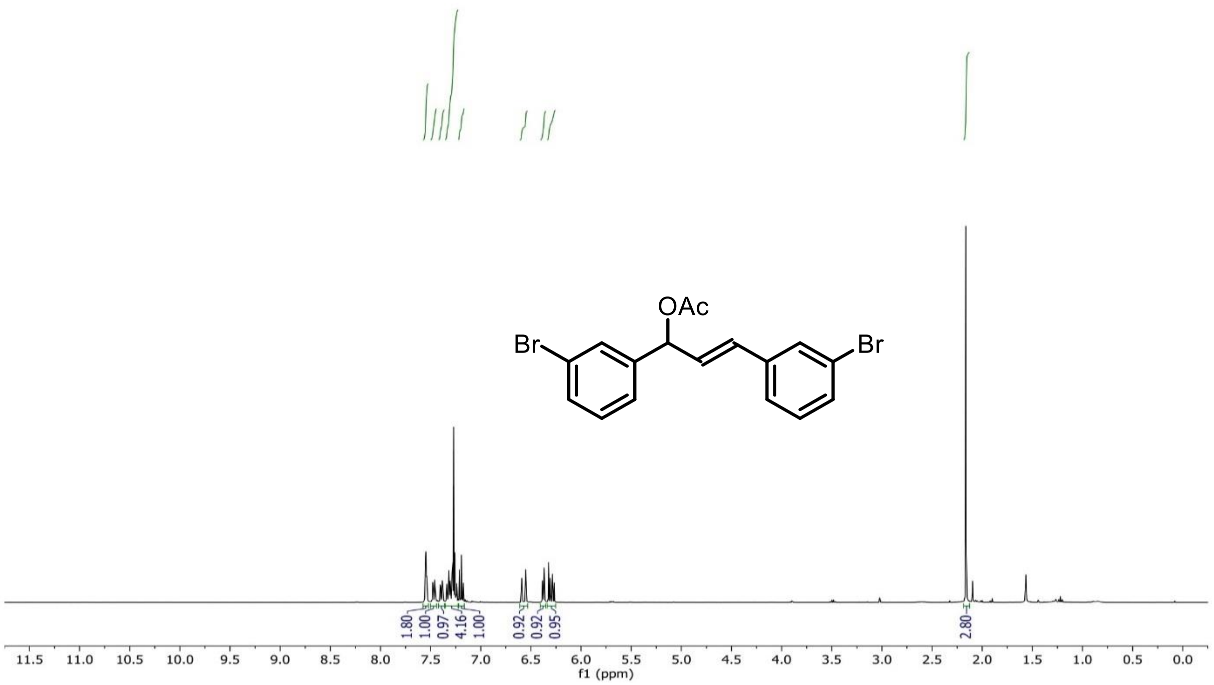

Compound **2j** ^{13}C NMR

(100 MHz, $CDCl_3$)

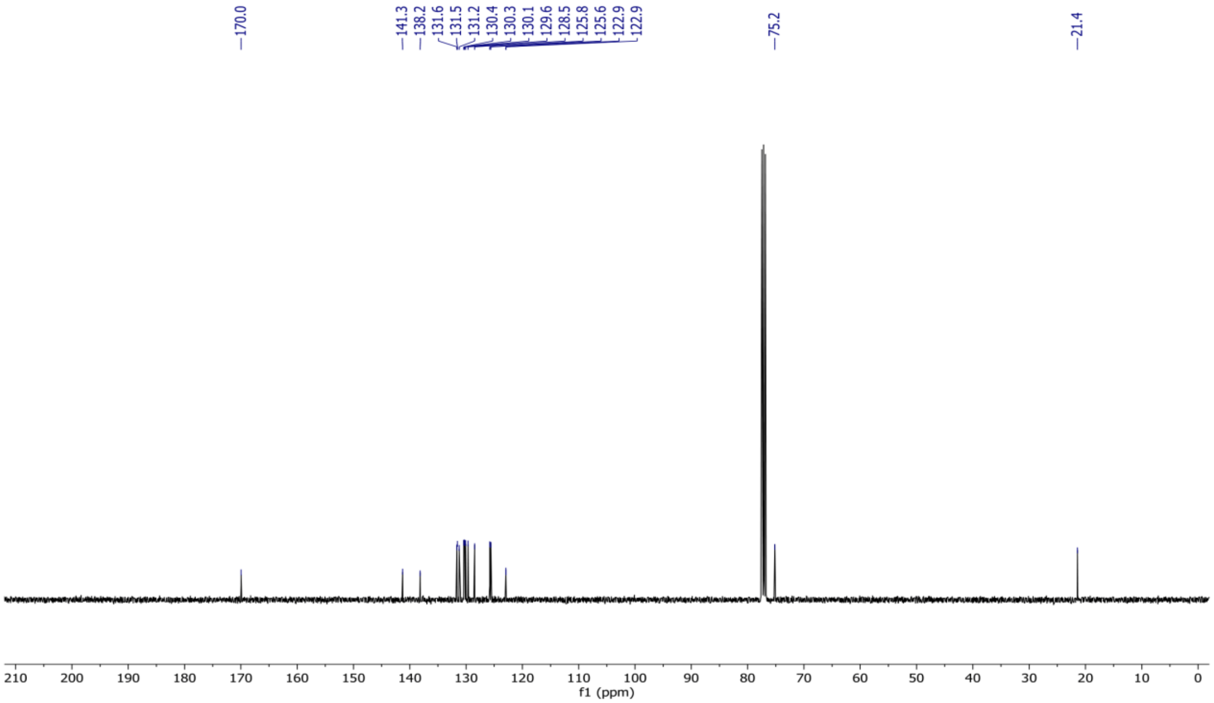

Compound **4a** ^{1}H NMR

(400 MHz, $CDCl_3$)

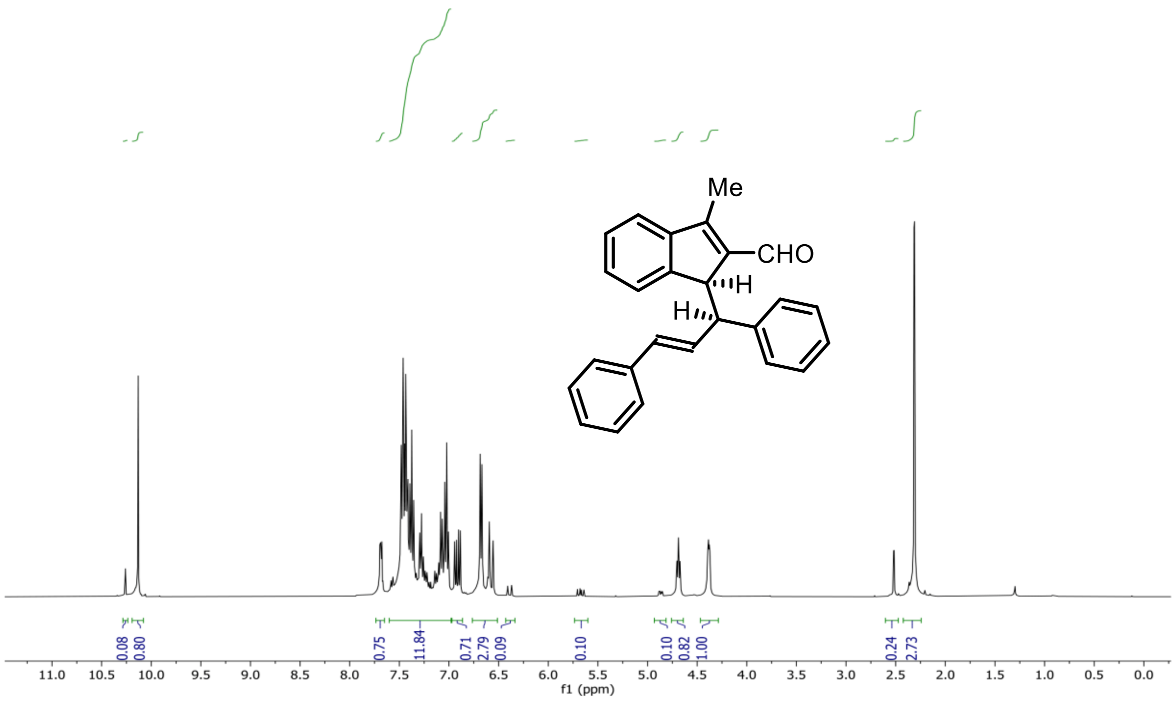

Compound **4a** ^{13}C NMR

(100 MHz, $CDCl_3$)

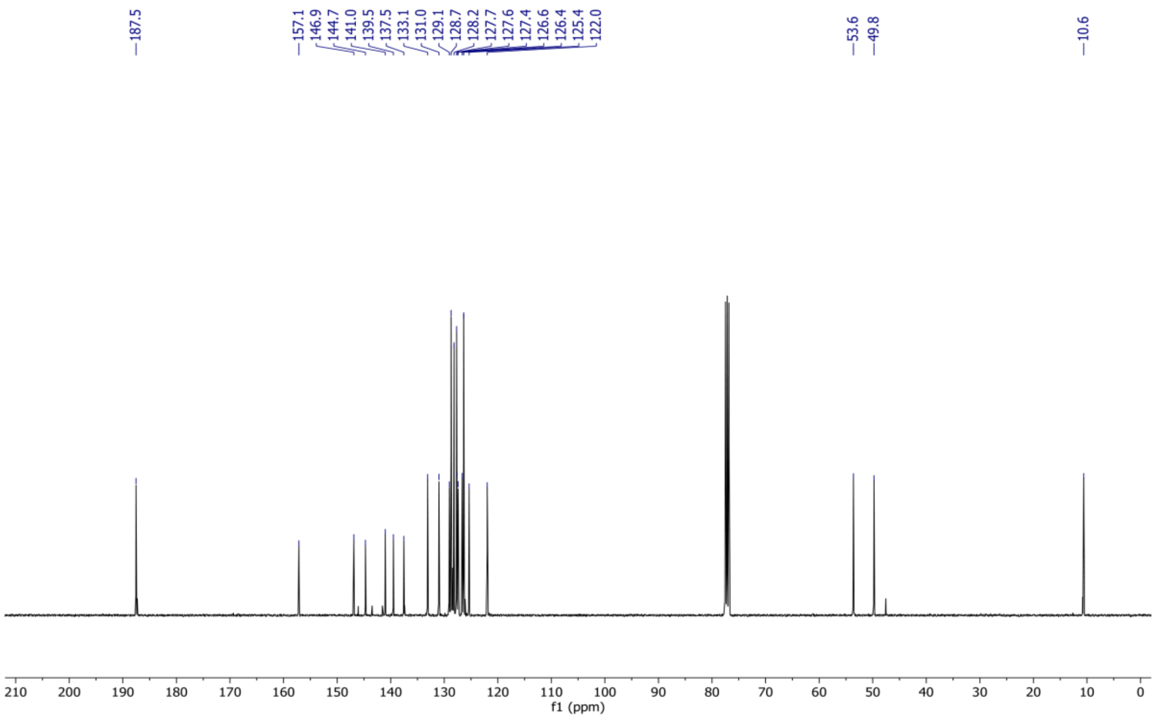

Compound **5a** ^{1}H NMR

(400 MHz, $CDCl_3$)

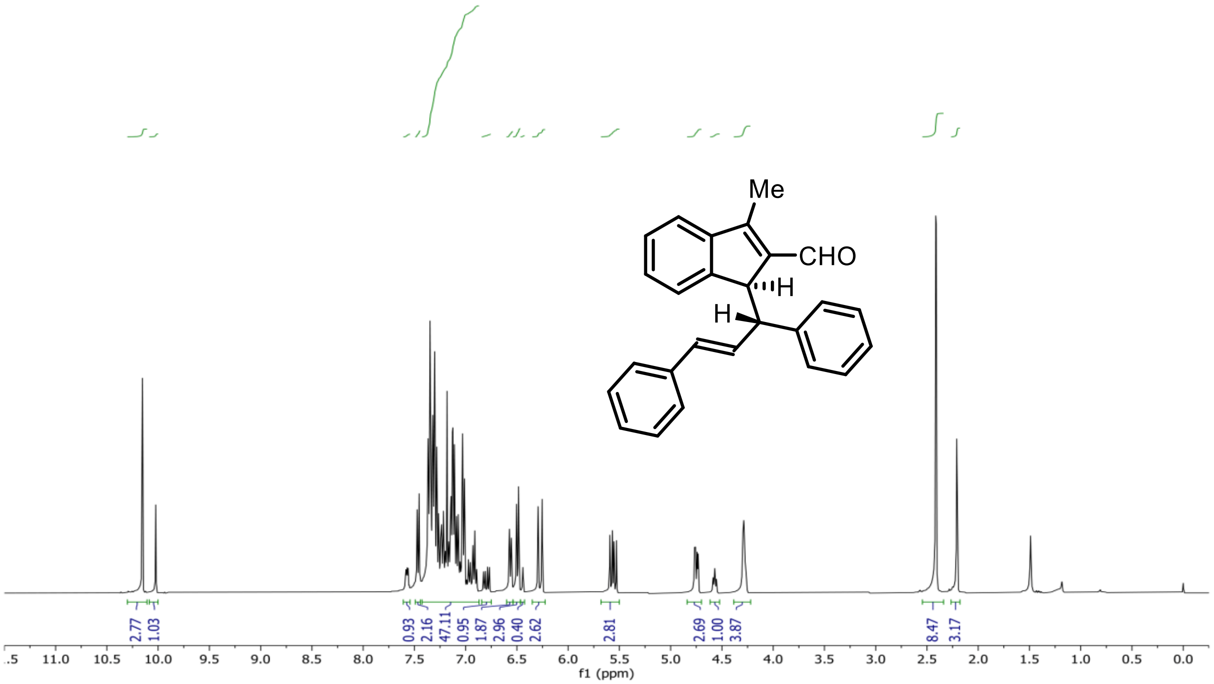

Compound **5a** ^{13}C NMR

(100 MHz, $CDCl_3$)

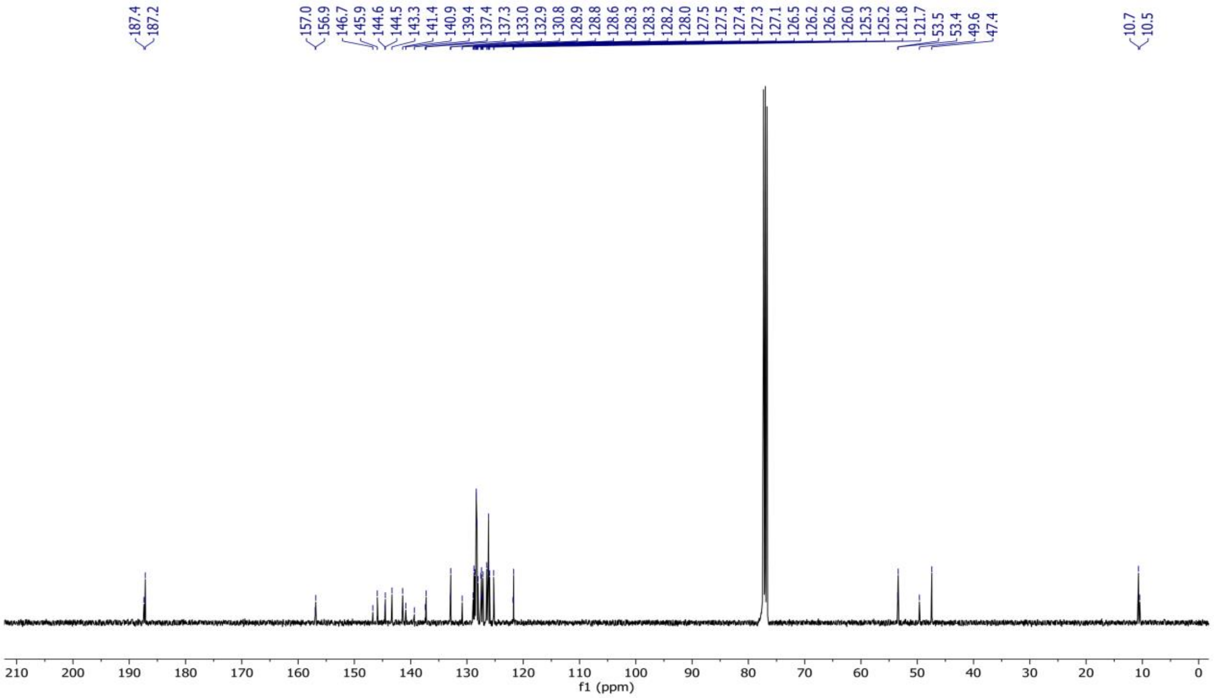

Compound **4b** ^{1}H NMR

(400 MHz, $CDCl_3$)

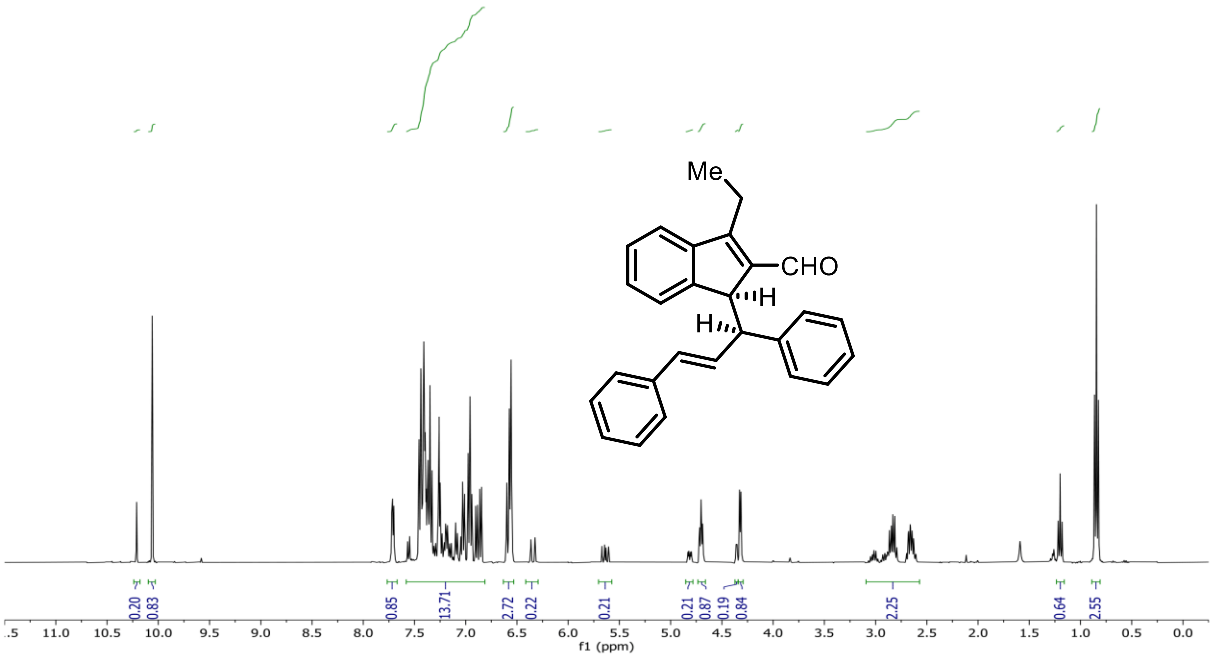

Compound **4b** ^{13}C NMR

(100 MHz, $CDCl_3$)

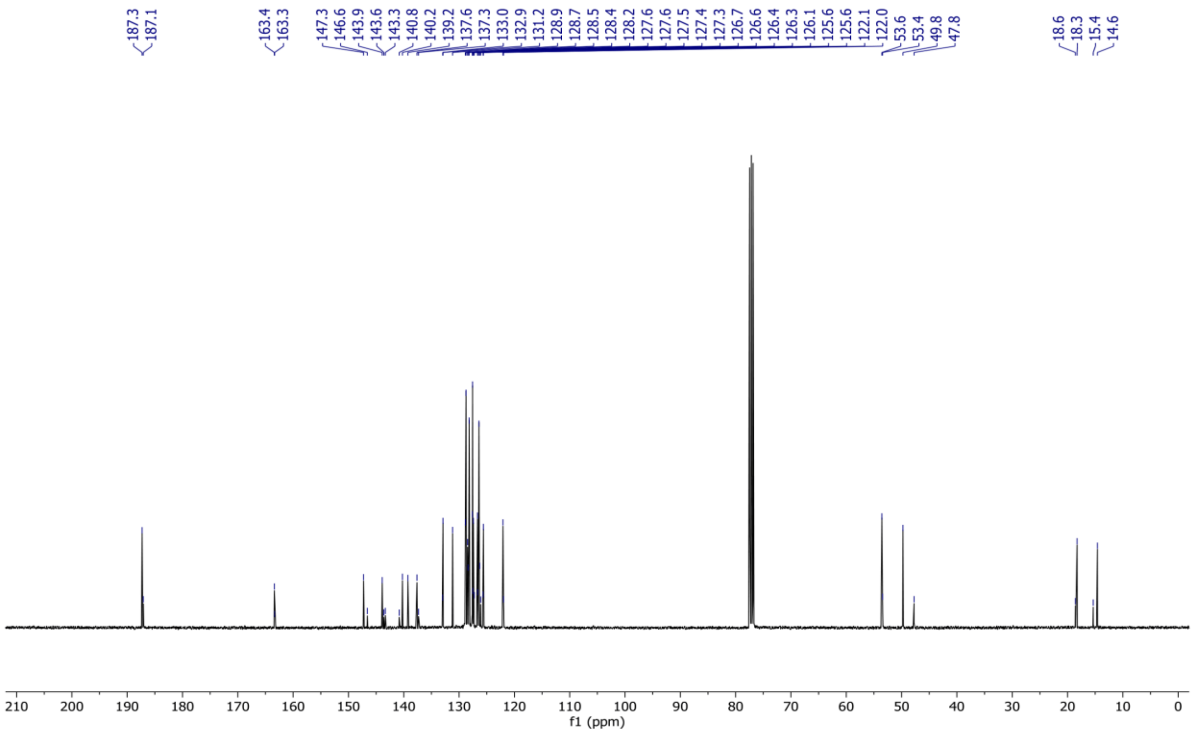

Compound **5b** ^{1}H NMR

(400 MHz, $CDCl_3$)

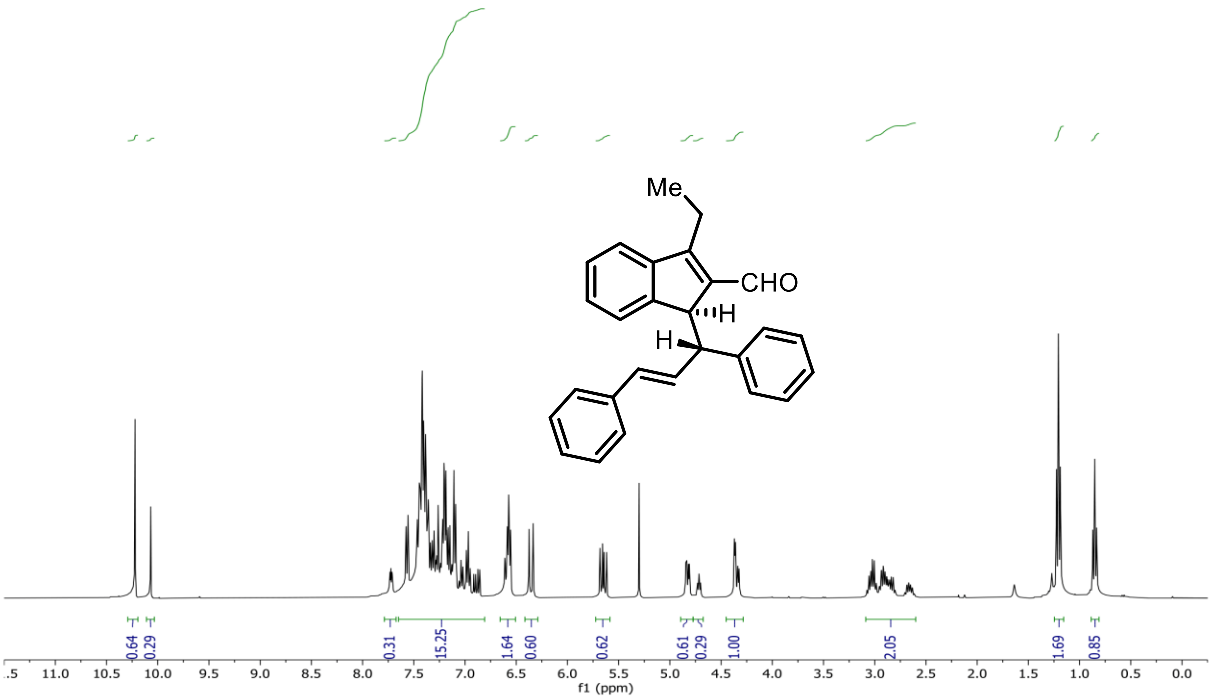

Compound **5b** ^{13}C NMR

(100 MHz, $CDCl_3$)

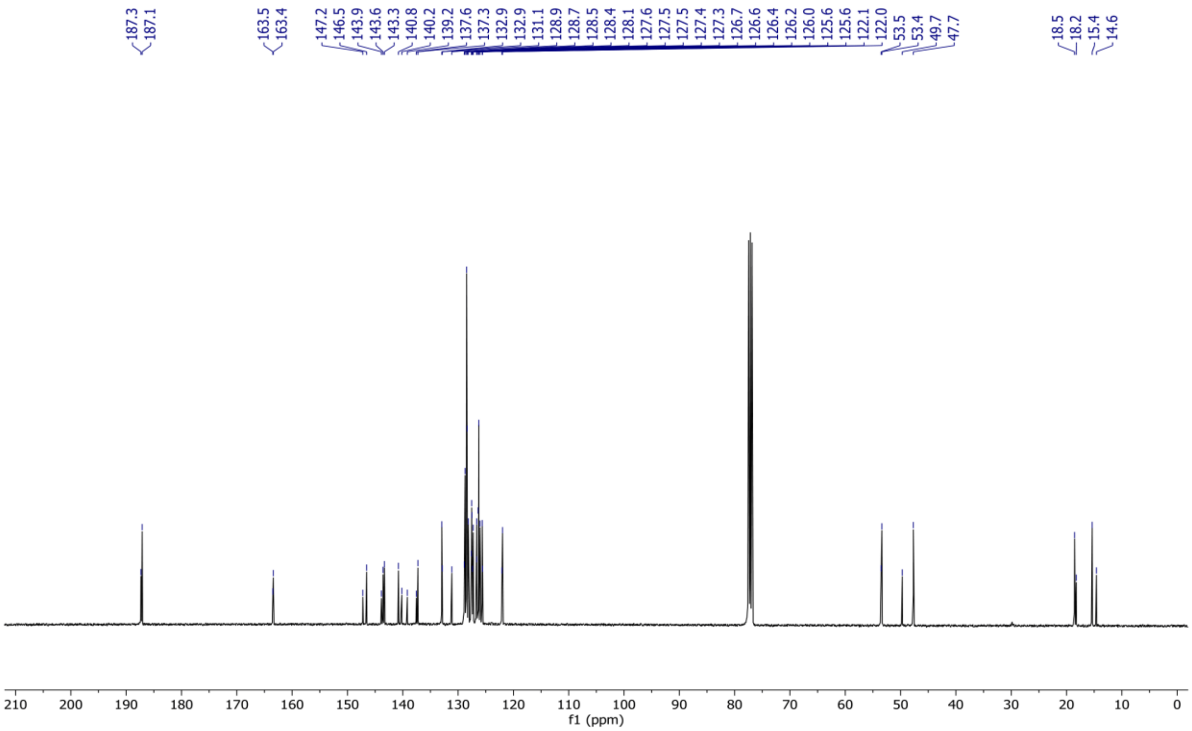

Compound **4c** ^{1}H NMR

(400 MHz, $CDCl_3$)

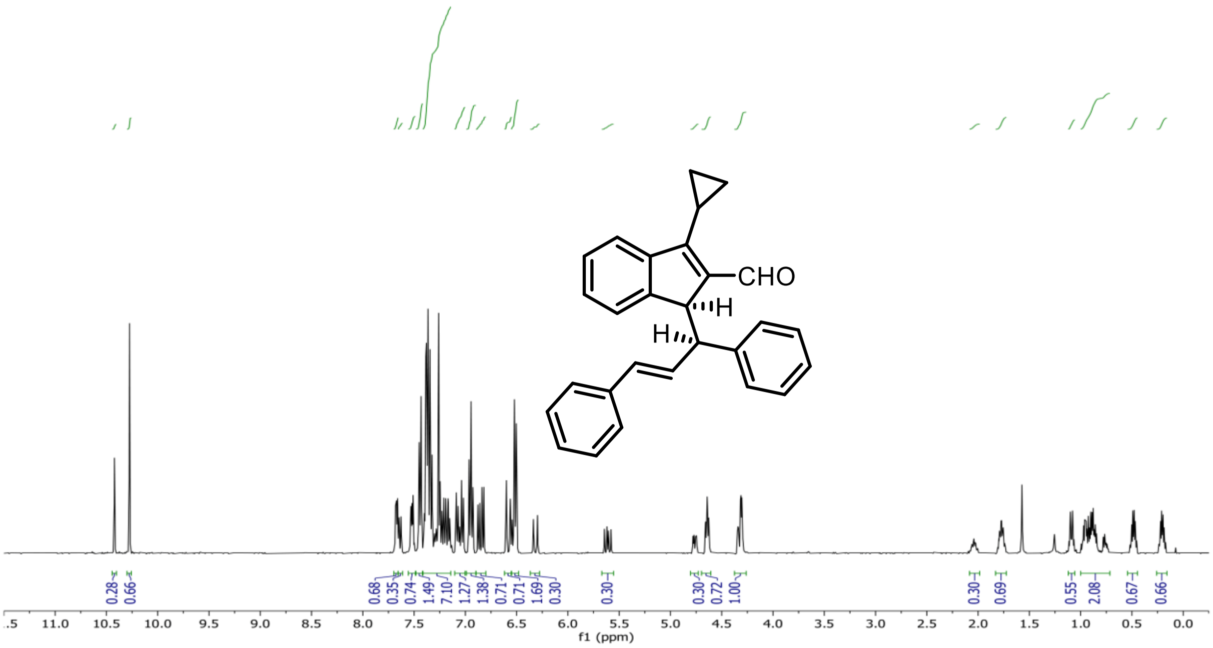

Compound **4c** ^{13}C NMR

(100 MHz, $CDCl_3$)

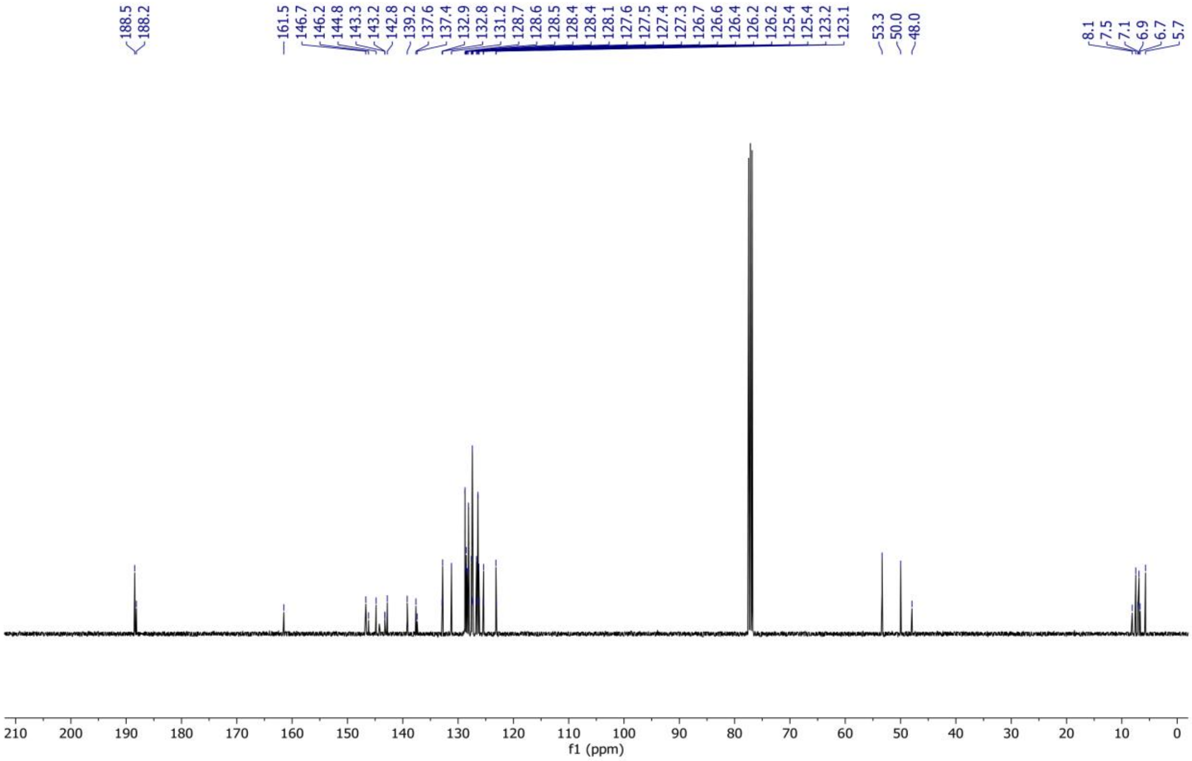

Compound **5c** ^{1}H NMR

(400 MHz, $CDCl_3$)

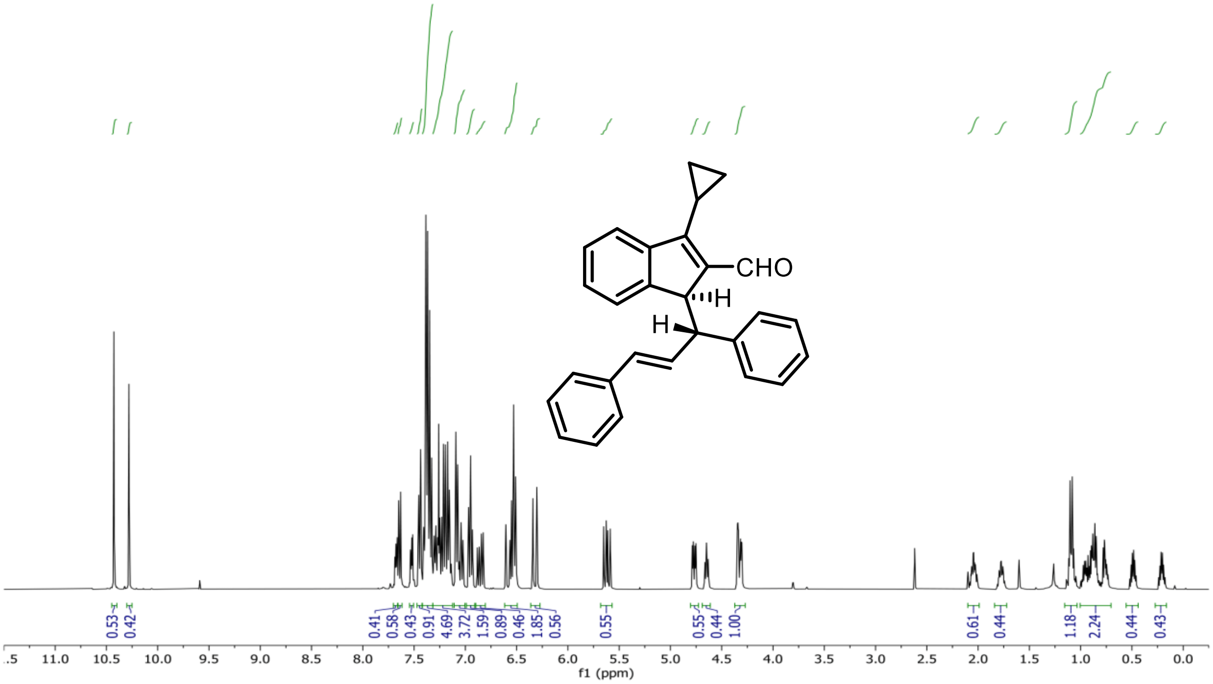

Compound **5c** ^{13}C NMR

(100 MHz, $CDCl_3$)

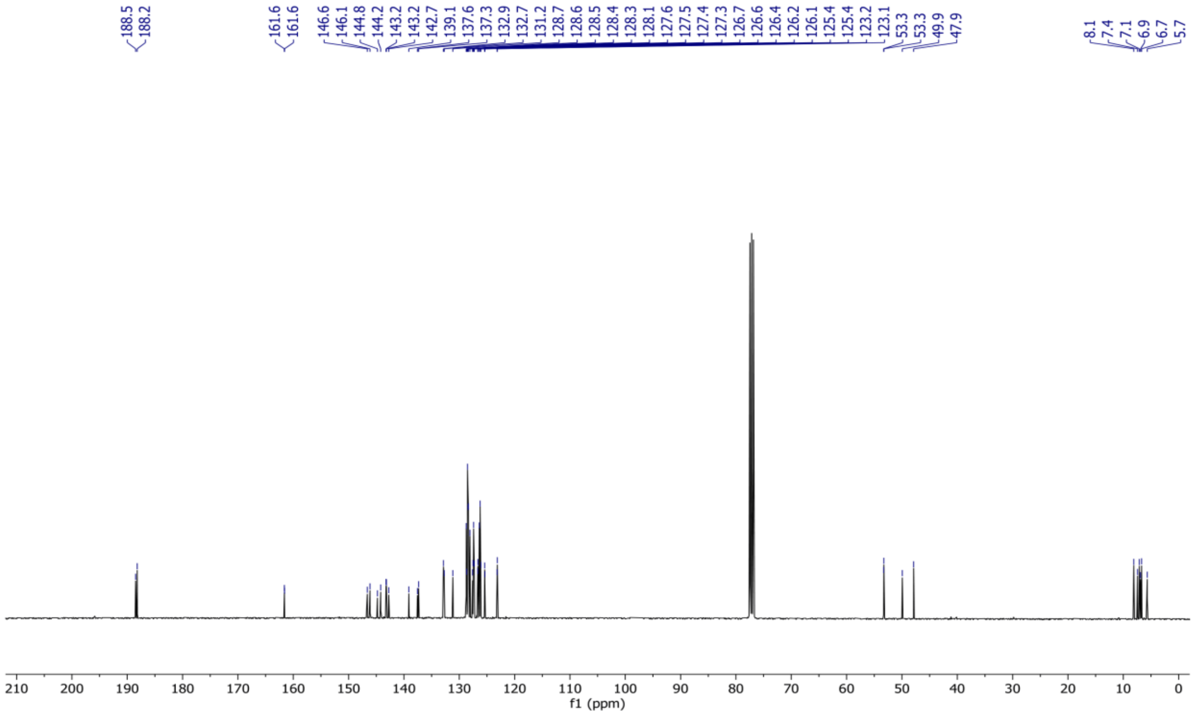

Compound **4d** ^{1}H NMR

(400 MHz, $CDCl_3$)

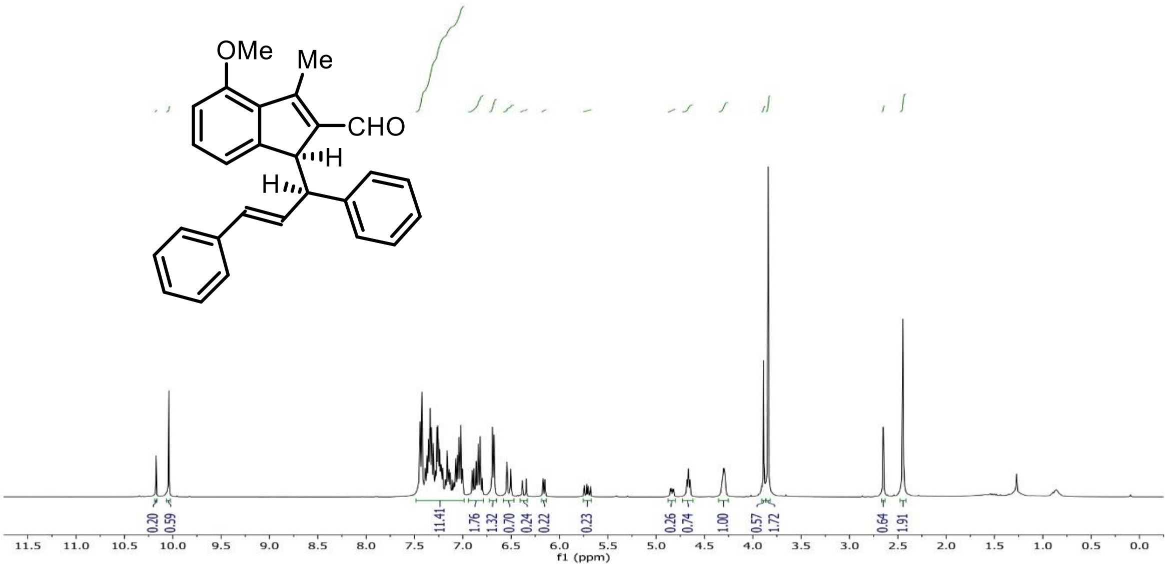

Compound **4d** ^{13}C NMR

(100 MHz, $CDCl_3$)

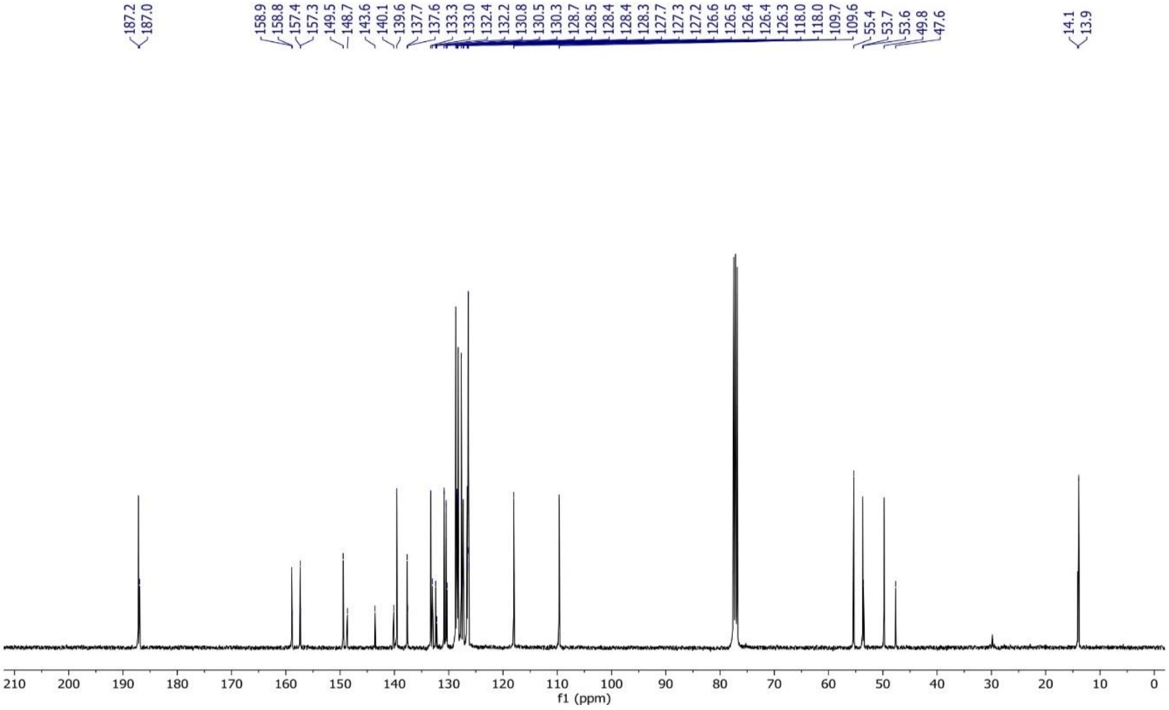

Compound **5d** ^{1}H NMR

(400 MHz, $CDCl_3$)

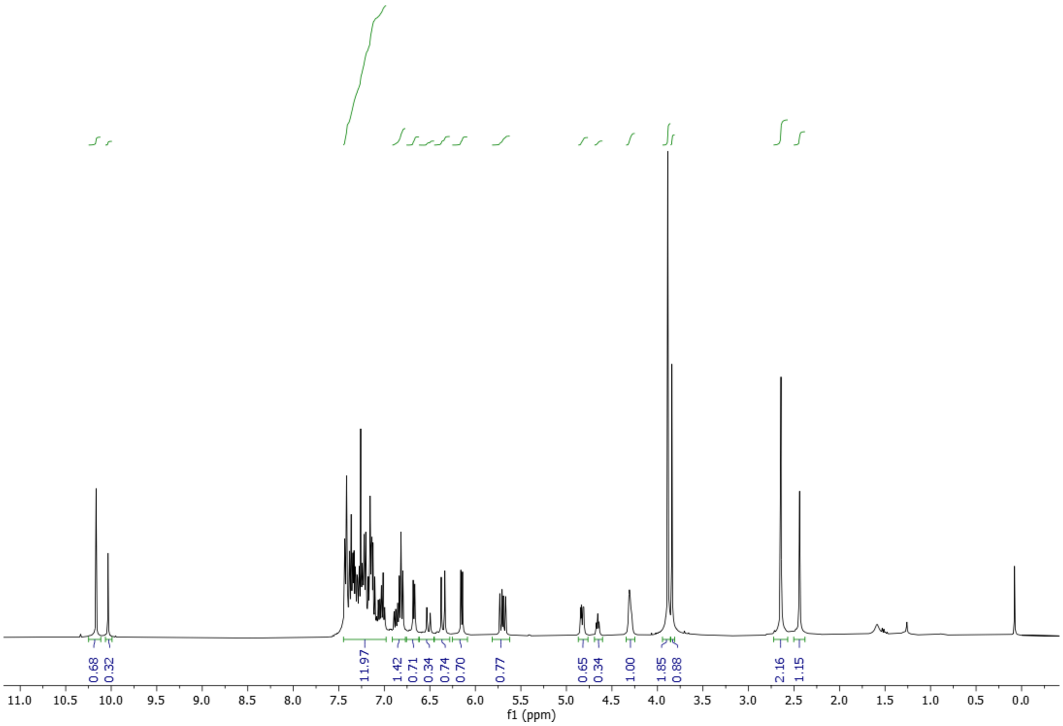

Compound **5d** ^{13}C NMR

(100 MHz, $CDCl_3$)

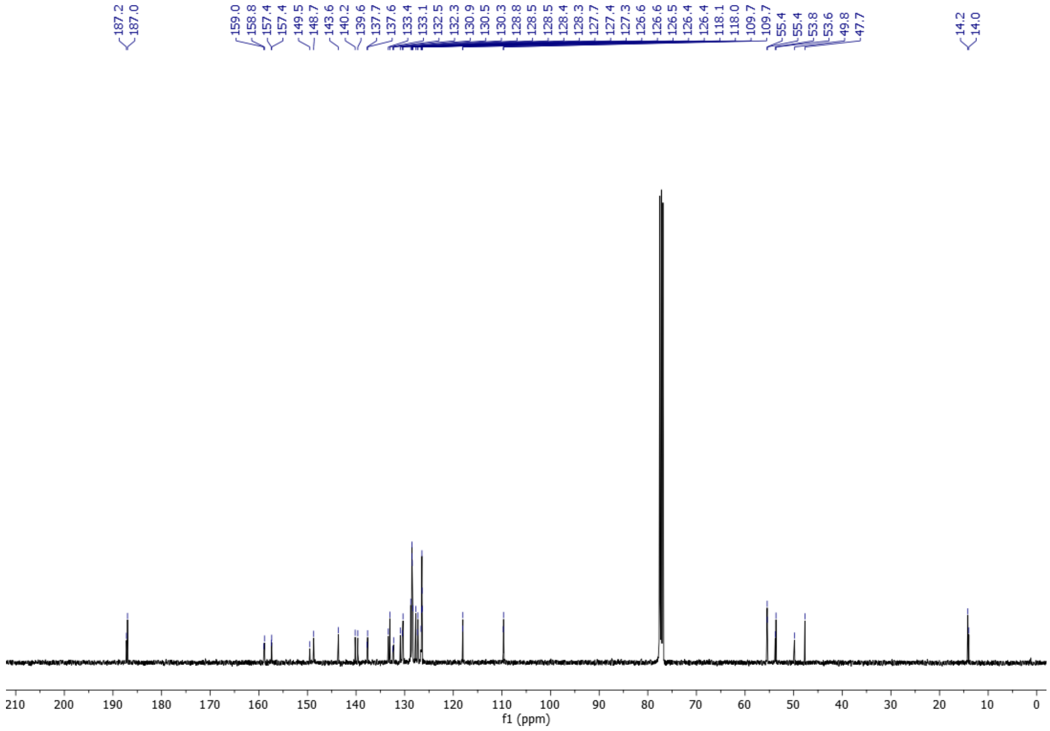

Compound **4e** ^{1}H NMR

(400 MHz, $CDCl_3$)

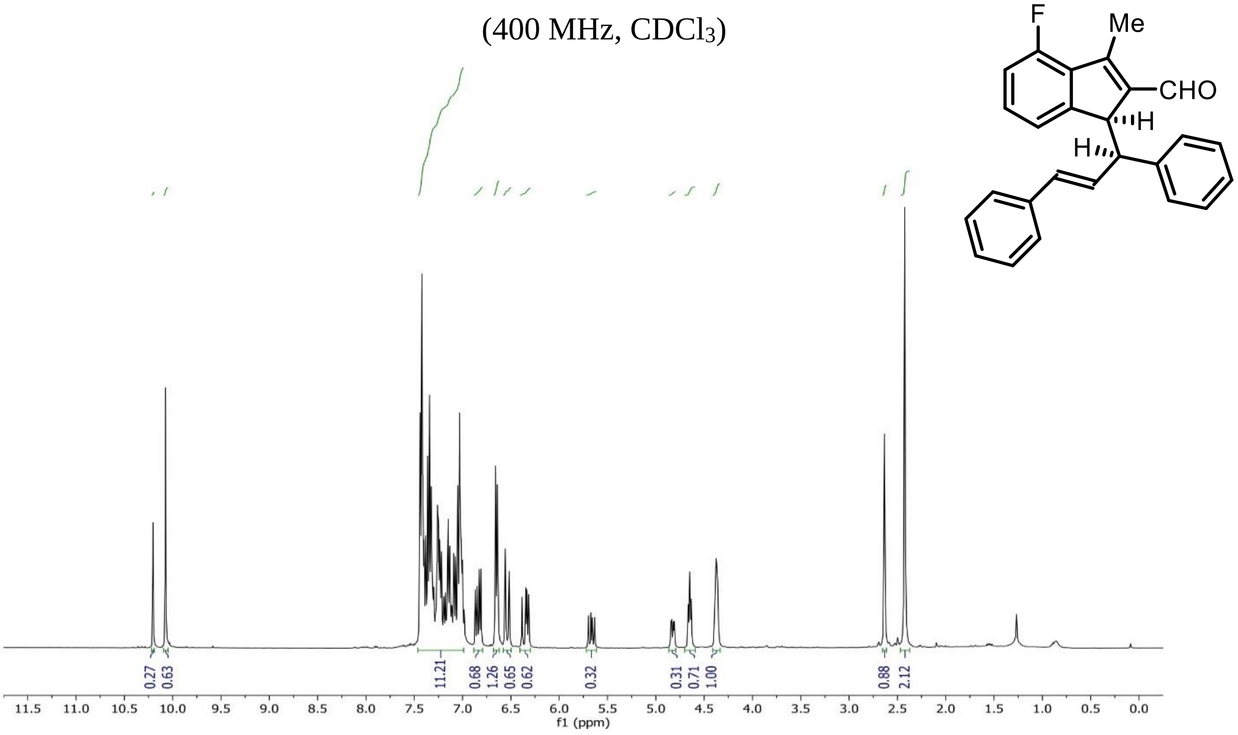

Compound **4e** ^{13}C NMR

(100 MHz, $CDCl_3$)

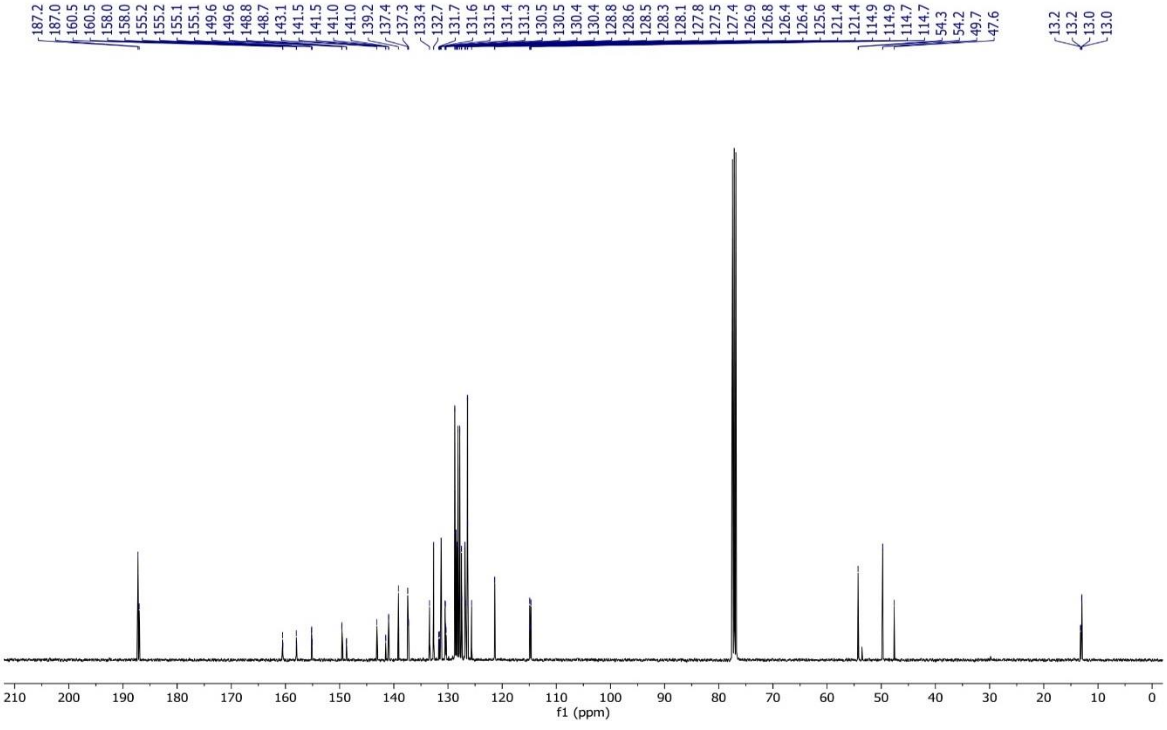

Compound **4e** ^{19}F NMR

(375 MHz, $CDCl_3$)

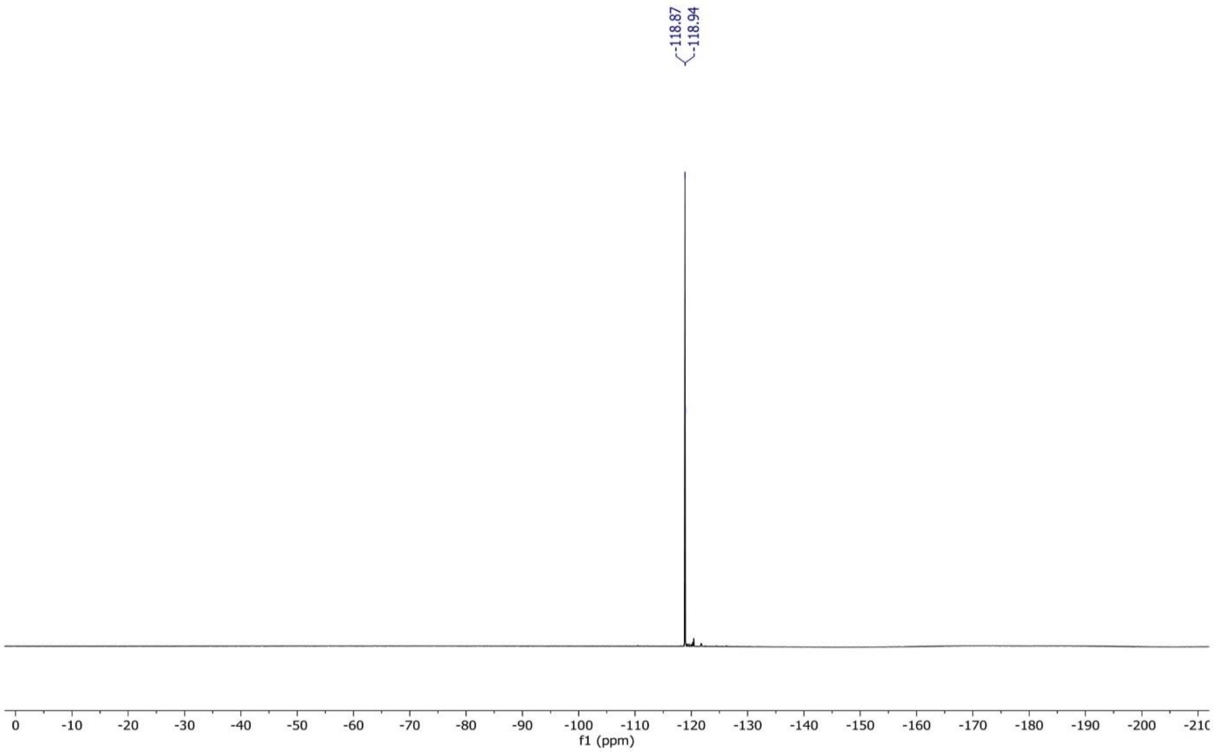

Compound **5e** ^{1}H NMR

(400 MHz, $CDCl_3$)

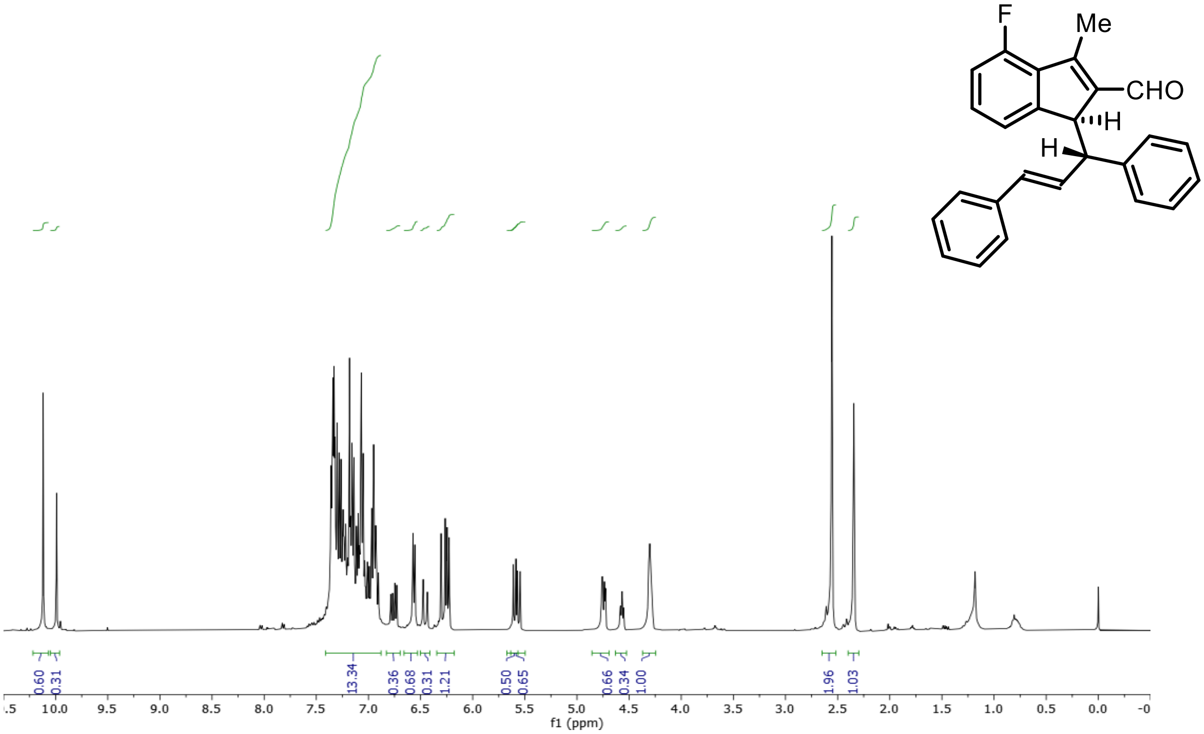

Compound **5e** ^{13}C NMR

(100 MHz, $CDCl_3$)

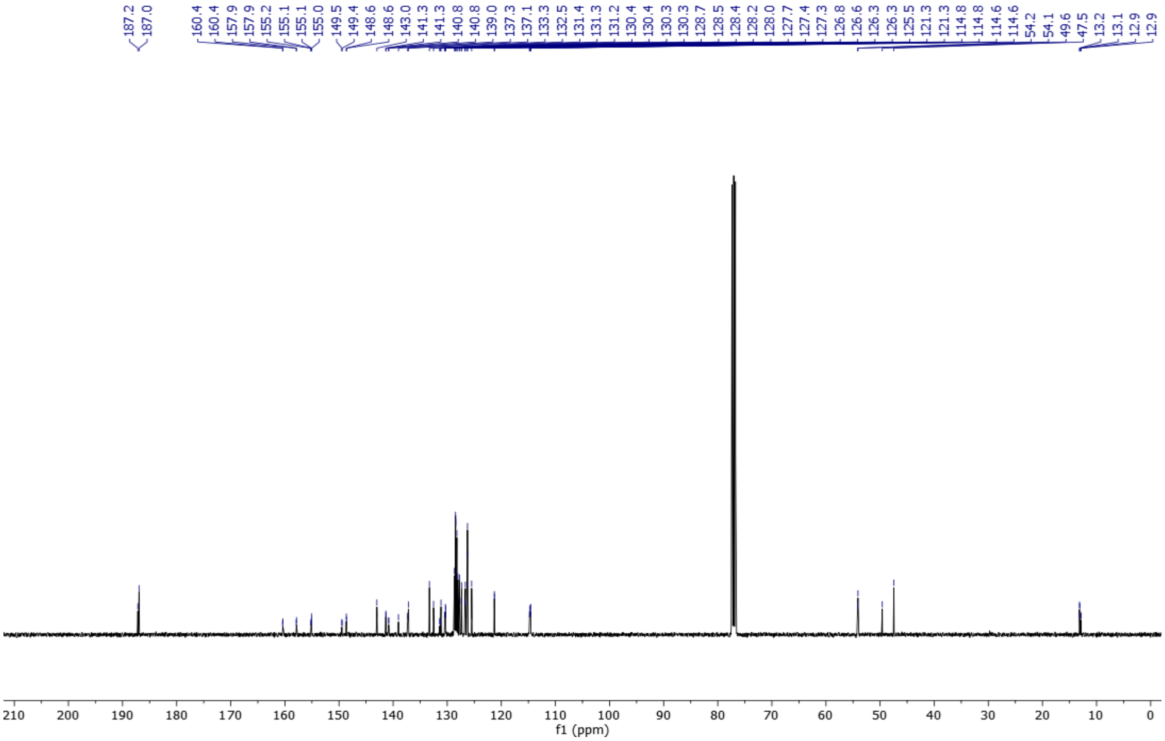

Compound **5e** ^{19}F NMR

(375 MHz, $CDCl_3$)

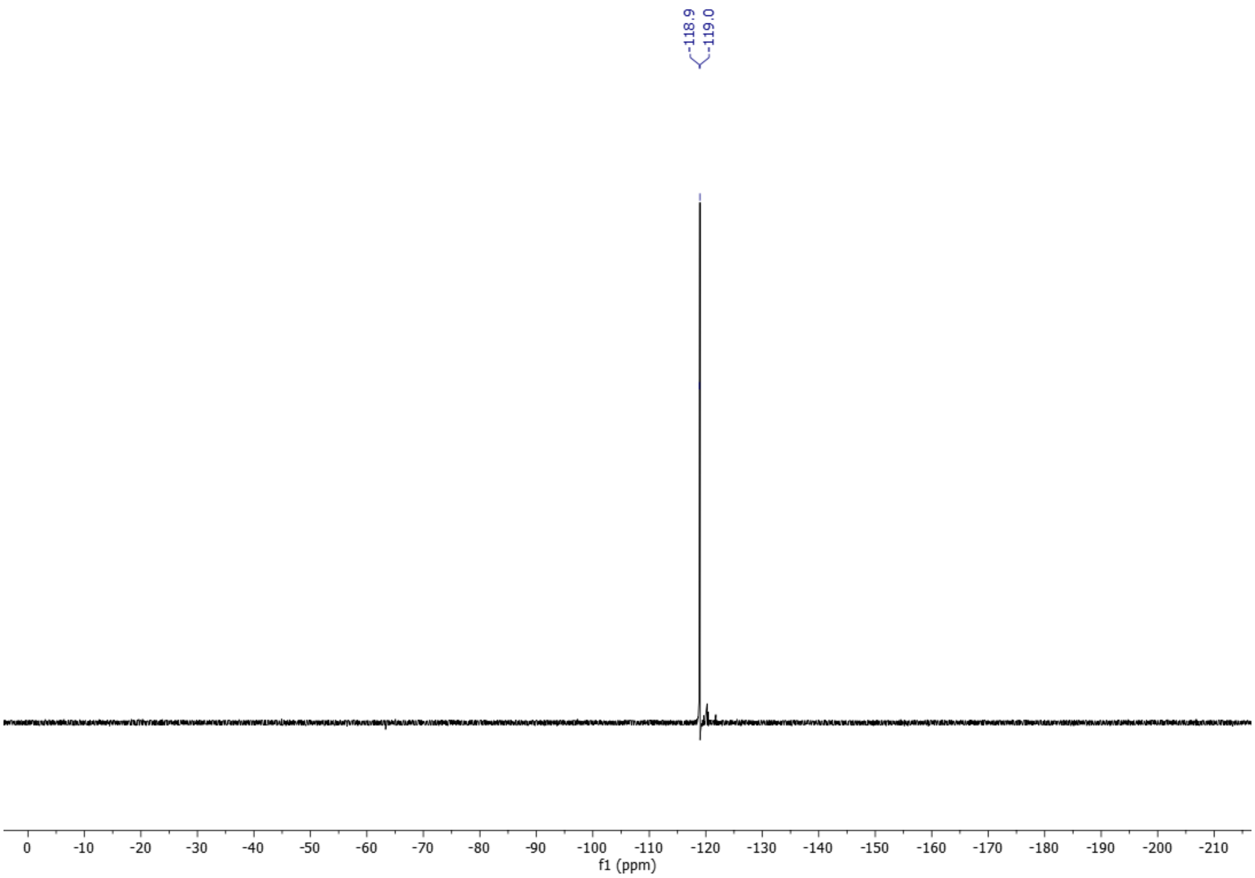

Compound **4f** ^{1}H NMR

(400 MHz, $CDCl_3$)

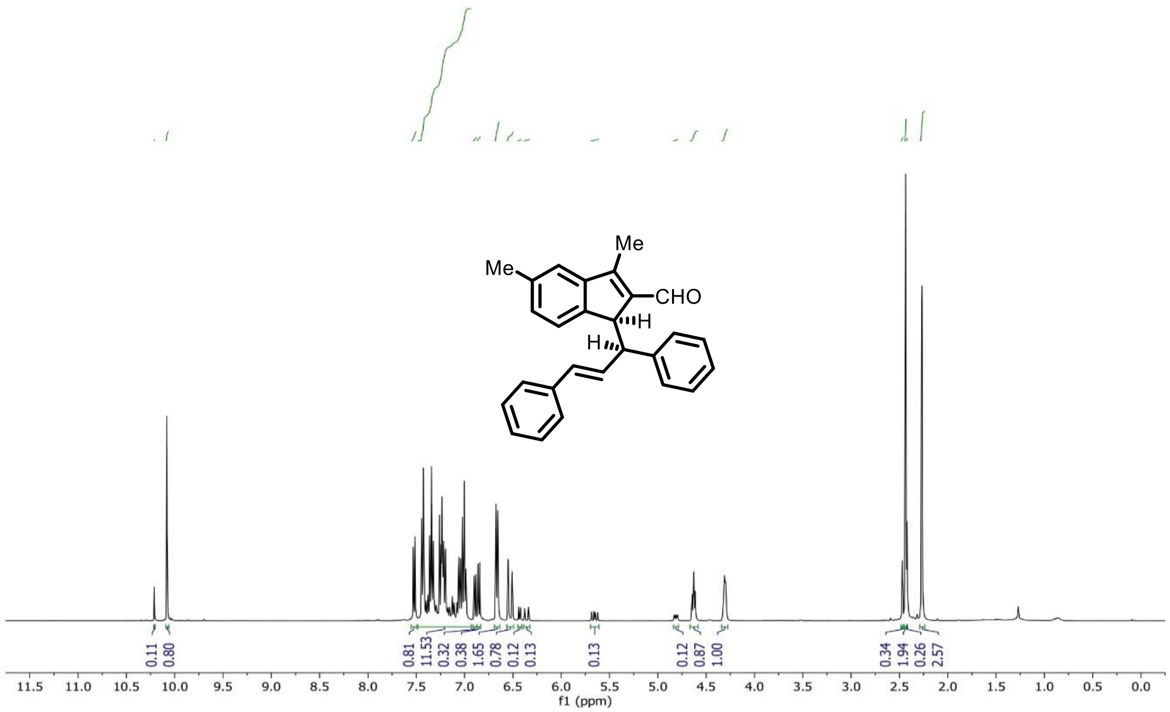

Compound **4f** ^{13}C NMR

(100 MHz, $CDCl_3$)

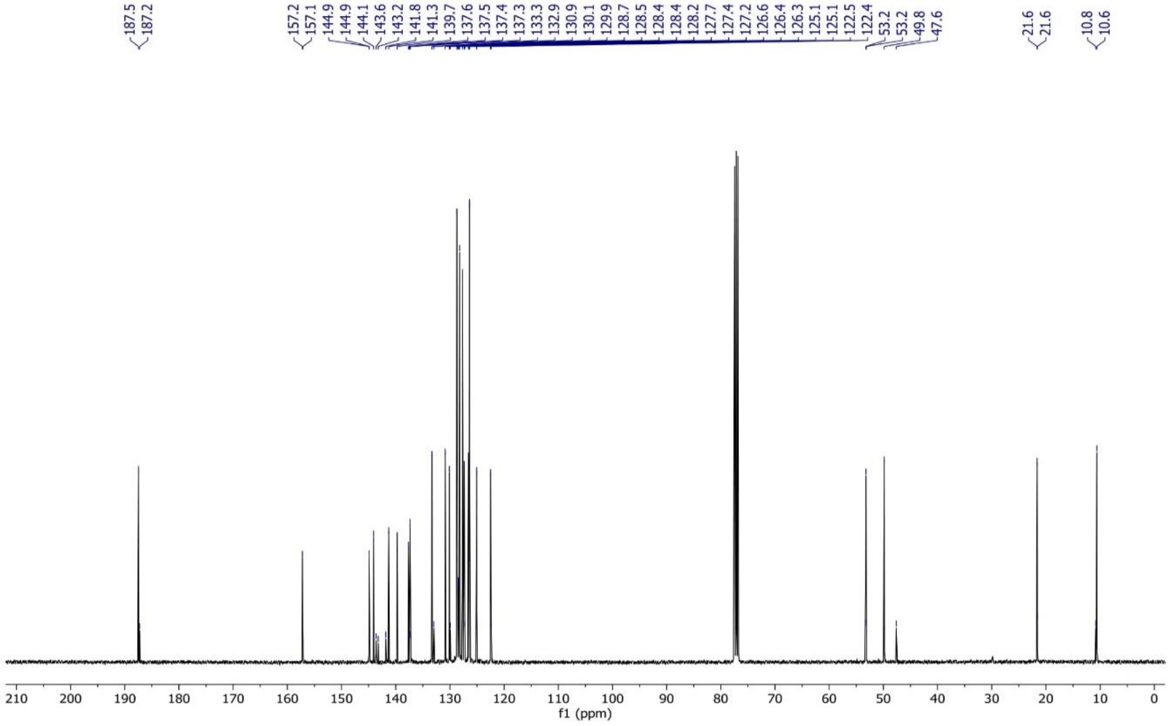

Compound **5f** ^{1}H NMR

(400 MHz, $CDCl_3$)

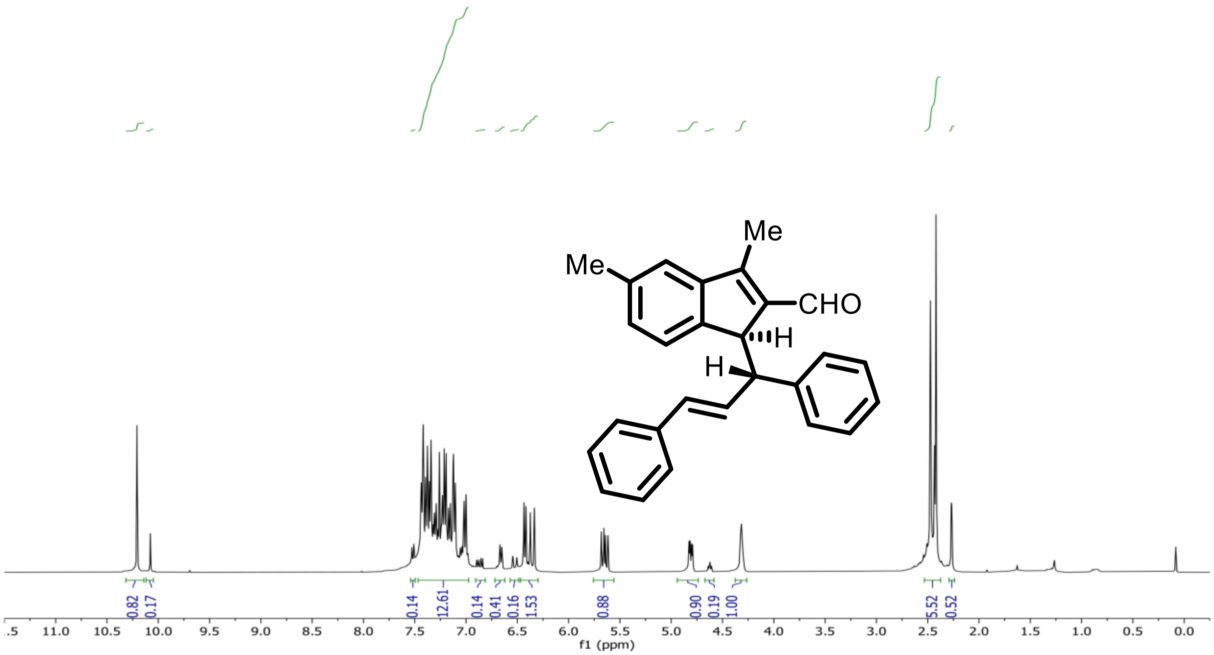

Compound **5f** ^{13}C NMR

(100 MHz, $CDCl_3$)

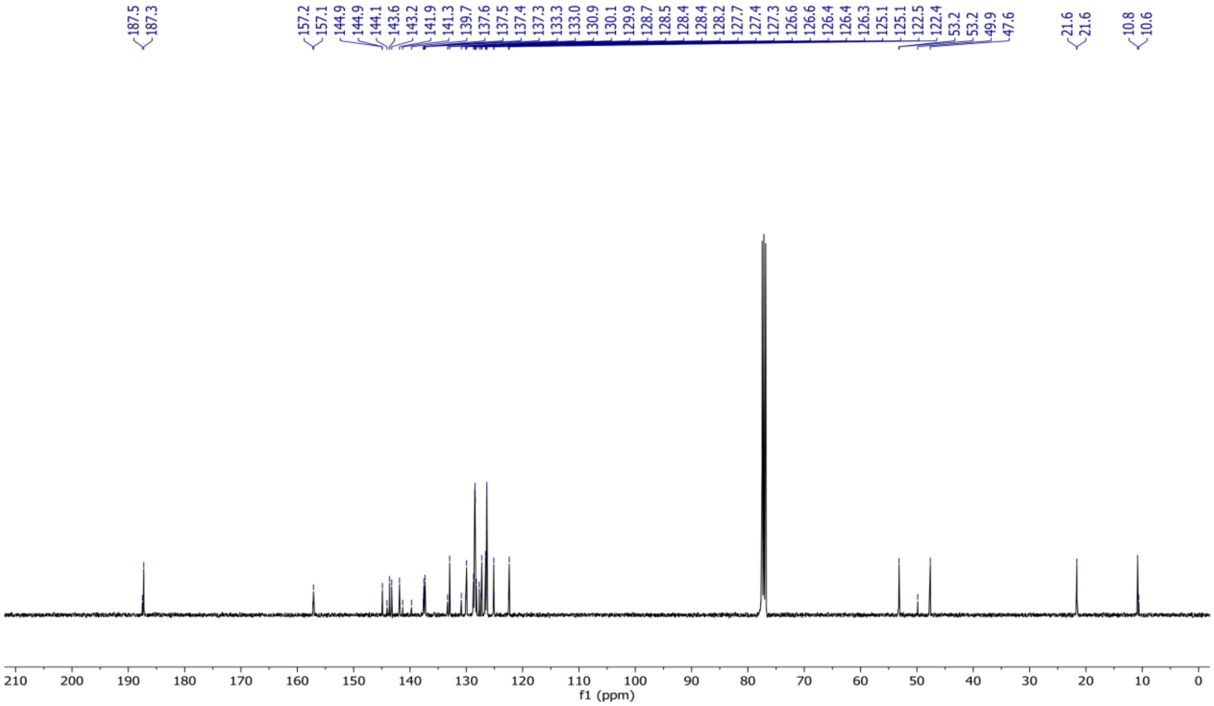

Compound **4g** ^{1}H NMR

(400 MHz, $CDCl_3$)

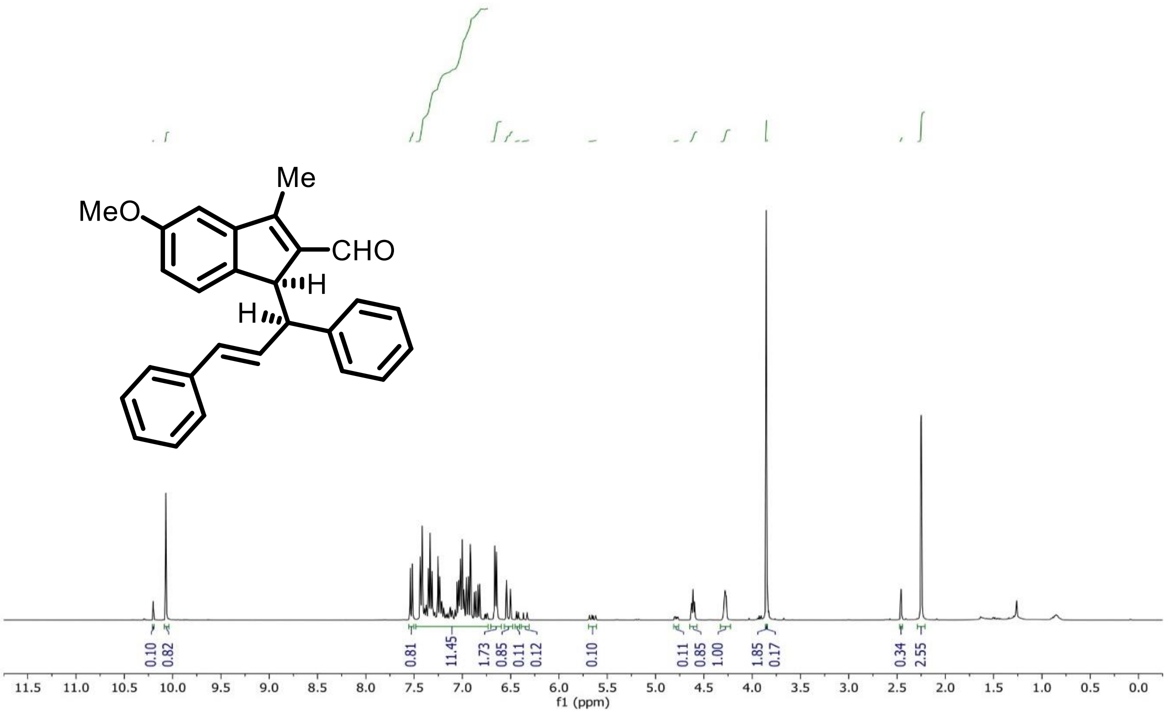

Compound **4g** ^{13}C NMR

(100 MHz, $CDCl_3$)

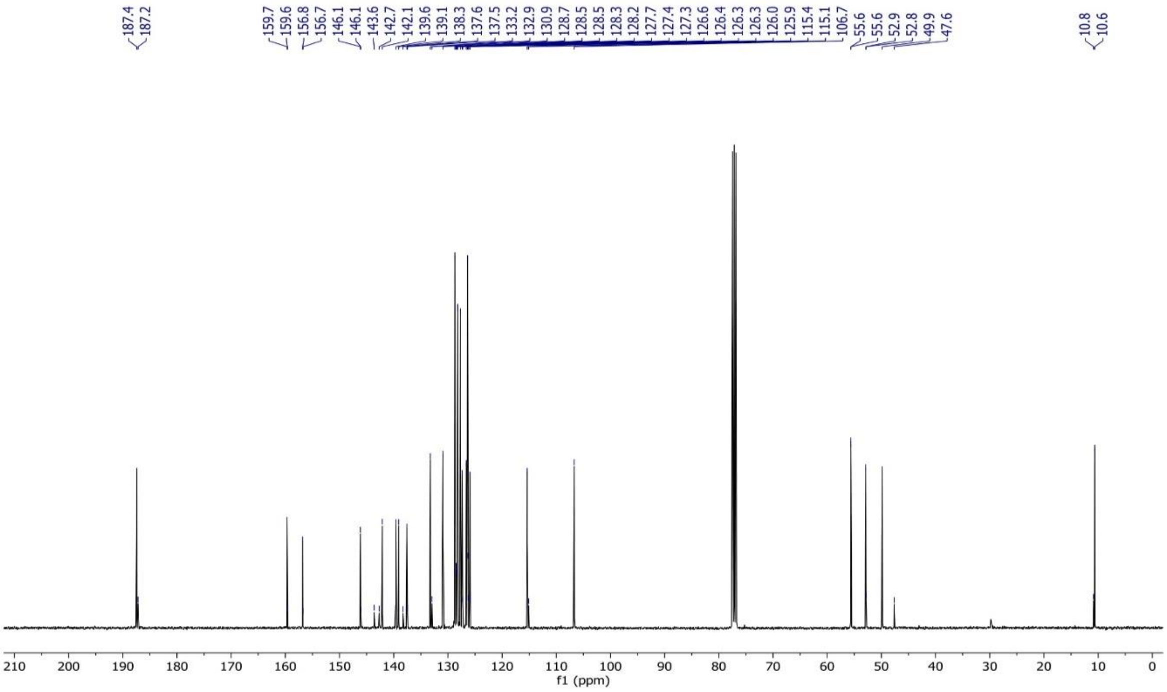

Compound **5g** ^{1}H NMR

(400 MHz, $CDCl_3$)

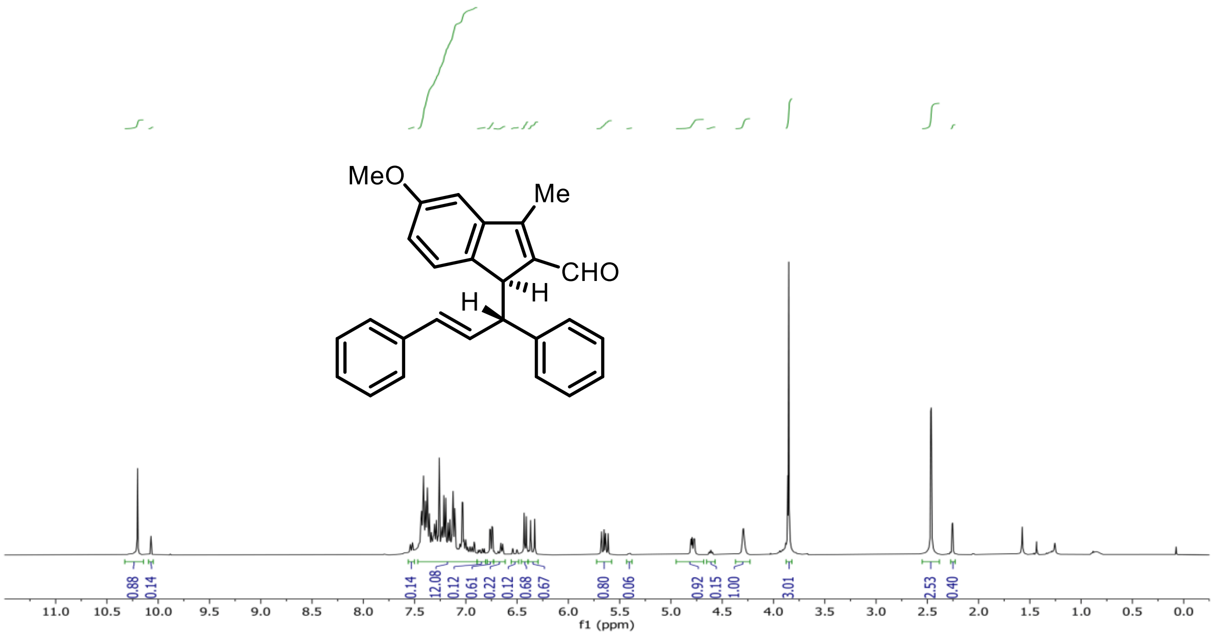

Compound **5g** ^{13}C NMR

(100 MHz, $CDCl_3$)

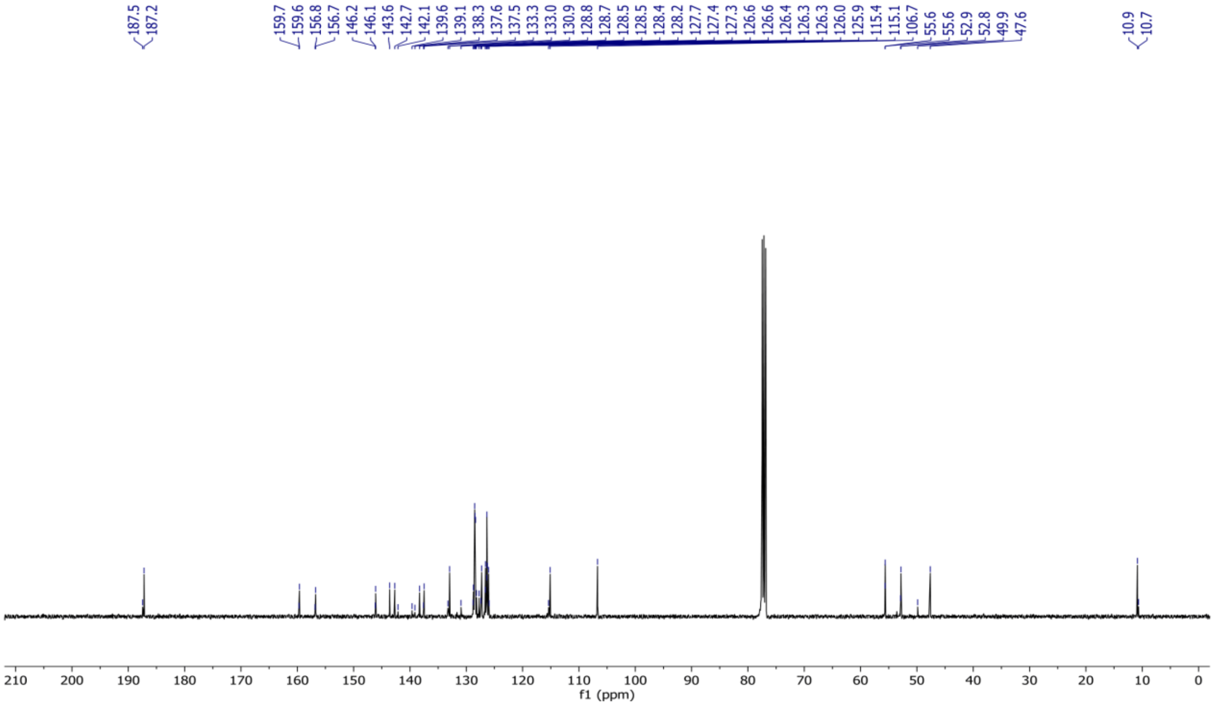

Compound **4h** ^{1}H NMR

(400 MHz, $CDCl_3$)

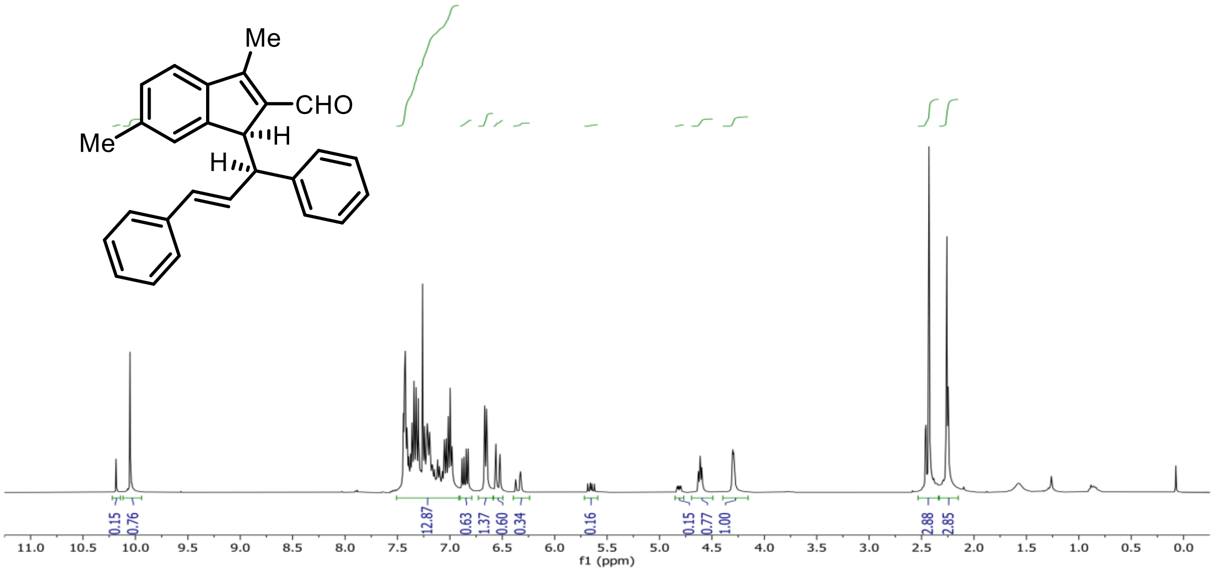

Compound **4h** ^{13}C NMR

(100 MHz, $CDCl_3$)

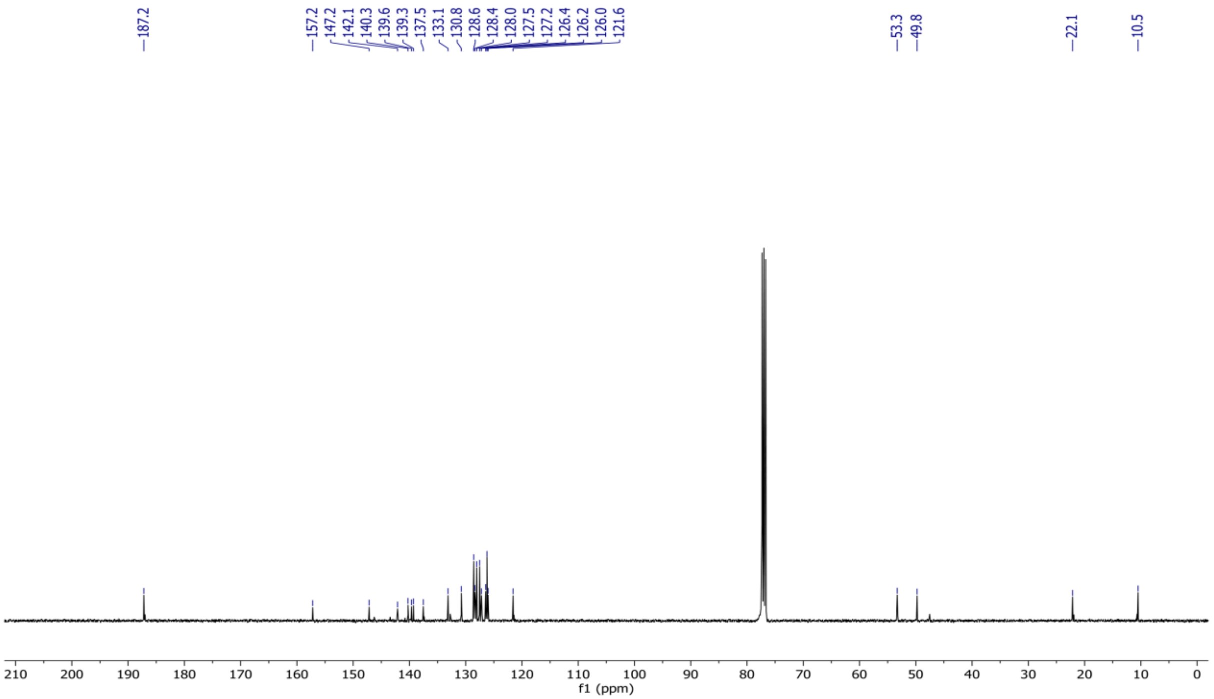

Compound **5h** ^{1}H NMR

(400 MHz, $CDCl_3$)

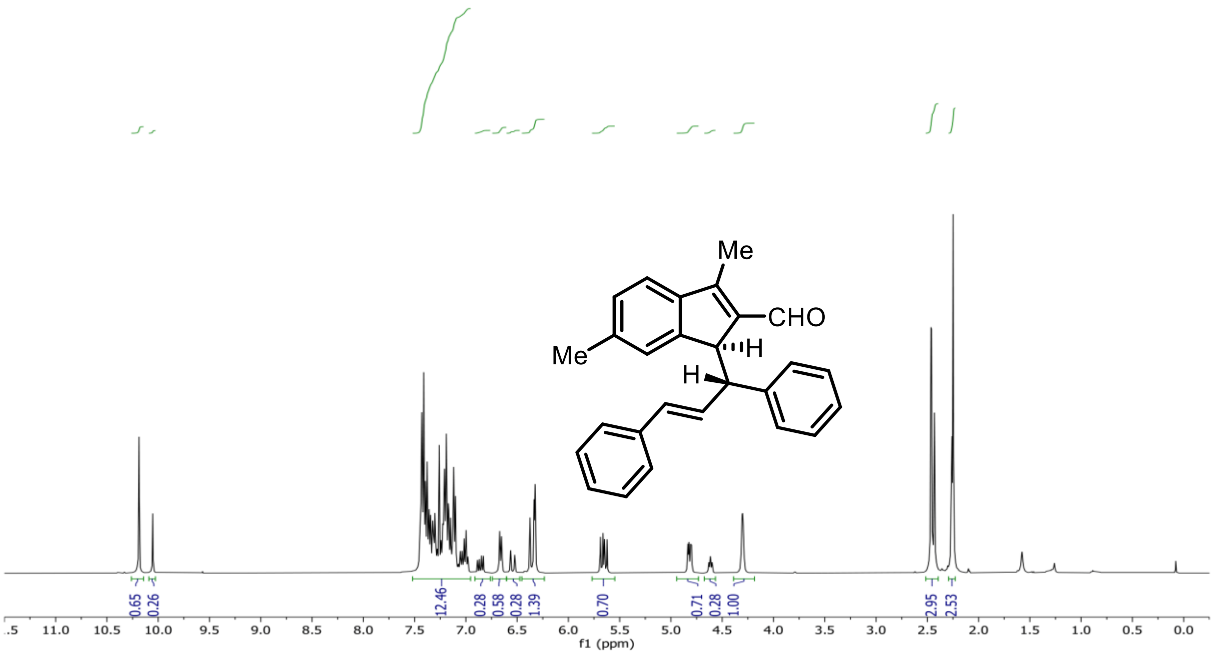

Compound **5h** ^{13}C NMR

(100 MHz, $CDCl_3$)

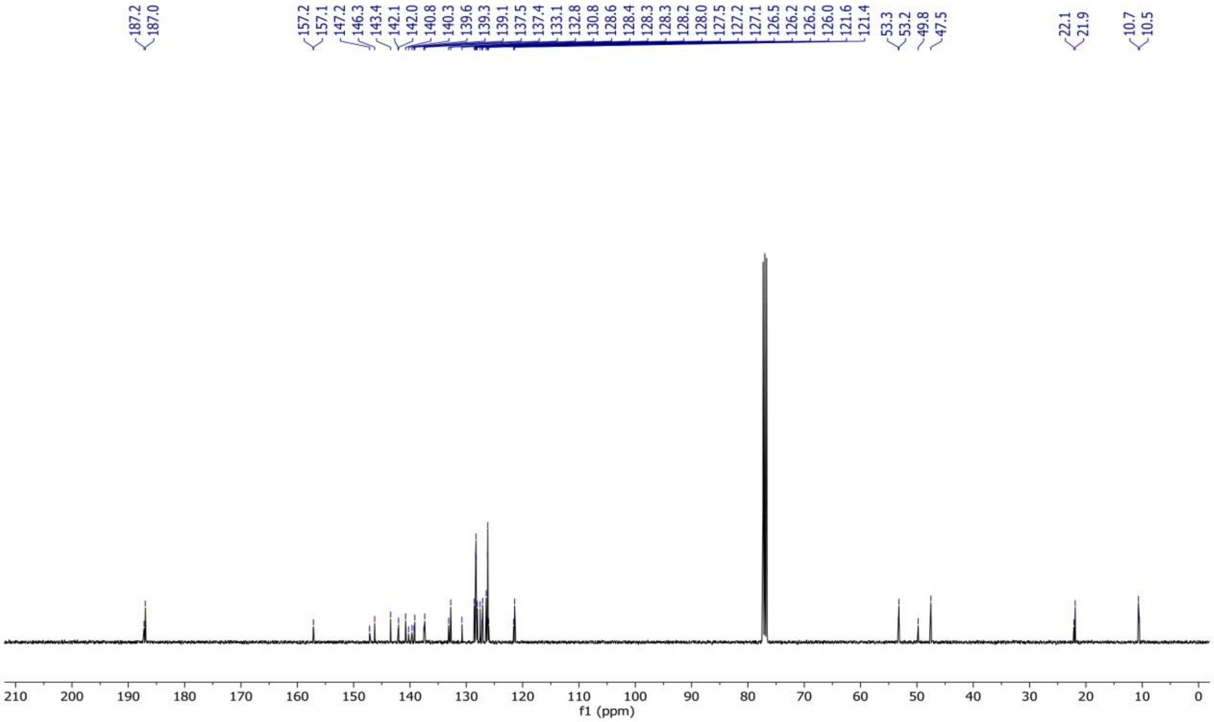

Compound **4i** ^{1}H NMR

(400 MHz, $CDCl_3$)

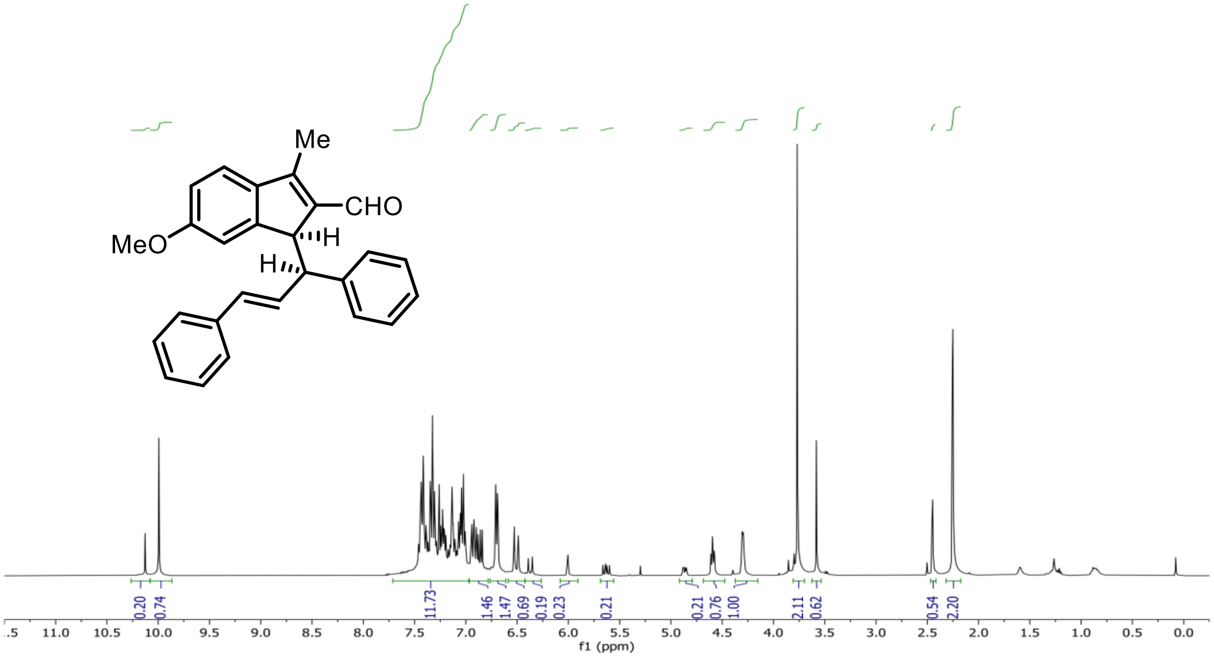

Compound **4i** ^{13}C NMR

(100 MHz, $CDCl_3$)

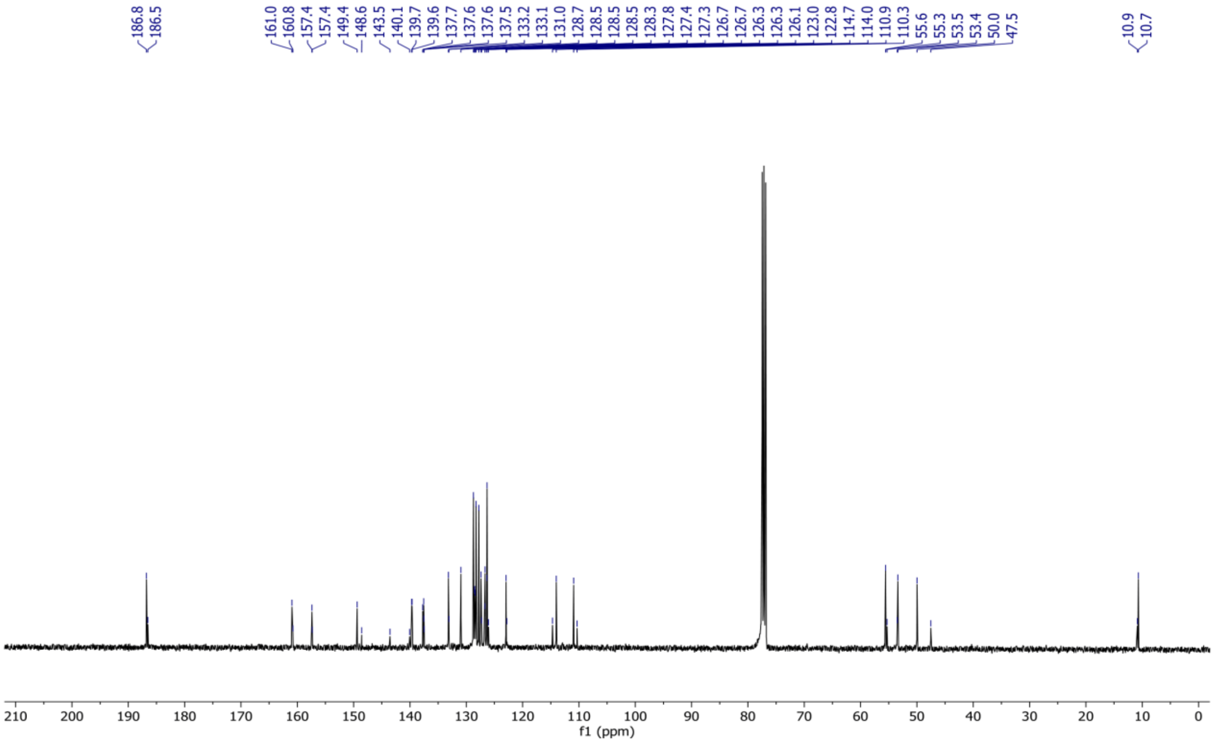

Compound **5i** ^{1}H NMR

(400 MHz, $CDCl_3$)

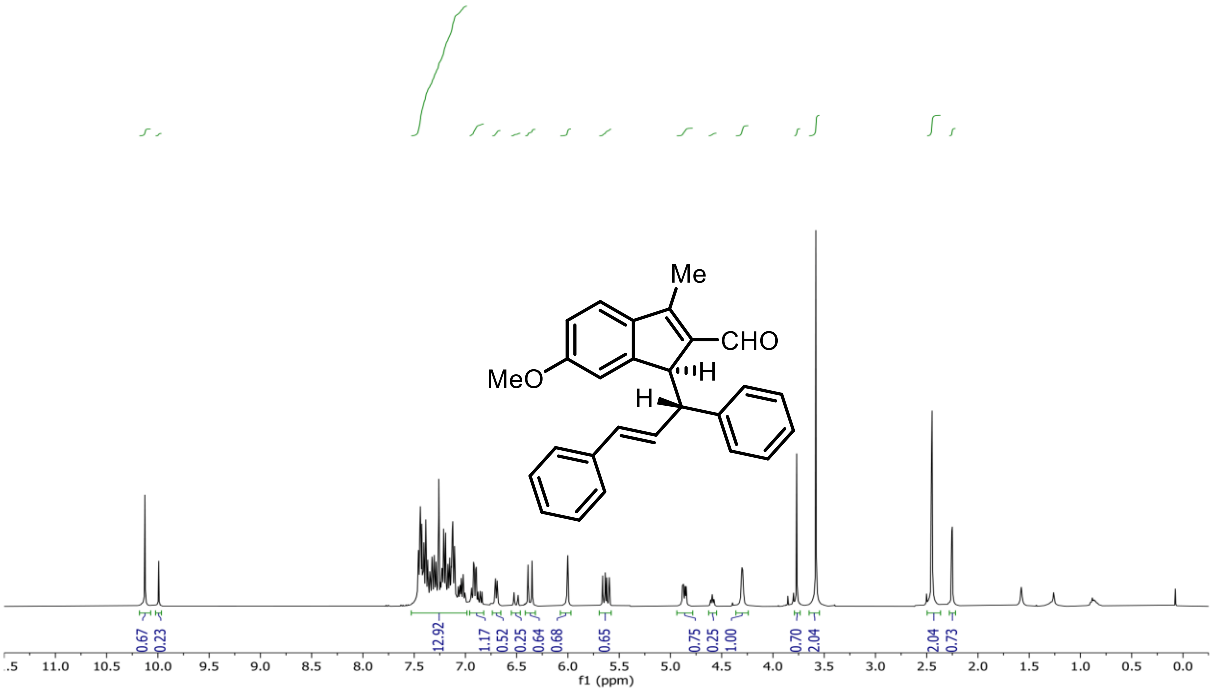

Compound **5i** ^{13}C NMR

(100 MHz, $CDCl_3$)

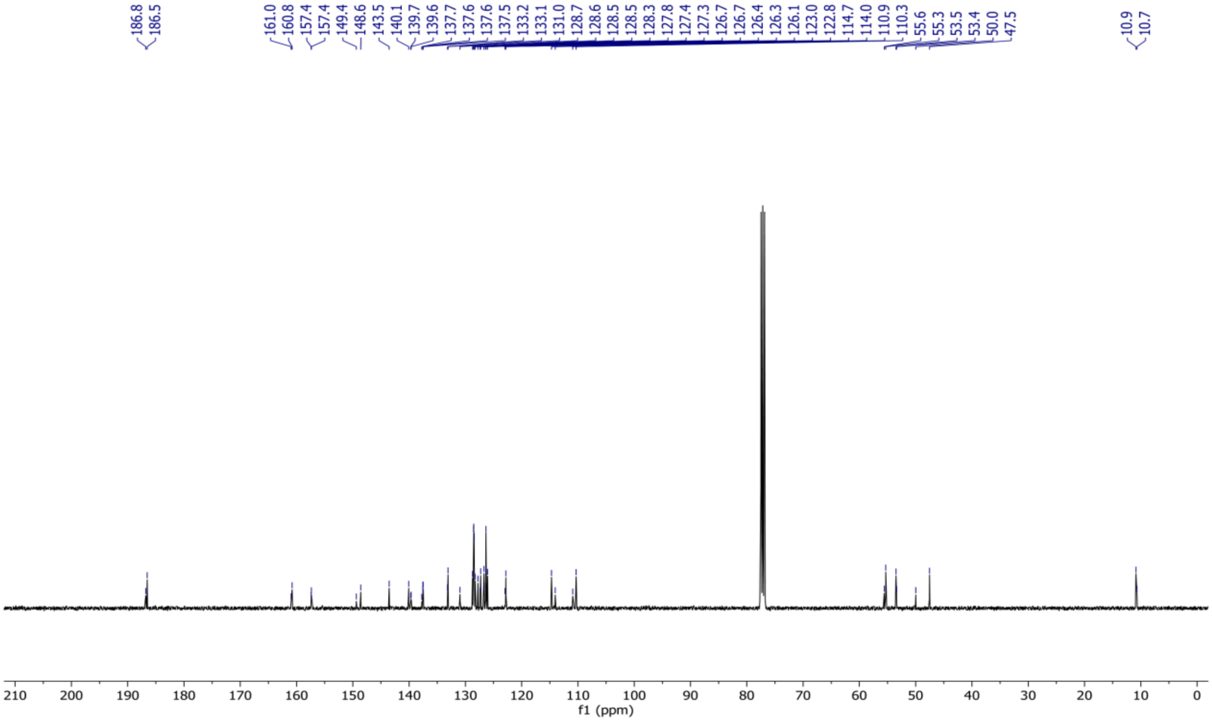

Compound **4j** ^{1}H NMR

(400 MHz, $CDCl_3$)

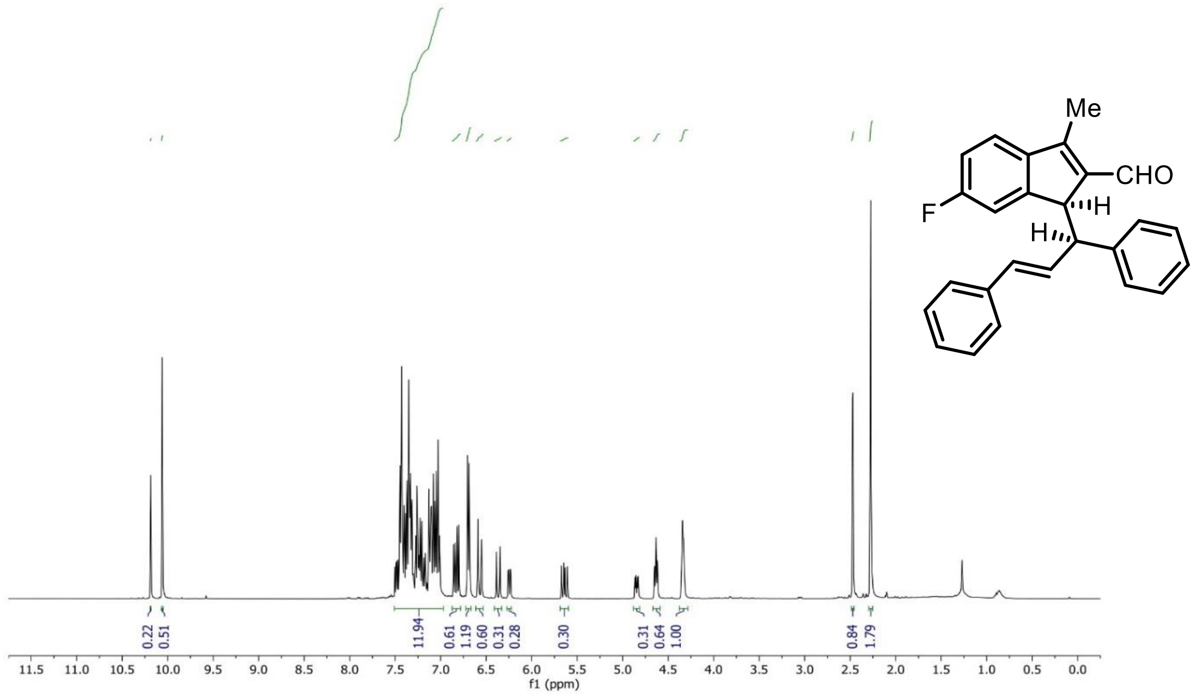

Compound **4j** ^{13}C NMR

(100 MHz, $CDCl_3$)

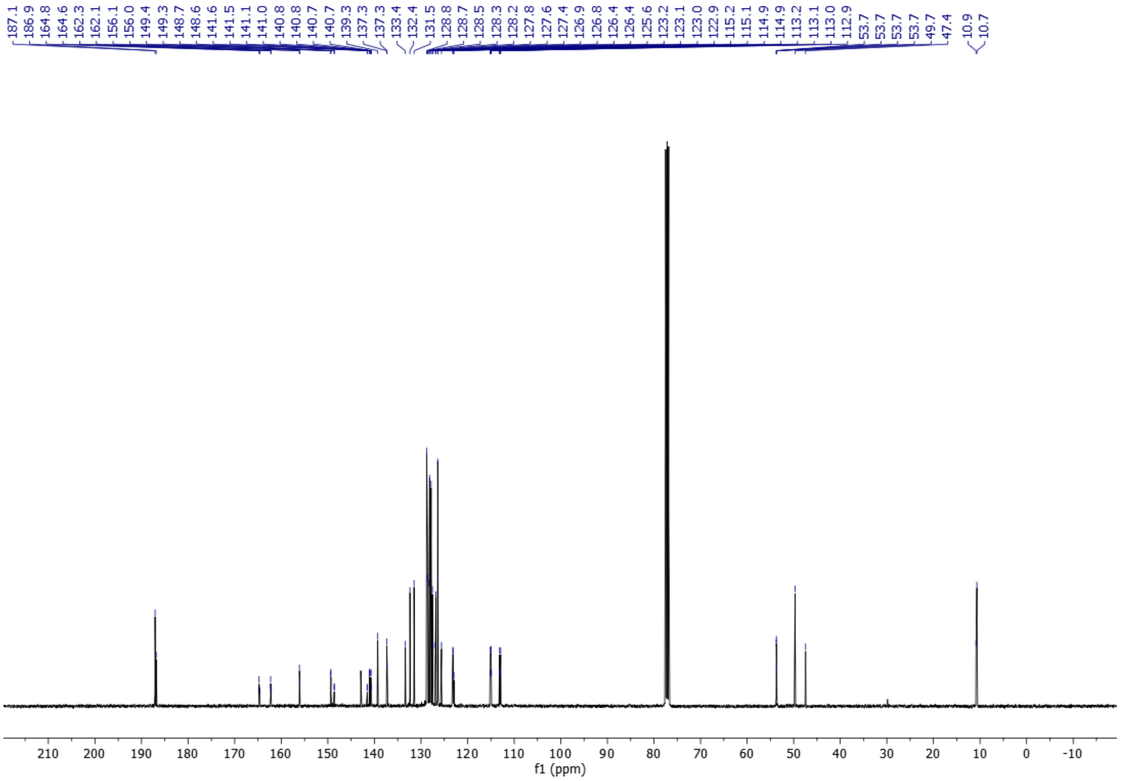

Compound **4j** ^{19}F NMR

(375 MHz, $CDCl_3$)

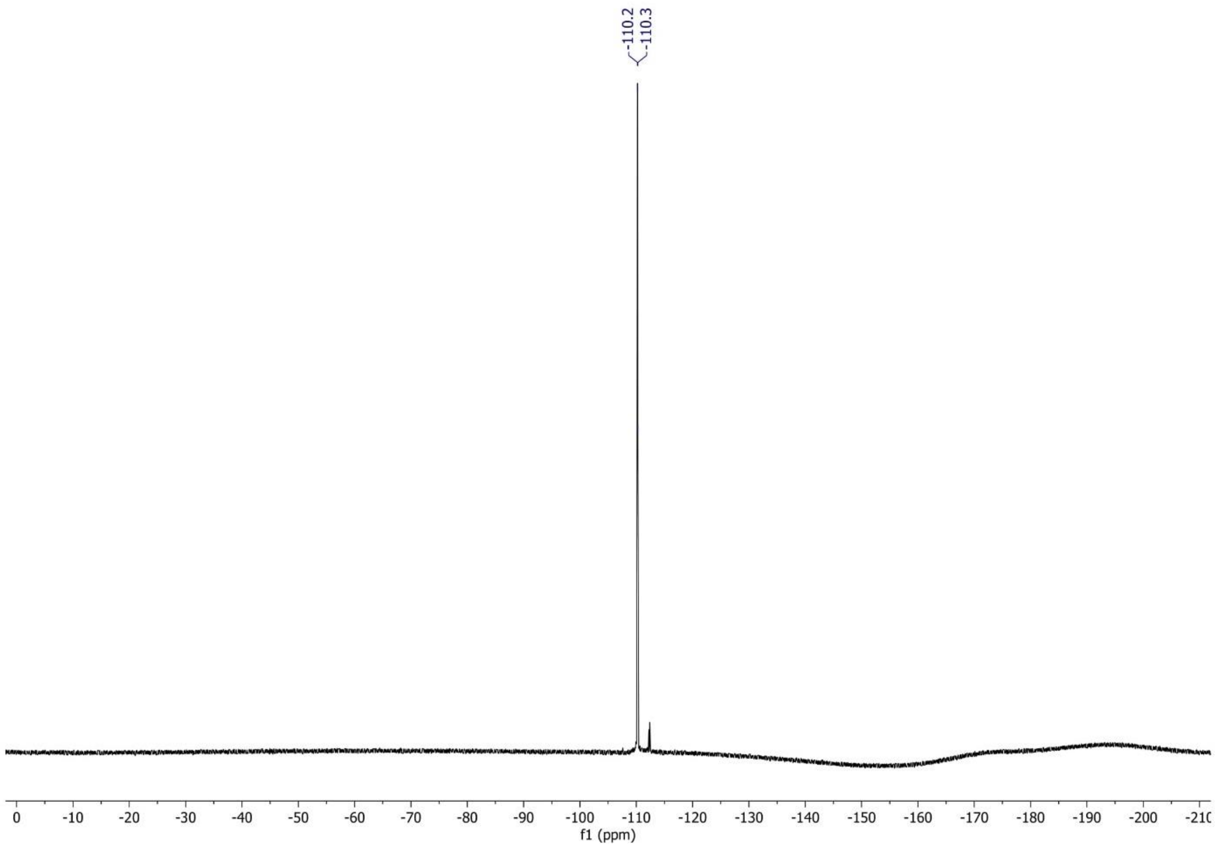

Compound **5j** ^{1}H NMR

(400 MHz, $CDCl_3$)

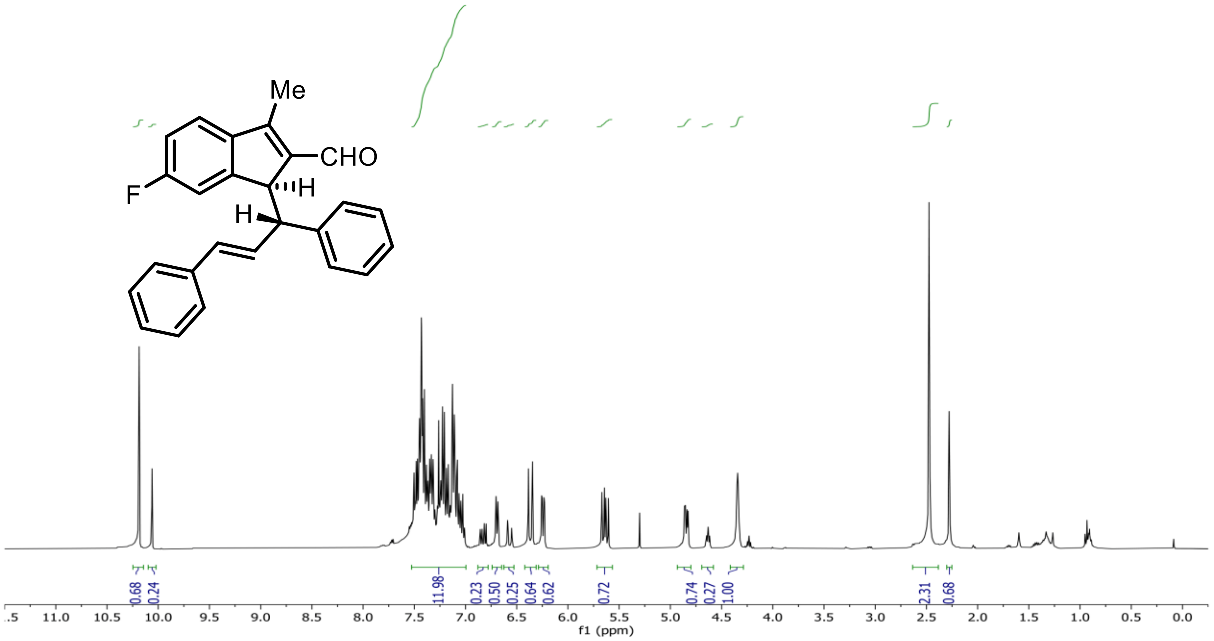

Compound **5j** ^{13}C NMR

(100 MHz, $CDCl_3$)

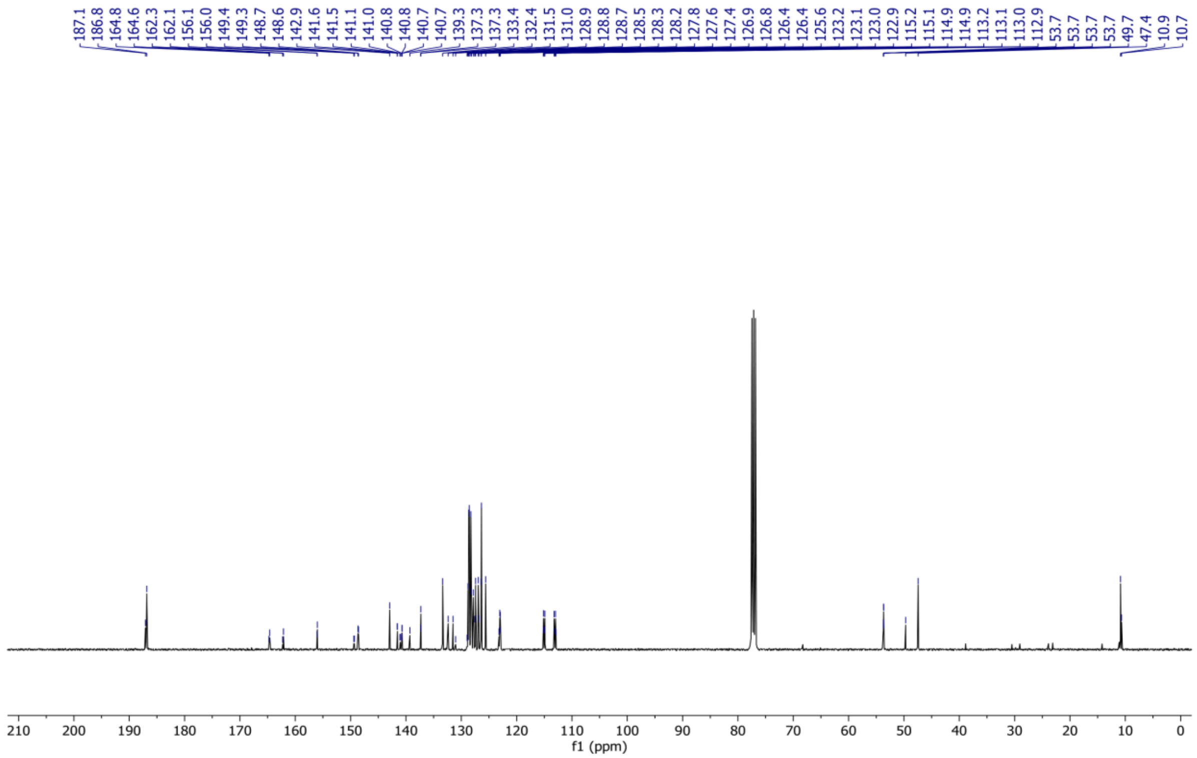

Compound **5j** ^{19}F NMR

(375 MHz, $CDCl_3$)

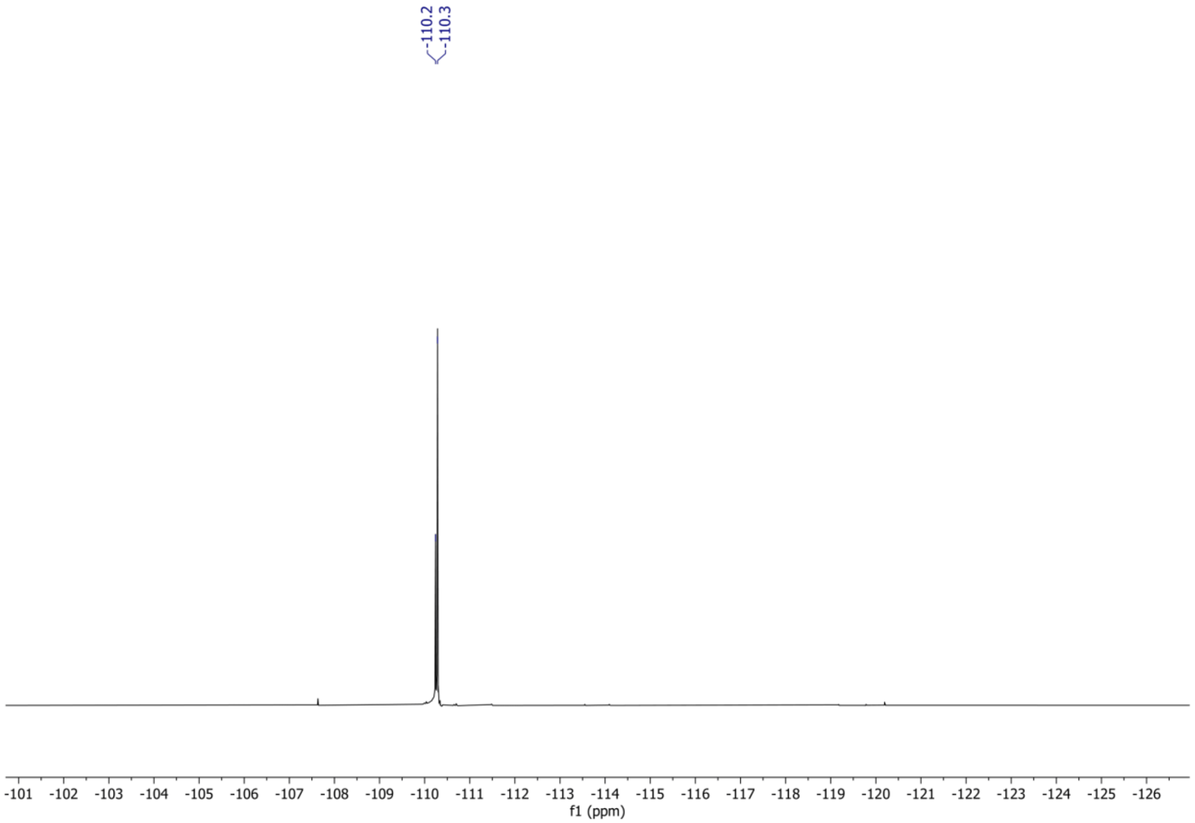

Compound **4k** ^{1}H NMR

(400 MHz, $CDCl_3$)

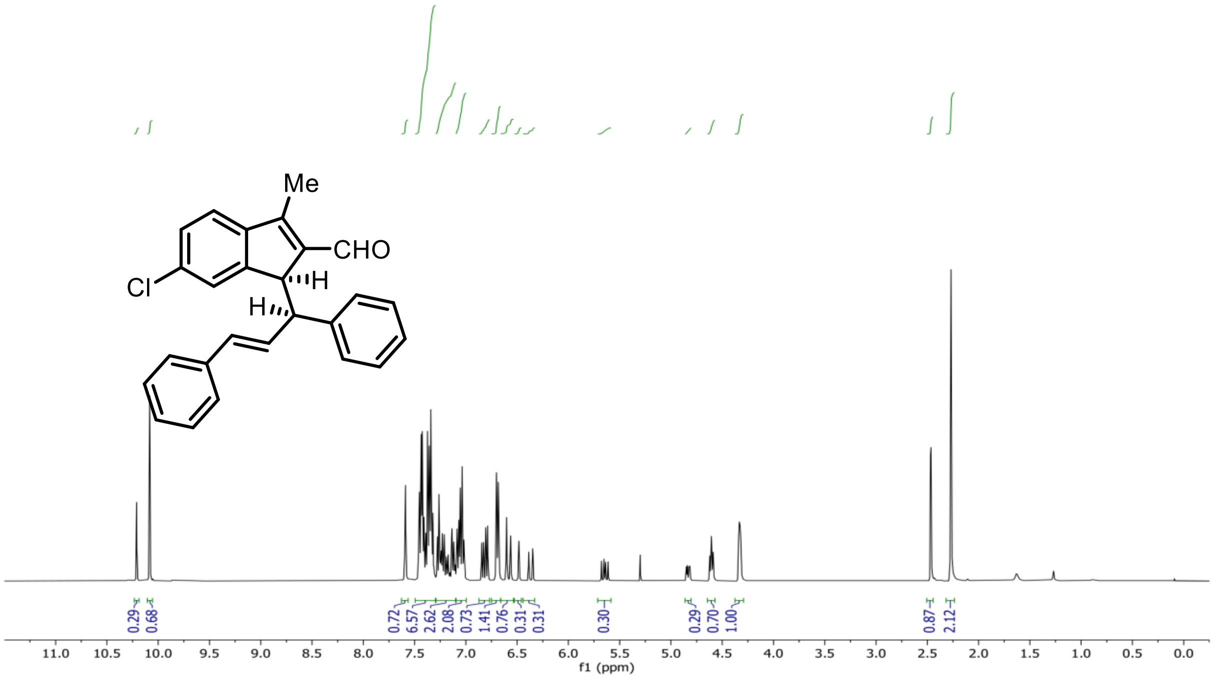

Compound **4k** ^{13}C NMR

(100 MHz, $CDCl_3$)

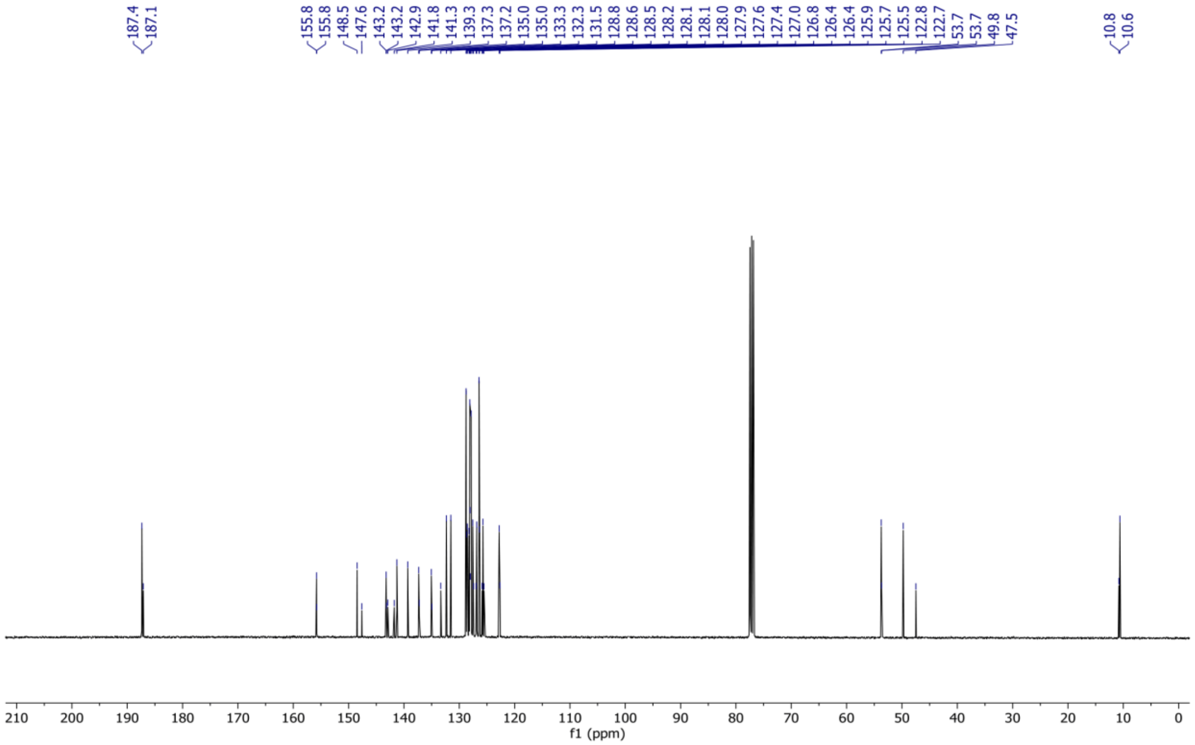

Compound **5k** ^{1}H NMR

(400 MHz, $CDCl_3$)

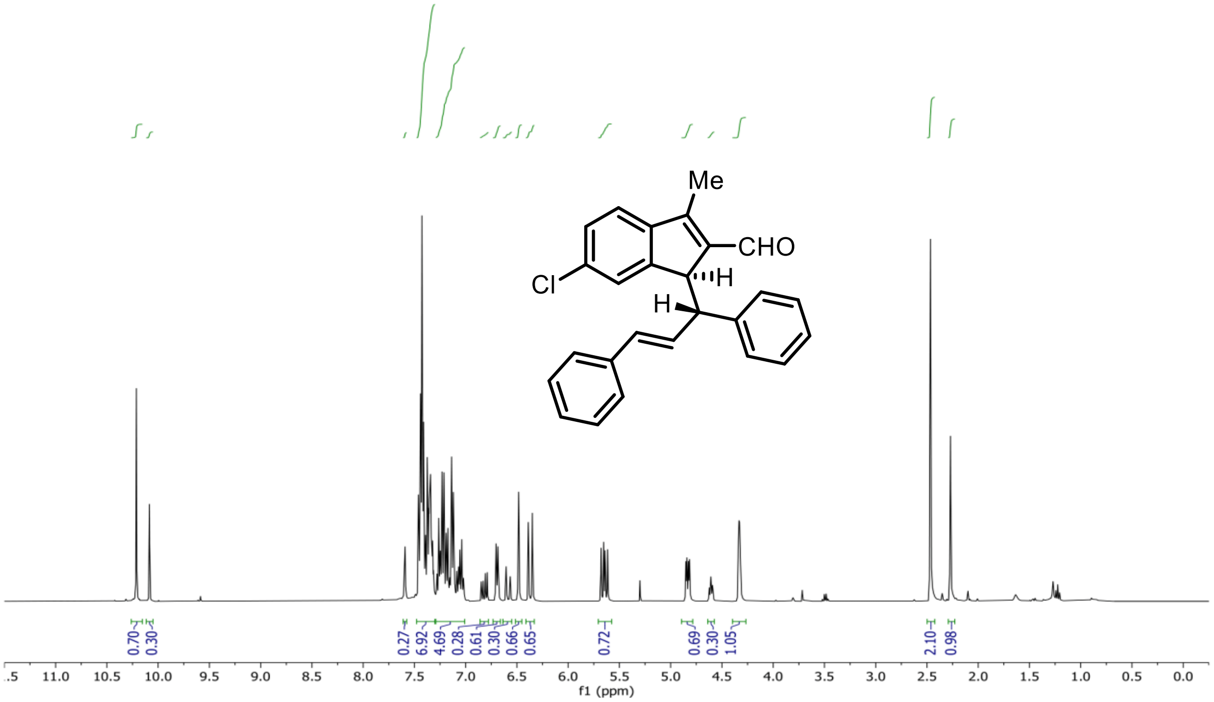

Compound **5k** ^{13}C NMR

(100 MHz, $CDCl_3$)

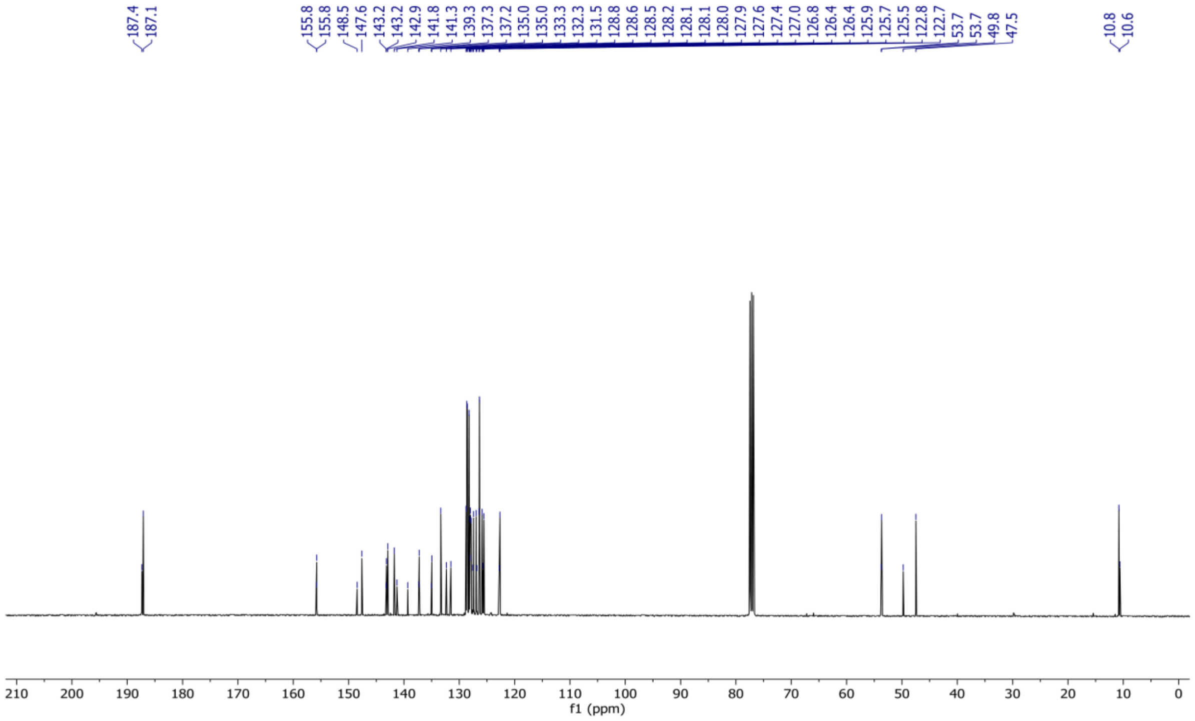

Compound **4l** ^{1}H NMR

(400 MHz, $CDCl_3$)

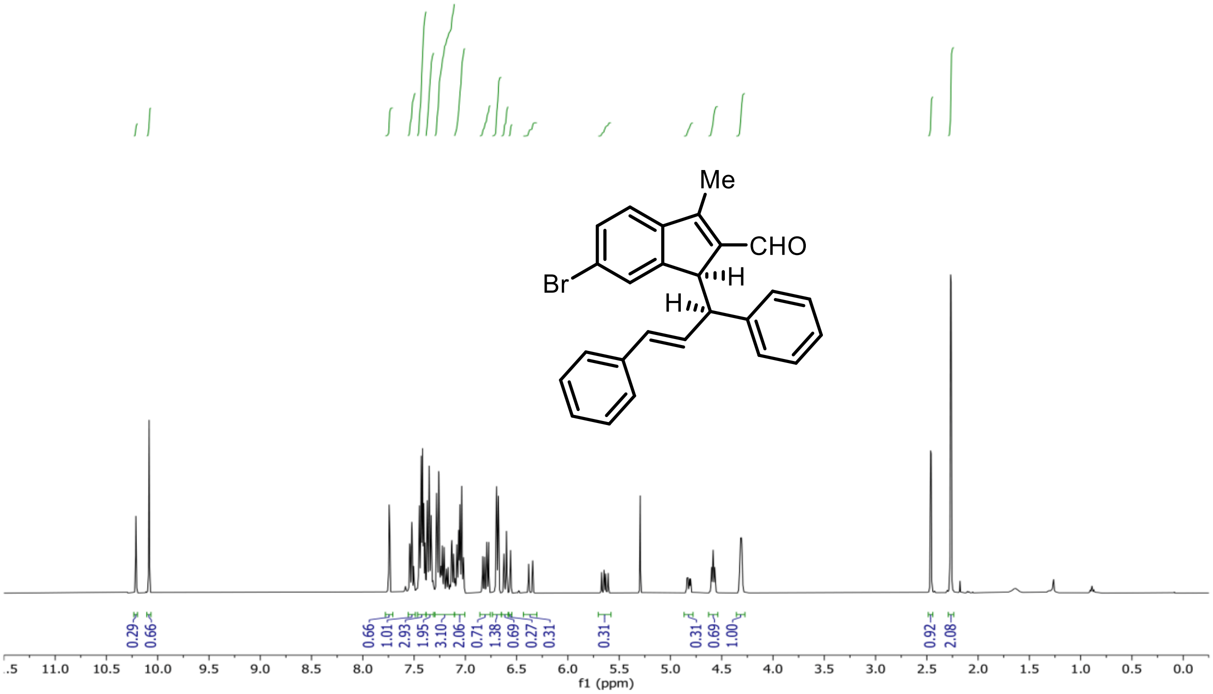

Compound **4l** ^{13}C NMR

(100 MHz, $CDCl_3$)

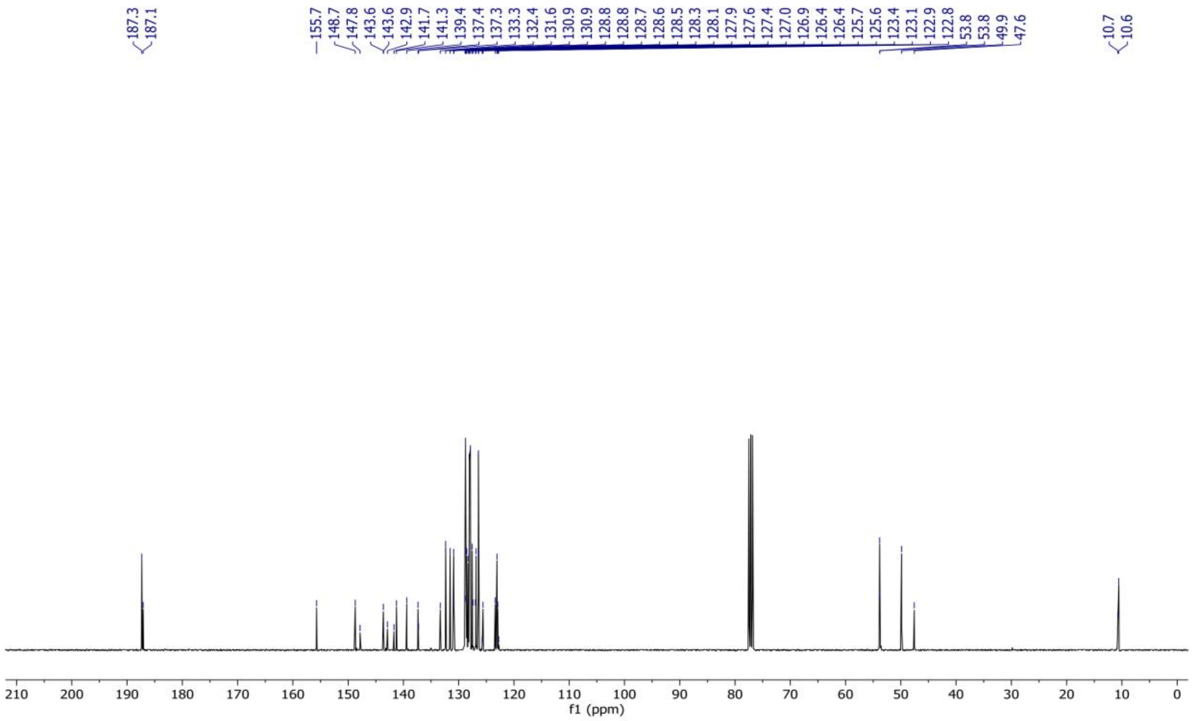

Compound **5l** ^{1}H NMR

(400 MHz, $CDCl_3$)

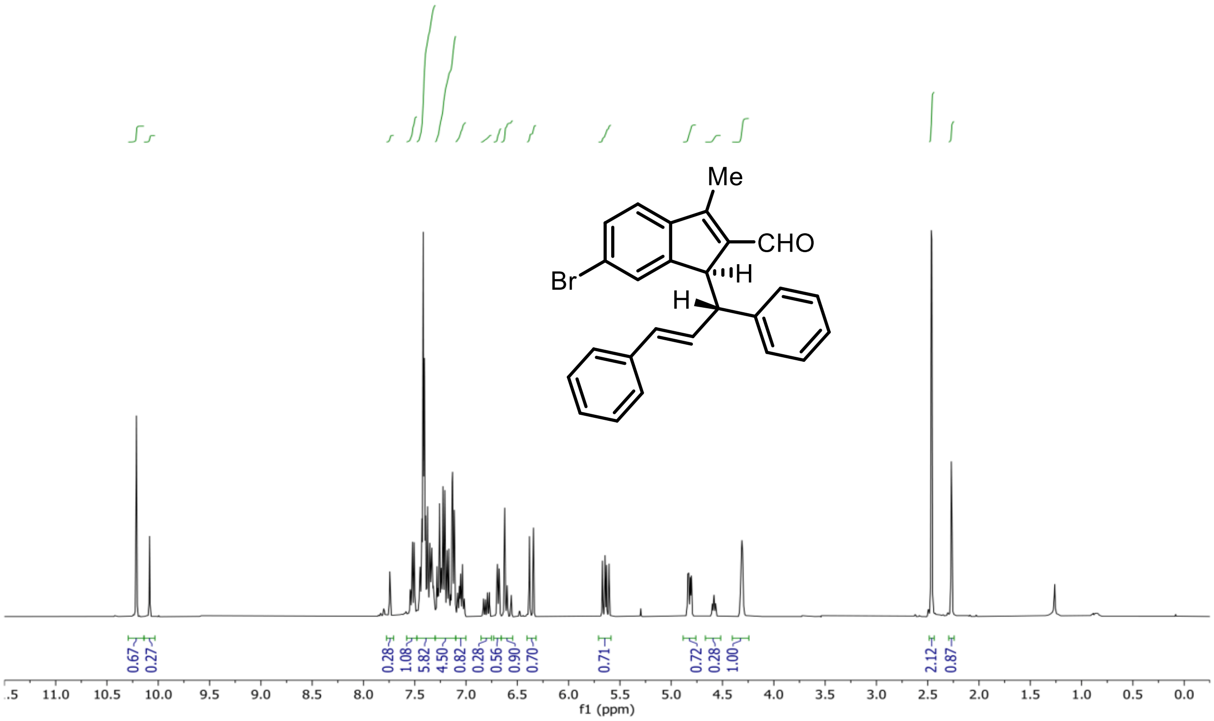

Compound **4l** ^{13}C NMR

(100 MHz, $CDCl_3$)

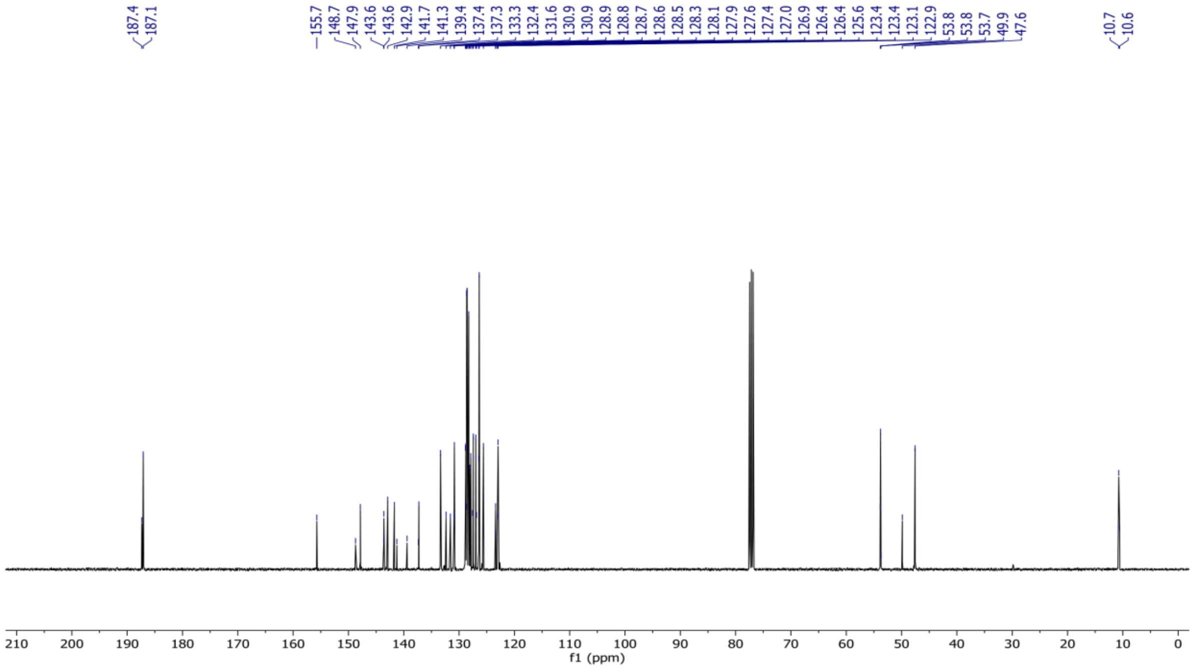

Compound **4m** ^{1}H NMR

(400 MHz, $CDCl_3$)

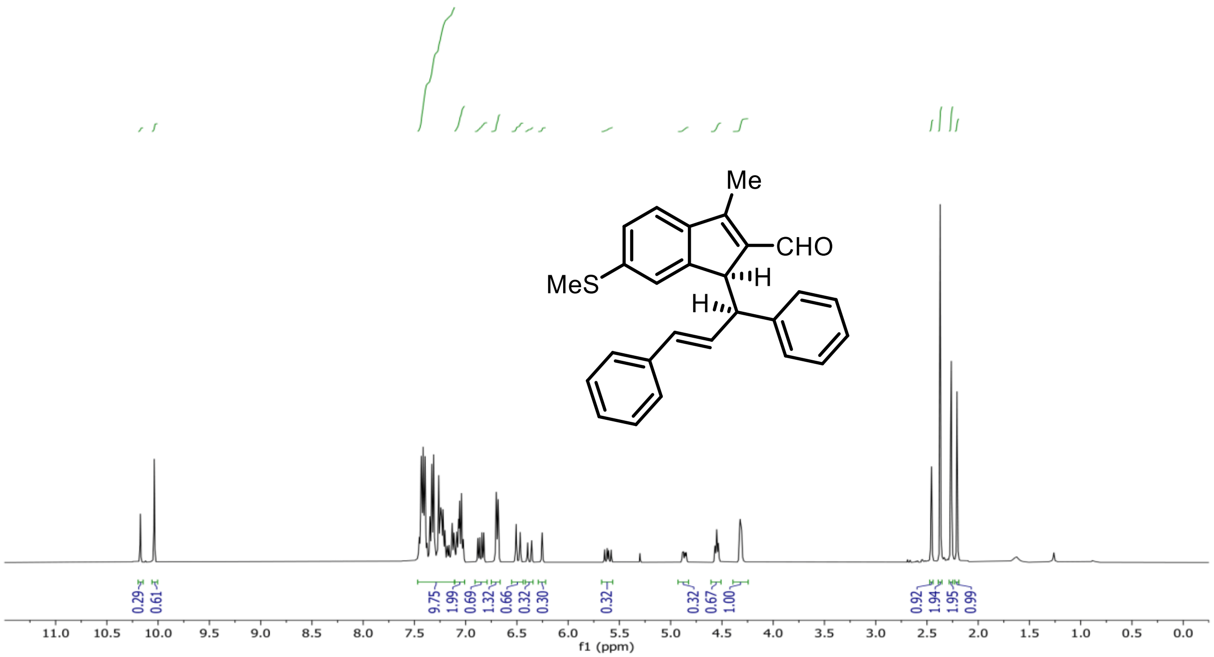

Compound **4m** ^{13}C NMR

(100 MHz, $CDCl_3$)

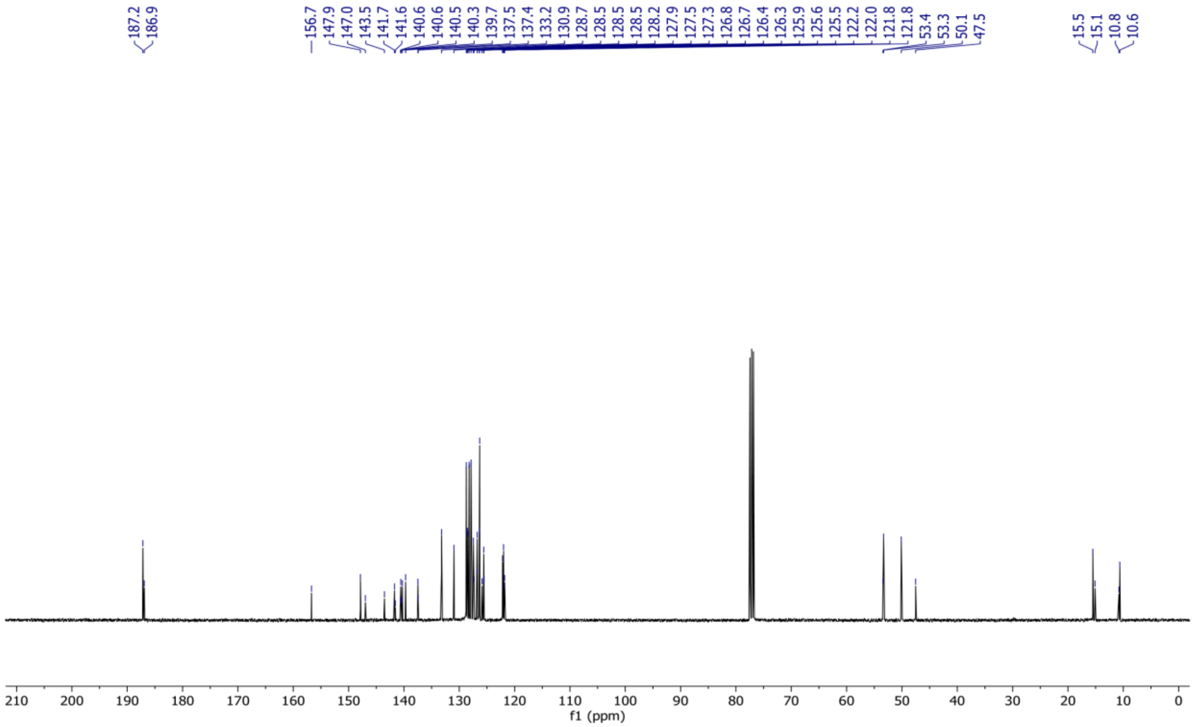

Compound **5m** ^{1}H NMR

(400 MHz, $CDCl_3$)

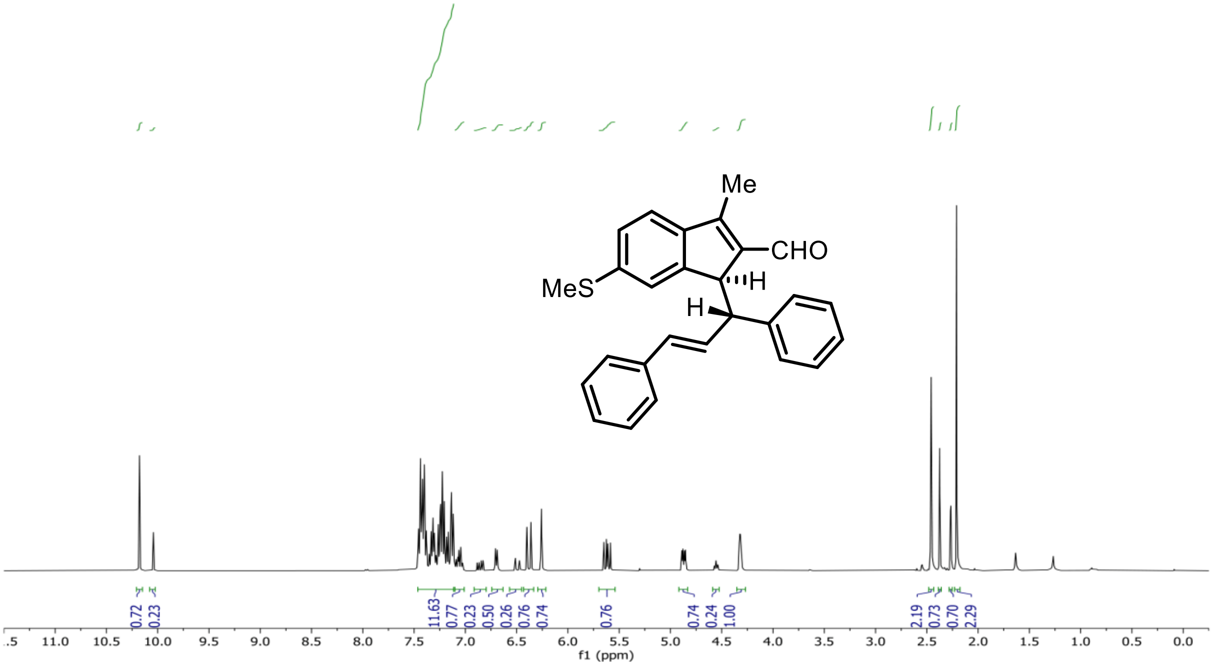

Compound **5m** ^{13}C NMR

(100 MHz, $CDCl_3$)

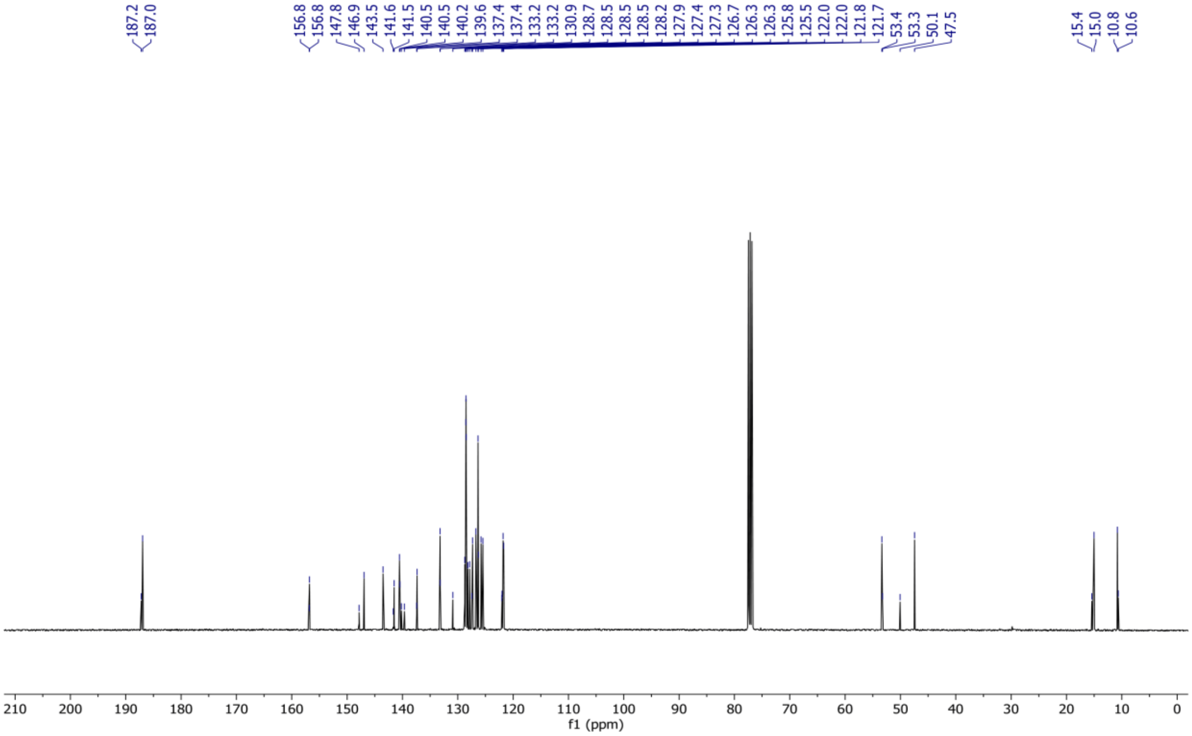

Compound **4n** ^{1}H NMR

(400 MHz, $CDCl_3$)

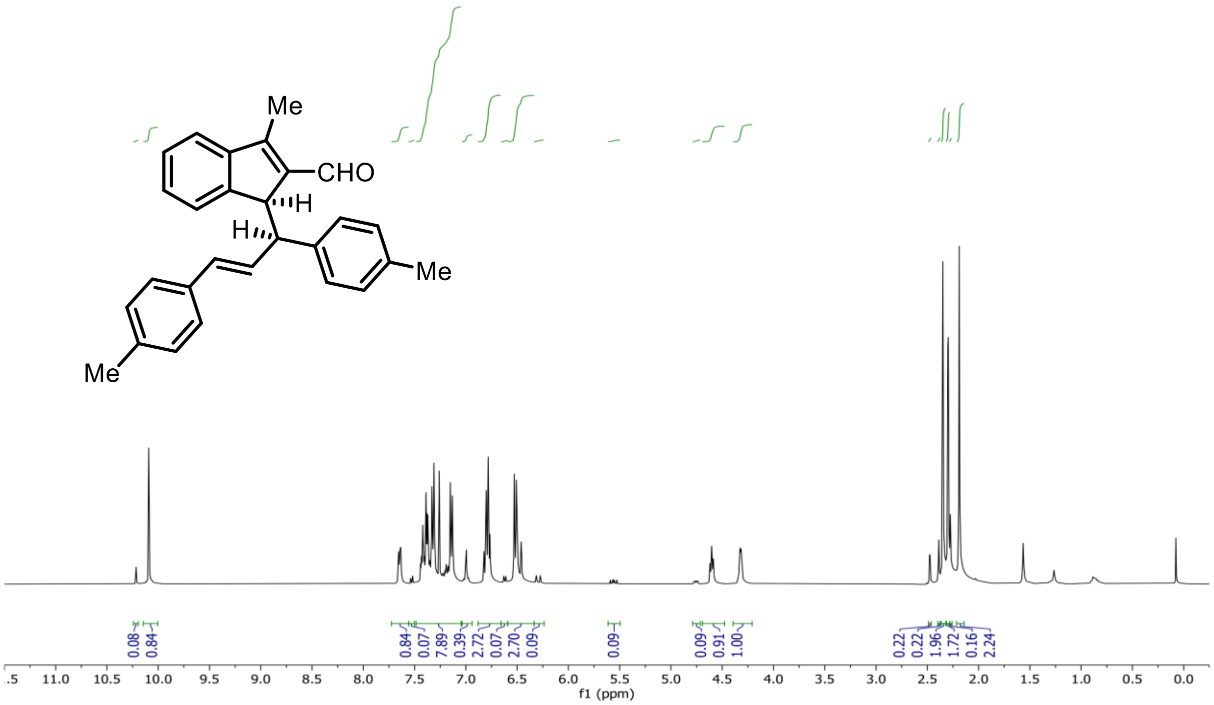

Compound **4n** ^{13}C NMR

(100 MHz, $CDCl_3$)

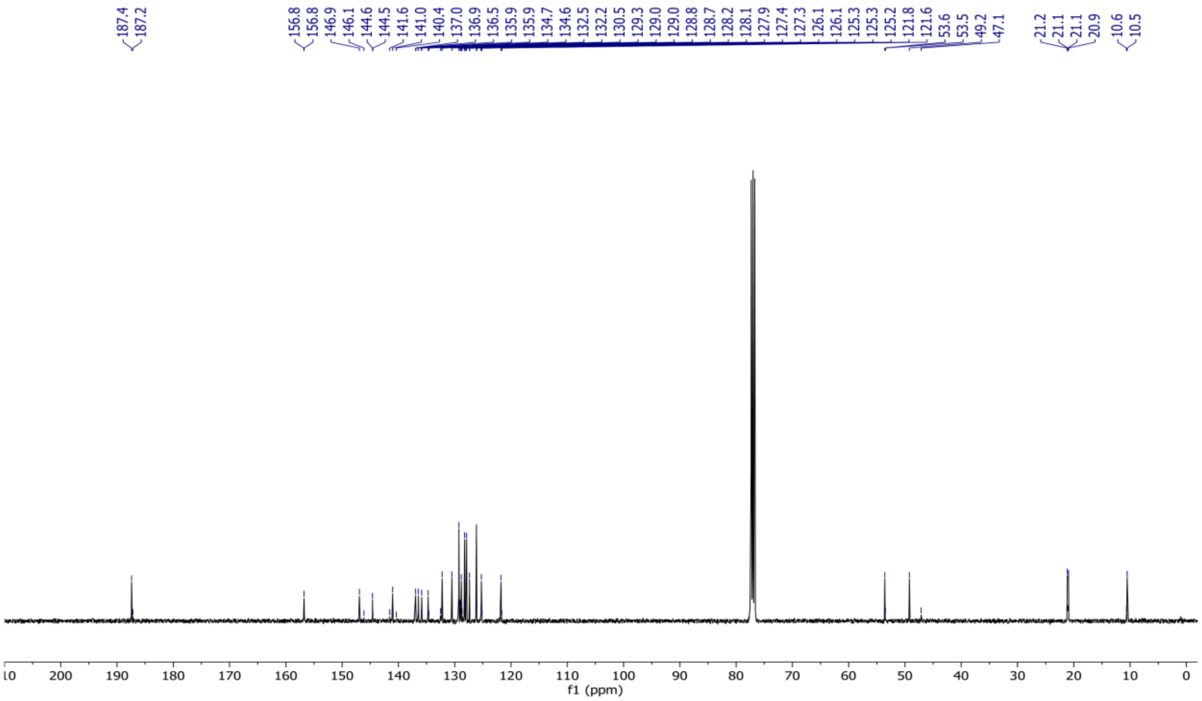

Compound **5n** ^{1}H NMR

(400 MHz, $CDCl_3$)

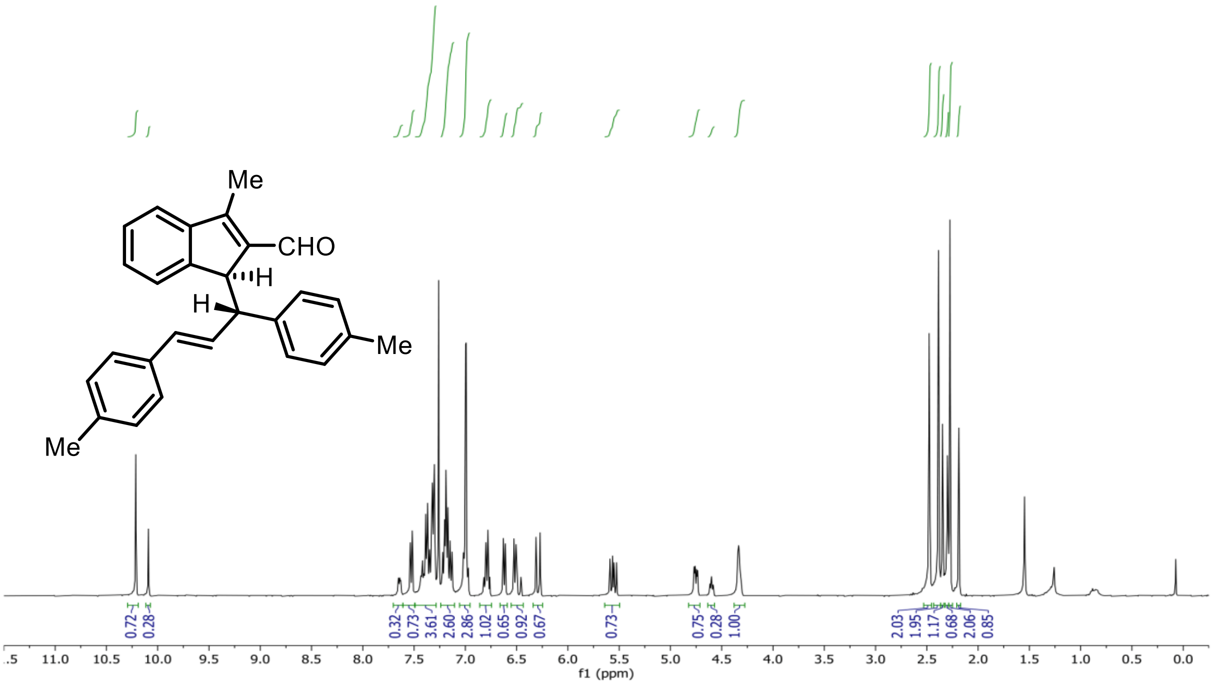

Compound **5n** ^{13}C NMR

(100 MHz, $CDCl_3$)

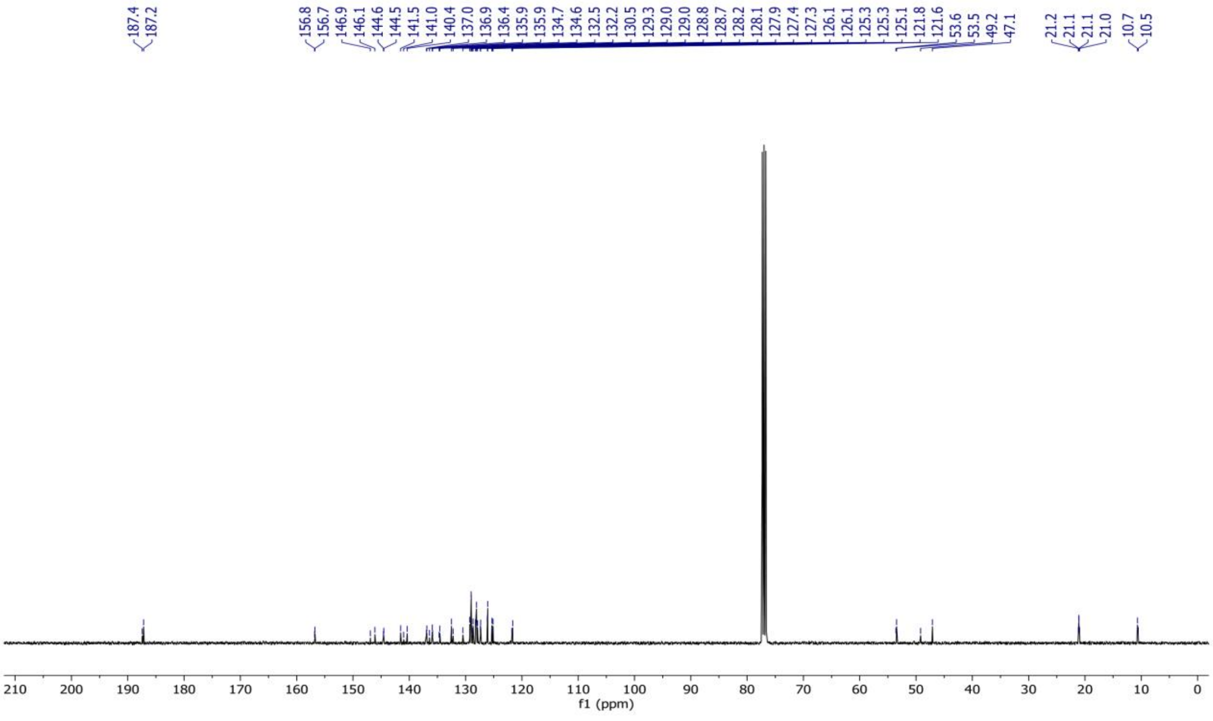

Compound **4o** ^{1}H NMR

(400 MHz, $CDCl_3$)

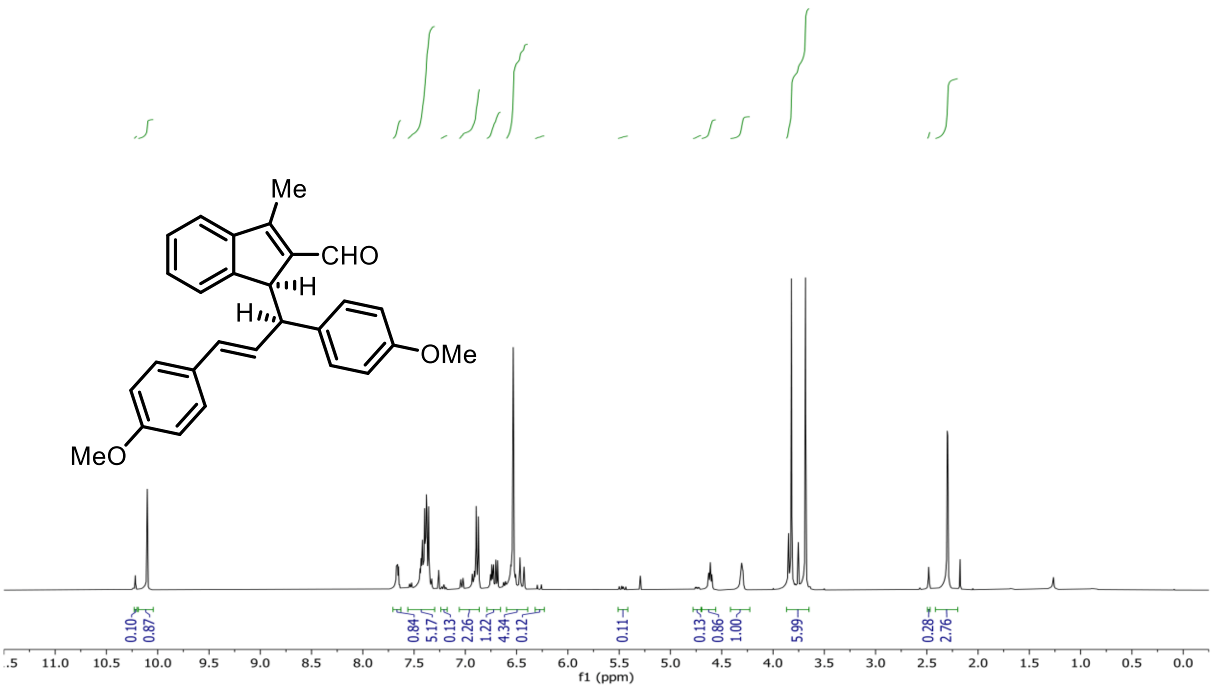

Compound **4o** ^{13}C NMR

(100 MHz, $CDCl_3$)

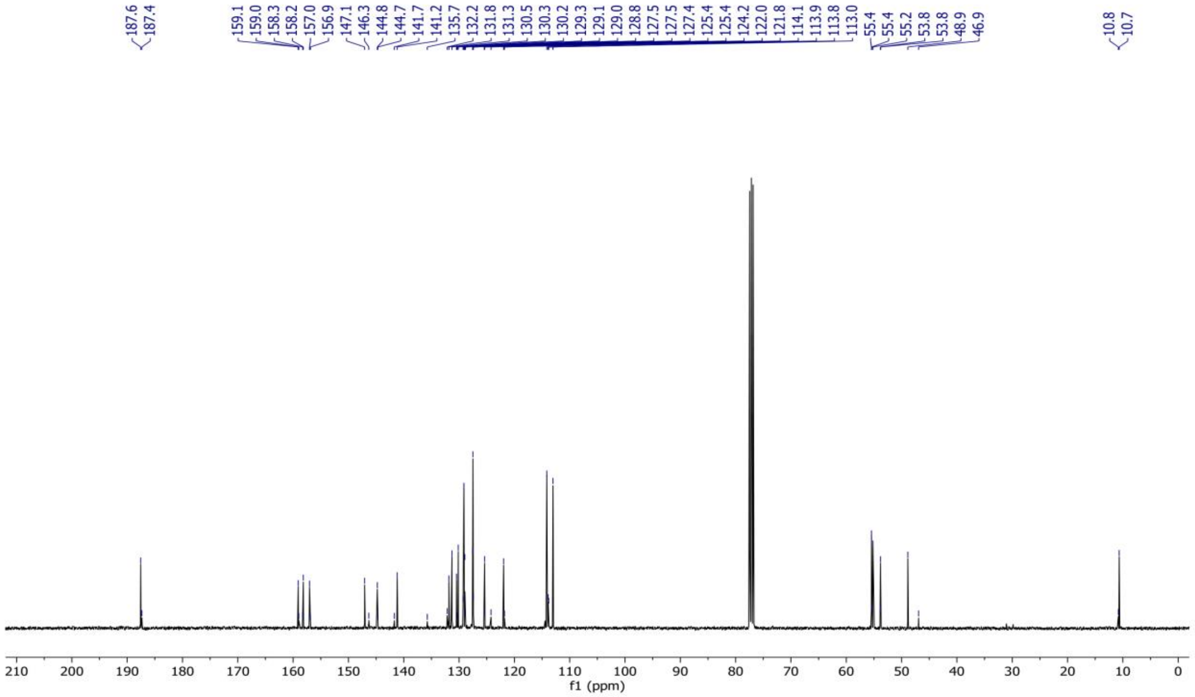

Compound **5o** ^{1}H NMR

(400 MHz, $CDCl_3$)

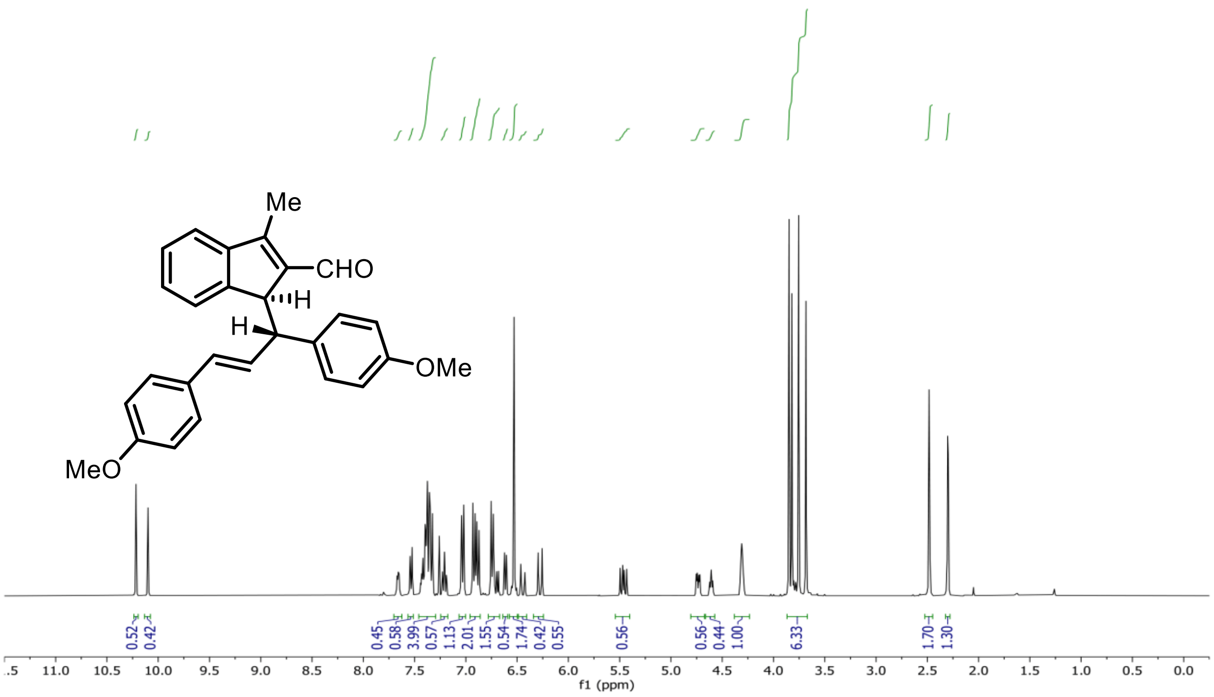

Compound **5o** ^{13}C NMR

(100 MHz, $CDCl_3$)

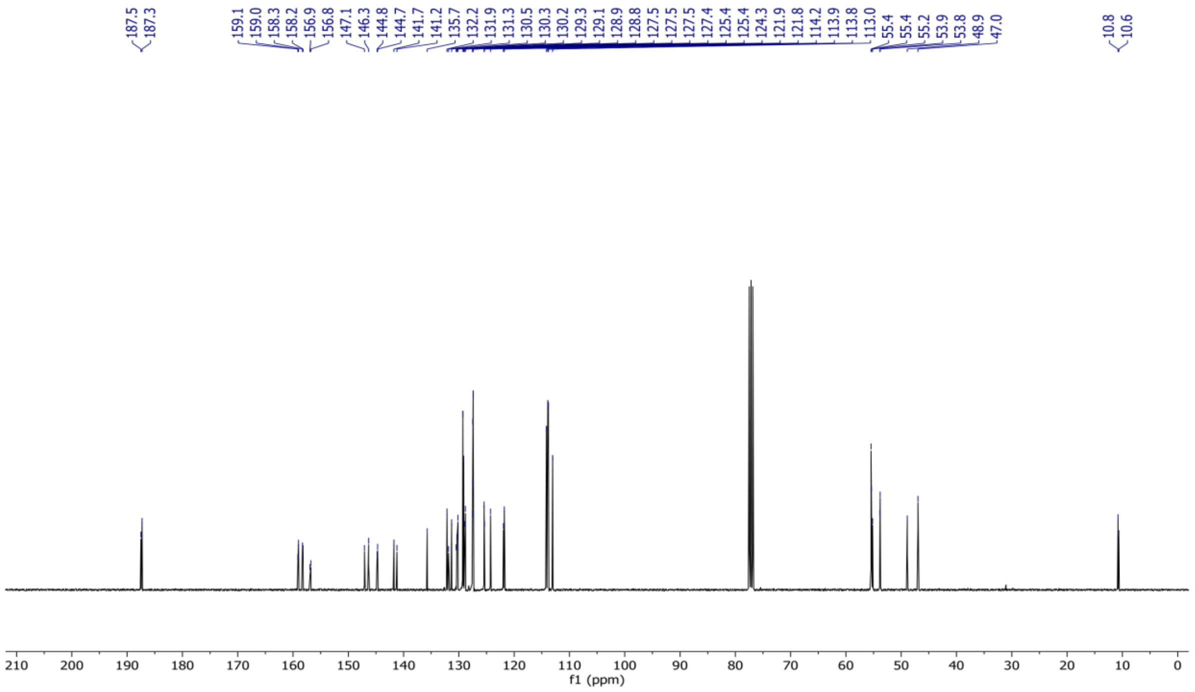

Compound **4p** ^{1}H NMR

(400 MHz, $CDCl_3$)

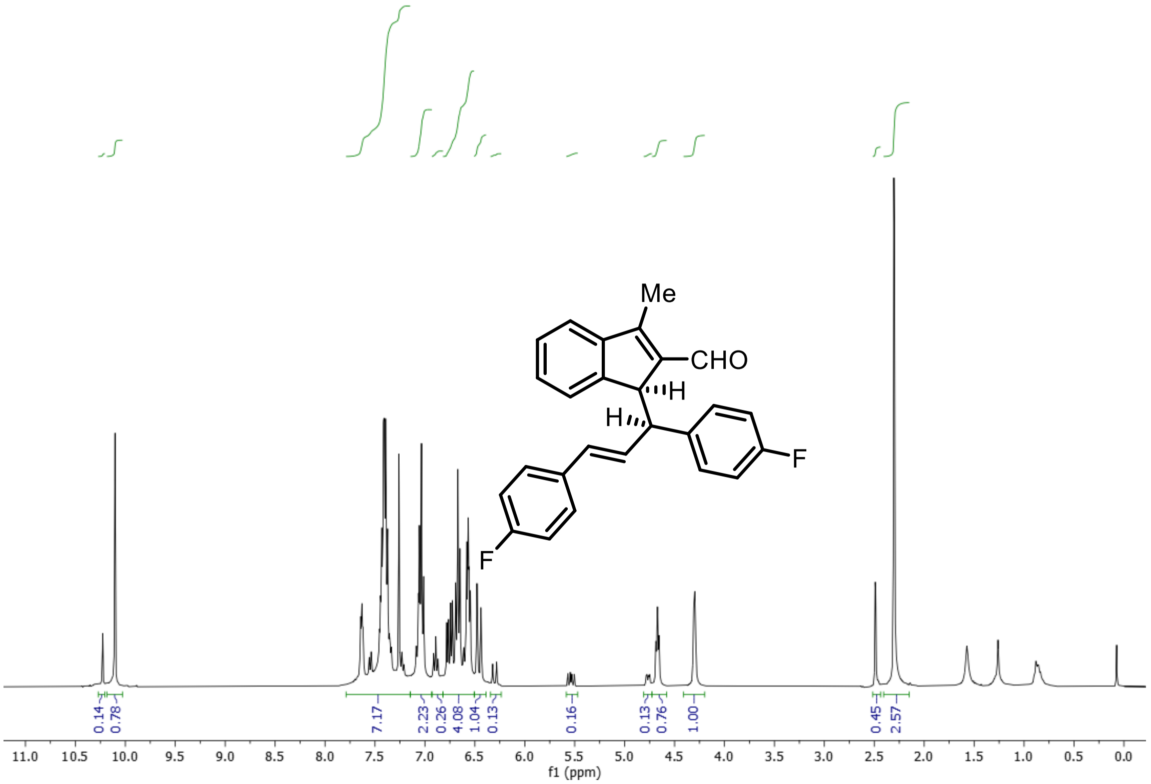

Compound **4p** ^{13}C NMR

(100 MHz, $CDCl_3$)

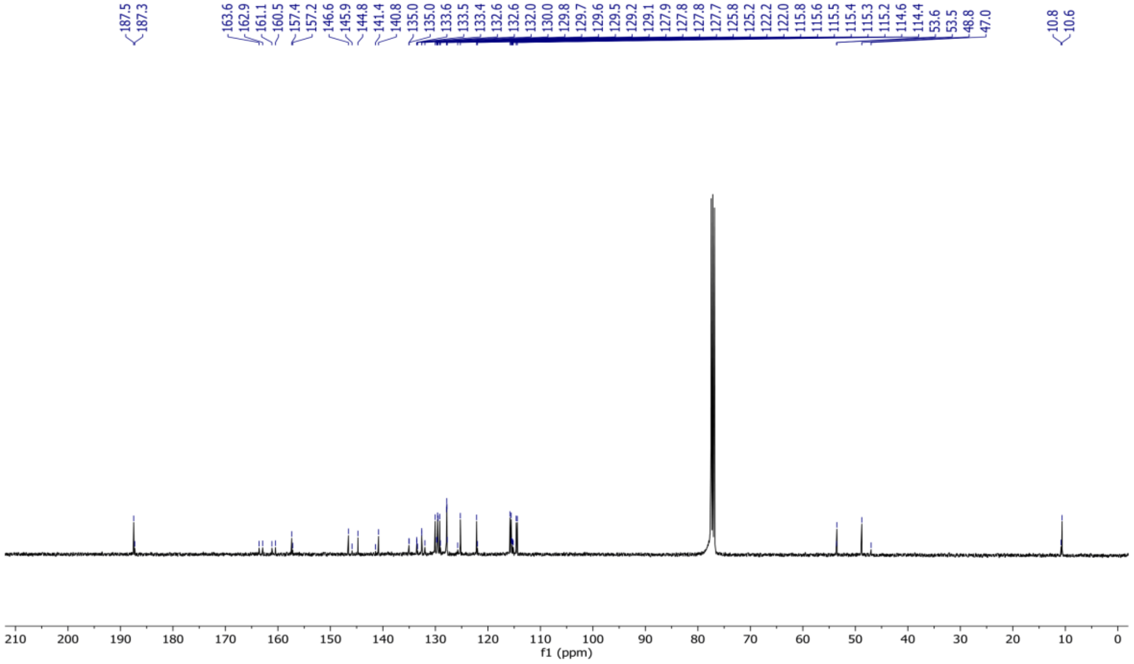

Compound **4p** ^{19}F NMR

(375 MHz, $CDCl_3$)

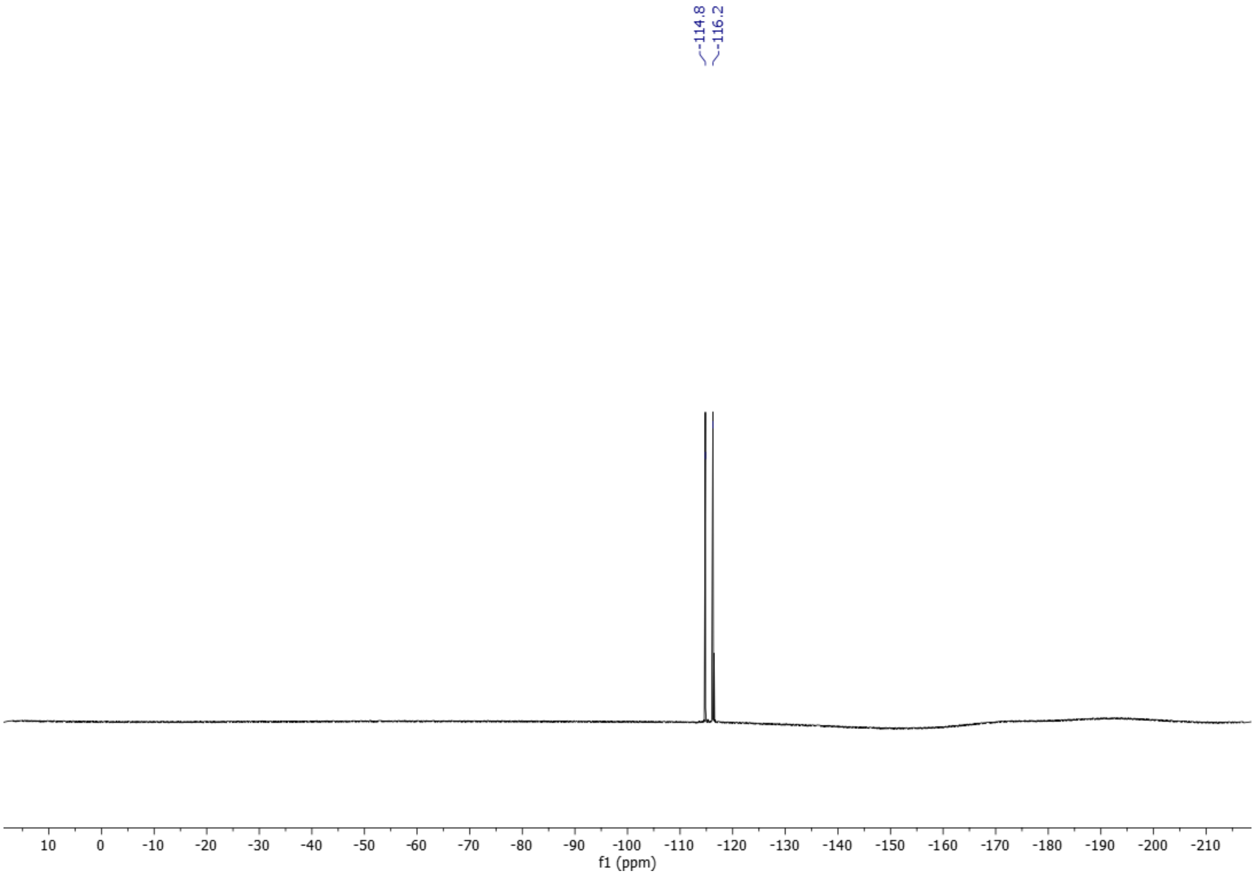

Compound **5p** ^{1}H NMR

(400 MHz, $CDCl_3$)

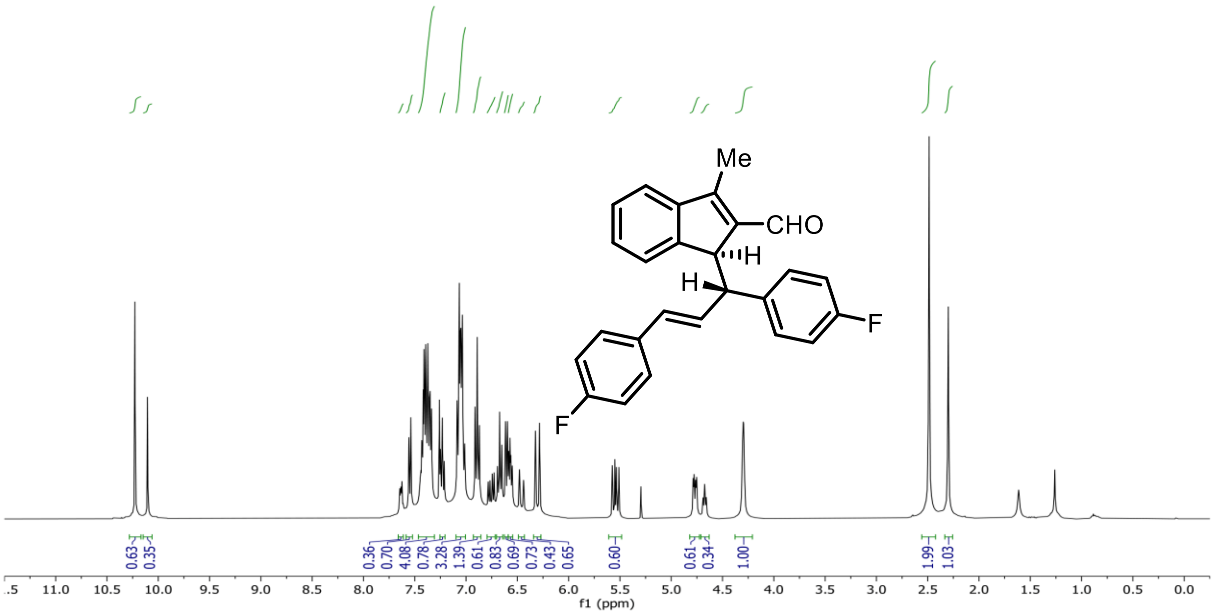

Compound **5p** ^{13}C NMR

(100 MHz, $CDCl_3$)

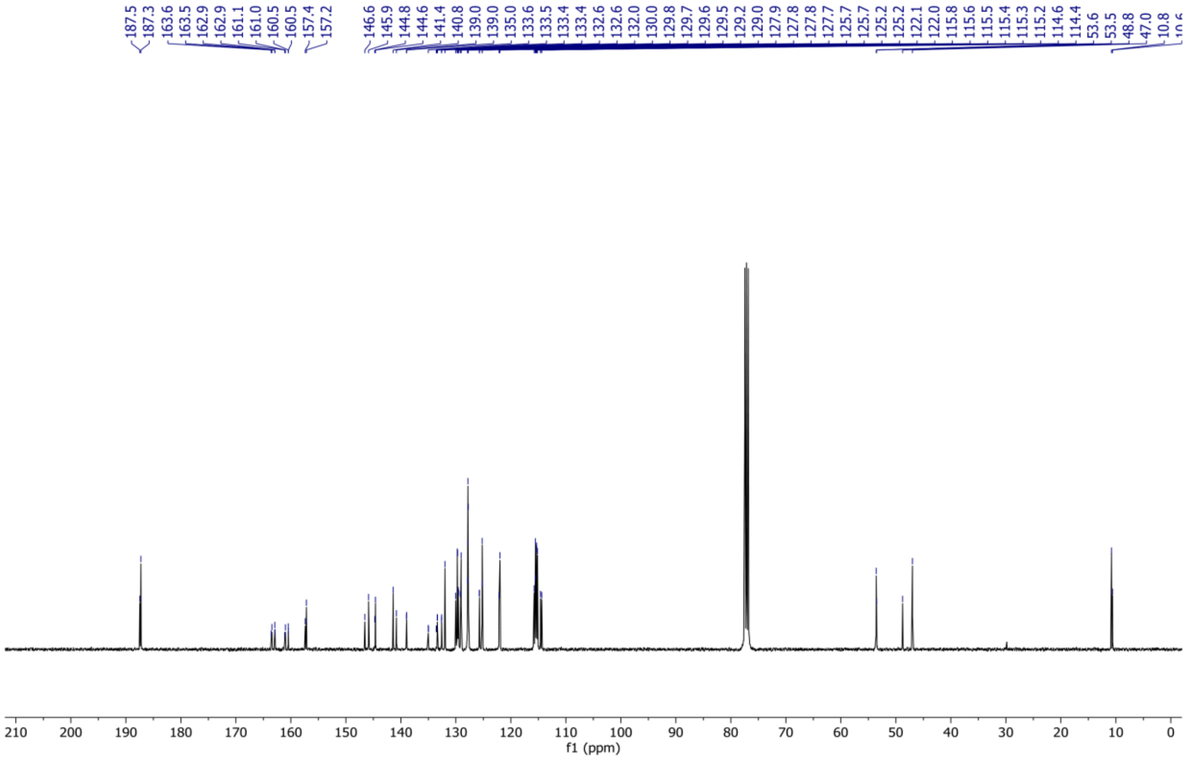

Compound **5p** ^{19}F NMR

(375 MHz, $CDCl_3$)

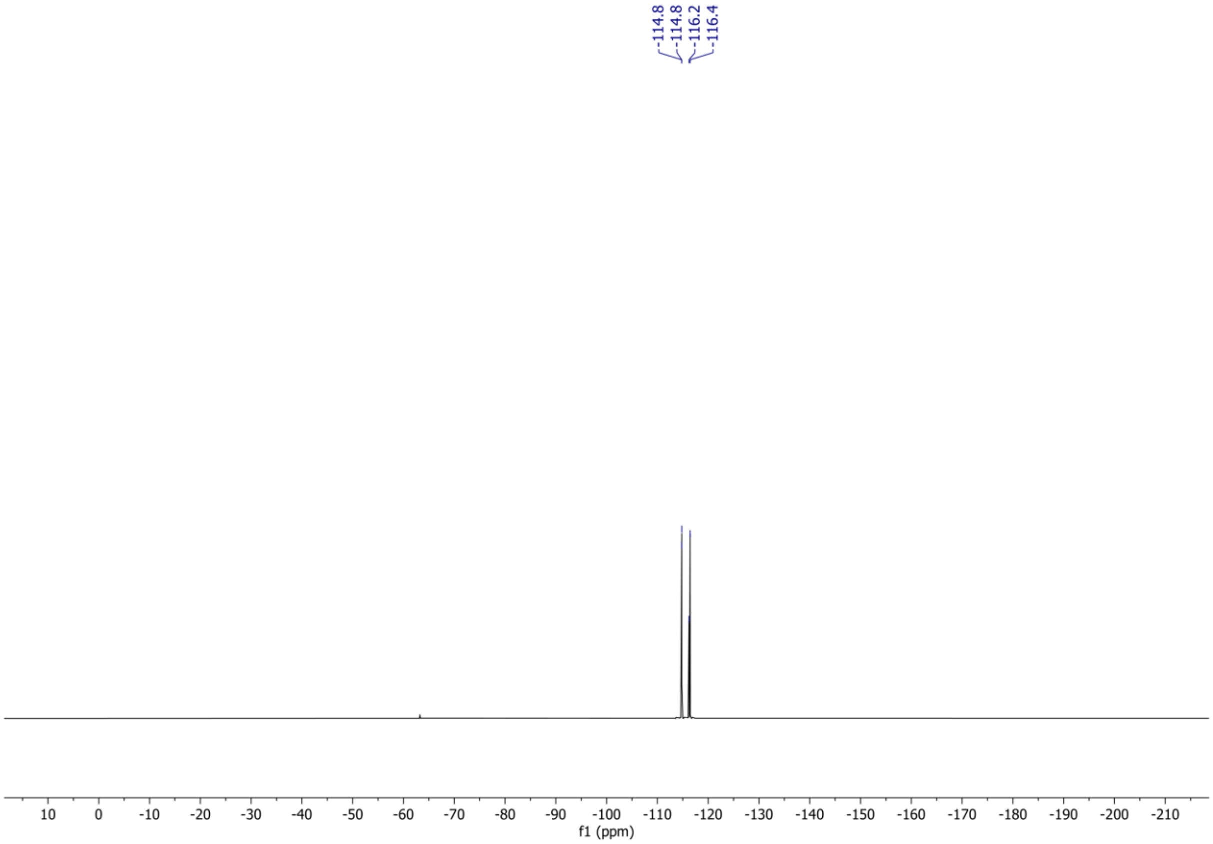

Compound **4q** ^{1}H NMR

(400 MHz, $CDCl_3$)

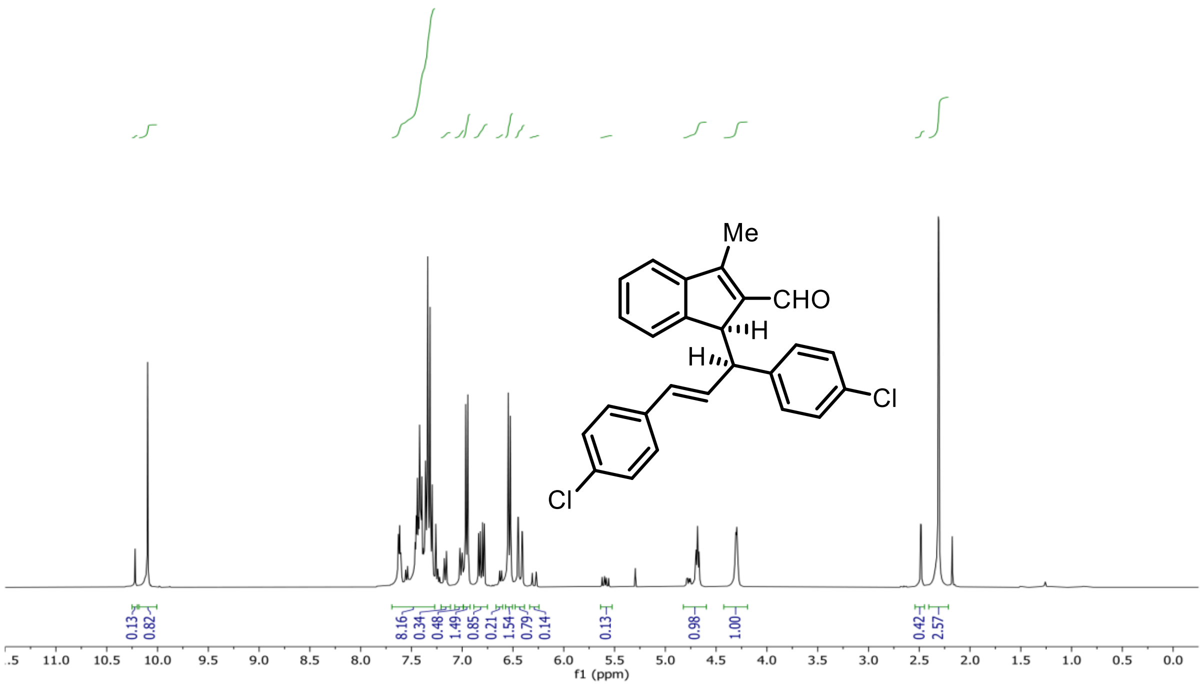

Compound **4q** ^{13}C NMR

(100 MHz, $CDCl_3$)

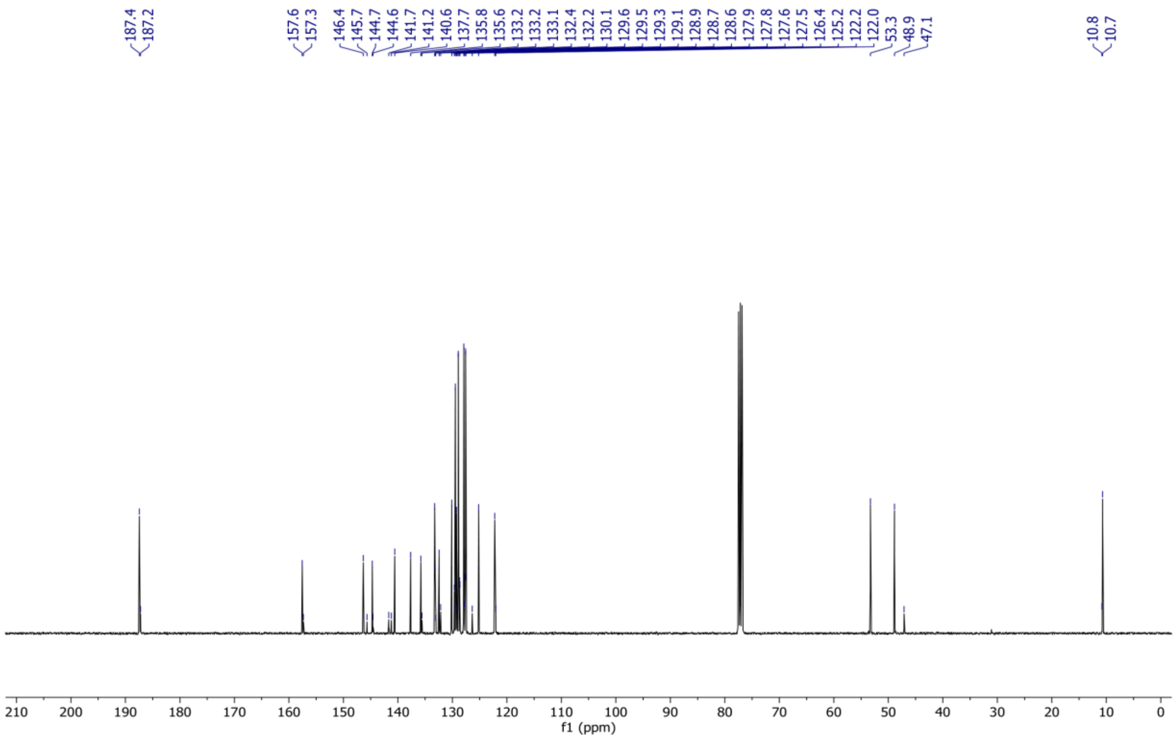

Compound **5q** ^{1}H NMR

(400 MHz, $CDCl_3$)

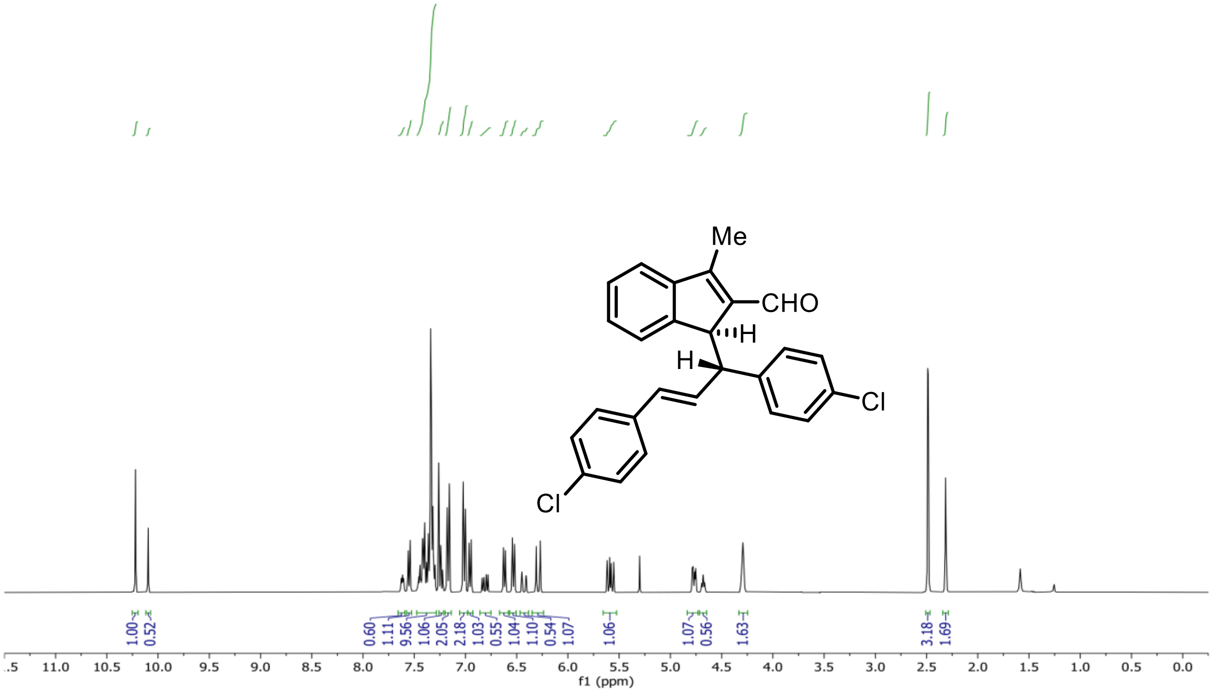

Compound **5q** ^{13}C NMR

(100 MHz, $CDCl_3$)

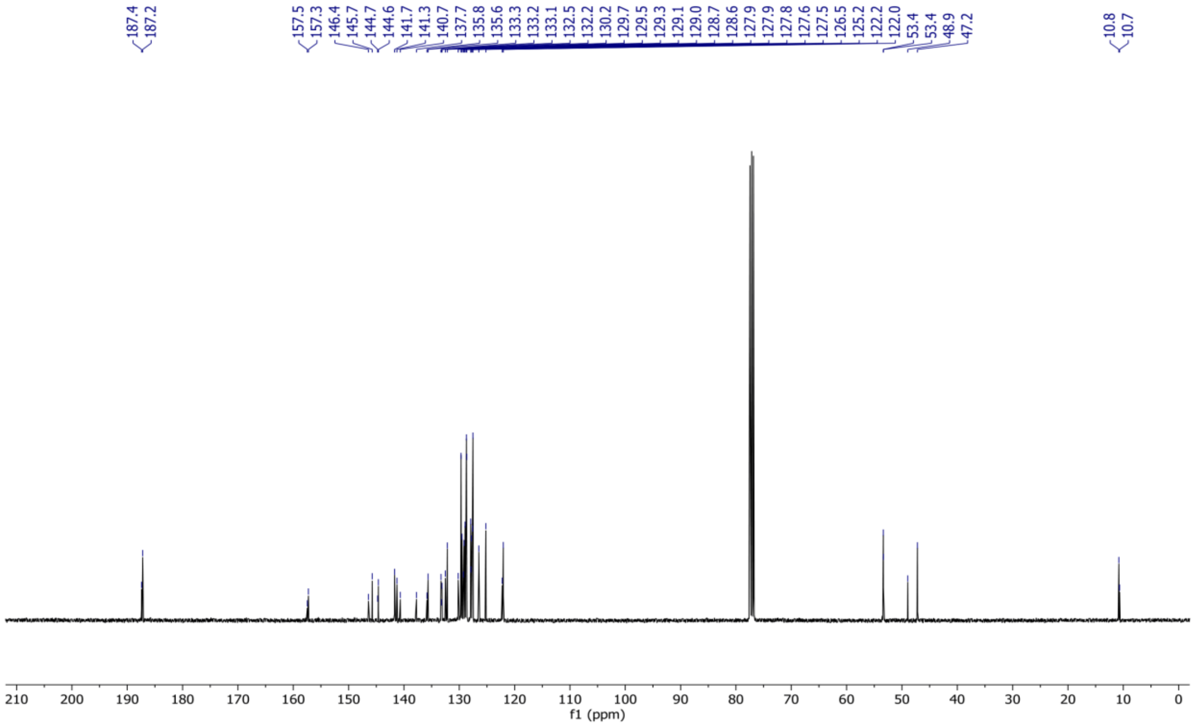

Compound **4r** ^{1}H NMR

(400 MHz, $CDCl_3$)

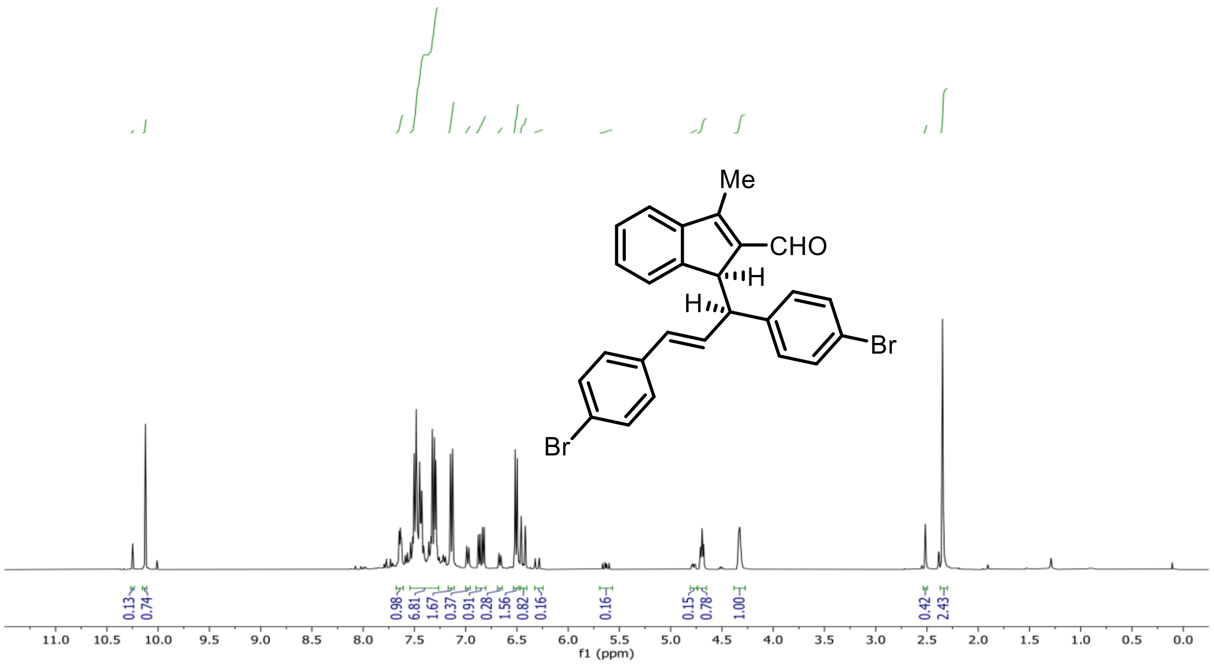

Compound **4r** ^{13}C NMR

(100 MHz, $CDCl_3$)

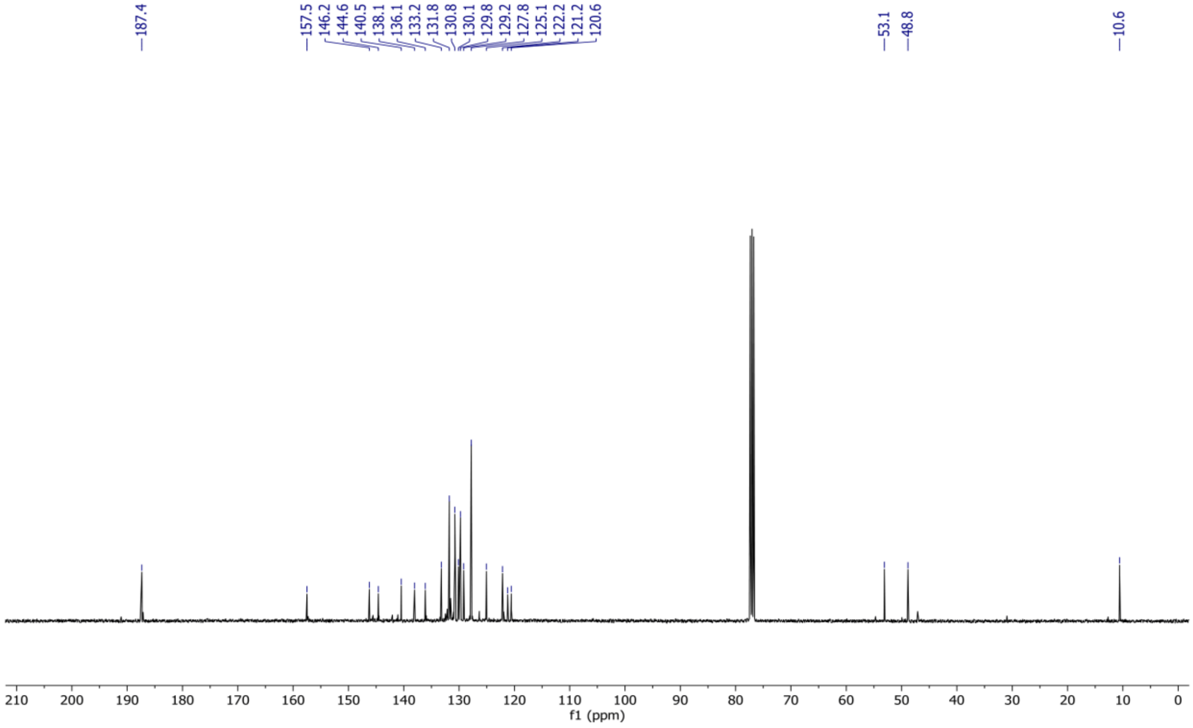

Compound **5r** ^{1}H NMR

(400 MHz, $CDCl_3$)

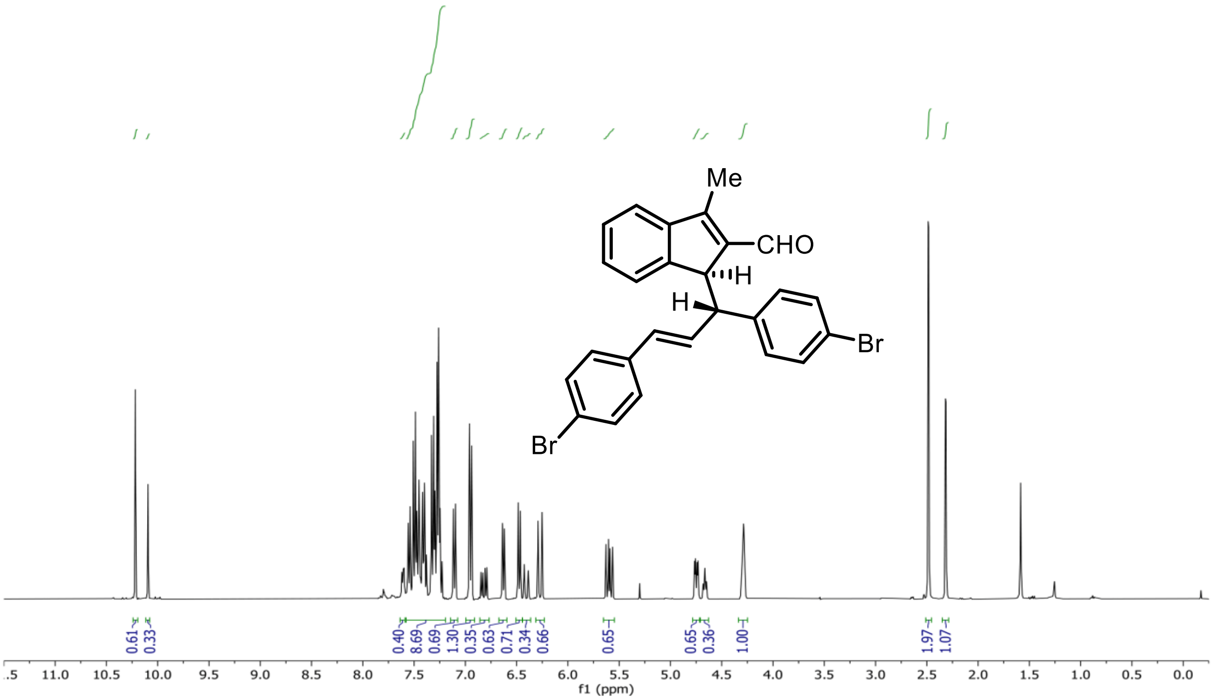

Compound **5r** ^{13}C NMR

(100 MHz, $CDCl_3$)

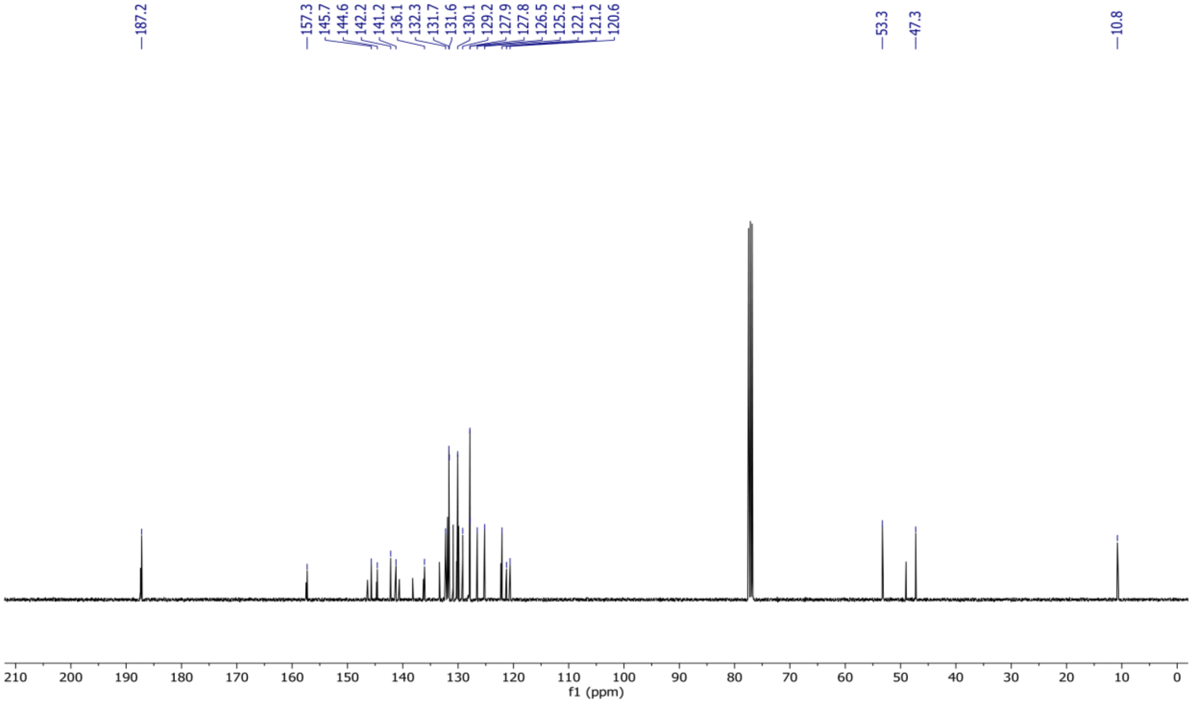

Compound **4s** ^{1}H NMR

(400 MHz, $CDCl_3$)

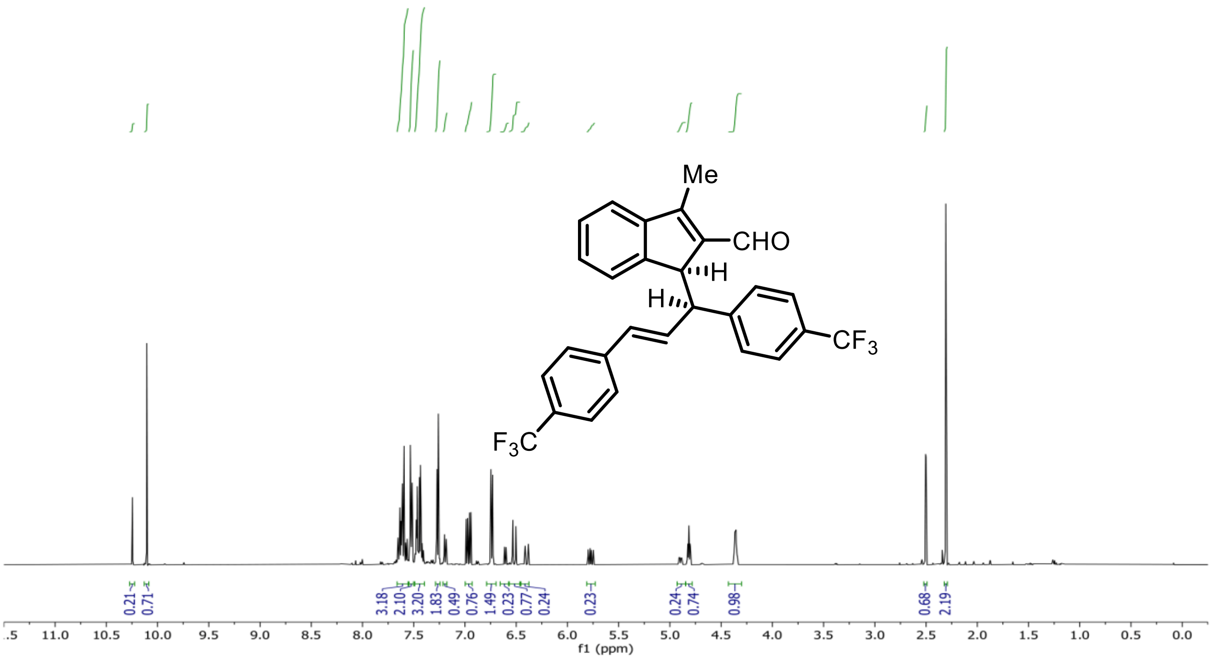

Compound **4s** ^{13}C NMR

(100 MHz, $CDCl_3$)

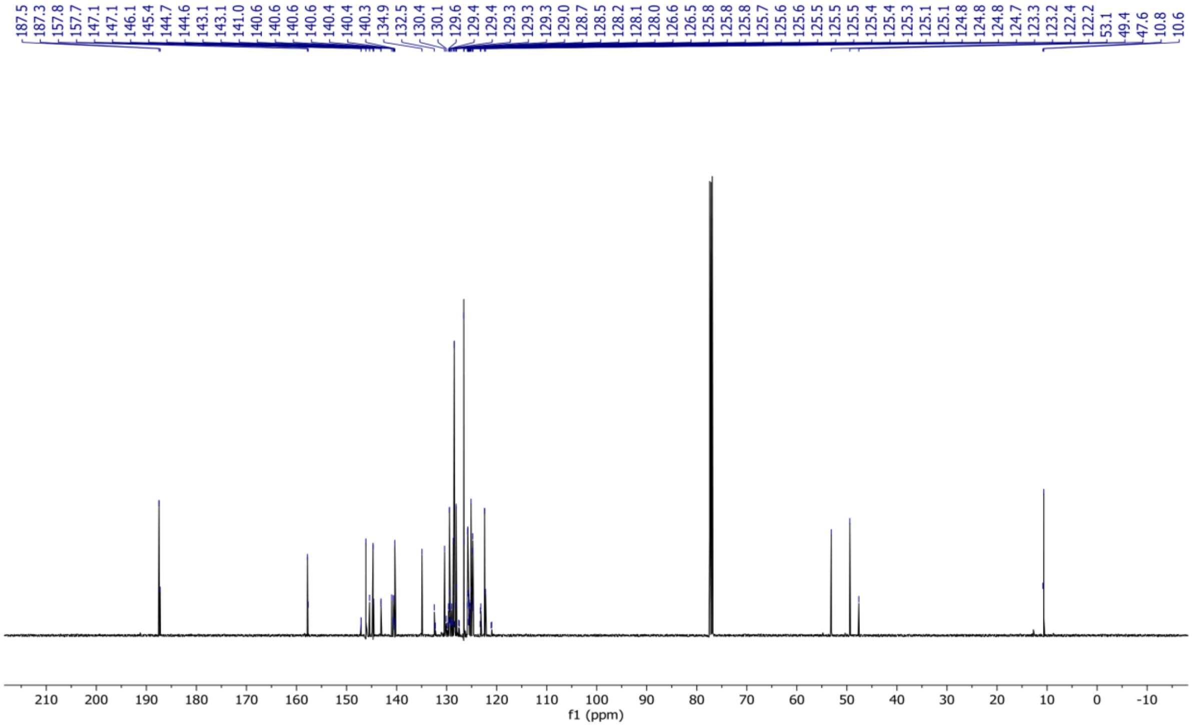

Compound **4s** ^{19}F NMR

(375 MHz, $CDCl_3$)

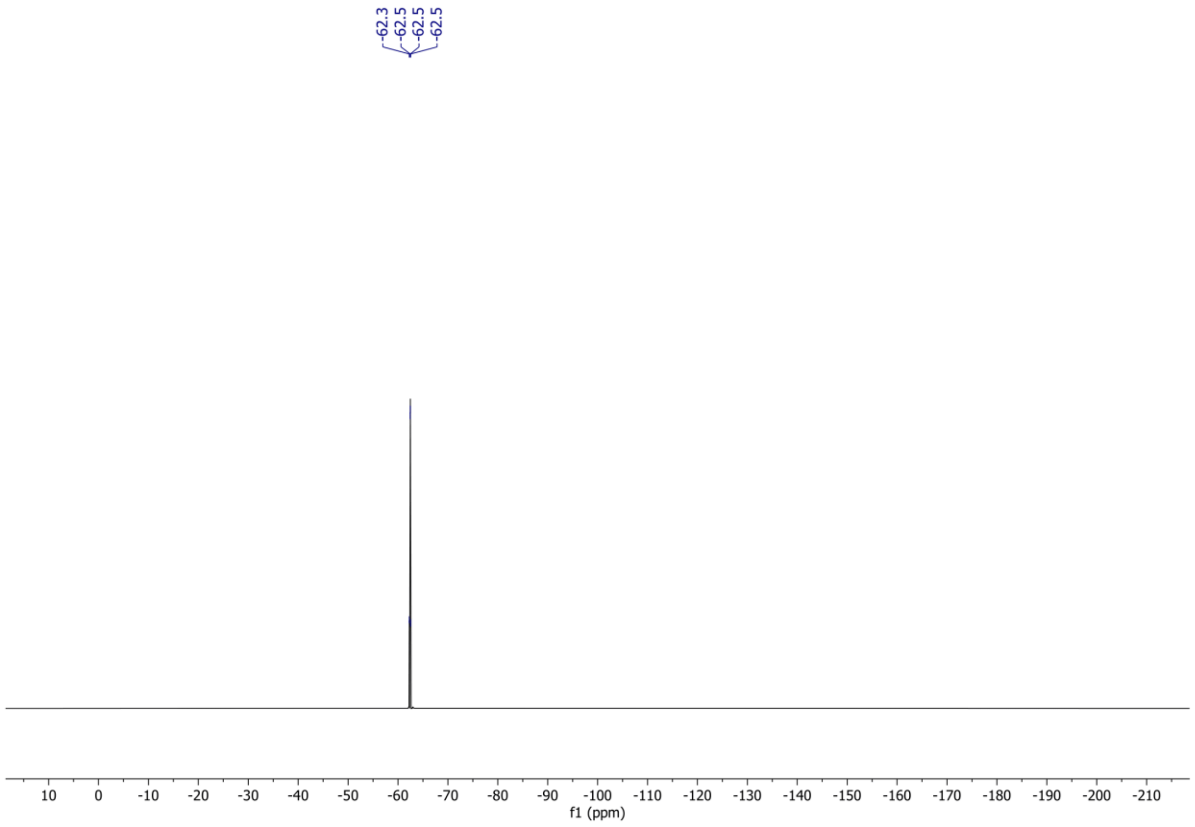

Compound **5s** ^{1}H NMR

(400 MHz, $CDCl_3$)

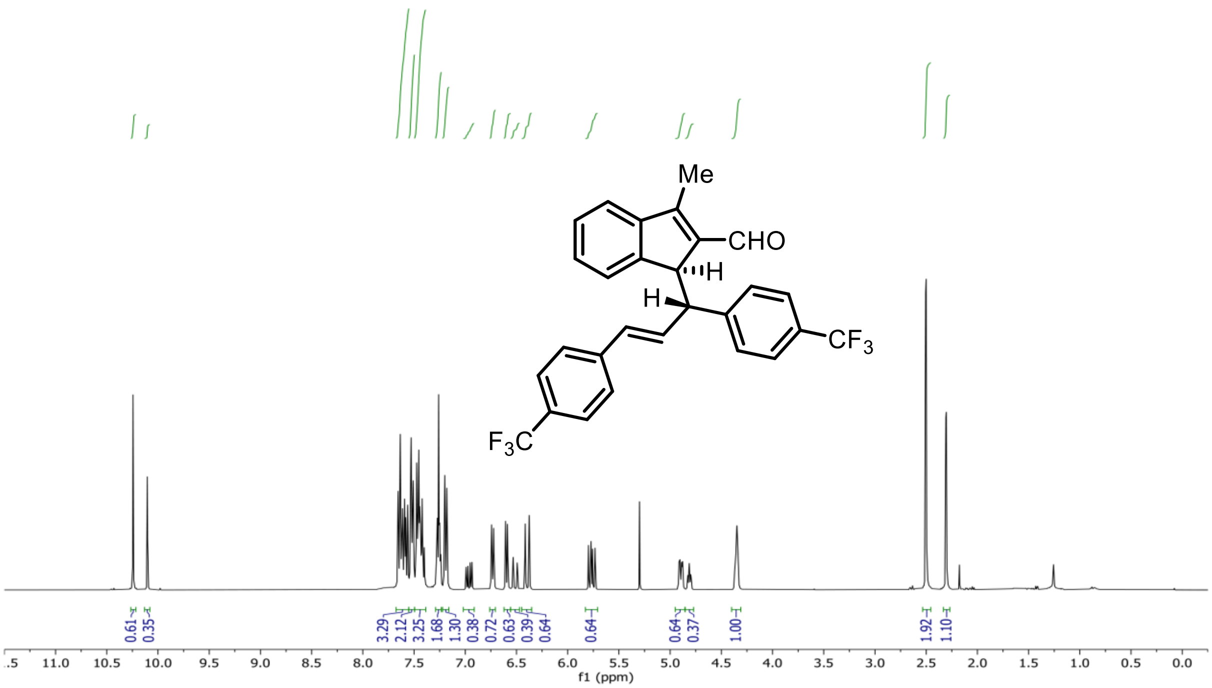

Compound **5s** ^{13}C NMR

(100 MHz, $CDCl_3$)

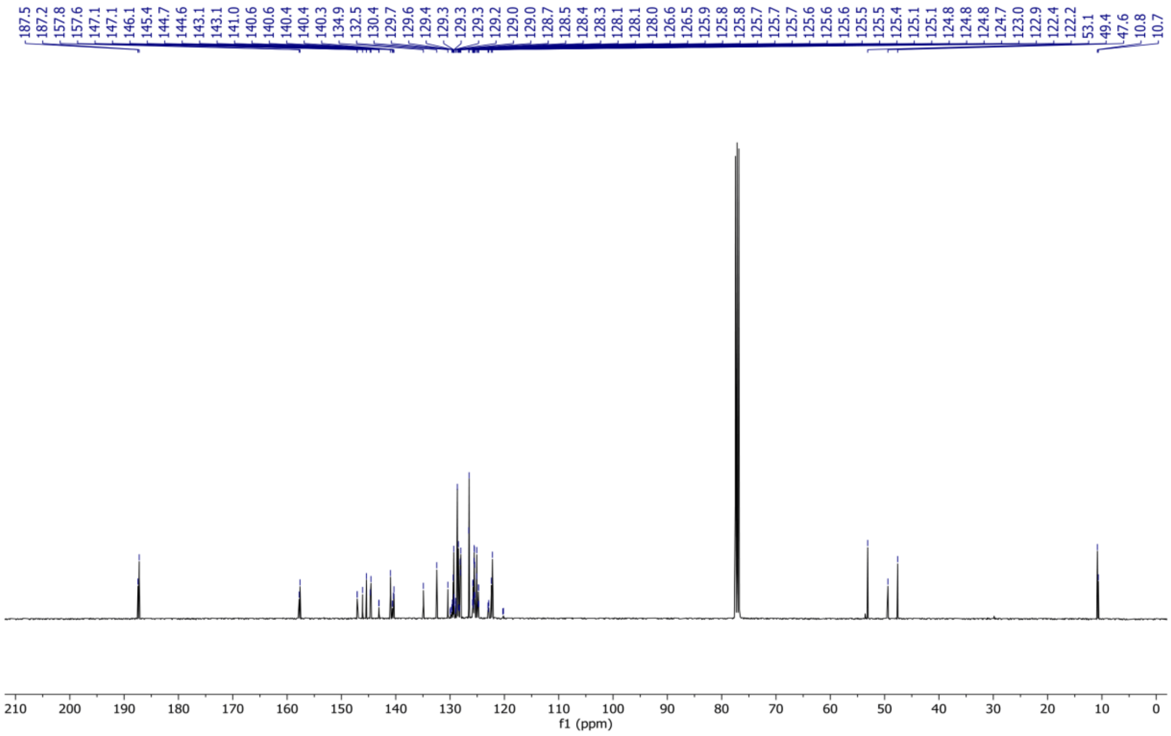

Compound **5s** ^{19}F NMR

(100 MHz, $CDCl_3$)

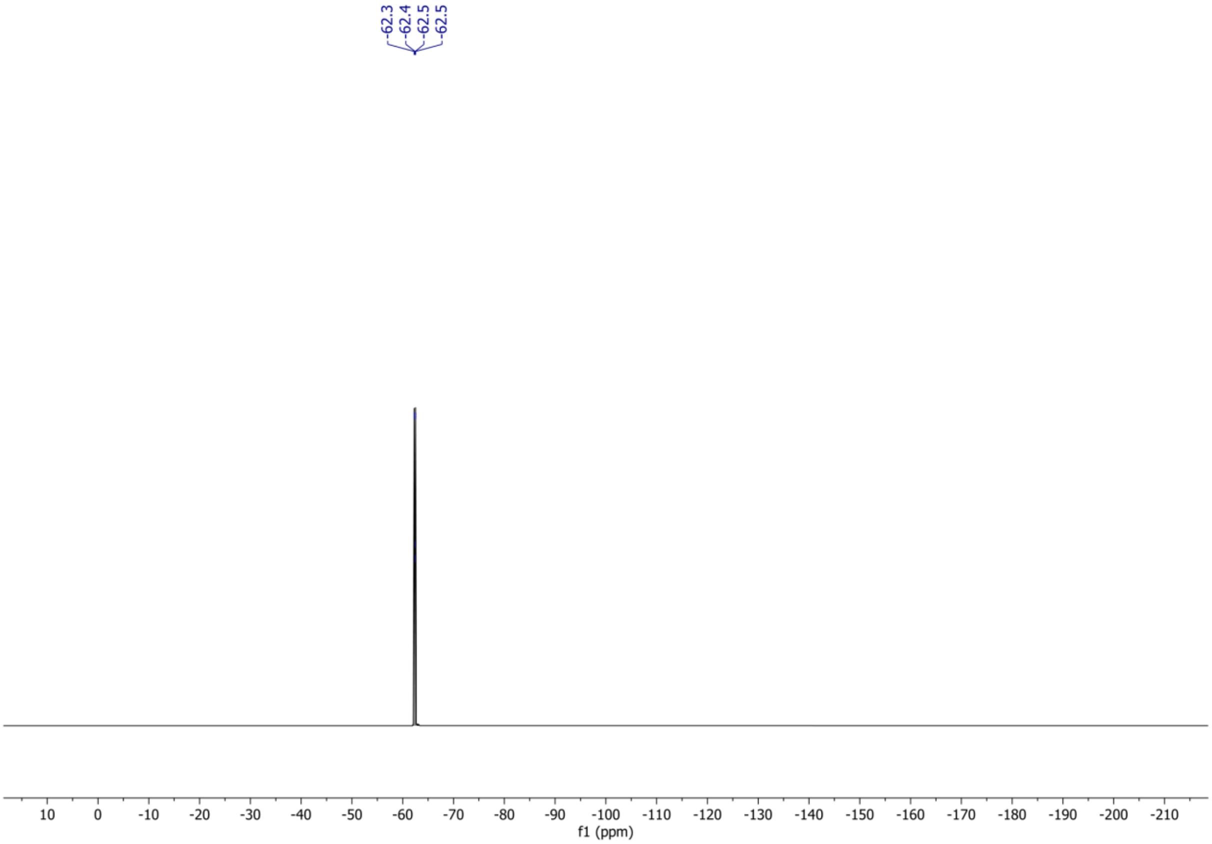

Compound **4t** ^{1}H NMR

(400 MHz, $CDCl_3$)

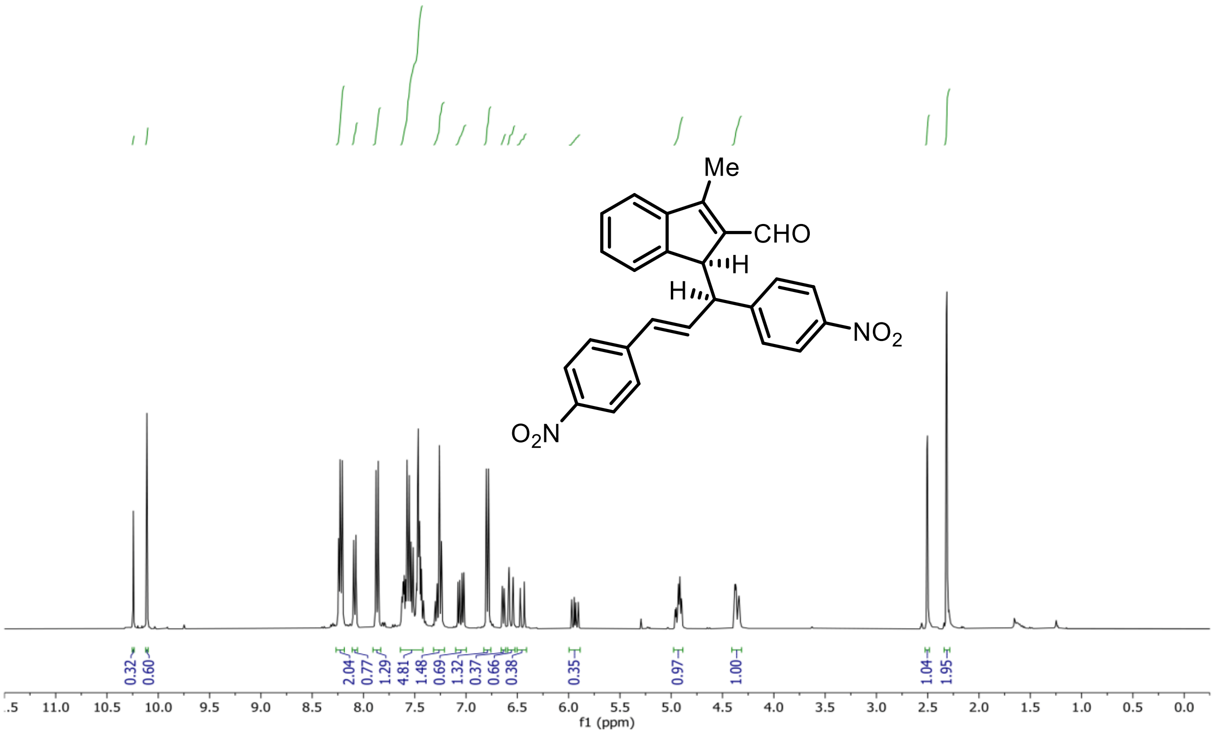

Compound **4t** ^{13}C NMR

(100 MHz, $CDCl_3$)

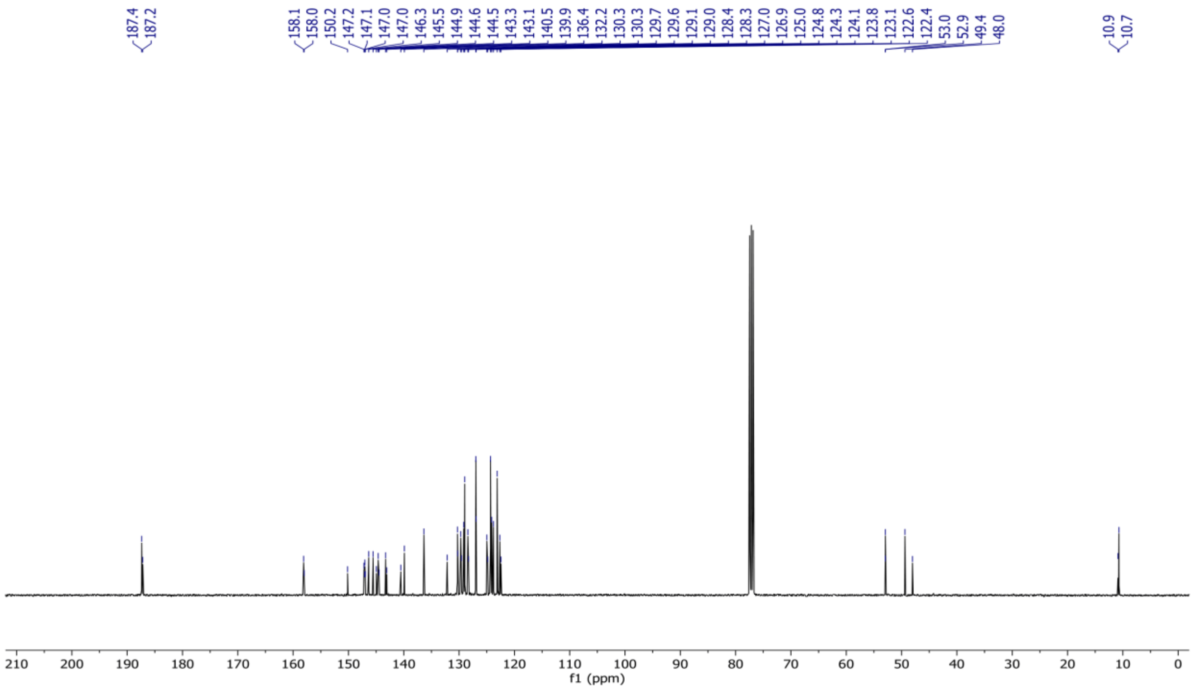

Compound **5t** ^{1}H NMR

(400 MHz, $CDCl_3$)

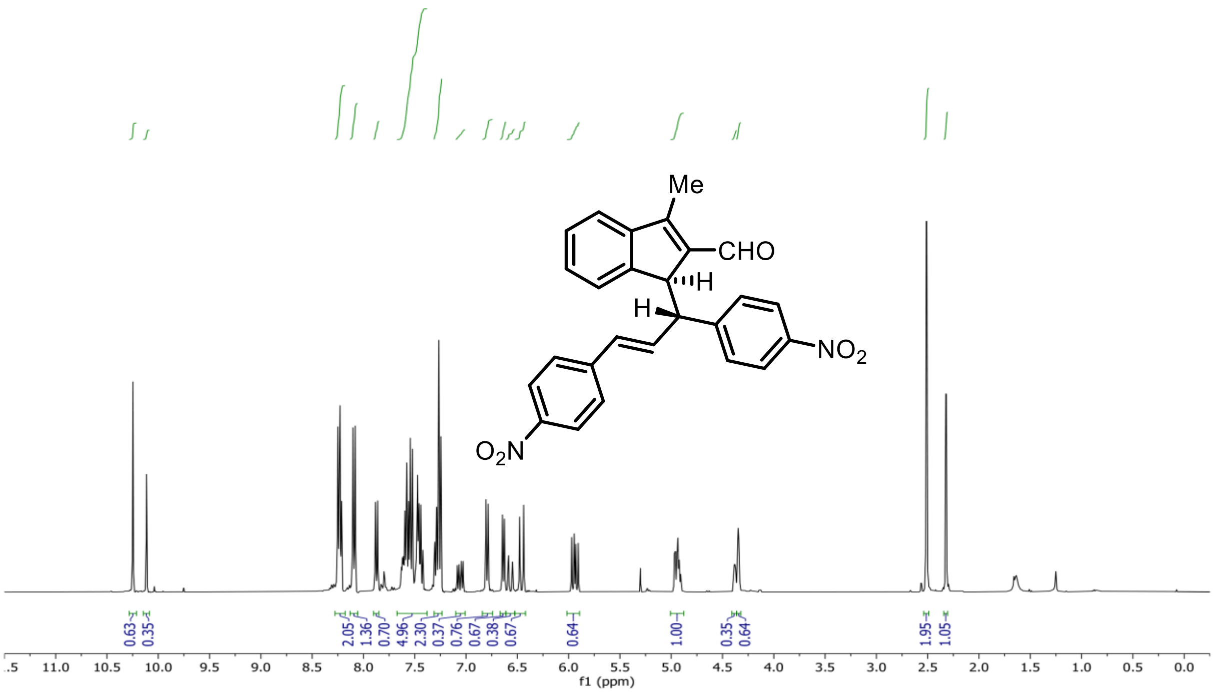

Compound **5t** ^{13}C NMR

(100 MHz, $CDCl_3$)

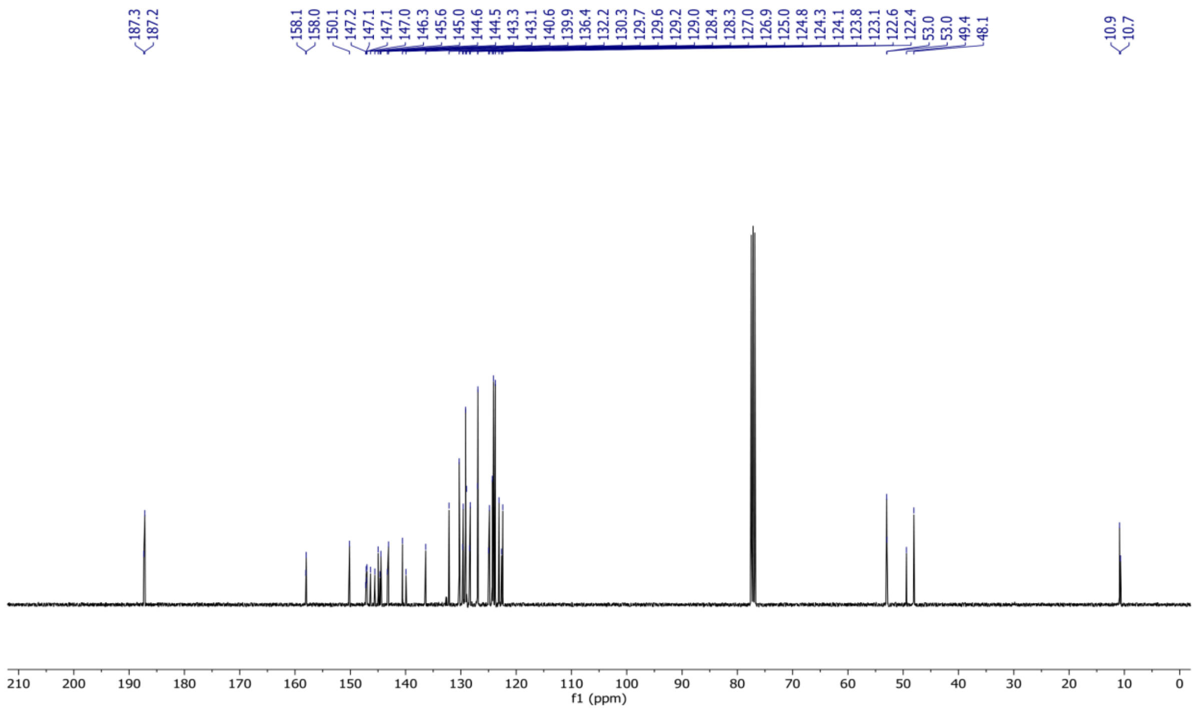

Compound **4u** ^{1}H NMR

(400 MHz, $CDCl_3$)

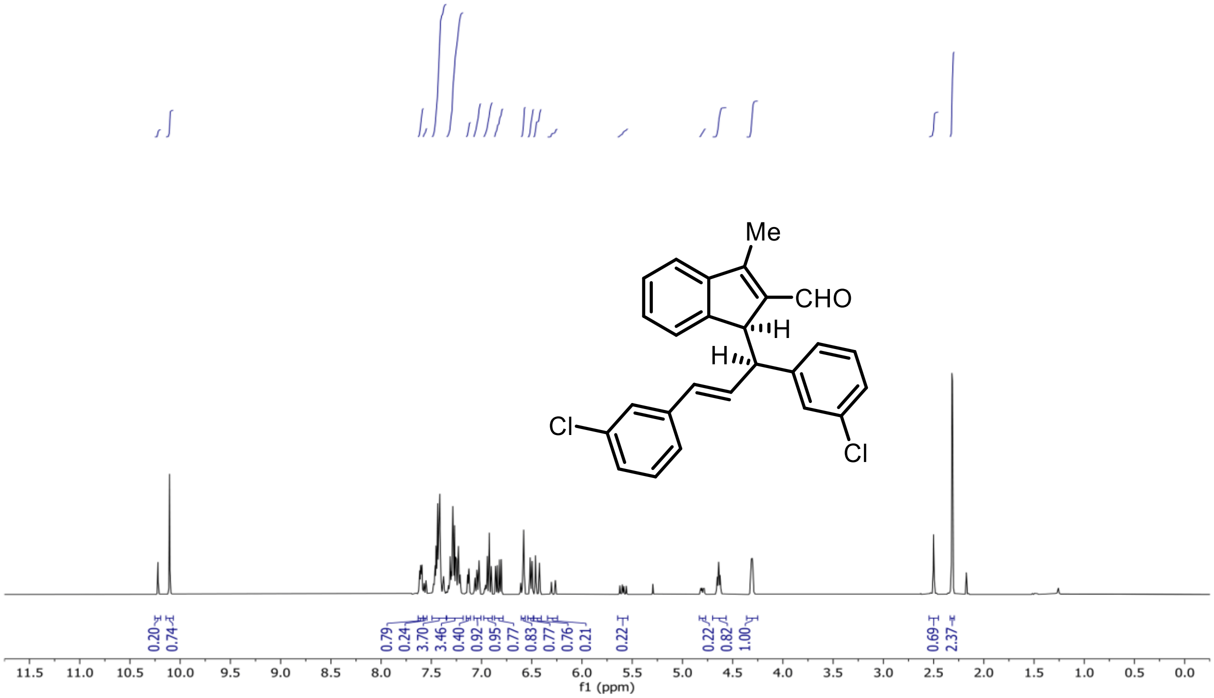

Compound **4u** ^{13}C NMR

(100 MHz, $CDCl_3$)

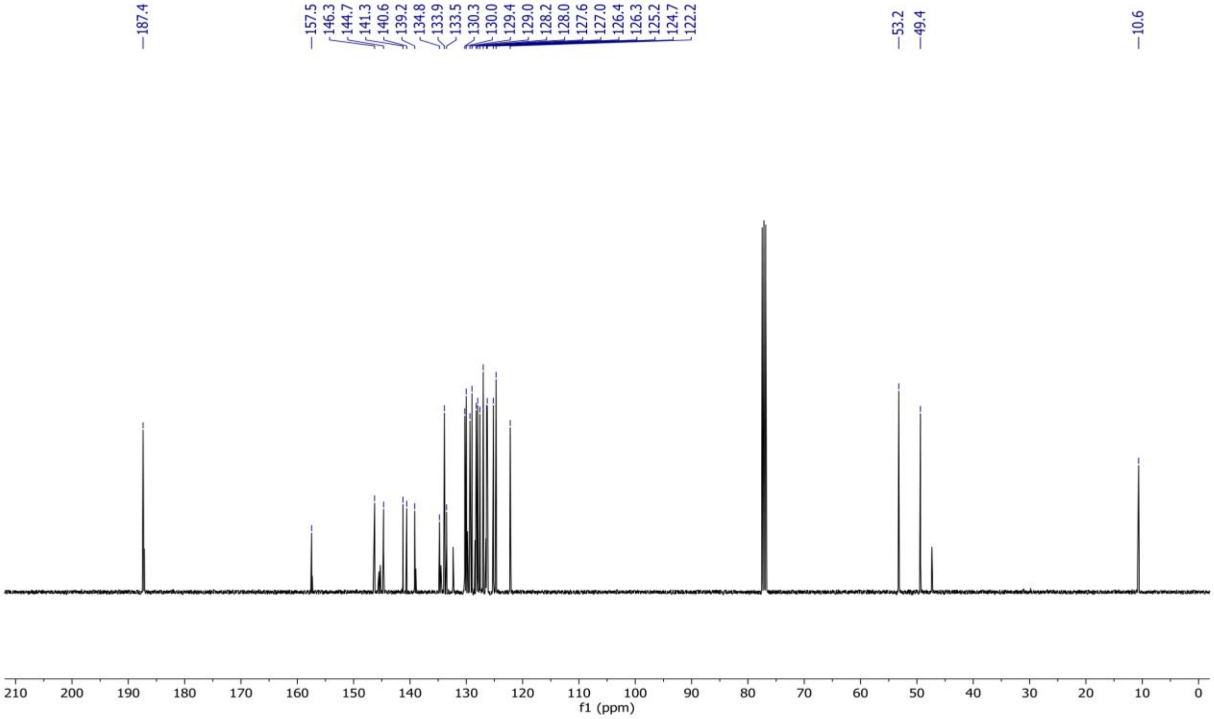

Compound **5u** ^{1}H NMR

(400 MHz, $CDCl_3$)

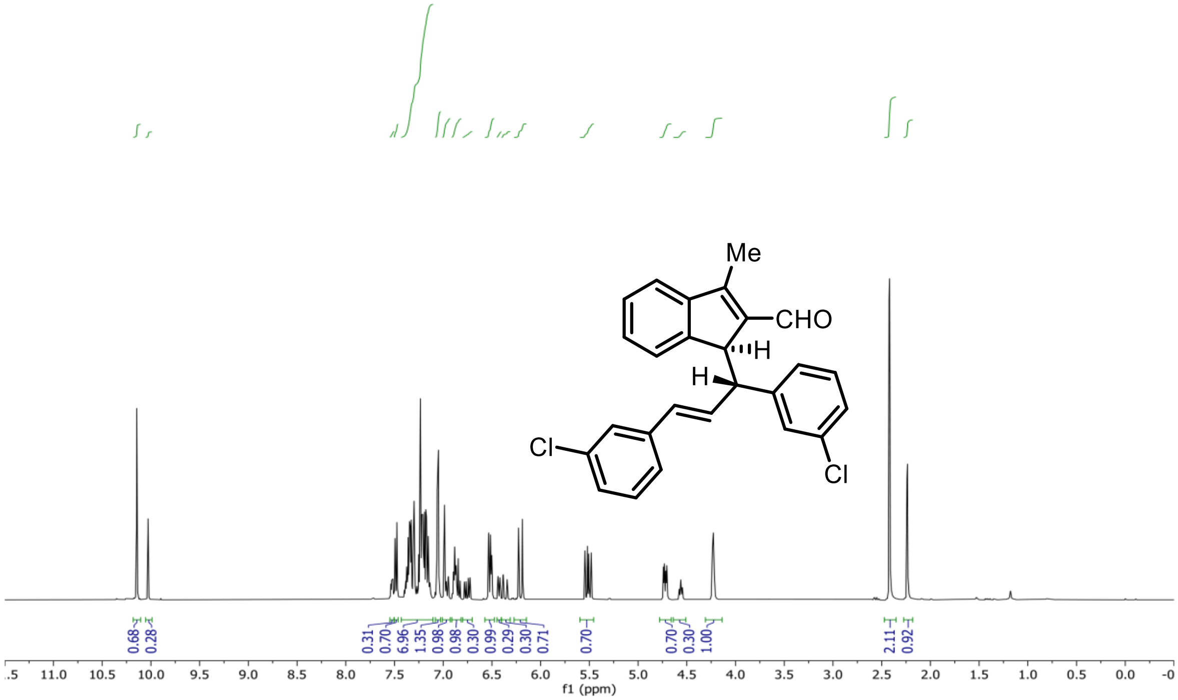

Compound **5u** ^{13}C NMR

(100 MHz, $CDCl_3$)

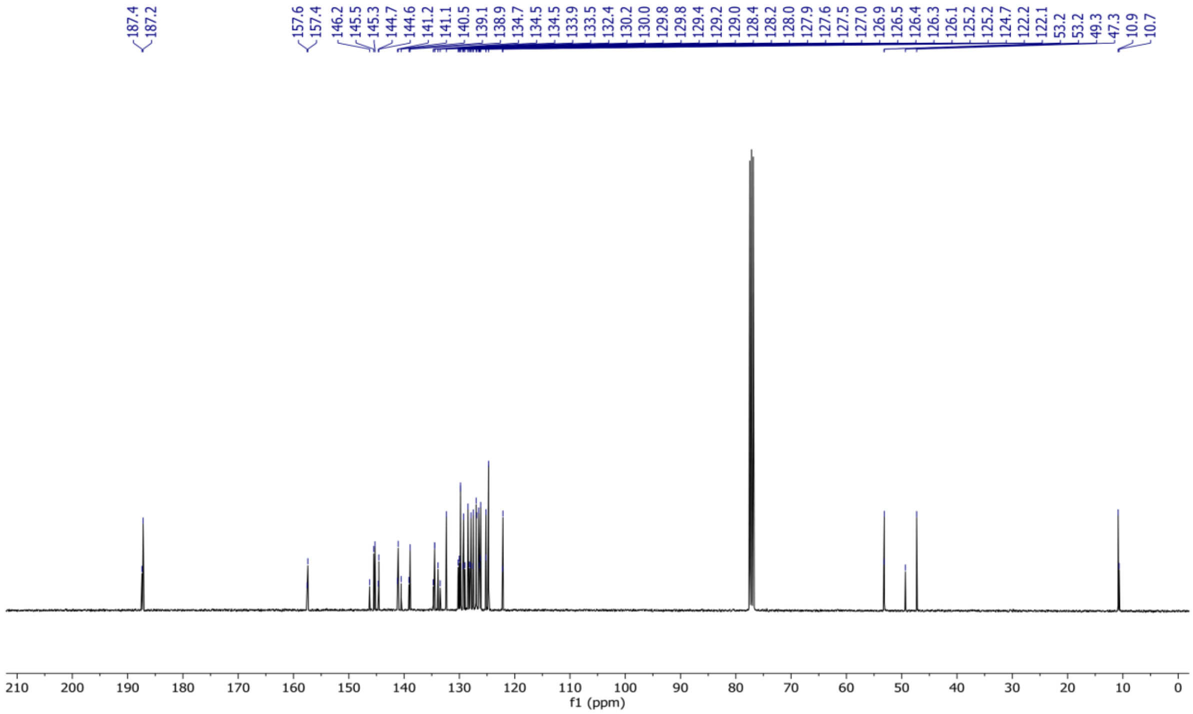

Compound **4v** ^{1}H NMR

(400 MHz, $CDCl_3$)

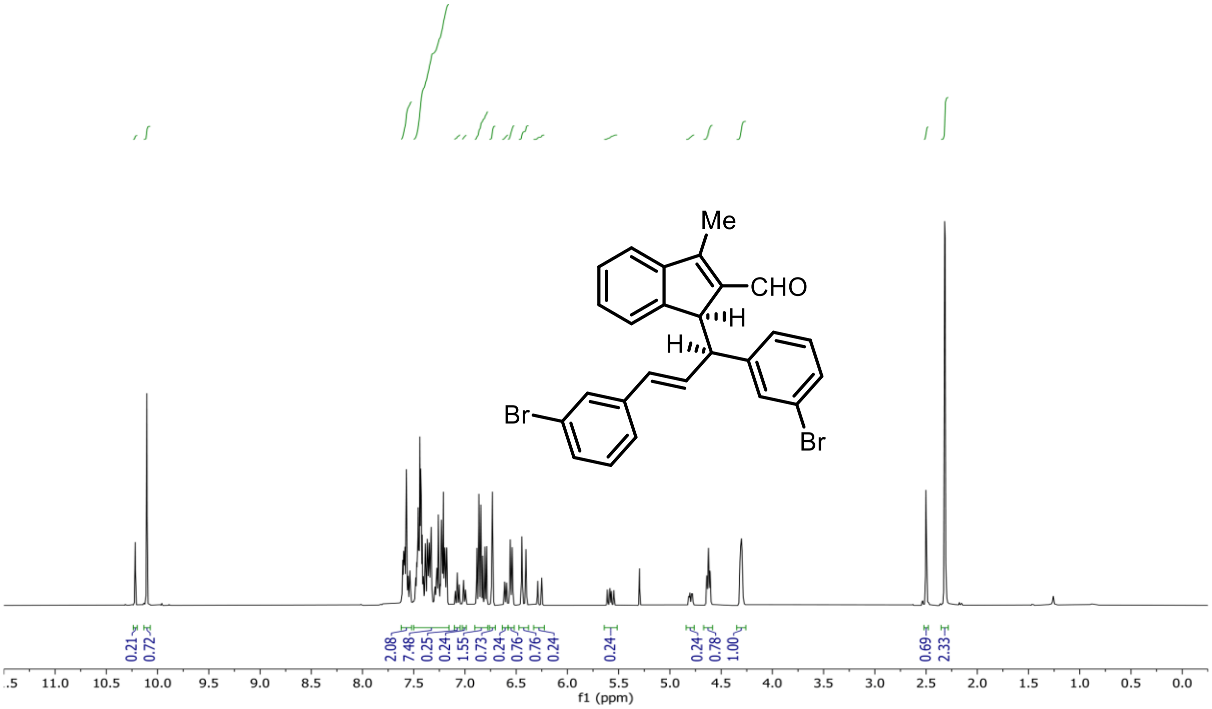

Compound **4v** ^{13}C NMR

(100 MHz, $CDCl_3$)

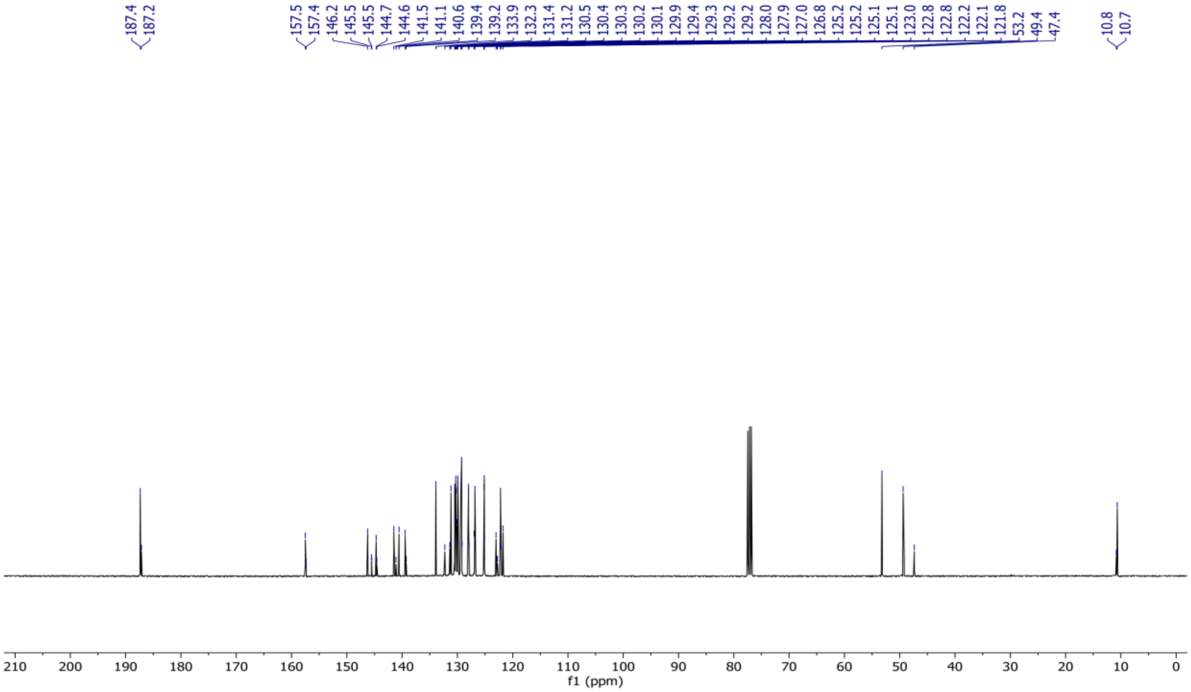

Compound **5v** ^{1}H NMR

(400 MHz, $CDCl_3$)

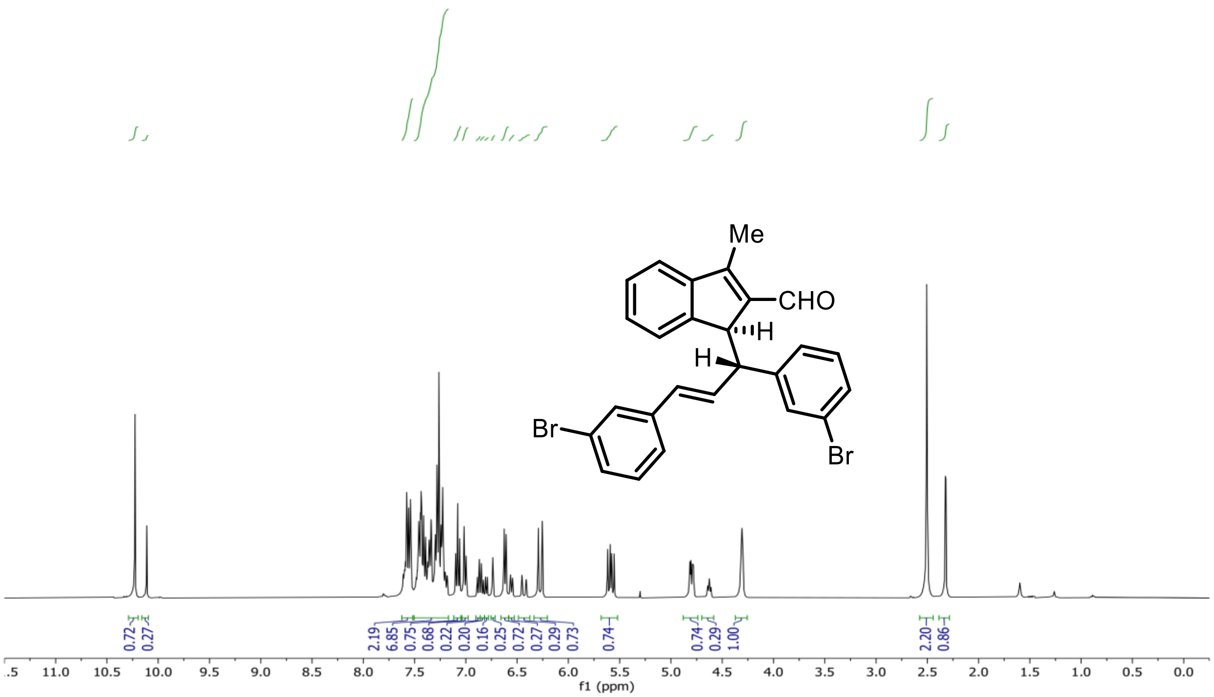

Compound **5v** ^{13}C NMR

(100 MHz, $CDCl_3$)

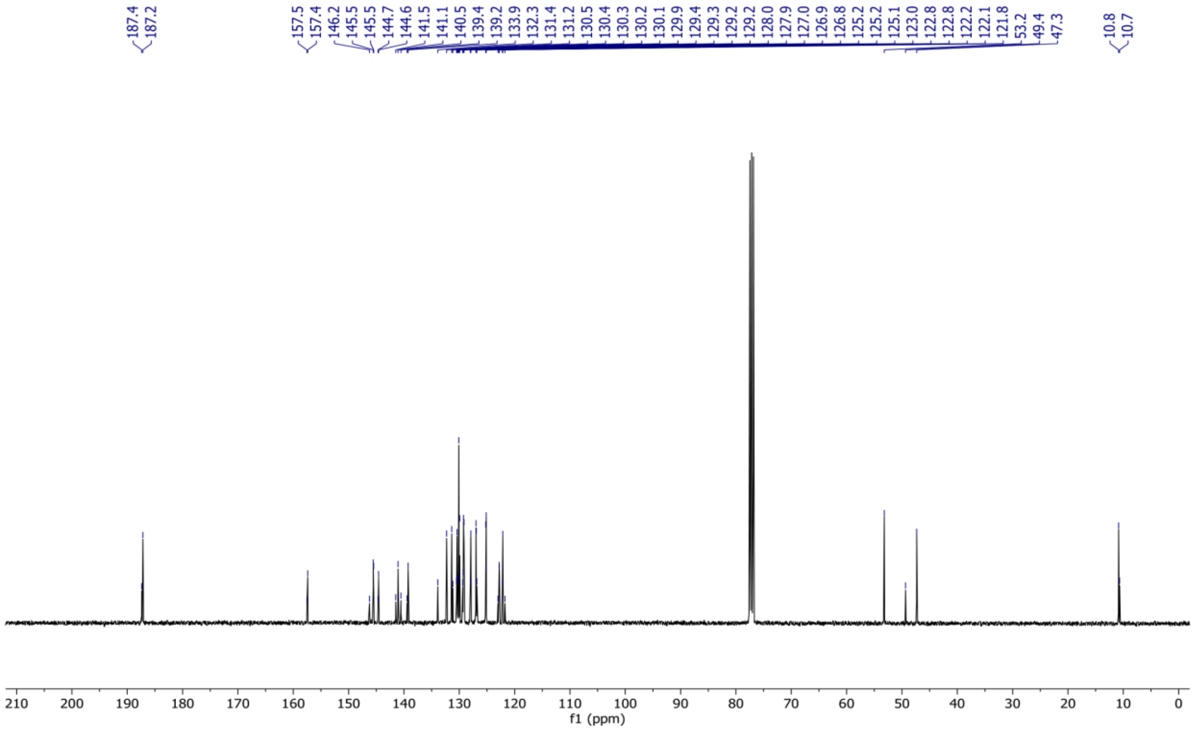

Part 3: UPCC Spectra

Table of Contents

Racemate of **4a**/**5a** S127

Conditions A – Enantioselective (**4a**) S127

Conditions B – Enantioselective (**5a**) S128

Racemate of **4b**/**5b** S129

Conditions A – Enantioselective (**4b**) S129

Conditions B – Enantioselective (**5b**) S130

Racemate of **4c**/**5c** S131

Conditions A – Enantioselective (**4c**) S131

Conditions B – Enantioselective (**5c**) S132

Racemate of **4d**/**5d** S133

Conditions A – Enantioselective (**4d**) S133

Conditions B – Enantioselective (**5d**) S134

Racemate of **4e**/**5e** S135

Conditions A – Enantioselective (**4e**) S135

Conditions B – Enantioselective (**5e**) S136

Racemate of **4f**/**5f** S137

Conditions A – Enantioselective (**4f**) S137

Conditions B – Enantioselective (**5f**) S138

Racemate of **4g**/**5g** S139

Conditions A – Enantioselective (**4g**) S139

Conditions B – Enantioselective (**5g**) S140

Racemate of **4h**/**5h** S141

Conditions A – Enantioselective (**4h**) S141

Conditions B – Enantioselective (**5h**) S142

Racemate of **4i**/**5i** S143

Conditions A – Enantioselective (**4i**) S143

Conditions B – Enantioselective (**5i**) S144

Racemate of **4j**/**5j** S145

Conditions A – Enantioselective (**4j**)........S145
Conditions B – Enantioselective (**5j**)........S146
Racemate of **4k**/**5k**........S147
Conditions A – Enantioselective (**4k**)........S147
Conditions B – Enantioselective (**5k**)........S148
Racemate of **4l**/**5l**........S149
Conditions A – Enantioselective (**4l**)........S149
Conditions B – Enantioselective (**5l**)........S150
Racemate of **4m**/**5m**........S151
Conditions A – Enantioselective (**4m**)........S151
Conditions B – Enantioselective (**5m**)........S152
Racemate of **4n**/**5n**........S153
Conditions A – Enantioselective (**4n**)........S153
Conditions B – Enantioselective (**5n**)........S154
Racemate of **4o**/**5o**........S155
Conditions A – Enantioselective (**4o**)........S155
Conditions B – Enantioselective (**5o**)........S156
Racemate of **4p**/**5p**........S157
Conditions A – Enantioselective (**4p**)........S157
Conditions B – Enantioselective (**5p**)........S158
Racemate of **4q**/**5q**........S159
Conditions A – Enantioselective (**4q**)........S159
Conditions B – Enantioselective (**5q**)........S160
Racemate of **4r**/**5r**........S161
Conditions A – Enantioselective (**4r**)........S161
Conditions B – Enantioselective (**5r**)........S162
Racemate of **4s**/**5s**........S163
Conditions A – Enantioselective (**4s**)........S163
Conditions B – Enantioselective (**5s**)........S164
Racemate of **4t**/**5t**........S165
Conditions A – Enantioselective (**4t**)........S165

Conditions B – Enantioselective (**5t**) S166
Racemate of **4u**/**5u** S167
Conditions A – Enantioselective (**4u**) S167
Conditions B – Enantioselective (**5u**) S168
Racemate of **4v**/**5v** S169
Conditions A – Enantioselective (**4v**) S169
Conditions B – Enantioselective (**5v**) S170

Racemate of **4a**/**5a**

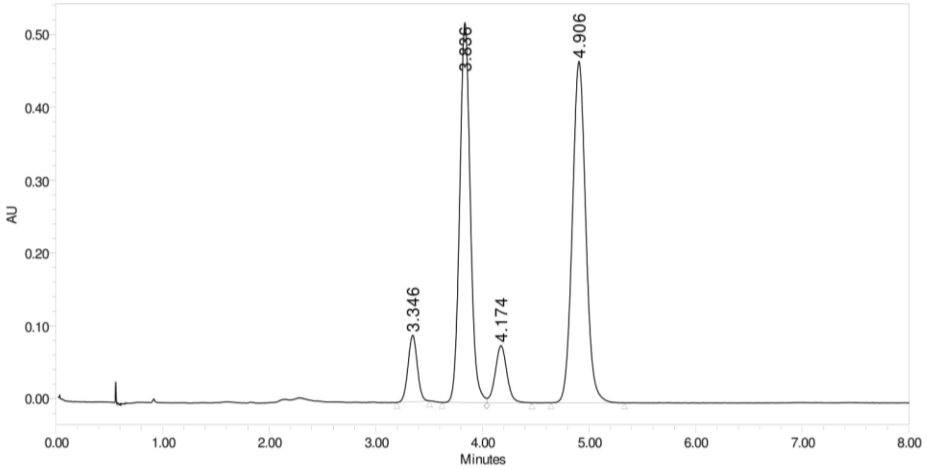

	Retention Time (min)	% Area
1	3.346	6.22
2	3.836	40.79
3	4.174	6.70
4	4.906	46.28

Conditions A – Enantioselective (**4a**)

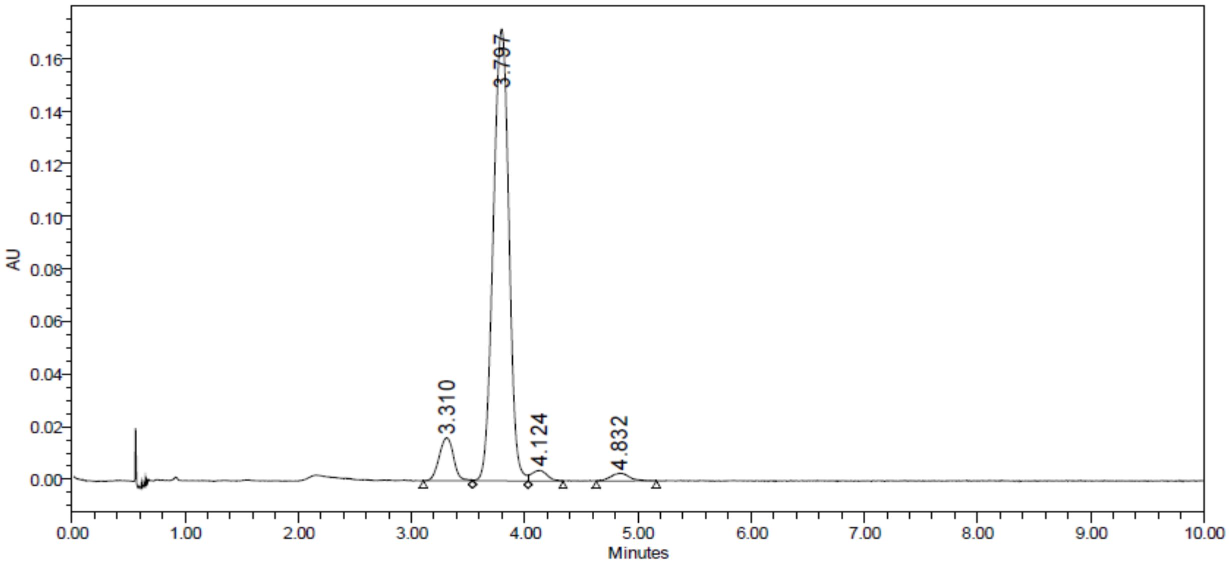

	Retention Time (min)	% Area
1	3.310	7.69
2	3.797	88.33
3	4.124	2.12
4	4.832	1.87

Conditions B – Enantioselective (**5a**)

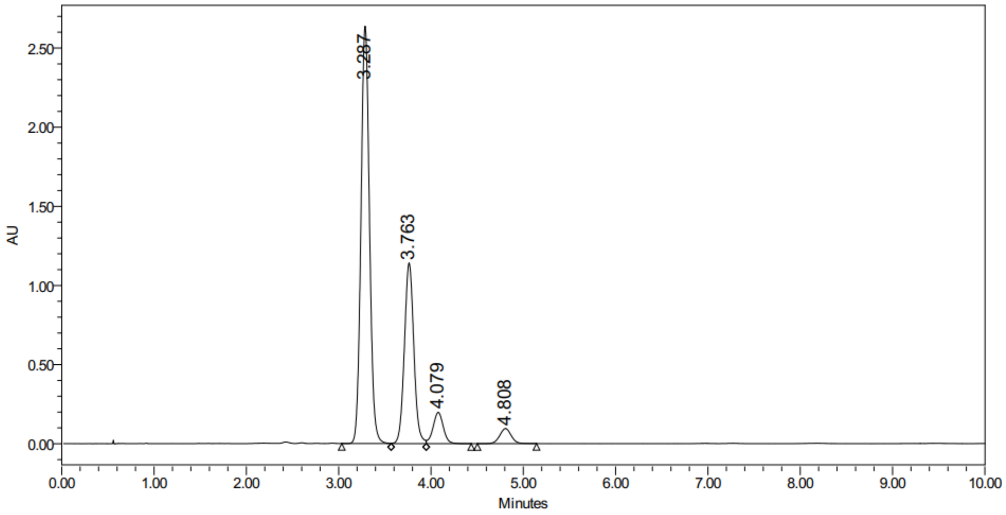

	Retention Time (min)	% Area
1	3.287	61.83
2	3.763	29.62
3	4.079	5.55
4	4.808	3.00

Racemate of **4b**/**5b**

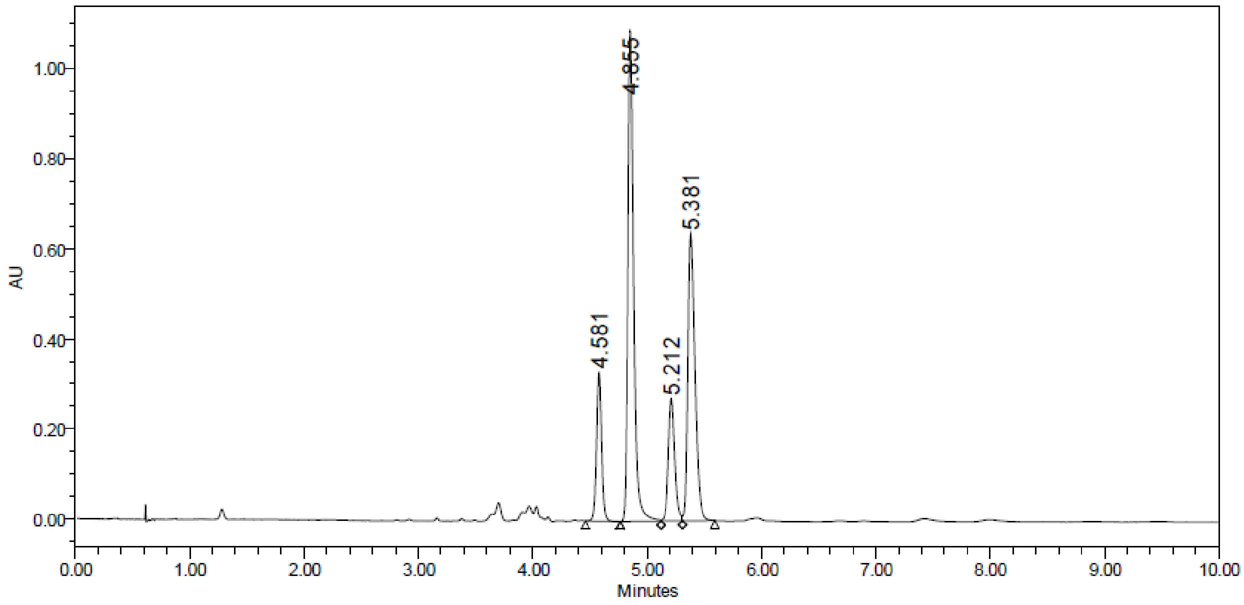

	Retention Time (min)	% Area
1	4.581	11.60
2	4.855	44.62
3	5.212	12.22
4	5.381	31.56

Conditions A – Enantioselective (**4b**)

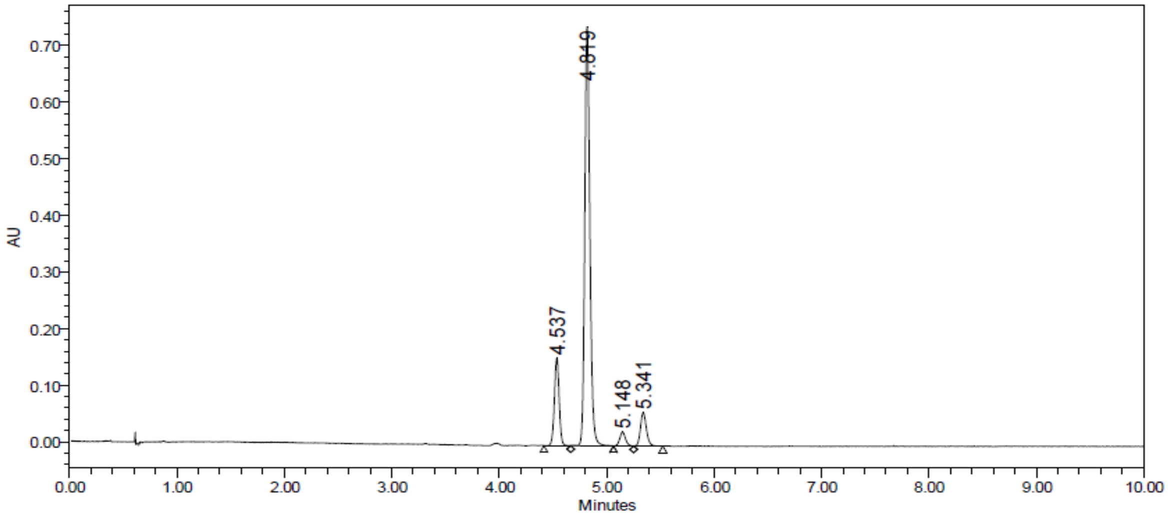

	Retention Time (min)	% Area
1	4.537	14.63
2	4.819	75.62
3	5.148	2.80
4	5.341	6.95

Conditions B – Enantioselective (**5b**)

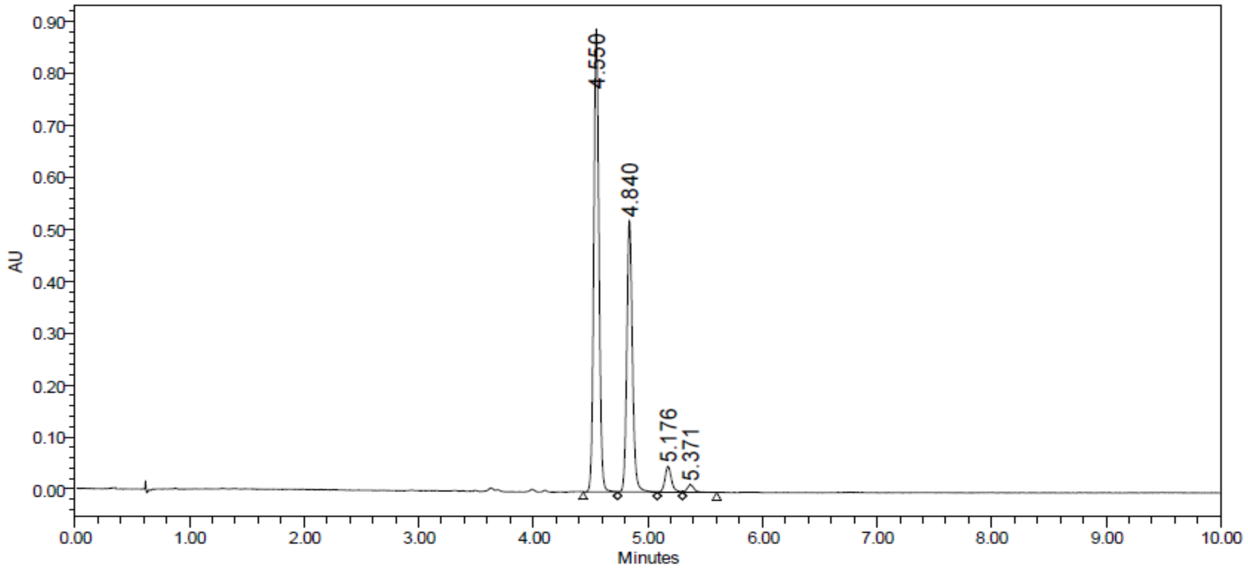

	Retention Time (min)	% Area
1	4.550	57.67
2	4.840	36.98
3	5.176	4.11
4	5.371	1.24

Racemate of **4c**/**5c**

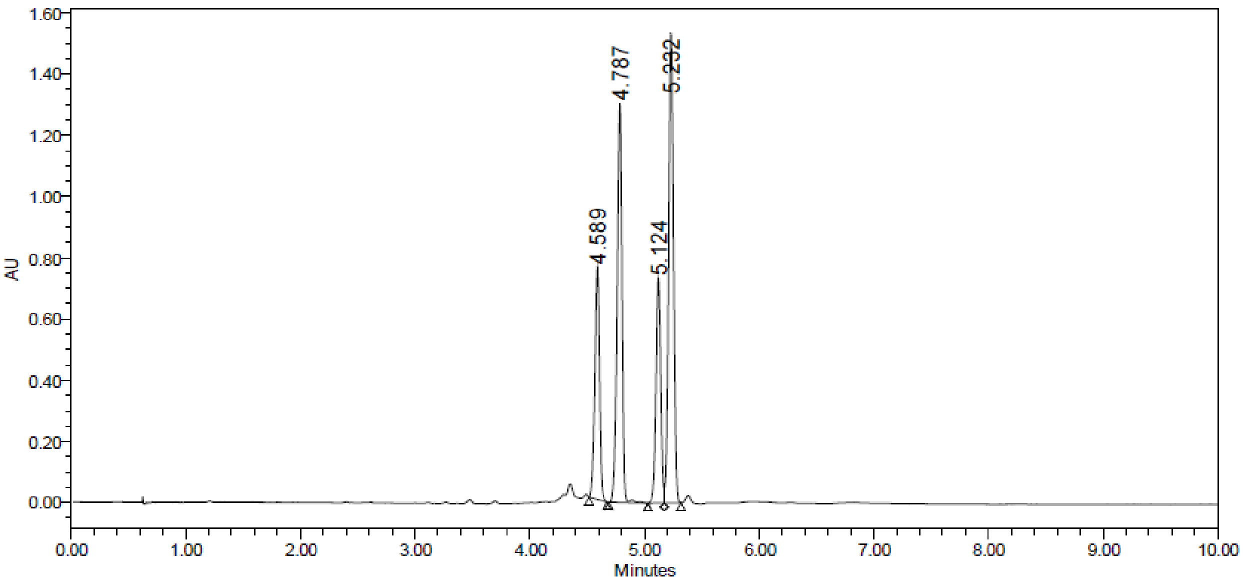

	Retention Time (min)	% Area
1	4.589	17.22
2	4.787	30.41
3	5.124	16.61
4	5.232	35.76

Conditions A – Enantioselective (**4c**)

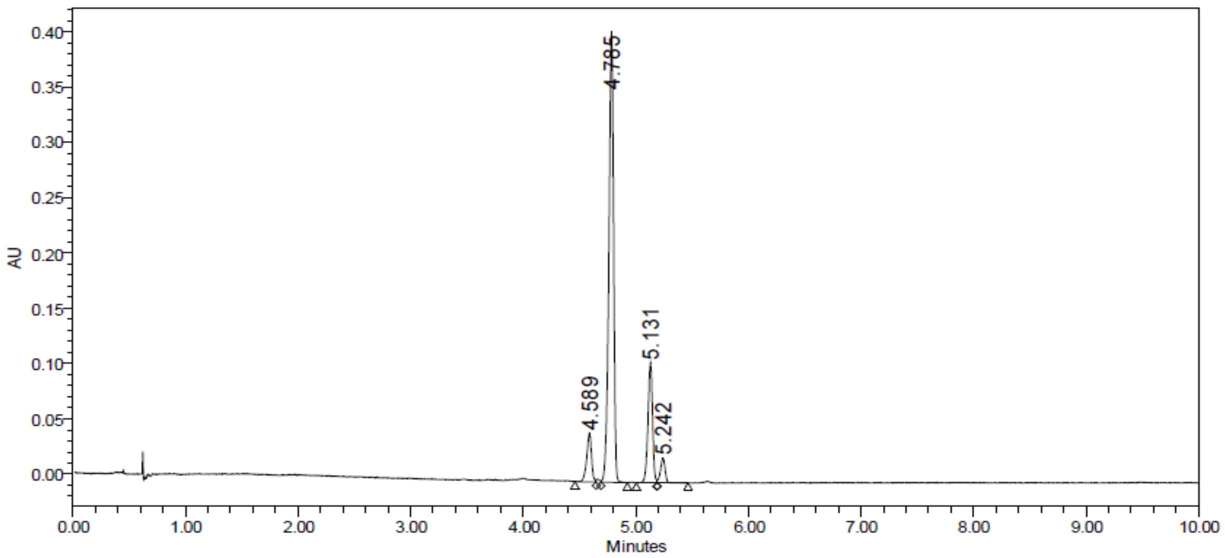

	Retention Time (min)	% Area
1	4.589	7.72
2	4.785	70.51
3	5.131	18.05
4	5.242	3.72

Conditions B – Enantioselective (**5c**)

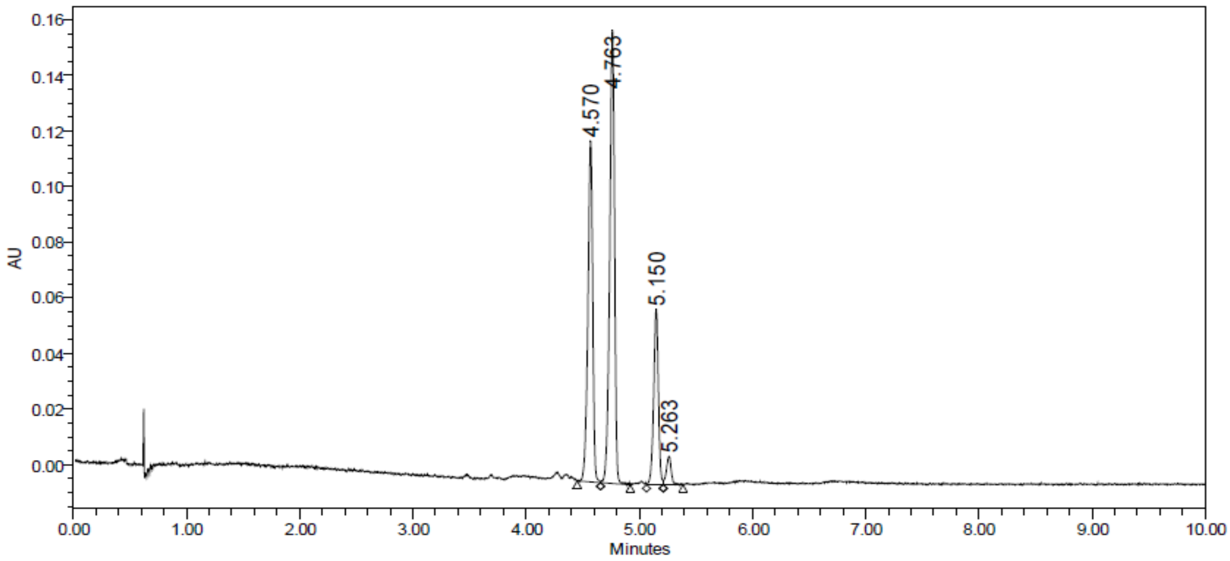

	Retention Time (min)	% Area
1	5.150	17.16
2	5.263	2.85
3	4.570	34.07
4	4.763	45.92

Racemate of **4d**/**5d**

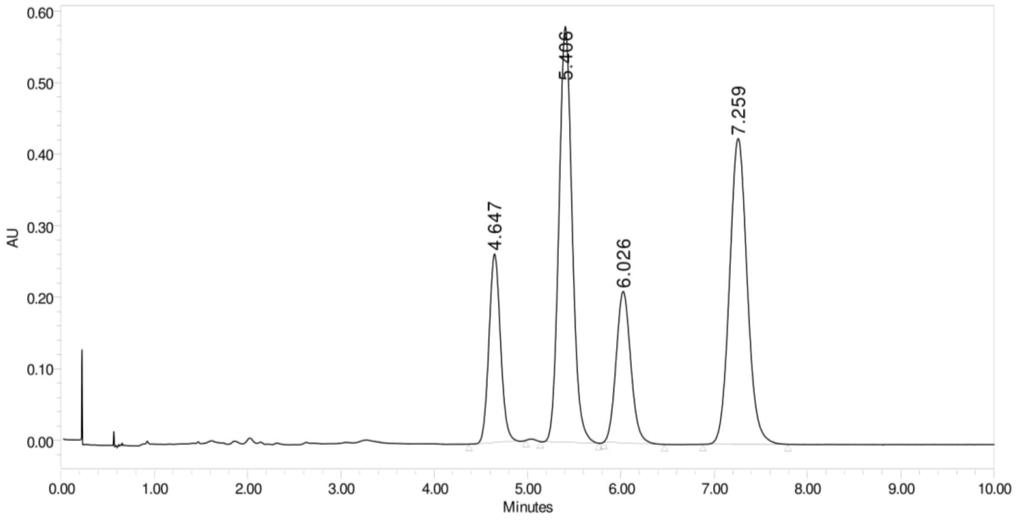

	Retention Time (min)	% Area
1	4.647	14.20
2	5.406	35.97
3	6.026	14.48
4	7.259	35.35

Conditions A – Enantioselective (**4d**)

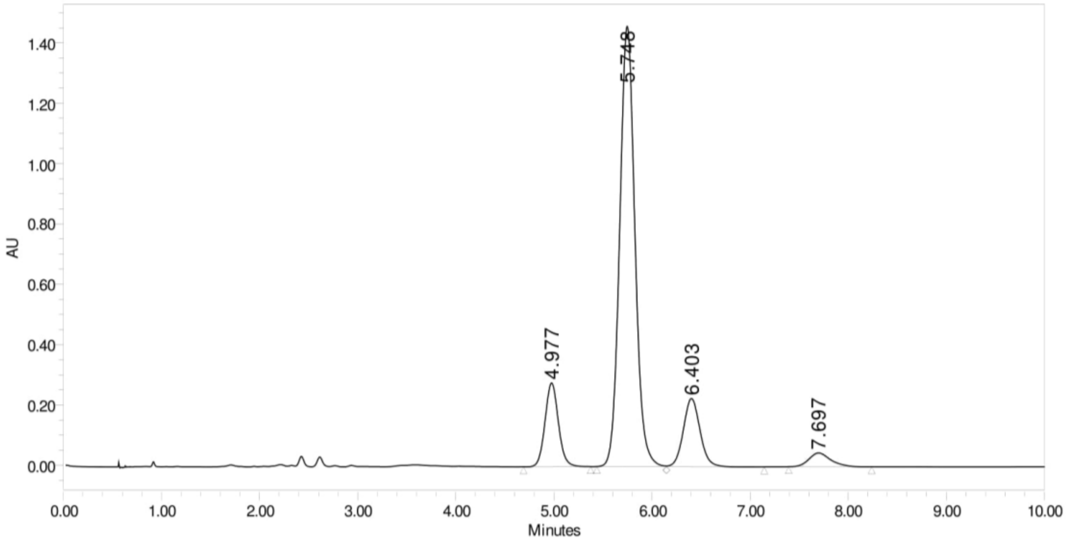

	Retention Time (min)	% Area
1	6.403	12.27
2	7.697	3.21
3	4.977	12.10
4	5.748	72.42

Conditions B – Enantioselective (**5d**)

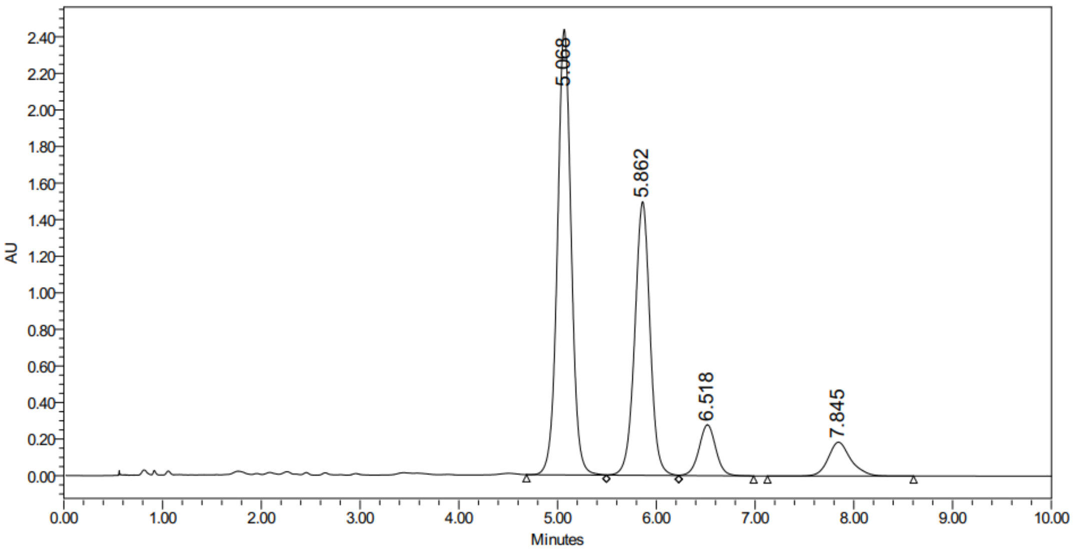

	Retention Time (min)	% Area
1	5.068	51.37
2	5.862	35.05
3	6.518	7.19
4	7.845	6.39

Racemate of **4e**/**5e**

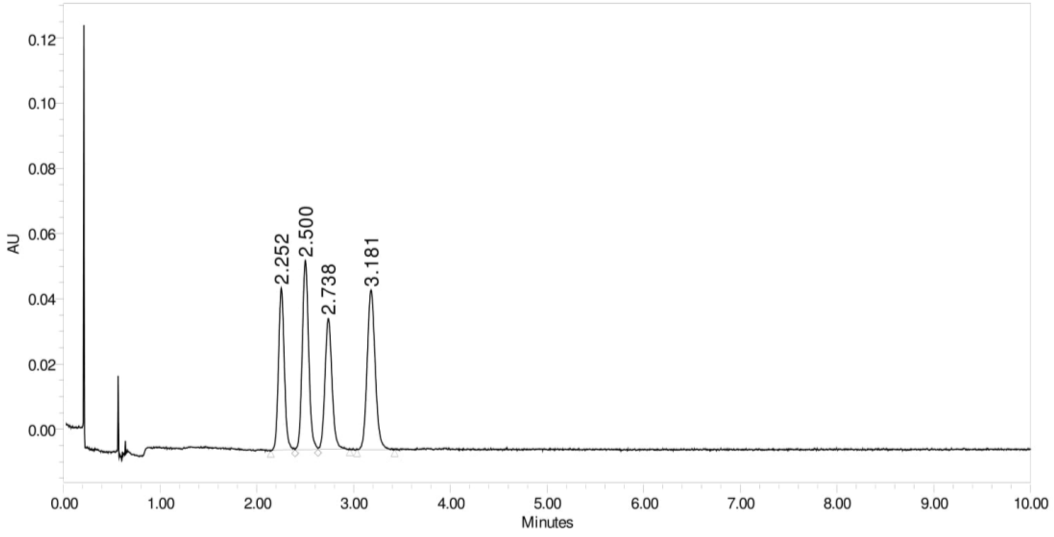

	Retention Time (min)	% Area
1	2.252	21.67
2	2.500	27.79
3	2.738	21.07
4	3.181	29.47

Conditions A – Enantioselective (**4e**)

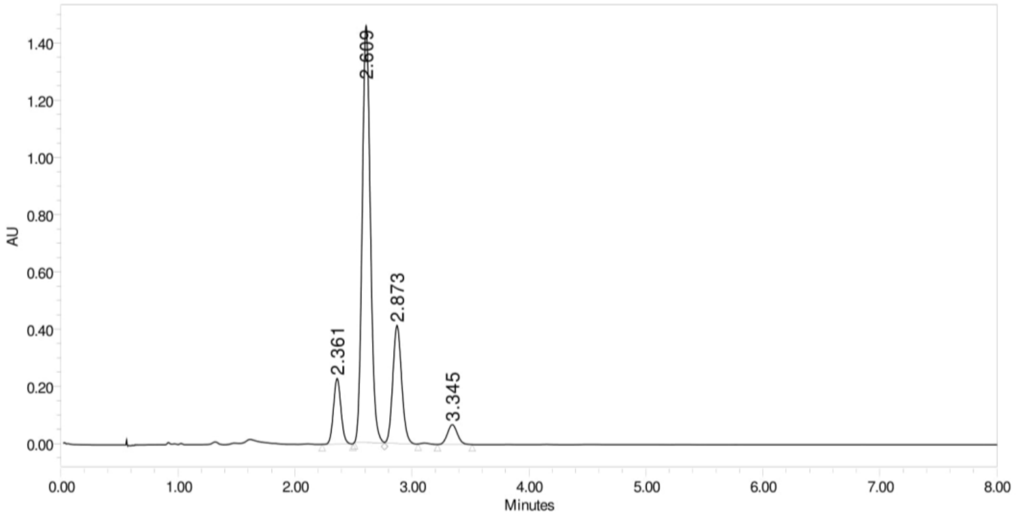

	Retention Time (min)	% Area
1	2.361	9.50
2	2.609	66.28
3	2.873	20.36
4	3.345	3.86

Conditions B – Enantioselective (**5e**)

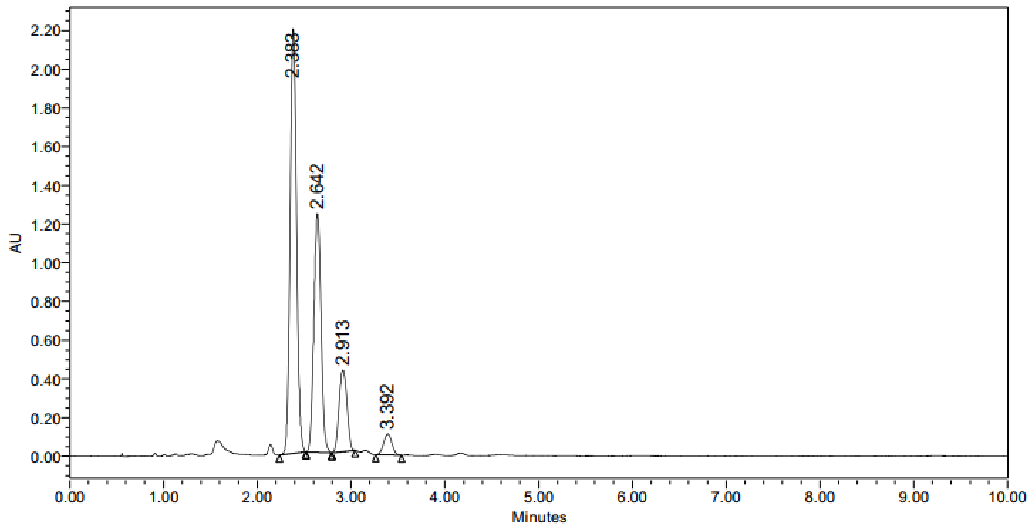

	Retention Time (min)	% Area
1	2.383	52.72
2	2.642	32.00
3	2.913	11.97
4	3.392	3.31

Racemate of **4f**/**5f**

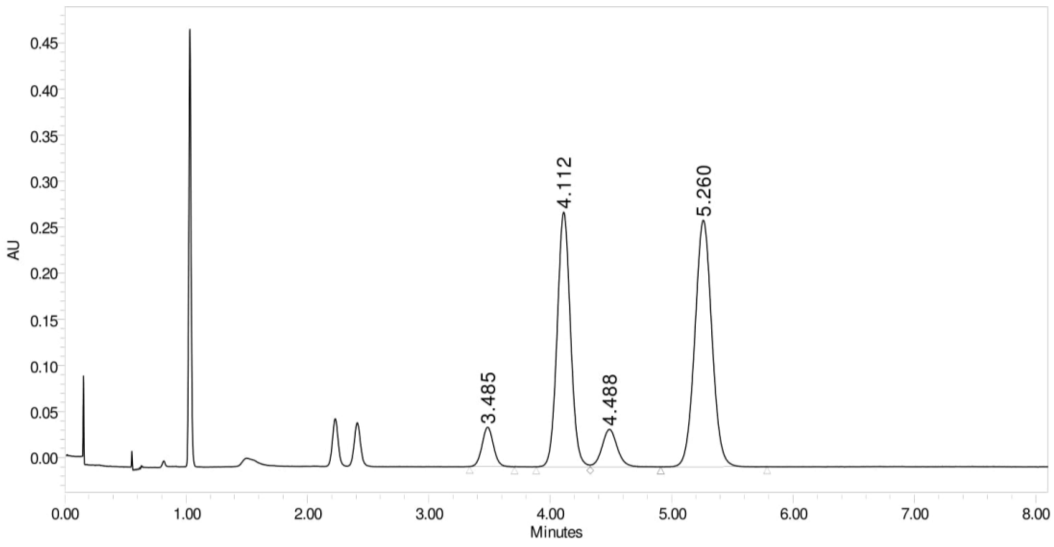

	Retention Time (min)	% Area
1	3.485	5.26
2	4.112	39.36
3	4.488	6.67
4	5.260	48.71

Conditions A – Enantioselective (**4f**)

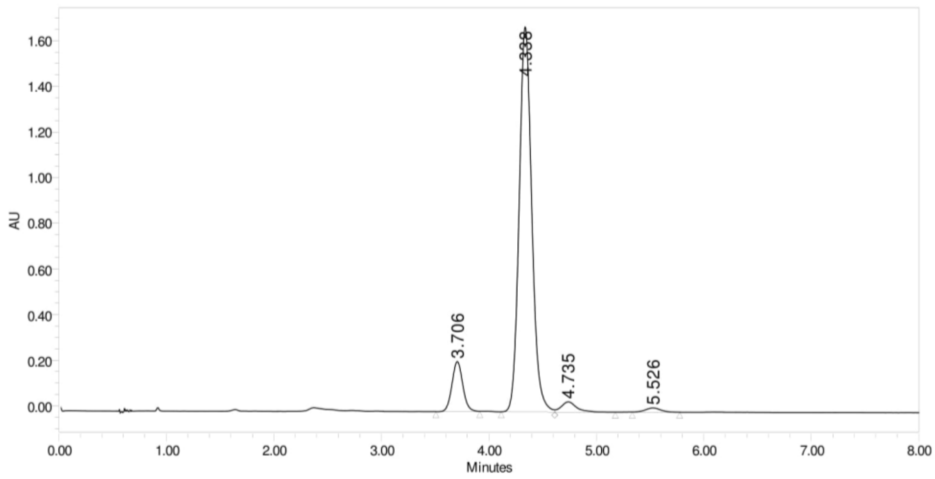

	Retention Time (min)	% Area
1	3.706	9.63
2	4.338	86.55
3	4.735	2.74
4	5.526	1.09

Conditions B – Enantioselective (**5f**)

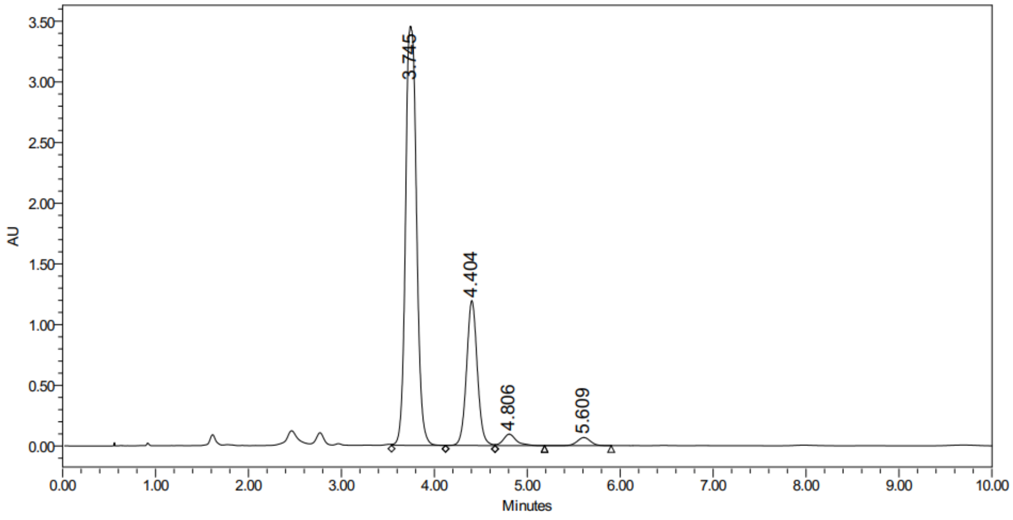

	Retention Time (min)	% Area
1	3.745	71.64
2	4.404	24.39
3	4.806	2.30
4	5.609	1.67

Racemate of **4g**/**5g**

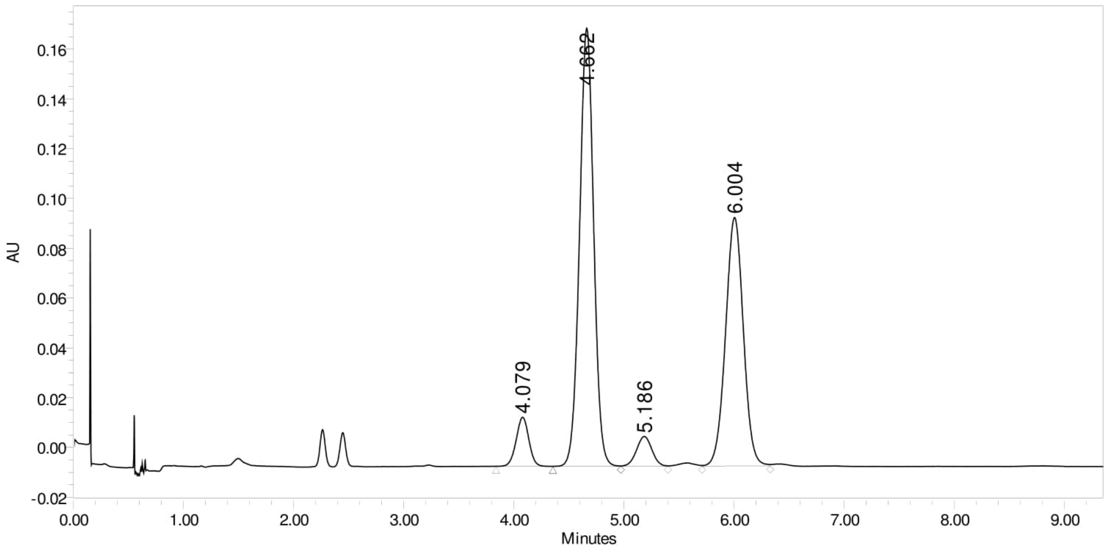

	Retention Time (min)	% Area
1	4.079	5.32
2	4.662	52.99
3	5.186	3.90
4	6.004	37.78

Conditions A – Enantioselective (**4g**)

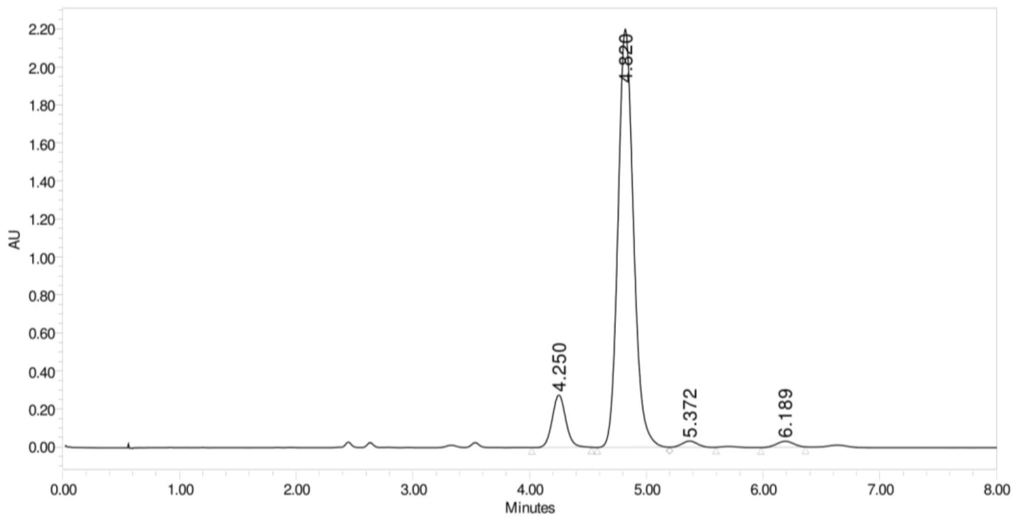

	Retention Time (min)	% Area
1	4.250	9.63
2	4.820	87.74
3	5.372	1.29
4	6.189	1.35

Conditions B – Enantioselective (**5g**)

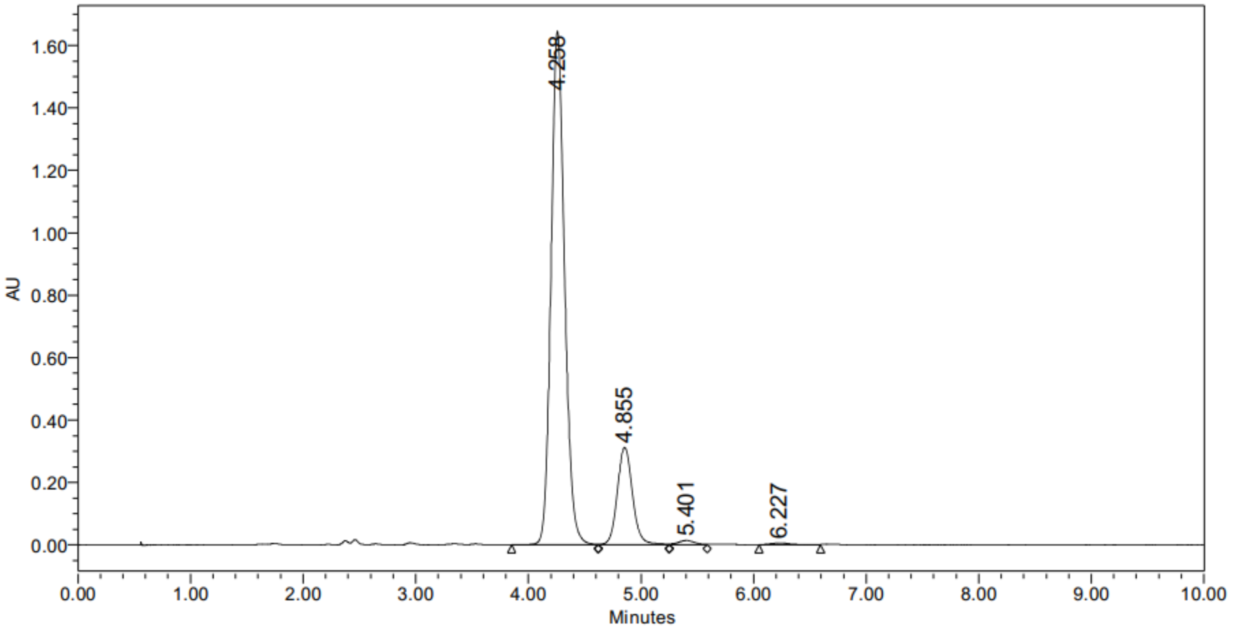

	Retention Time (min)	% Area
1	4.258	81.68
2	4.855	17.24
3	5.401	0.78
4	6.227	0.30

Racemate of **4h**/**5h**

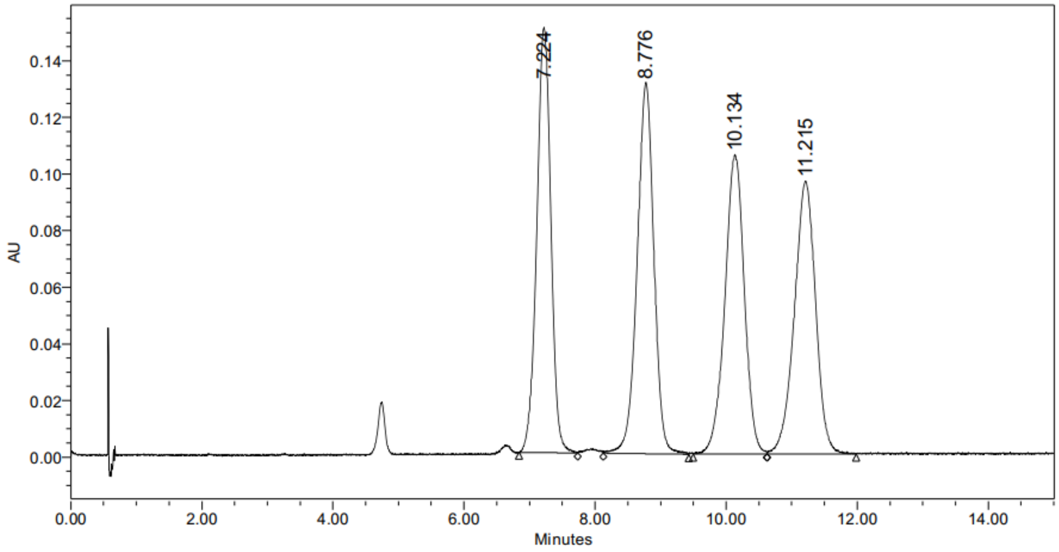

	Retention Time (min)	% Area
1	7.224	25.04
2	8.776	26.16
3	10.134	24.47
4	11.215	24.33

Conditions A – Enantioselective (**4h**)

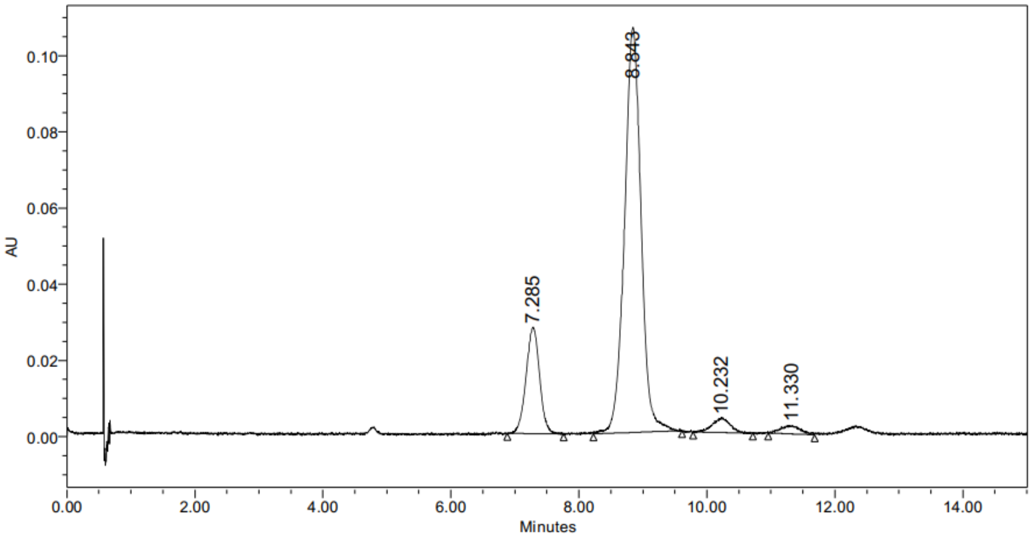

	Retention Time (min)	% Area
1	7.285	16.79
2	8.843	78.31
3	10.232	3.00
4	11.330	1.90

Conditions B – Enantioselective (**5h**)

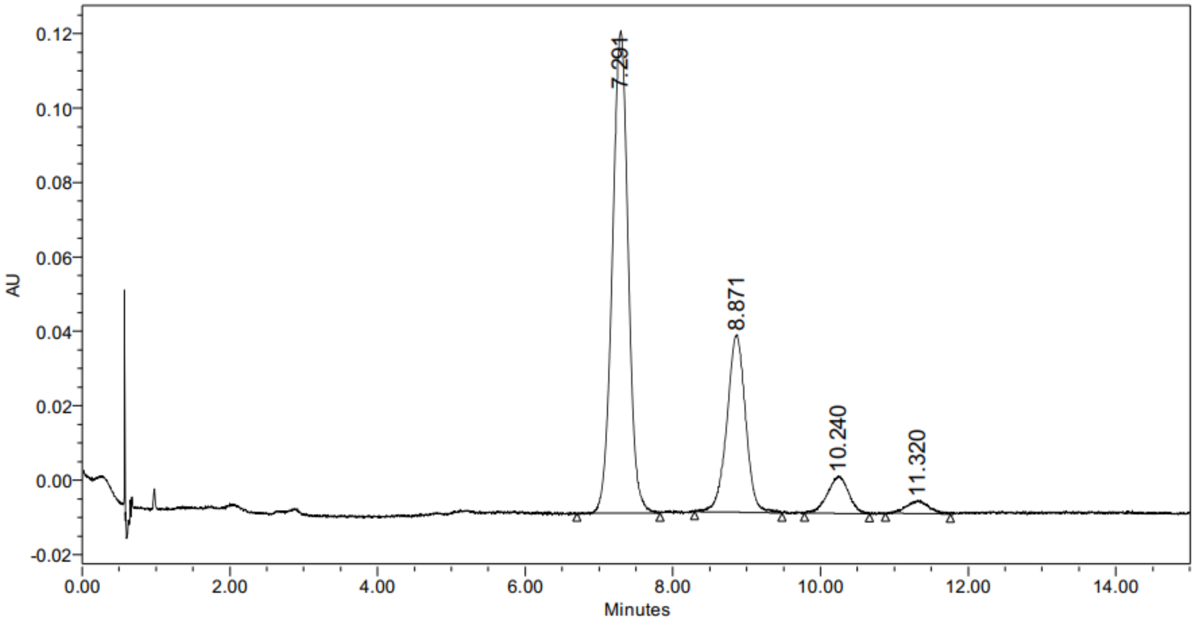

	Retention Time (min)	% Area
1	7.291	63.48
2	8.871	27.65
3	10.240	6.47
4	11.320	2.40

Racemate of **4i**/**5i**

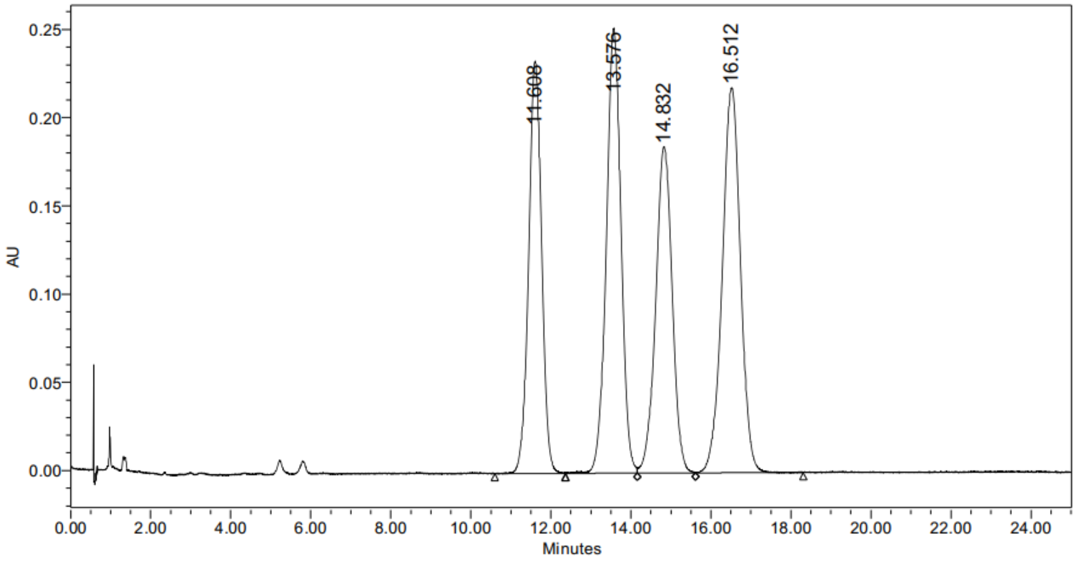

	Retention Time (min)	% Area
1	11.608	21.99
2	13.576	26.90
3	14.832	22.24
4	16.512	28.87

Conditions A – Enantioselective (**4i**)

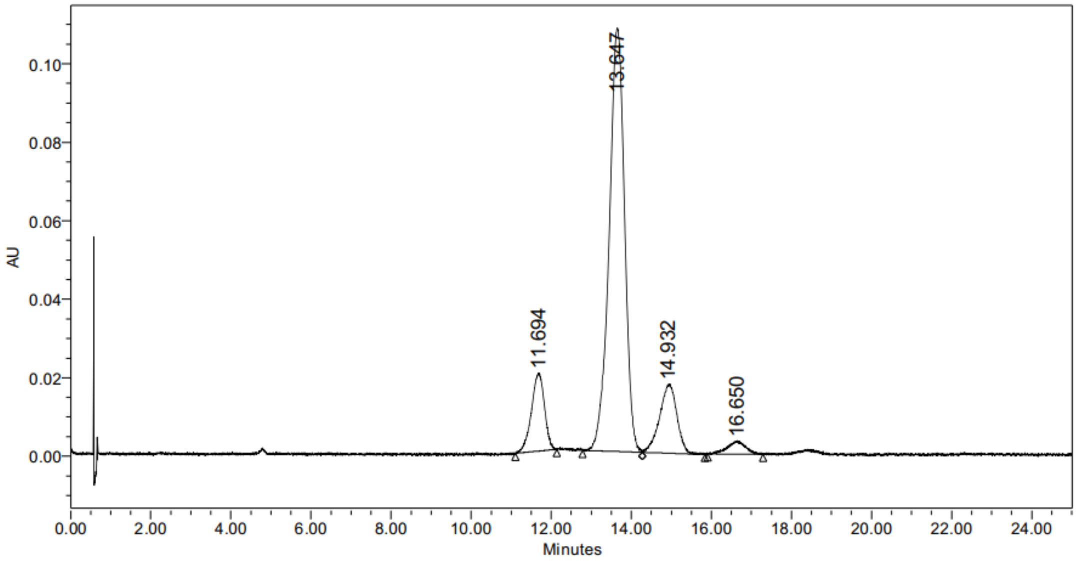

	Retention Time (min)	% Area
1	11.694	11.30
2	13.647	72.72
3	14.932	13.34
4	16.650	2.64

Conditions B – Enantioselective (**5i**)

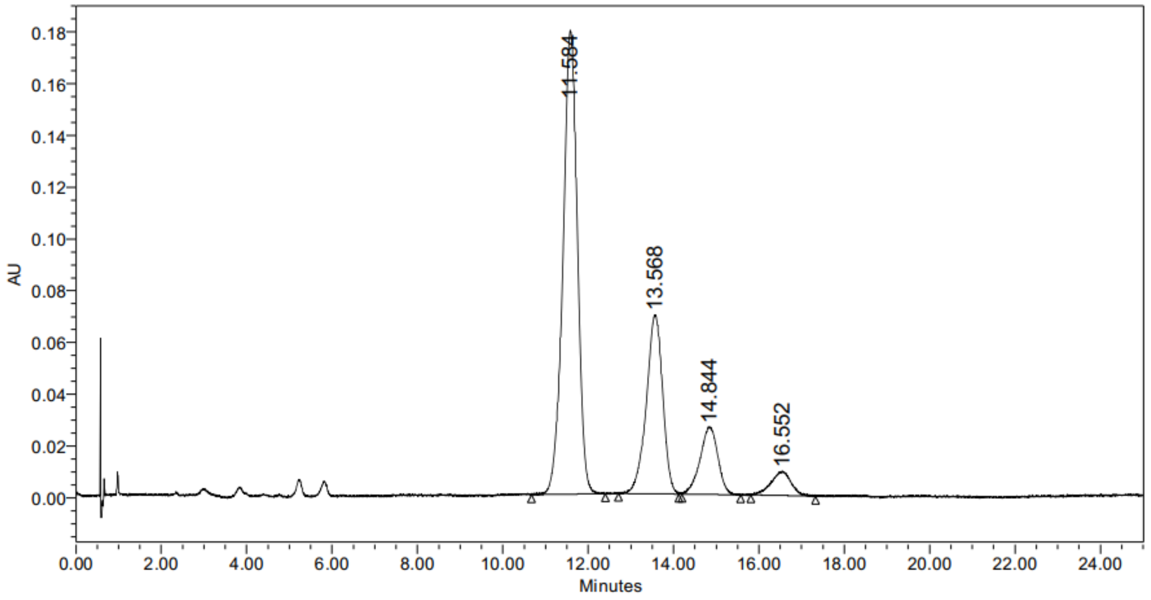

	Retention Time (min)	% Area
1	11.584	59.12
2	13.568	25.94
3	14.844	10.65
4	16.552	4.29

Racemate of **4j**/**5j**

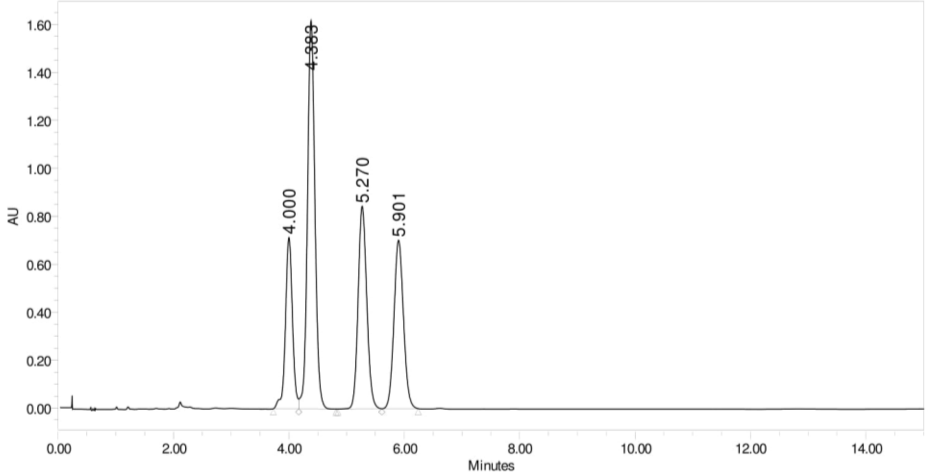

	Retention Time (min)	% Area
1	4.000	16.06
2	4.383	38.76
3	5.270	23.58
4	5.901	21.60

Conditions A – Enantioselective (**4j**)

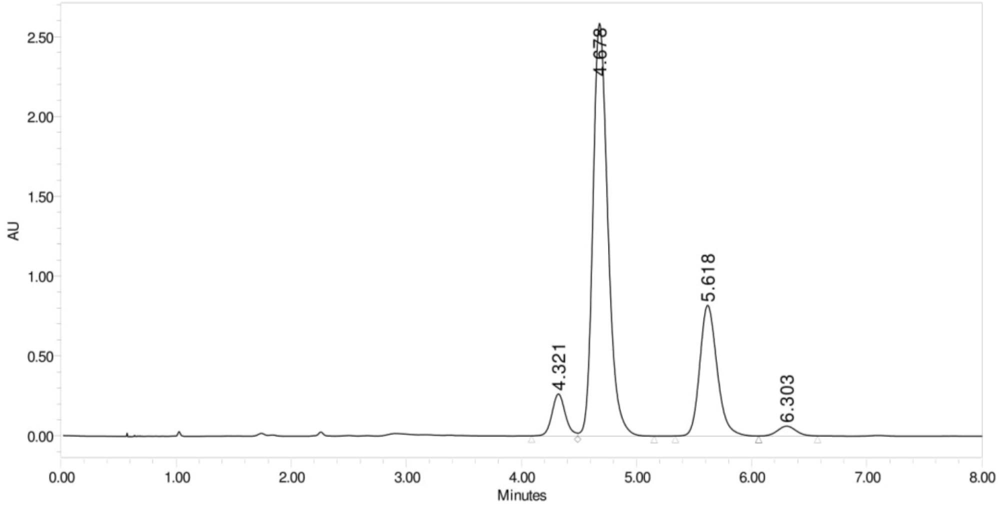

	Retention Time (min)	% Area
1	4.321	6.09
2	4.678	67.27
3	5.618	24.67
4	6.303	1.97

Conditions B – Enantioselective (**5j**)

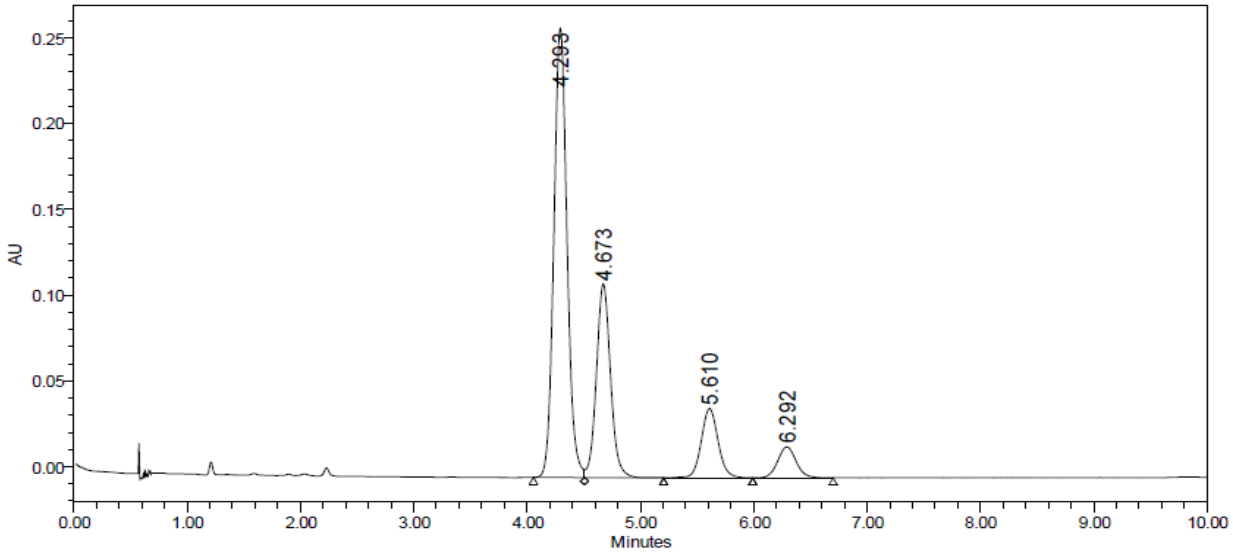

	Retention Time (min)	% Area
1	4.293	56.87
2	4.673	26.38
3	5.610	11.26
4	6.292	5.49

Racemate of **4k**/**5k**

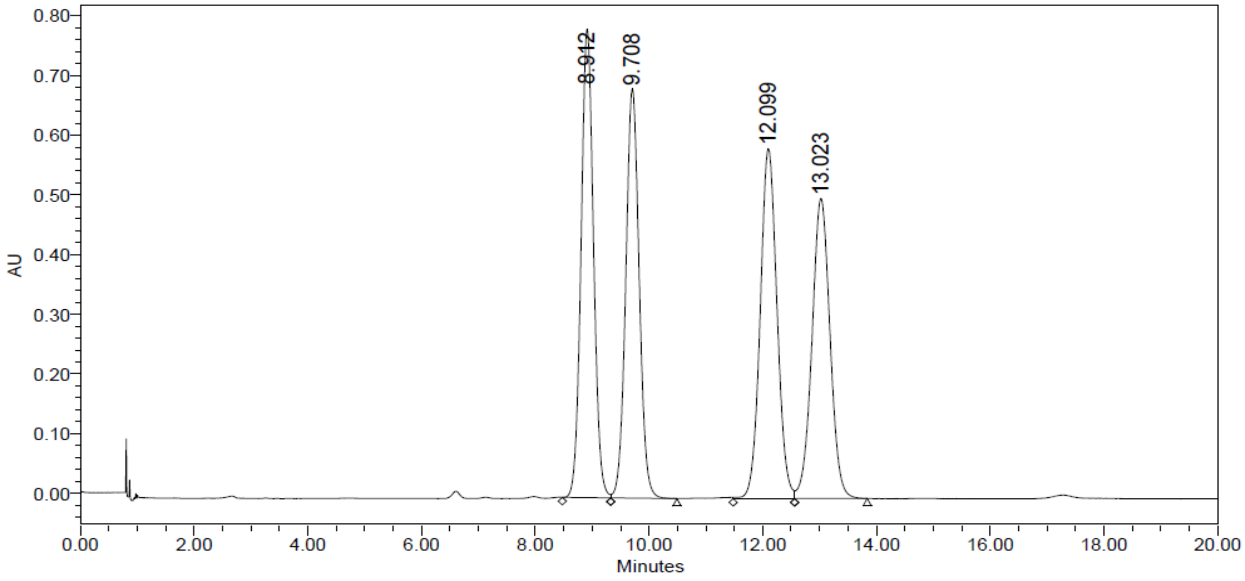

	Retention Time (min)	% Area
1	12.099	25.94
2	13.023	23.97
3	8.912	25.77
4	9.708	24.32

Conditions A – Enantioselective (**4k**)

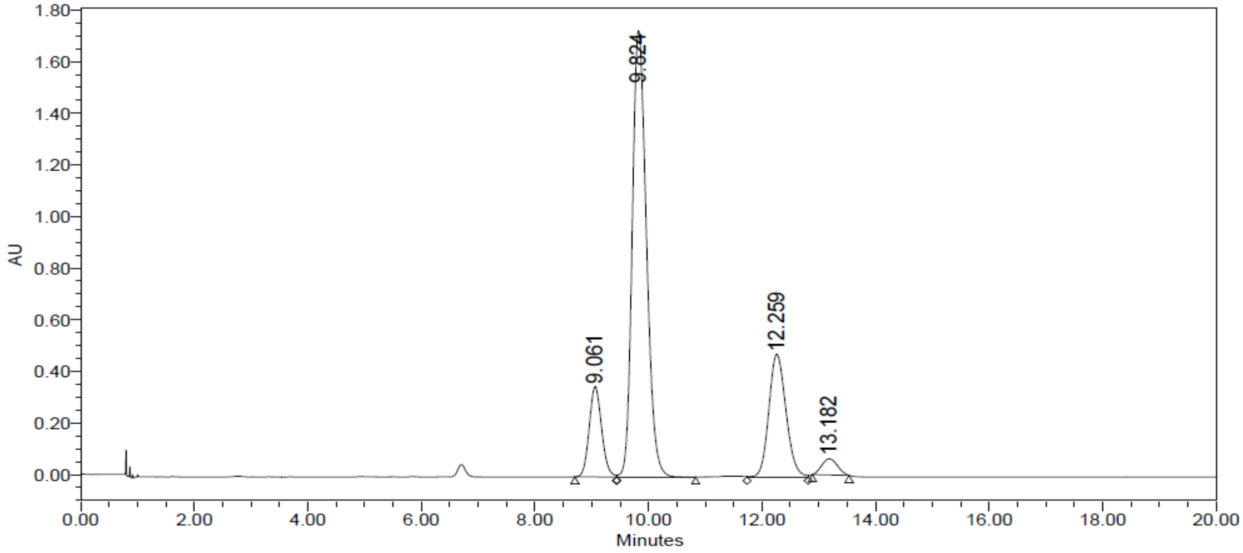

	Retention Time (min)	% Area
1	9.061	11.43
2	9.824	64.52
3	12.259	21.46
4	13.182	2.59

Conditions B – Enantioselective (**5k**)

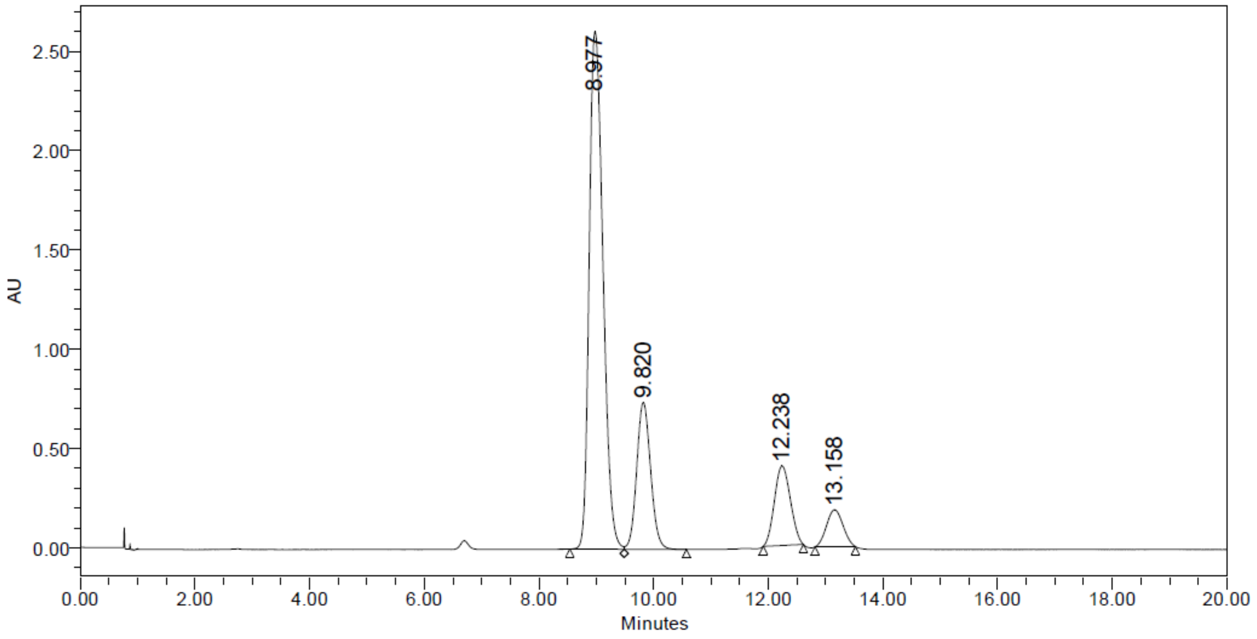

	Retention Time (min)	% Area
1	8.977	64.51
2	9.820	18.52
3	12.238	11.46
4	13.158	5.51

Racemate of **4l**/**5l**

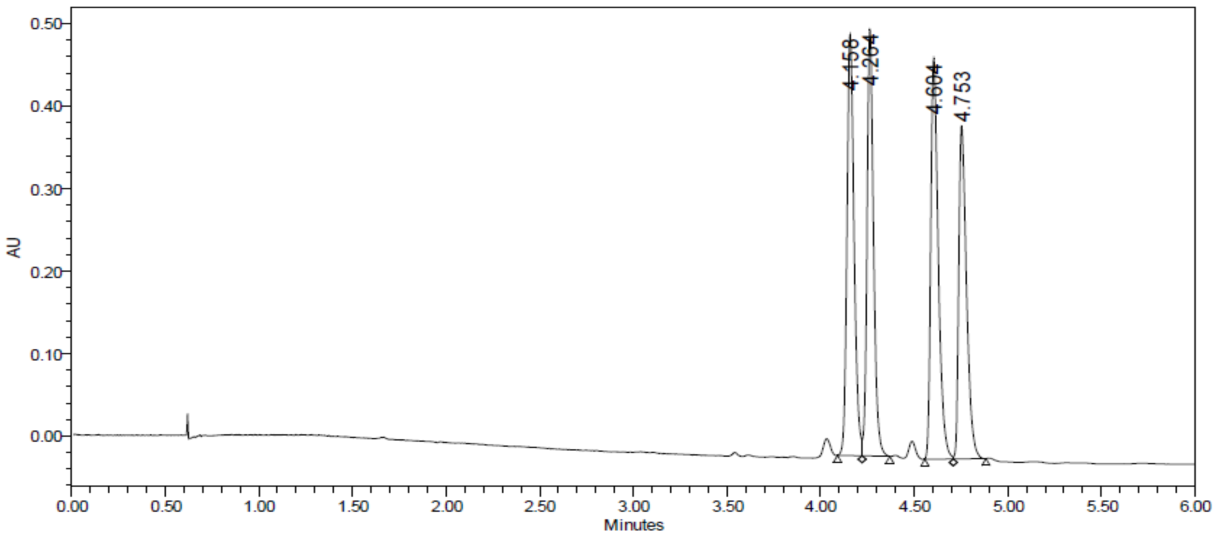

	Retention Time (min)	% Area
1	4.158	26.28
2	4.264	24.43
3	4.604	26.53
4	4.753	22.76

Conditions A – Enantioselective (**4l**)

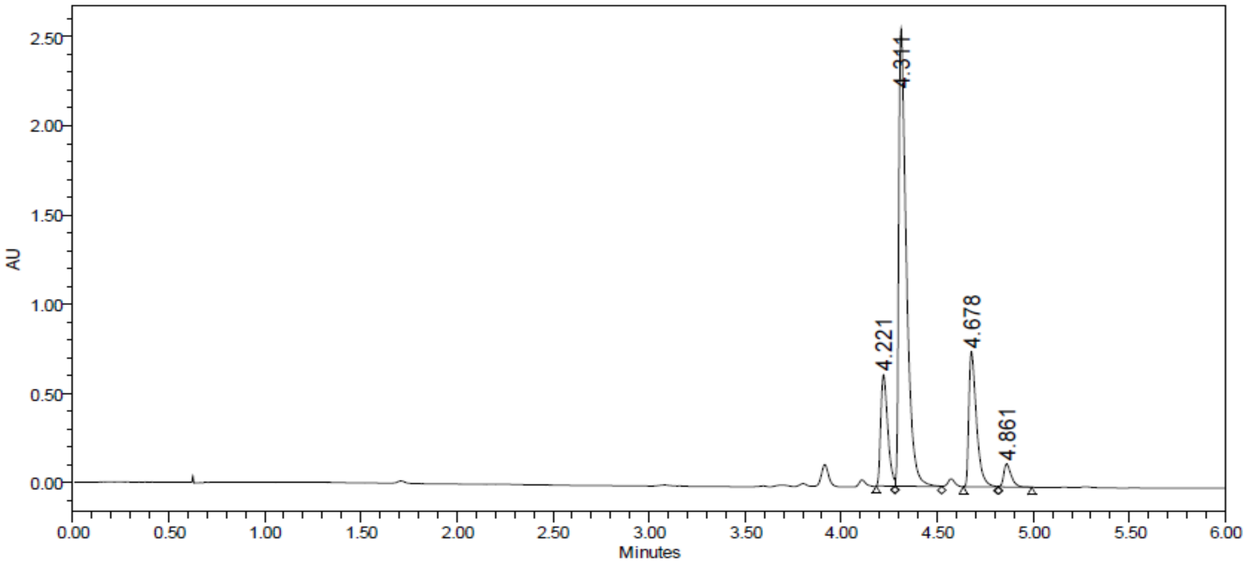

	Retention Time (min)	% Area
1	4.221	14.13
2	4.311	63.90
3	4.678	18.77
4	4.861	3.20

Conditions B – Enantioselective (**5l**)

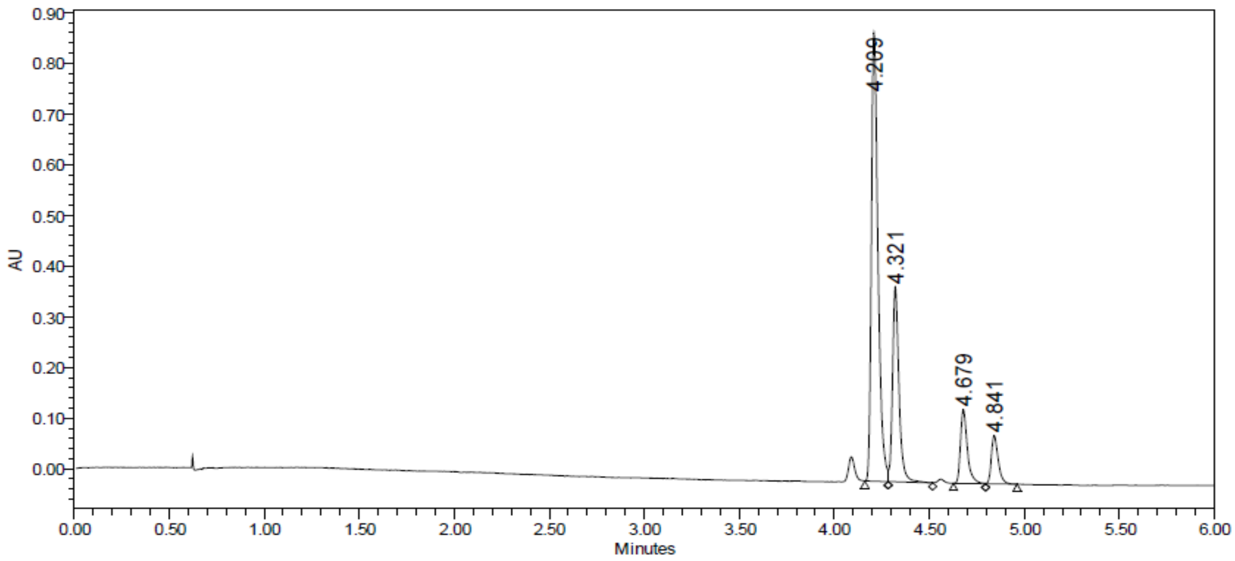

	Retention Time (min)	% Area
1	4.209	58.67
2	4.321	24.81
3	4.679	10.02
4	4.841	6.50

Racemate of **4m/5m**

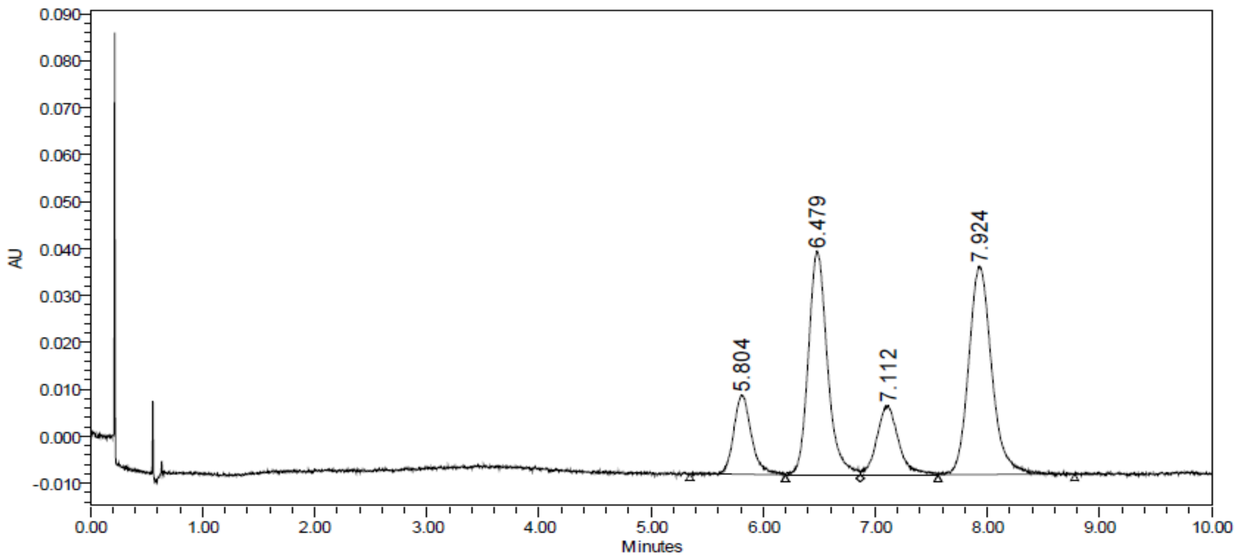

	Retention Time (min)	% Area
1	5.804	11.77
2	6.479	35.52
3	7.112	12.48
4	7.924	40.23

Conditions A – Enantioselective (**4m**)

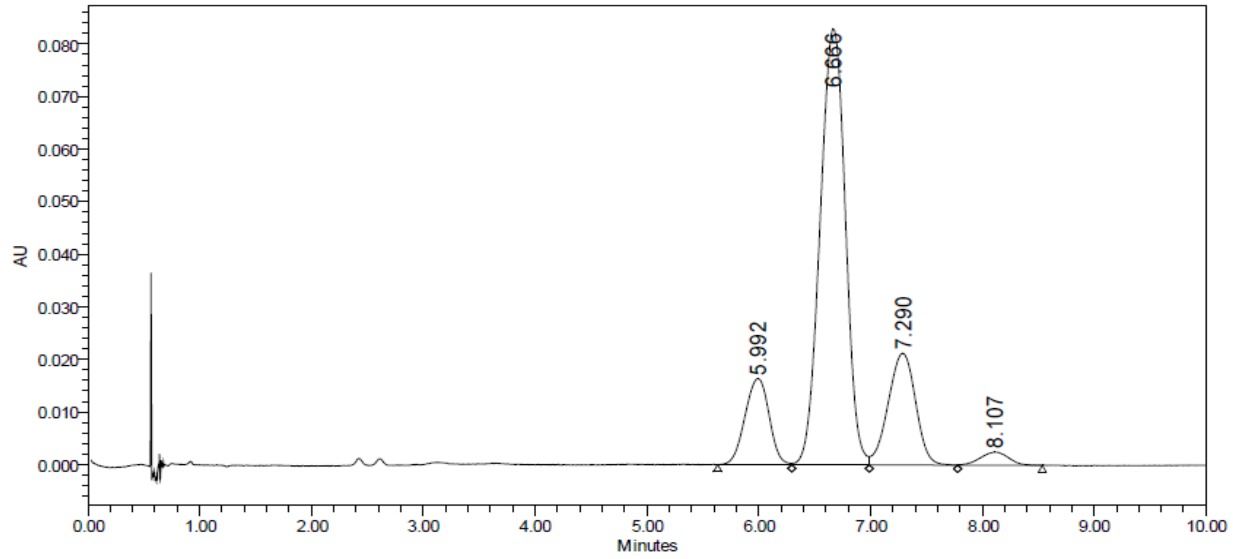

	Retention Time (min)	% Area
1	5.992	12.55
2	6.666	66.66
3	7.290	18.55
4	8.107	2.24

Conditions B – Enantioselective (**5m**)

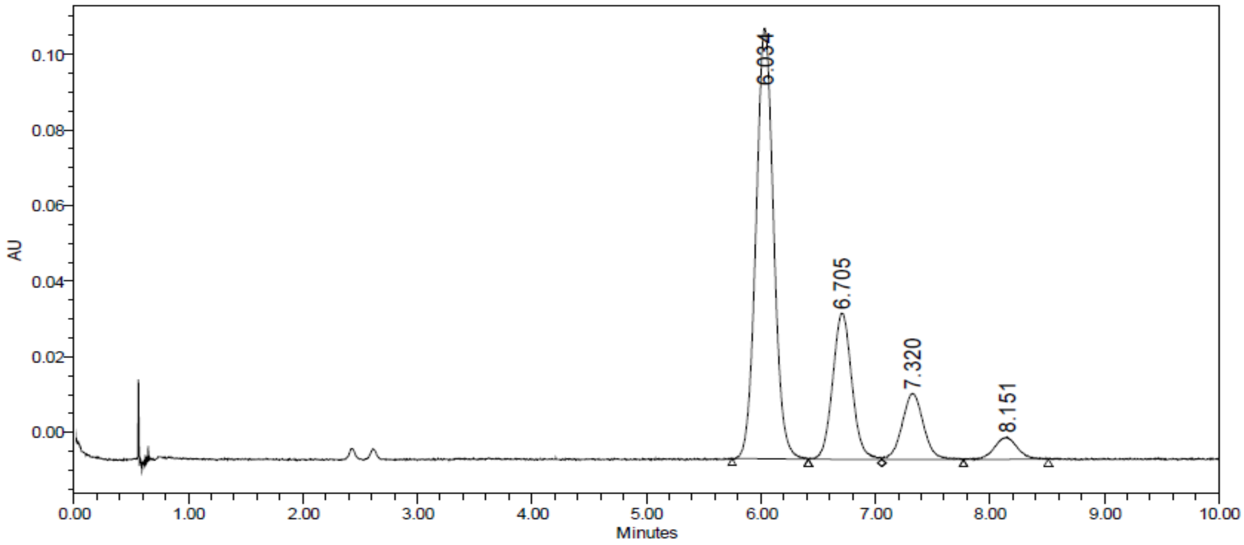

	Retention Time (min)	% Area
1	6.034	61.60
2	6.705	22.76
3	7.320	11.50
4	8.151	4.13

Racemate of **4n**/**5n**

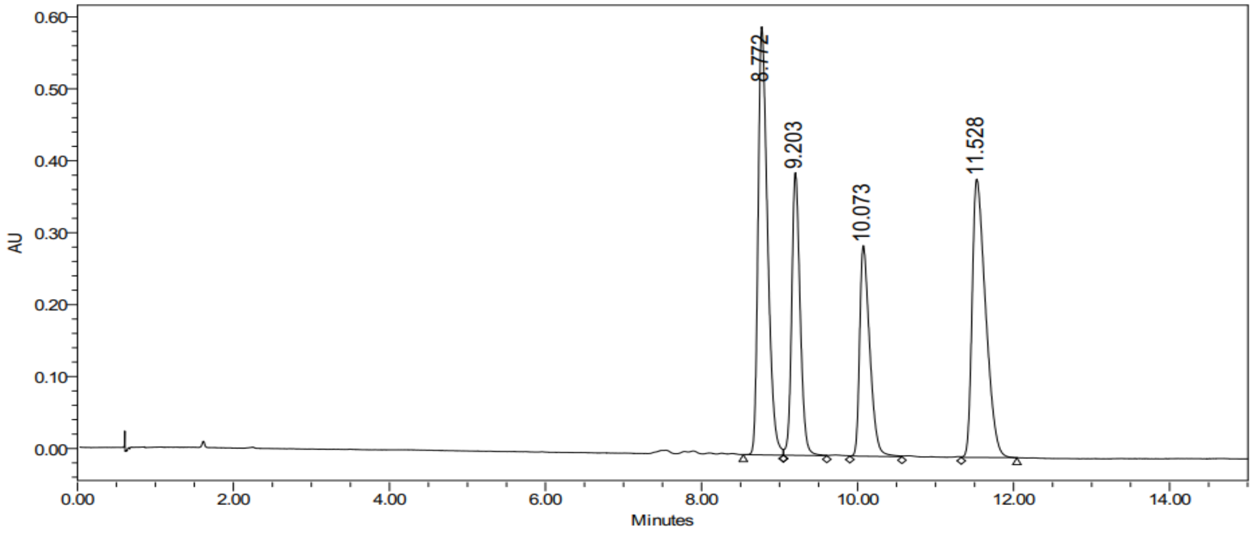

	Retention Time (min)	% Area
1	8.772	32.94
2	9.203	19.34
3	10.073	17.25
4	11.528	30.47

Conditions A – Enantioselective (**4n**)

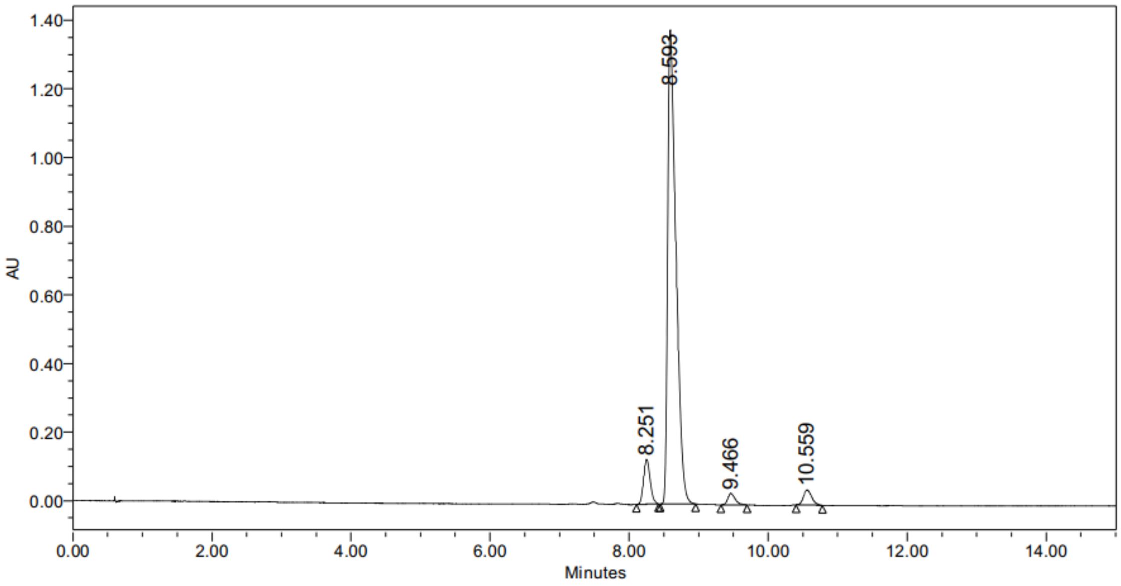

	Retention Time (min)	% Area
1	8.251	6.60
2	8.593	88.77
3	9.466	1.87
4	10.559	2.76

Conditions B – Enantioselective (**5n**)

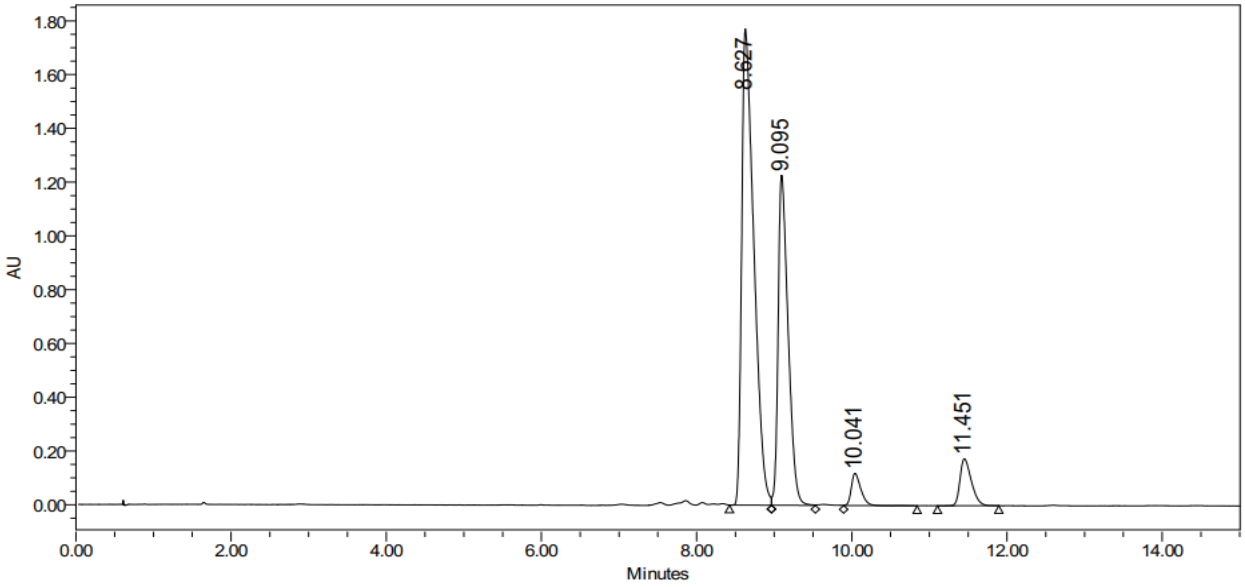

	Retention Time (min)	% Area
1	8.627	58.31
2	9.095	33.19
3	10.041	3.10
4	11.451	5.39

Racemate of **4o**/**5o**

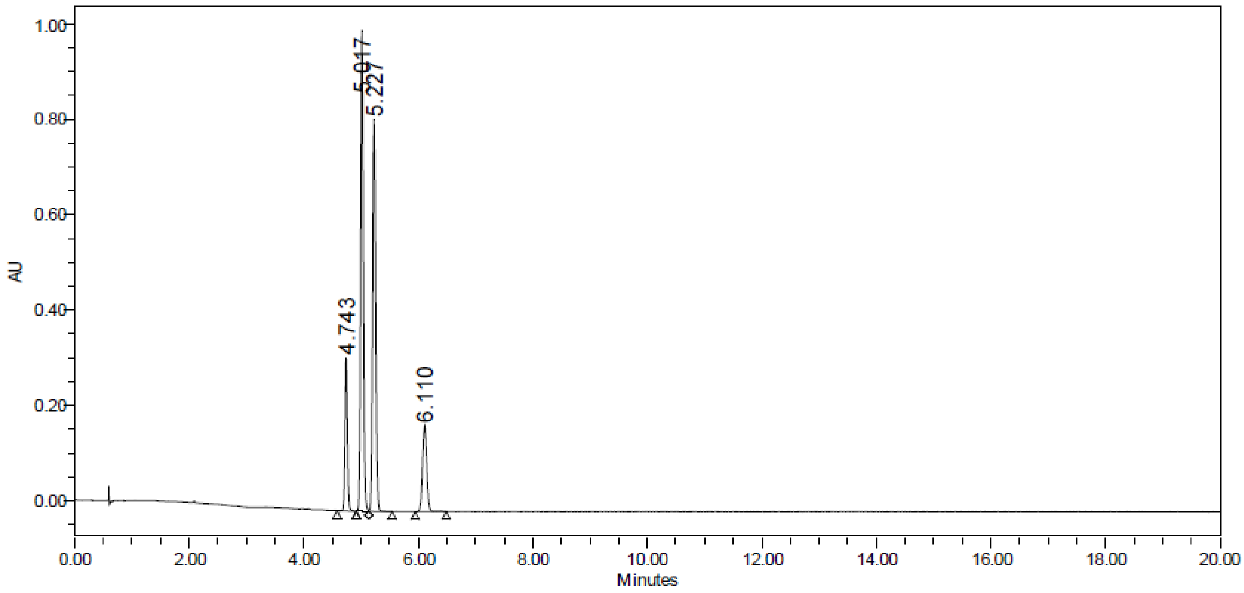

	Retention Time (min)	% Area
1	4.743	11.97
2	5.017	39.70
3	5.227	36.71
4	6.110	11.63

Conditions A – Enantioselective (**4o**)

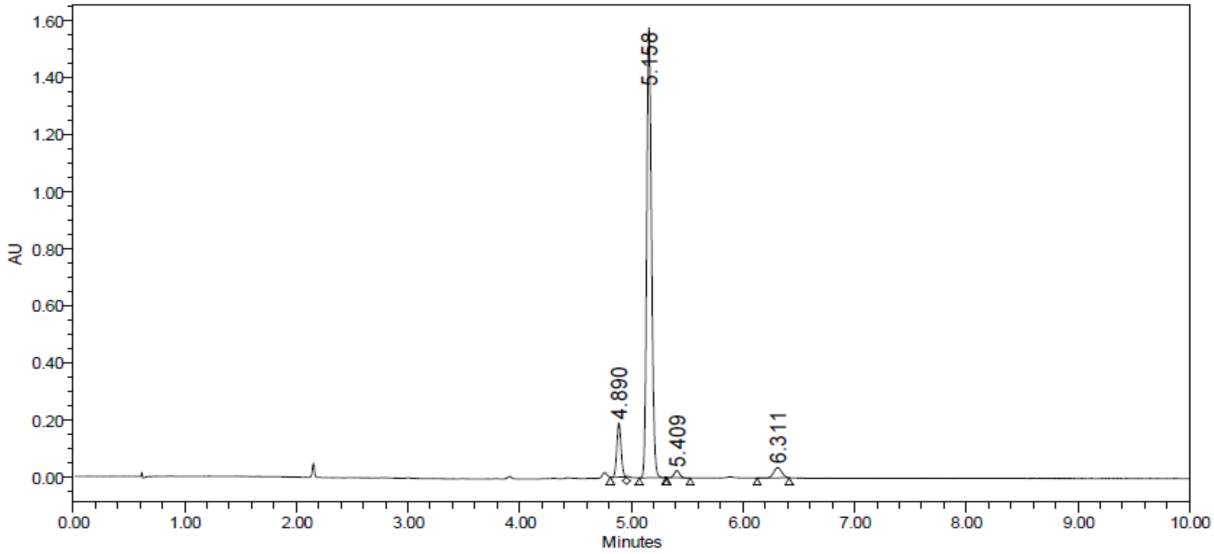

	Retention Time (min)	% Area
1	4.890	9.17
2	5.158	85.93
3	5.409	1.59
4	6.311	3.31

Conditions B – Enantioselective (**5o**)

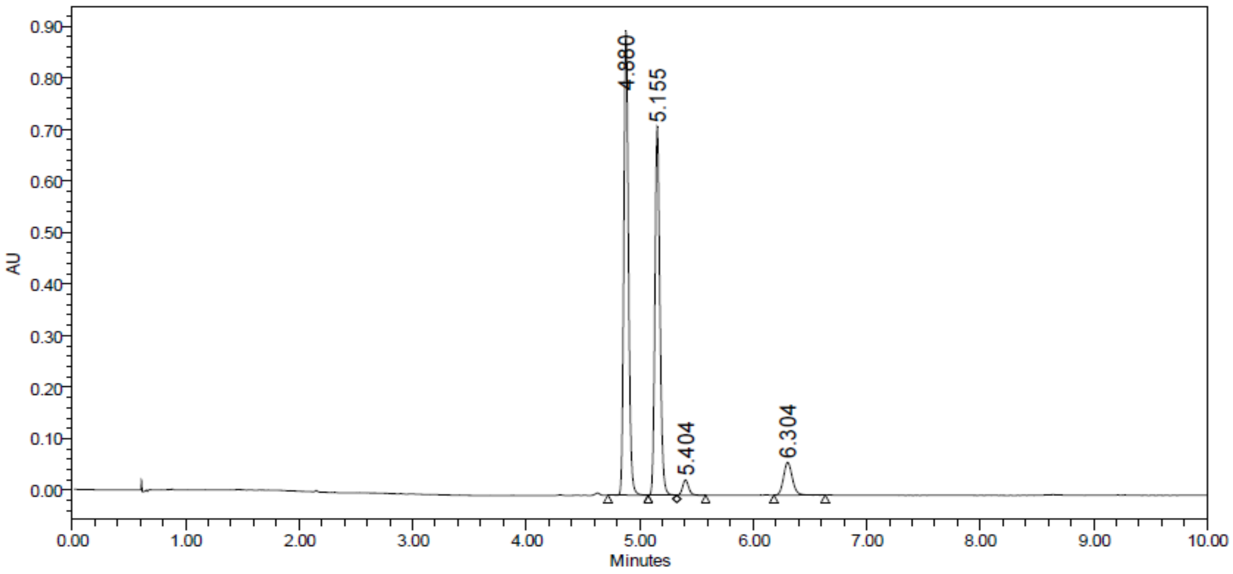

	Retention Time (min)	% Area
1	4.880	49.25
2	5.155	42.07
3	5.404	2.15
4	6.304	6.53

Racemate of **4p**/**5p**

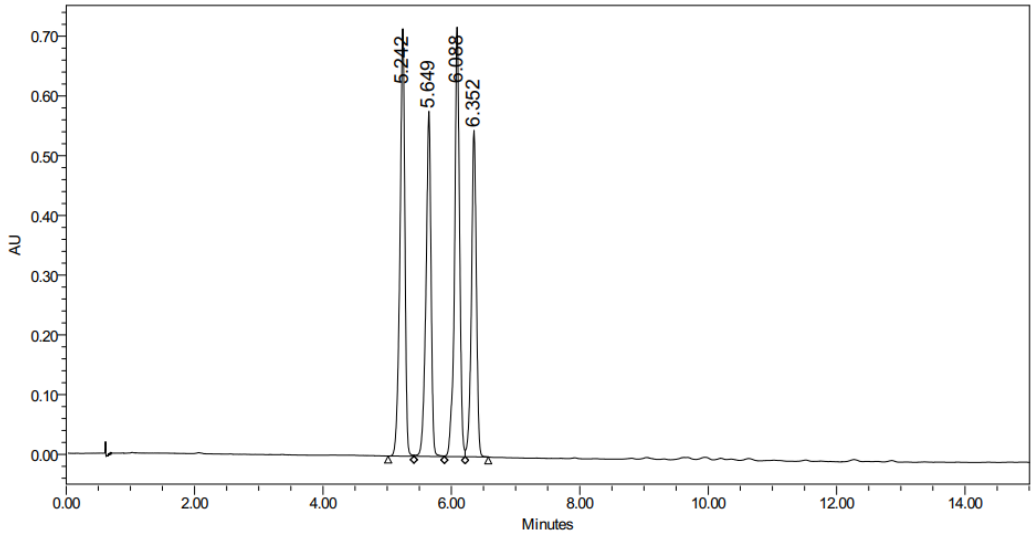

	Retention Time (min)	% Area
1	6.088	28.29
2	6.352	20.96
3	5.242	27.68
4	5.649	23.07

Conditions A – Enantioselective (**4p**)

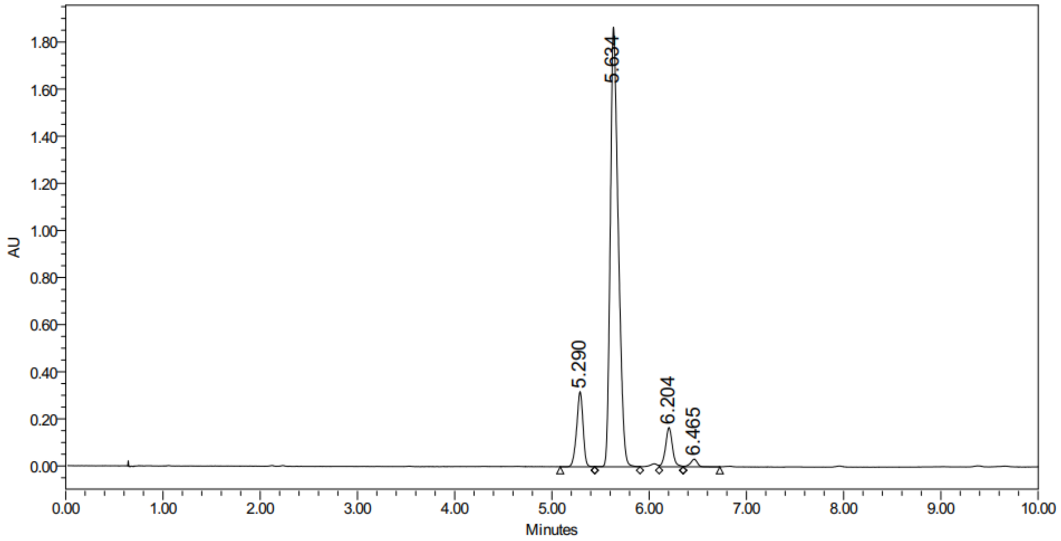

	Retention Time (min)	% Area
1	5.290	11.08
2	5.634	81.41
3	6.204	6.29
4	6.465	1.23

Conditions B – Enantioselective (**5p**)

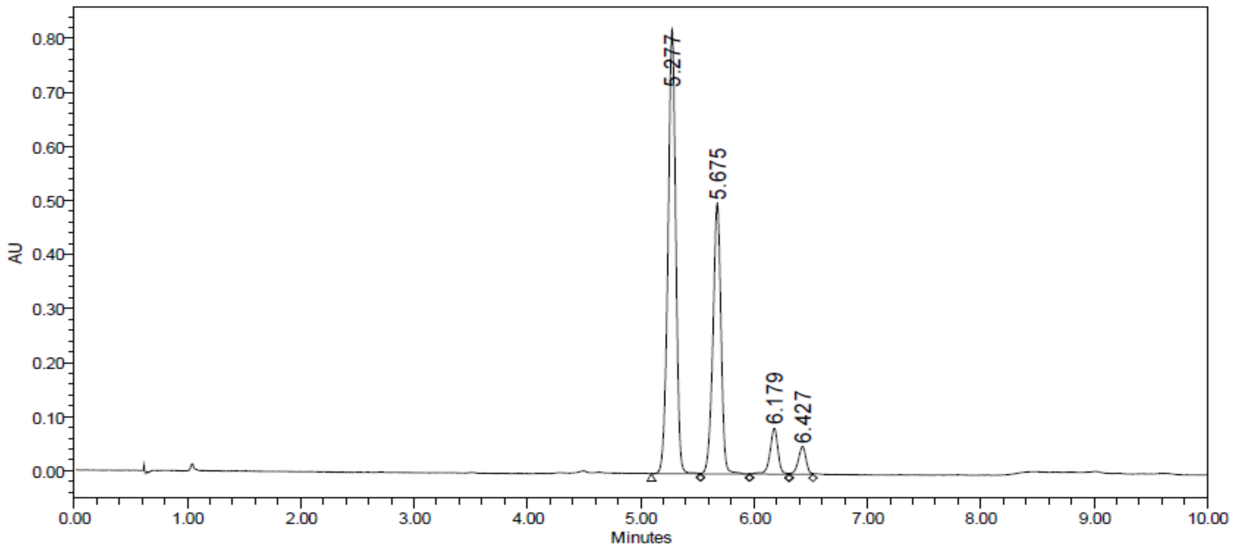

	Retention Time (min)	% Area
1	5.277	55.53
2	5.675	35.09
3	6.179	5.91
4	6.427	3.46

Racemate of **4q**/**5q**

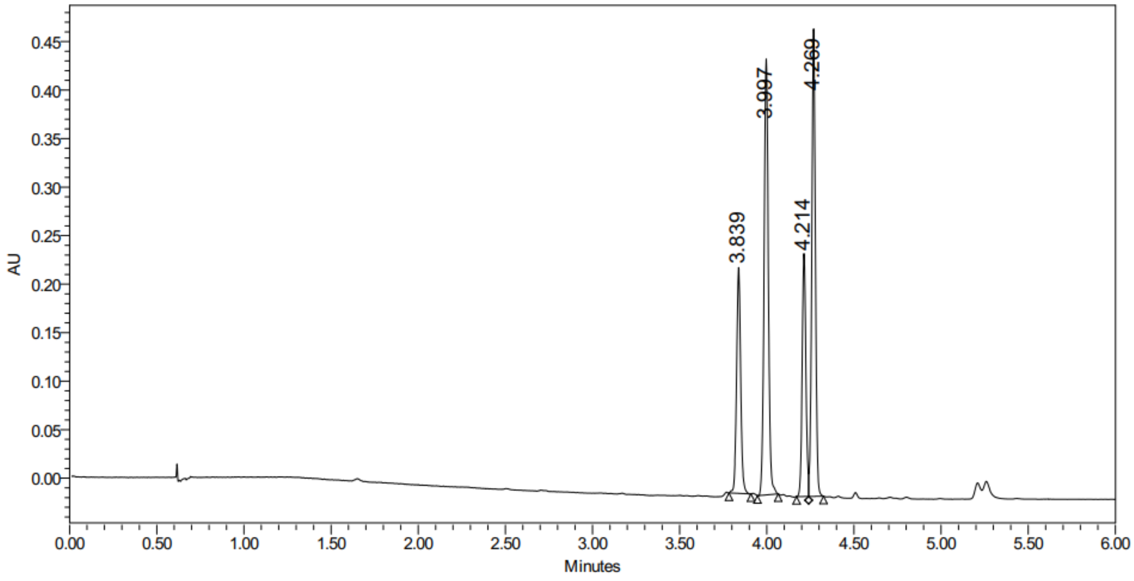

	Retention Time (min)	% Area
1	3.839	17.22
2	3.997	34.28
3	4.214	16.73
4	4.269	31.76

Conditions A – Enantioselective (**4q**)

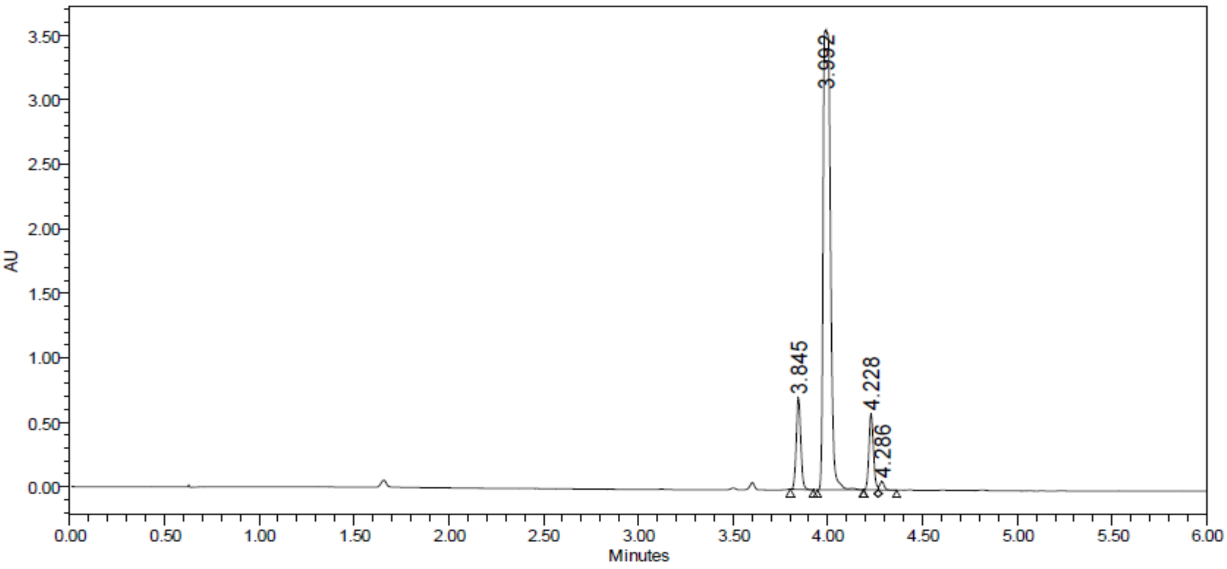

	Retention Time (min)	% Area
1	3.845	9.95
2	3.992	80.91
3	4.228	8.22
4	4.286	0.92

Conditions B – Enantioselective (**5q**)

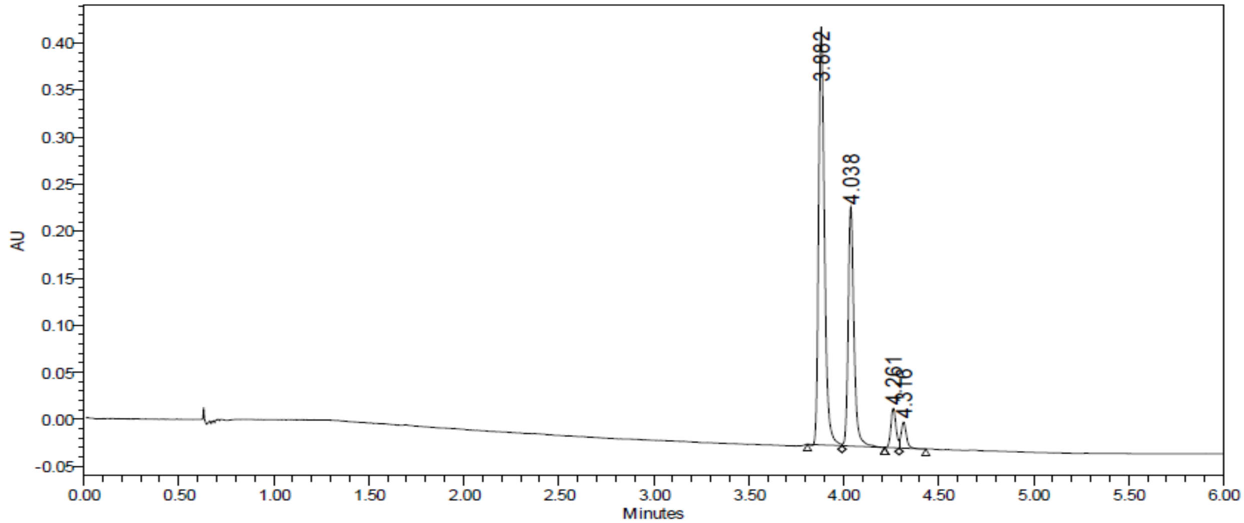

	Retention Time (min)	% Area
1	3.882	58.77
2	4.038	33.13
3	4.261	4.80
4	4.316	3.30

Racemate of **4r**/**5r**

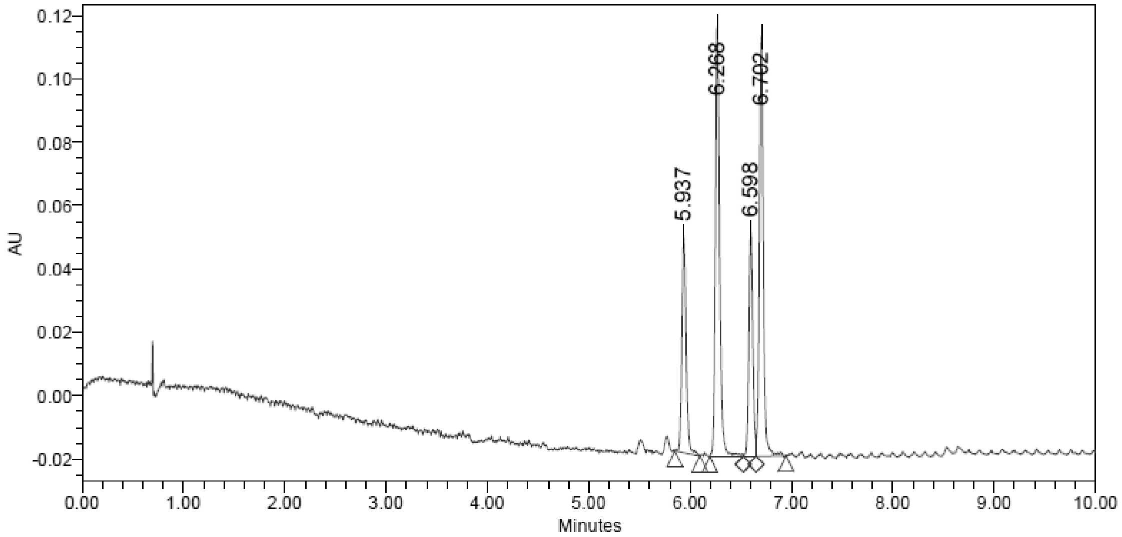

Peak Results

	RT	% Area
1	5.937	18.06
2	6.268	34.39
3	6.598	16.38
4	6.702	31.18

Conditions A – Enantioselective (**4r**)

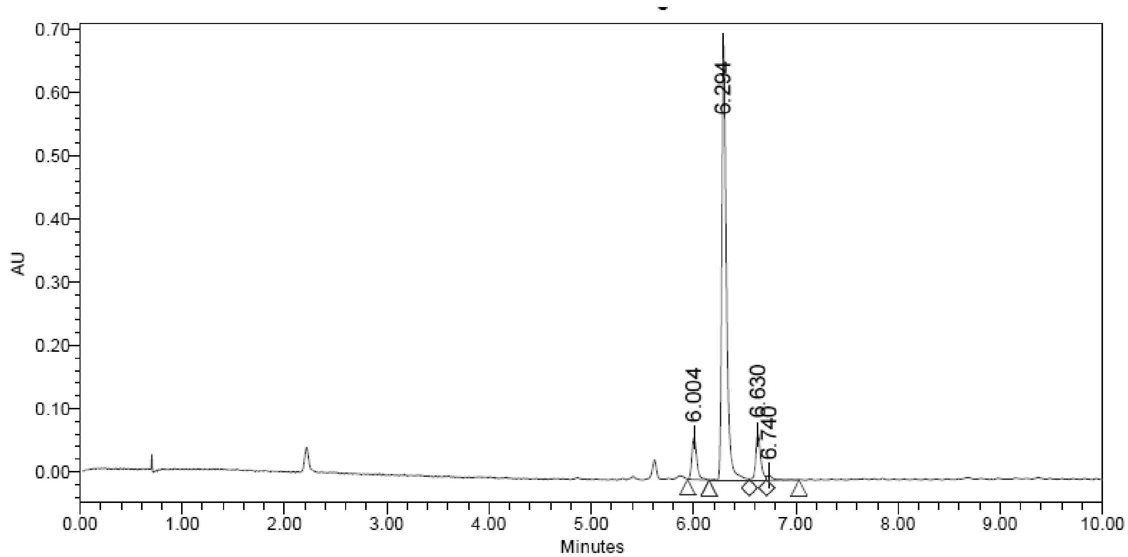

Peak Results

	RT	% Area
1	6.004	8.00
2	6.294	81.85
3	6.630	9.06
4	6.740	1.09

Conditions B – Enantioselective (**5r**)

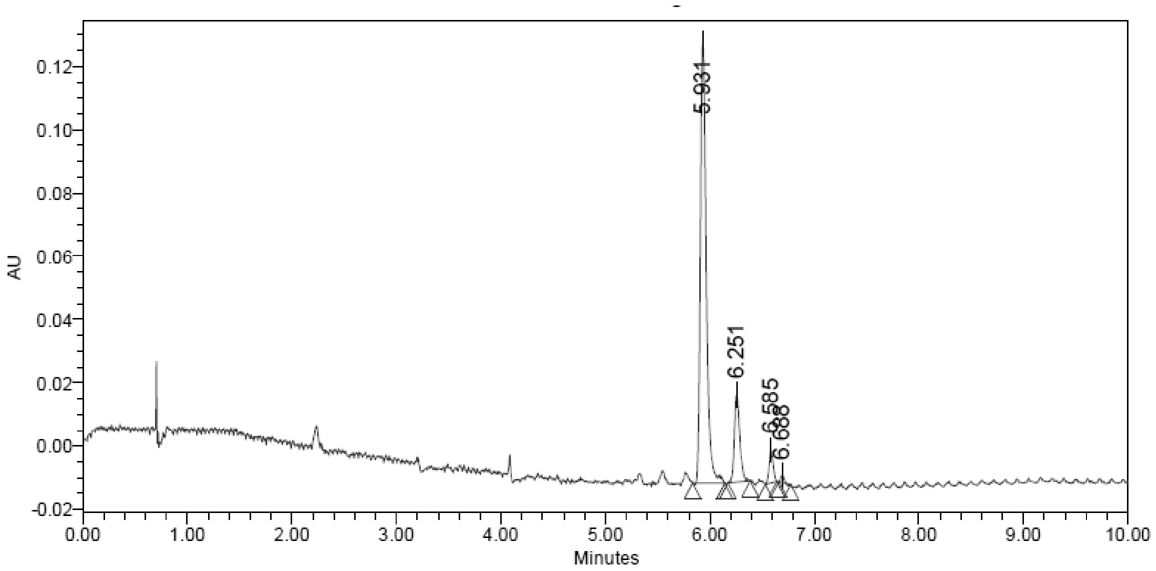

Peak Results

	RT	% Area
1	5.931	80.04
2	6.251	14.95
3	6.585	4.24
4	6.688	0.77

Racemate of **4s**/**5s**

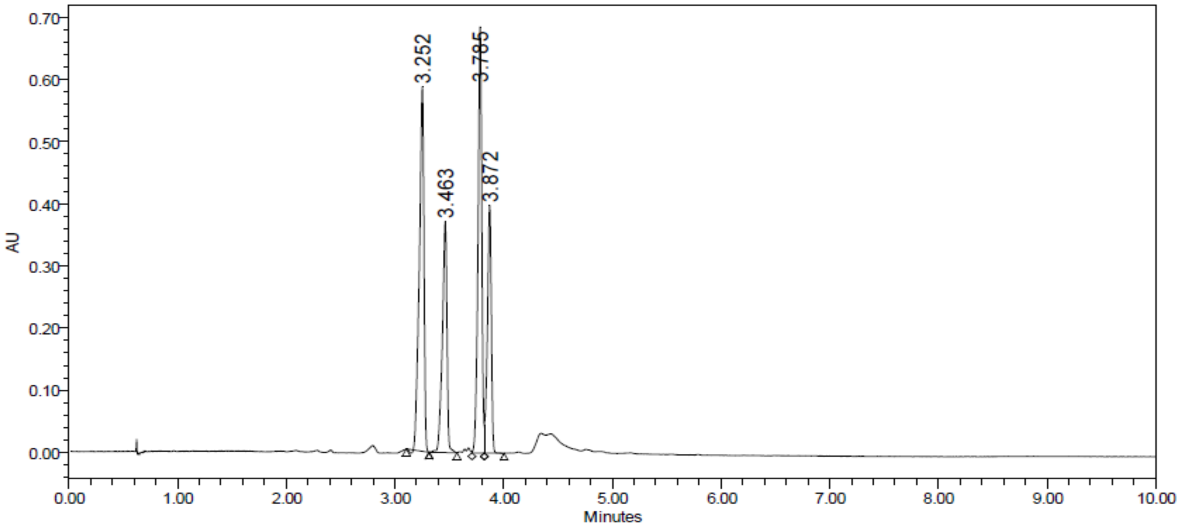

	Retention Time (min)	% Area
1	3.252	32.10
2	3.463	19.80
3	3.785	29.95
4	3.872	18.15

Conditions A – Enantioselective (**4s**)

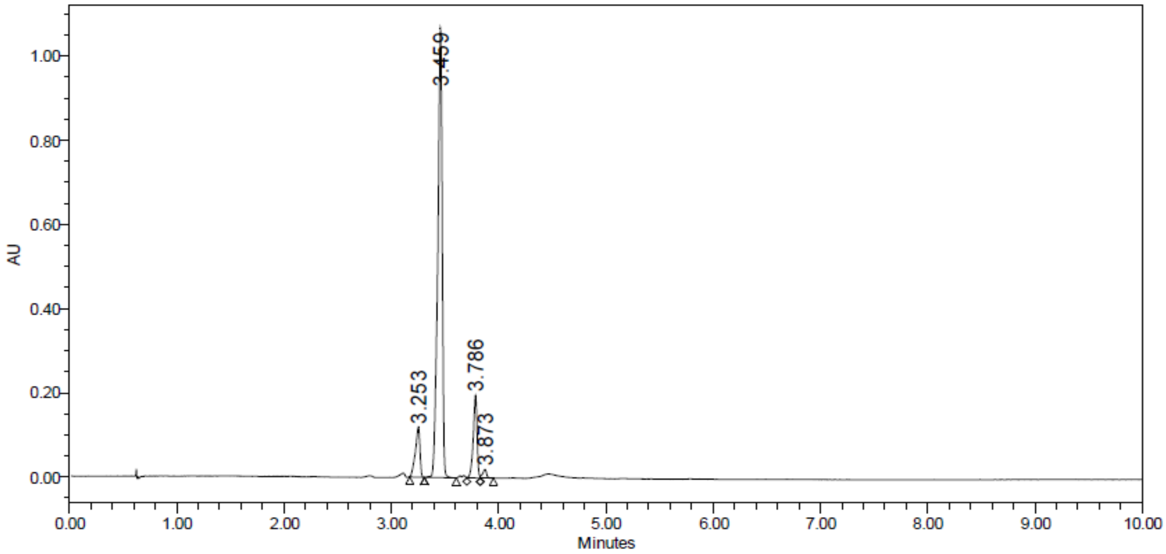

	Retention Time (min)	% Area
1	3.786	12.12
2	3.873	1.28
3	3.253	8.89
4	3.459	77.71

Conditions B – Enantioselective (**5s**)

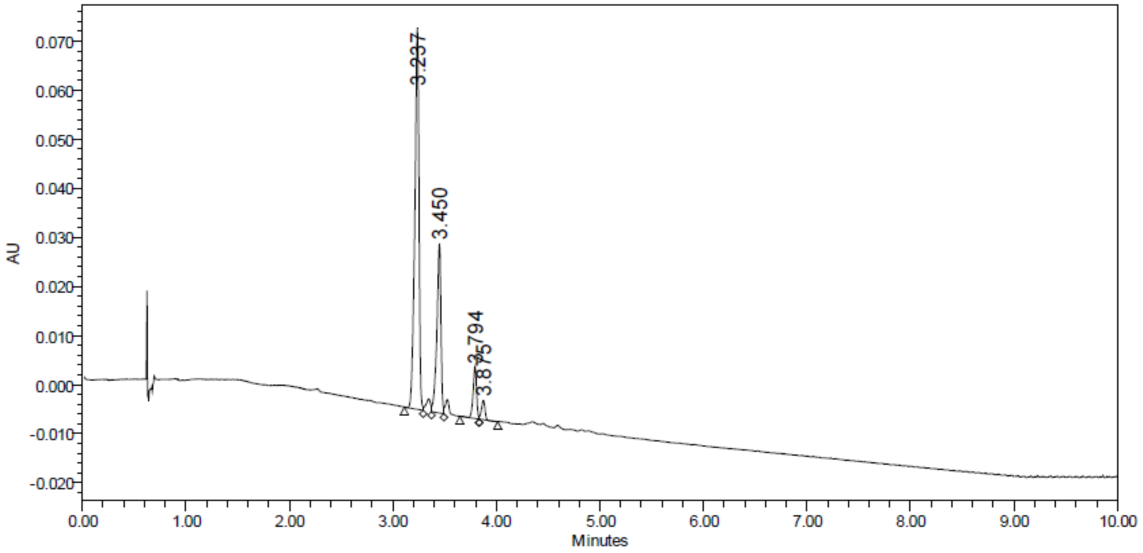

	Retention Time (min)	% Area
1	3.237	63.01
2	3.450	27.27
3	3.794	6.98
4	3.875	2.74

Racemate of **4t**/**5t**

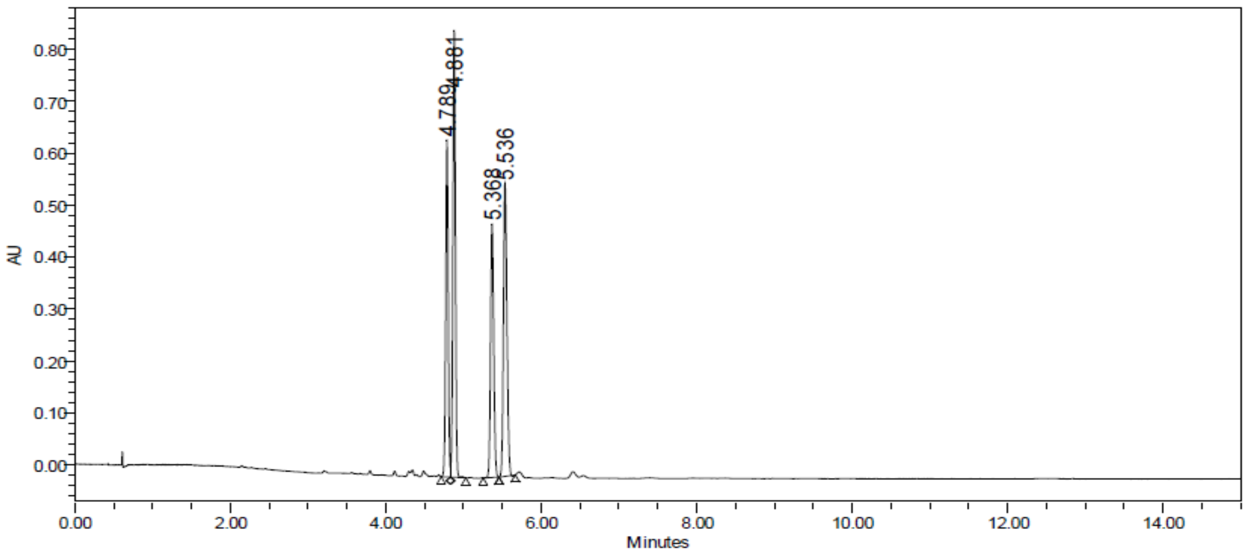

	Retention Time (min)	% Area
1	4.789	22.10
2	4.881	29.81
3	5.368	20.91
4	5.536	27.19

Conditions A – Enantioselective (**4t**)

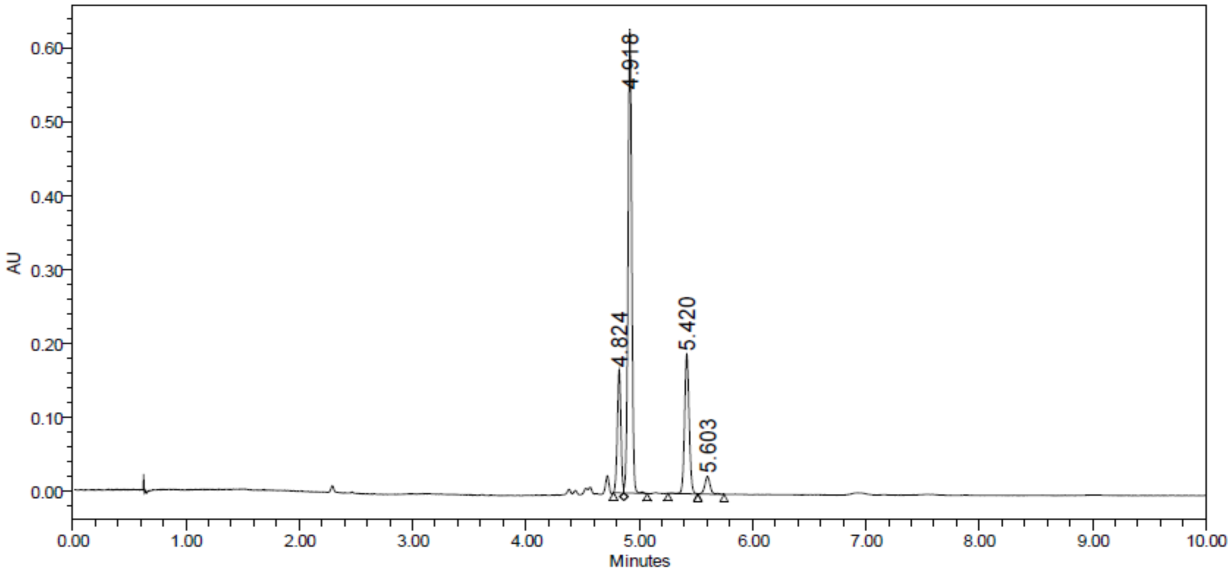

	Retention Time (min)	% Area
1	4.824	15.12
2	4.918	59.60
3	5.420	22.04
4	5.603	3.23

Conditions B – Enantioselective (**5t**)

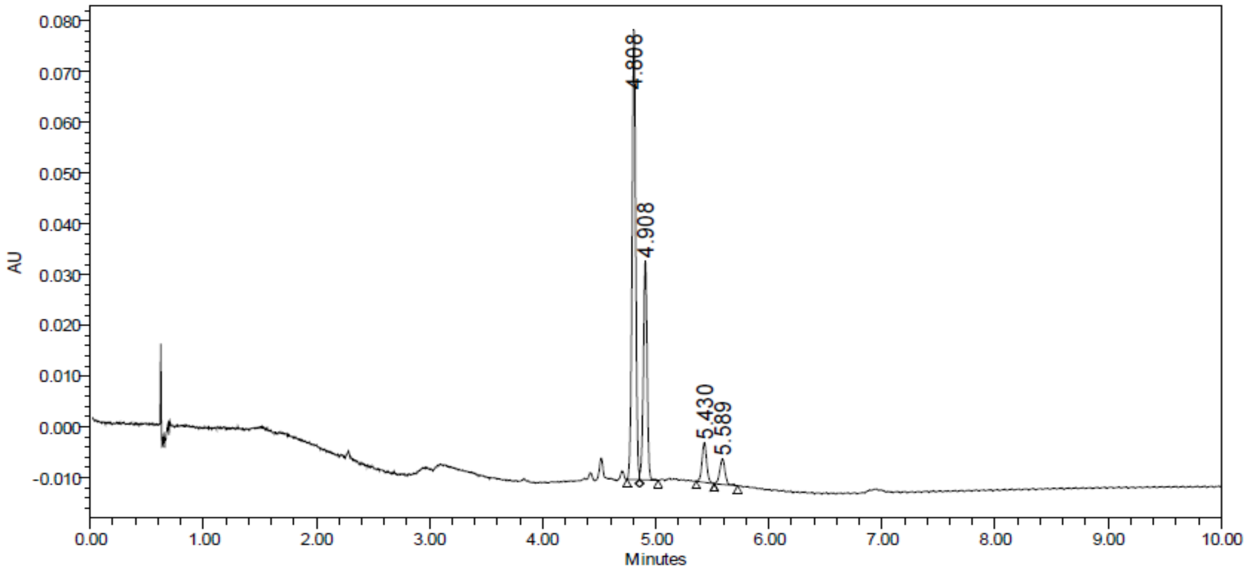

	Retention Time (min)	% Area
1	4.808	58.90
2	4.908	29.57
3	5.430	6.66
4	5.589	4.87

Racemate of **4u**/**5u**

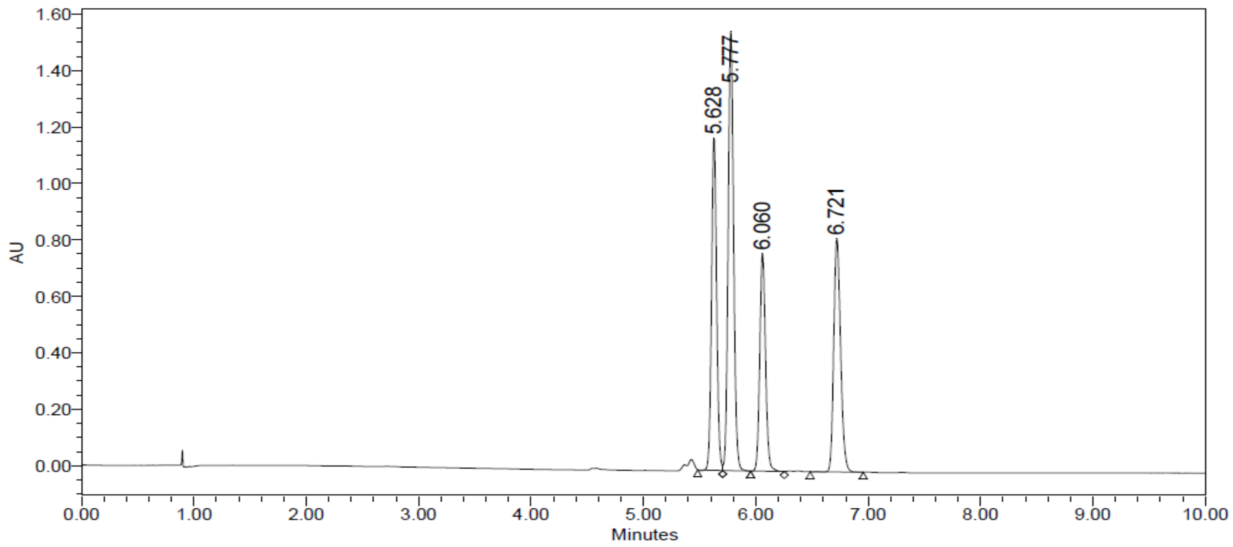

	Retention Time (min)	% Area
1	5.628	24.38
2	5.777	34.54
3	6.060	18.07
4	6.721	23.00

Conditions A – Enantioselective (**4u**)

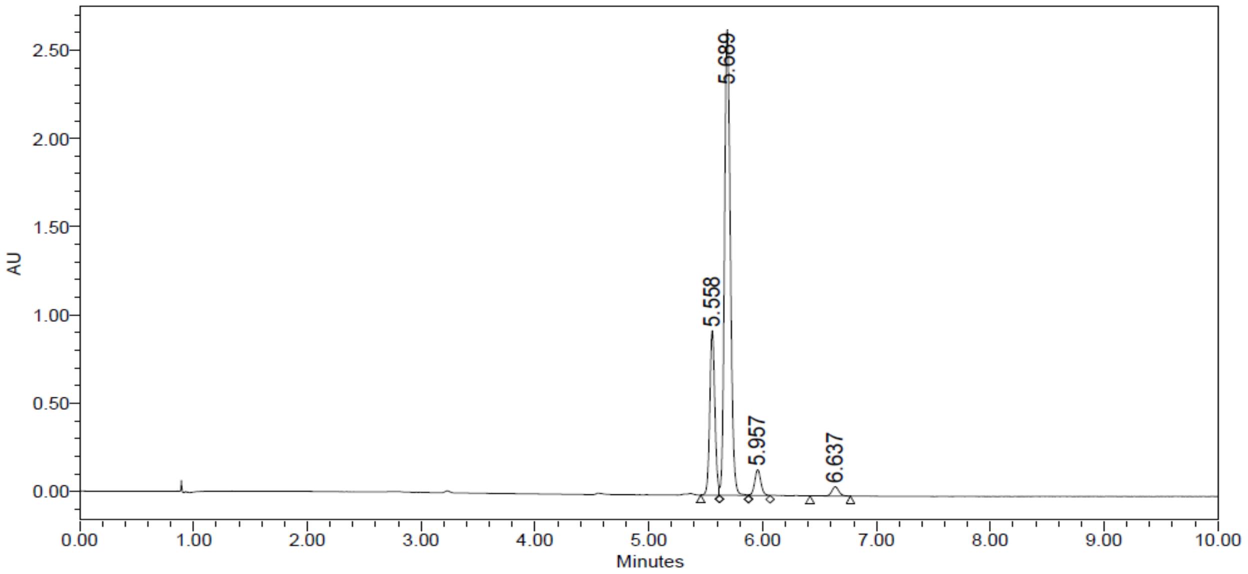

	Retention Time (min)	% Area
1	5.957	4.18
2	6.637	1.70
3	5.558	22.13
4	5.689	71.99

Conditions B – Enantioselective (**5u**)

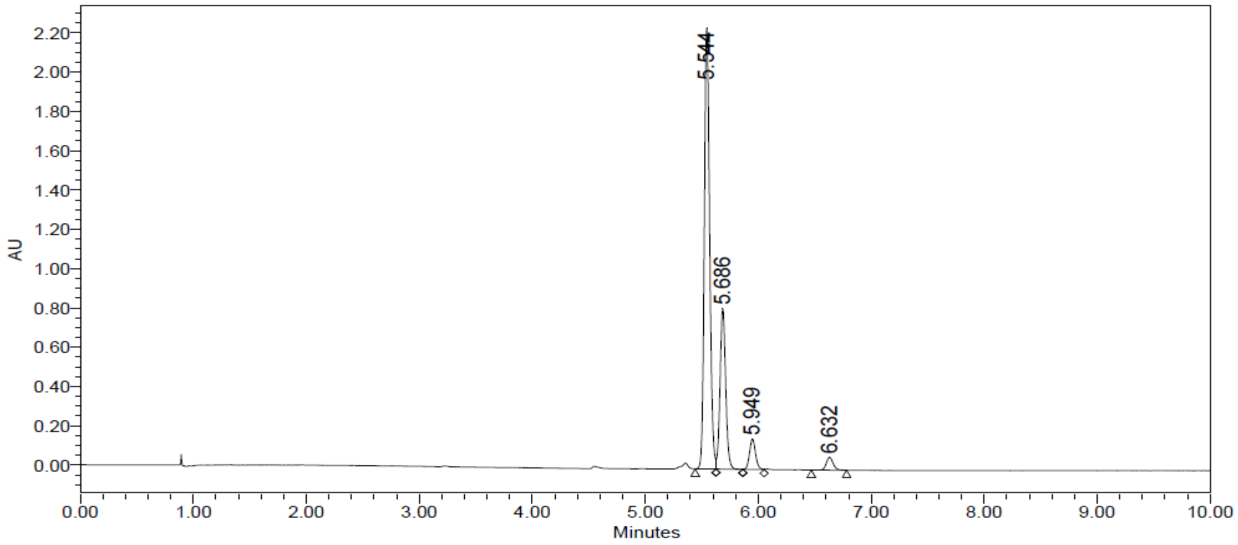

	Retention Time (min)	% Area
1	5.544	66.83
2	5.686	25.53
3	5.949	5.16
4	6.632	2.48

Racemate of **4v**/**5v**

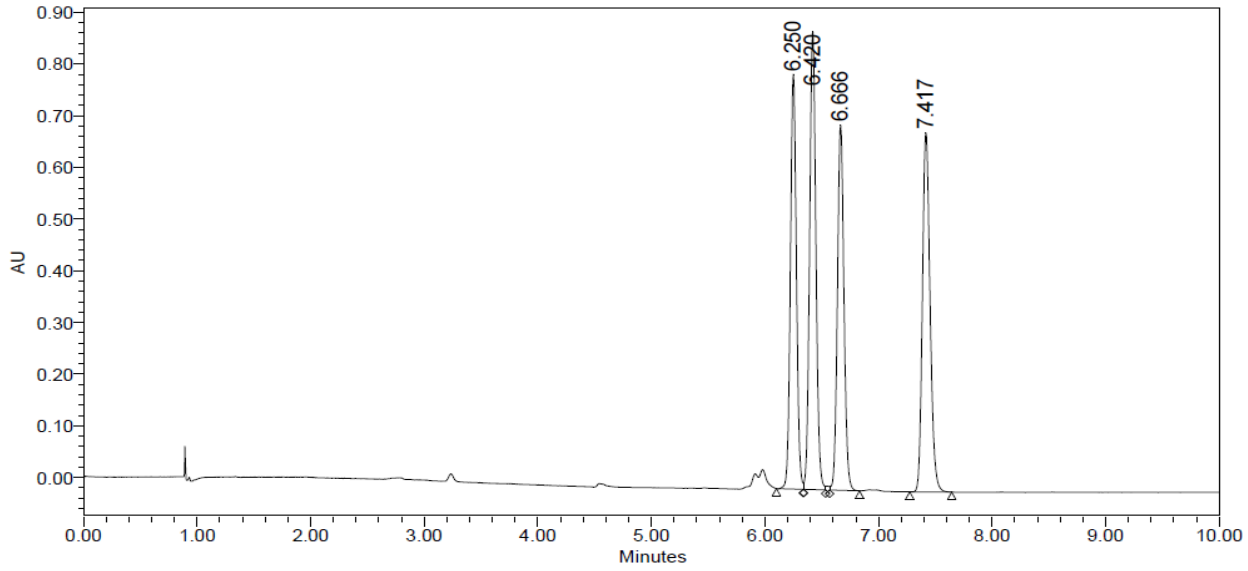

	Retention Time (min)	% Area
1	6.250	23.60
2	6.420	27.55
3	6.666	22.69
4	7.417	26.16

Conditions A – Enantioselective (**4v**)

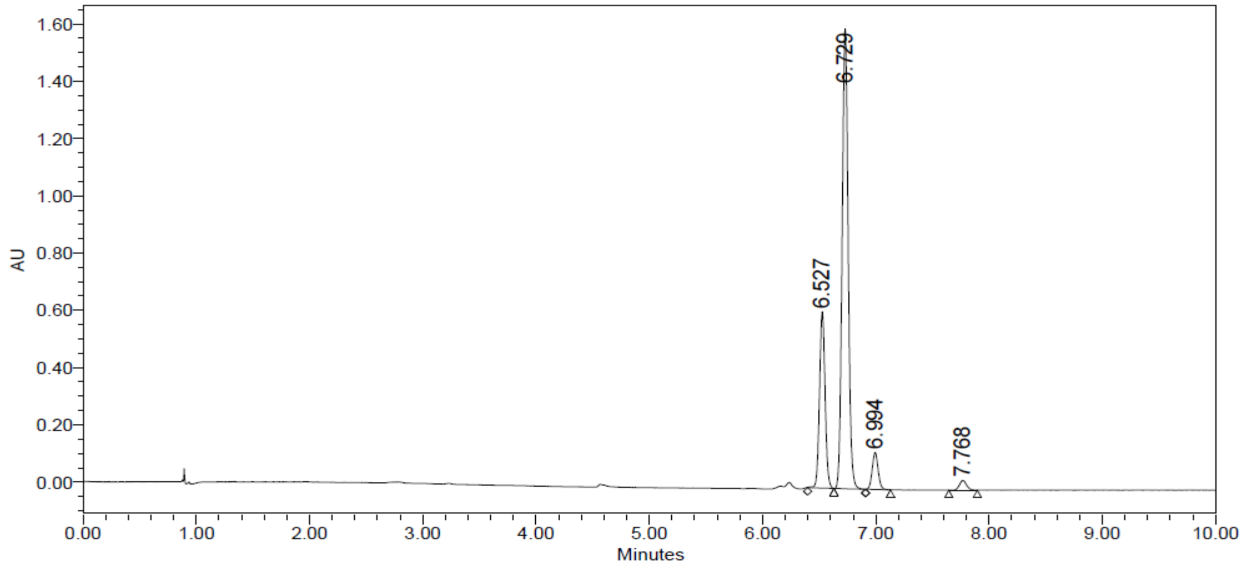

	Retention Time (min)	% Area
1	6.527	24.44
2	6.729	68.41
3	6.994	5.43
4	7.768	1.73

Conditions B – Enantioselective (**5v**)

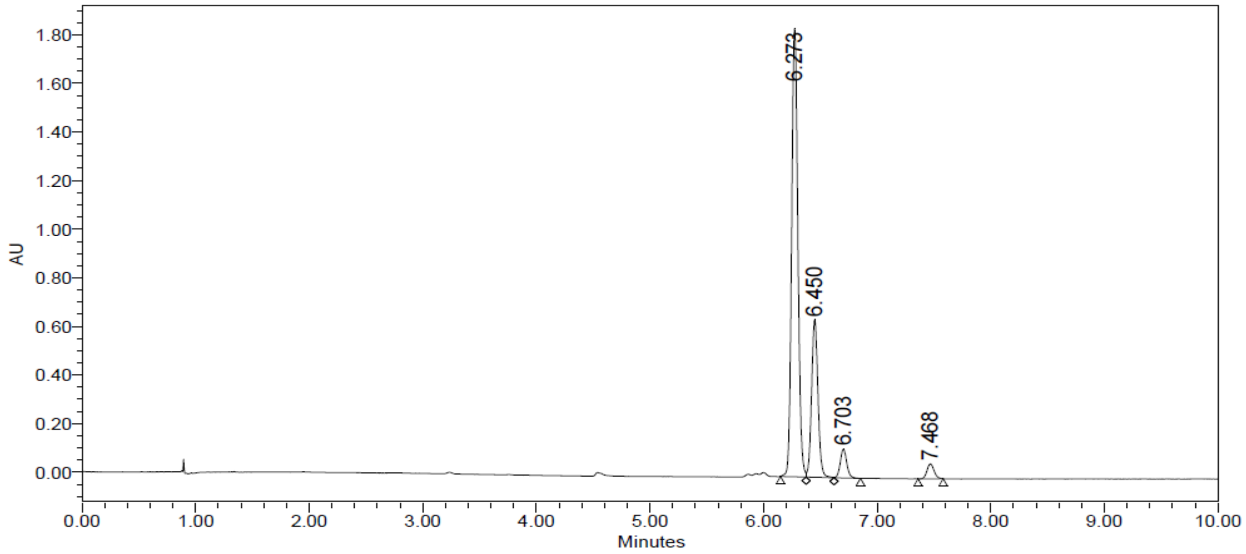

	Retention Time (min)	% Area
1	6.273	68.30
2	6.450	24.56
3	6.703	4.55
4	7.468	2.59

Part 4: Computational data

Density-functional theory (DFT) calculations were performed with Gaussian 16.[6] Geometry optimizations were performed with the B3LYP-D3 functional.[7,8] The LANL2DZ basis set with effective core potential (ECP)[9] was used for Pd, and the 6-31G(d) basis set was used for other atoms. Frequency calculations were performed at the same level of theory as for geometry optimization to characterize the stationary points as either minima (no imaginary frequencies) or first-order saddle points (one imaginary frequency) on the potential energy surface, as well as to obtain thermal Gibbs free energy corrections. Intrinsic reaction coordinate calculations were performed to ensure that the first-order saddle points found were true transition states (TS) connecting the reactants and the products. Single-point energies were calculated with the ωB97X-D functional,[10] with the SDD[11,12] basis set for Pd and the 6-311++G(d,p) basis set for other atoms. Solvation effects were incorporated with single-point energy calculations with the SMD[13] solvation model. Truhlar[14] correction for entropy and Head-Gordon[15] correction for enthalpy

---

[6] Frisch, M. J.; Trucks, G. W.; Schlegel, H. B.; Scuseria, G. E.; Robb, M. A.; Cheeseman, J. R.; Scalmani, G.; Barone, V.; Petersson, G. A.; Nakatsuji, H.; Li, X.; Caricato, M.; Marenich, A. V.; Bloino, J.; Janesko, B. G.; Gomperts, R.; Mennucci, B.; Hratchian, H. P.; Ortiz, J. V.; Izmaylov, A. F.; Sonnenberg, J. L.; Williams-Young, D.; Ding, F.; Lipparini, F.; Egidi, F.; Goings, J.; Peng, B.; Petrone, A.; Henderson, T.; Ranasinghe, D.; Zakrzewski, V. G.; Gao, J.; Rega, N.; Zheng, G.; Liang, W.; Hada, M.; Ehara, M.; Toyota, K.; Fukuda, R.; Hasegawa, J.; Ishida, M.; Nakajima, T.; Honda, Y.; Kitao, O.; Nakai, H.; Vreven, T.; Throssell, K.; Montgomery, J. A., Jr.; Peralta, J. E.; Ogliaro, F.; Bearpark, M. J.; Heyd, J. J.; Brothers, E. N.; Kudin, K. N.; Staroverov, V. N.; Keith, T. A.; Kobayashi, R.; Normand, J.; Raghavachari, K.; Rendell, A. P.; Burant, J. C.; Iyengar, S. S.; Tomasi, J.; Cossi, M.; Millam, J. M.; Klene, M.; Adamo, C.; Cammi, R.; Ochterski, J. W.; Martin, R. L.; Morokuma, K.; Farkas, O.; Foresman, J. B.; Fox, D. J. Gaussian, Inc., Wallingford CT, 2016.

[7] A. D. Becke, *J. Chem. Phys.* **1993**, *98*, 5648-5652.

[8] C. Lee, W. Yang, R. G. Parr, *Phys. Rev. B* **1998**, *37*, 785-789.

[9] P. J. Hay, W. R. Wadt, *J. Chem. Phys.* **1985**, *82*, 299-310.

[10] J. D. Chai, M. Head-Gordon, *Phys. Chem. Chem. Phys.* **2008**, *10*, 6615-6620.

[11] U. Haüssermann, M. Dolg, H. Stoll, H. Preuss, P. Schwerdtfeger, R. M. Pitzer, *Mol. Phys.* **1993**, *78*, 1211-1224.

[12] W. Küchle, M. Dolg, H. Stoll, H. Preuss, *J. Chem. Phys.* **1994**, *100*, 7535-7542.

[13] A. V. Marenich, C. J. Cramer, D. G. Truhlar, *J. Phys. Chem. B* **2009**, *113*, 6378–6396.

14 R. F. Ribeiro, A. V. Marenich, C. J. Cramer, D. G. Truhlar, *J. Phys. Chem. B* **2011**, *115*, 14556-14562.

[15] Y.-P. Li, J. Gomes, S. Mallikarjun Sharada, A. T. Bell, *J. Phys. Chem. C* **2015**, *119*, 1840-1850.

(frequency cut-off is 100 cm^{-1}) are performed using GoodVibes v3.0.1.[16] Unless otherwise noted, the relative energies reported in the text are Gibbs free energies with the solvent effect corrections. Molecular structure visualizations were obtained using CYLview.[17]

[16] G. Luchini, J. Alegre-Requena, J. Ifunes, J. Rodríguez-Guerra, J. Chen, R. Paton, GoodVibes v3.0.0, http://doi.org/10.5281/zenodo.595246 (2019).

[17] C. Y. Legault, CYLview, 1.0b, Université de Sherbrooke, http://www.cylview.org (2009).

Structure	E_SPC	E	ZPE	H_SPC	T.S	T.qh-S	G(T)_SPC	qh-G(T)_SPC
**1a**	-500.381006	-500.428608	0.178499	-500.191469	0.046539	0.046375	-500.238009	-500.237845
**3d**	-813.203109	-813.267472	0.313660	-812.872801	0.060652	0.058067	-812.933453	-812.930868
**H_2O**	-76.436653	-76.408962	0.021160	-76.411714	0.021446	0.021446	-76.433161	-76.433161
***E*,*E*-diphenylallyl**	-579.425206	-579.453042	0.228565	-579.183261	0.053612	0.051267	-579.236873	-579.234528
***Z*,*E*-diphenylallyl**	-579.422384	-579.448273	0.229143	-579.180031	0.053100	0.050971	-579.233131	-579.231002
***Z*,*Z*-diphenylallyl**	-579.417781	-579.441286	0.229340	-579.175512	0.051624	0.050321	-579.227136	-579.225833
**Ia**	-1237.135390	-1237.260027	0.468155	-1236.641262	0.082133	0.076277	-1236.723395	-1236.717540
**Ib**	-1237.130219	-1237.254109	0.468375	-1236.636176	0.081307	0.075237	-1236.717483	-1236.711414
**Ic**	-1237.130194	-1237.254472	0.468192	-1236.636020	0.082824	0.075975	-1236.718844	-1236.711995
**Id**	-1237.125280	-1237.251442	0.468225	-1236.631372	0.080454	0.075235	-1236.711826	-1236.706607
**IIa**	-2779.756148	-2778.767705	0.788675	-2778.918557	0.133214	0.121379	-2779.051772	-2779.039936
**IIb**	-2779.756189	-2778.770288	0.790101	-2778.917405	0.132125	0.120674	-2779.04953	-2779.038079
**IIc**	-2779.737593	-2778.749897	0.789759	-2778.899149	0.132869	0.121015	-2779.032018	-2779.020164
**TS-*endo***	-4016.905537	-4016.047623	1.259476	-4015.571231	0.191940	0.166691	-4015.763171	-4015.737922
**TS-*exo***	-4016.909539	-4016.053658	1.261043	-4015.574344	0.186695	0.166010	-4015.761039	-4015.740355
ent*1-TS-*exo	-4016.903850	-4016.043918	1.259976	-4015.569253	0.192240	0.166129	-4015.761493	-4015.735382
ent2*-TS-*exo	-4016.905148	-4016.046469	1.259459	-4015.570809	0.192756	0.167129	-4015.763566	-4015.737938

1a

H	-0.967437	-2.006998	-0.876078
C	-1.526922	-0.103583	0.000026
C	-0.698372	0.983227	-0.000015
C	-0.730467	-1.388896	0.000069
H	-0.967401	-2.006900	0.876298
C	0.693188	-0.896823	0.000026
C	1.894145	-1.594433	0.000020
C	3.093724	-0.871860	-0.000023
C	3.089817	0.529044	-0.000057
C	1.886261	1.235957	-0.000052
C	0.689144	0.514203	-0.000014
H	1.906918	-2.681450	0.000048
H	4.040895	-1.404567	-0.000031
H	4.032970	1.068579	-0.000088
H	1.882031	2.322745	-0.000077
C	-1.051271	2.438531	-0.000051
H	-2.128702	2.615333	-0.000109
H	-0.622865	2.933988	0.880651
H	-0.622772	2.933967	-0.880718
C	-2.981826	-0.110275	0.000019
O	-3.652525	-1.133646	0.000056
H	-3.477966	0.883912	-0.000025

3d

C	-1.077810	3.270803	0.184671
C	0.038351	1.483346	-0.865920
C	1.147492	2.520717	-0.546486
C	0.442422	3.581414	0.334626
H	-1.675563	4.149188	-0.078241
H	-1.473640	2.867948	1.120531
H	0.188244	1.030342	-1.850830
H	2.024264	2.079712	-0.066036
H	1.484312	2.966188	-1.489313
H	0.751808	3.492869	1.379489
H	0.685314	4.596383	0.002699
N	-1.222096	2.240649	-0.865012
H	-1.303377	2.703742	-1.768330
C	0.012421	0.322780	0.162706
F	0.044605	0.928222	1.446454
C	1.271754	-0.535777	0.036932
C	1.446422	-1.368331	-1.076058
C	2.272738	-0.488859	1.012335
C	2.604397	-2.131248	-1.216329
H	0.663554	-1.436078	-1.826379
C	3.430318	-1.258086	0.874152
H	2.140603	0.148461	1.878736
C	3.602541	-2.078807	-0.240290
H	2.723370	-2.773368	-2.085072
H	4.198178	-1.213210	1.642162
H	4.503741	-2.676552	-0.346966
C	-1.237270	-0.548489	0.118758
C	-1.638685	-1.198843	1.291811
C	-1.951852	-0.780731	-1.061335
C	-2.732354	-2.063202	1.286354
H	-1.090327	-1.017975	2.209992
C	-3.041321	-1.652966	-1.069374
H	-1.677081	-0.268776	-1.976714
C	-3.436504	-2.296957	0.103629
H	-3.032719	-2.556388	2.207187
H	-3.587851	-1.819343	-1.994007
H	-4.287963	-2.972268	0.096983

***E*,*E*-diphenylallyl**

C	-1.247042	1.024645	-0.000003
H	-1.257273	2.116903	-0.000005
C	-0.000000	0.403262	-0.000001
H	-0.000000	-0.688717	0.000001
C	1.247042	1.024645	-0.000001
H	1.257273	2.116903	-0.000003
C	2.530023	0.391977	0.000002
C	3.720815	1.180387	0.000001
C	2.740646	-1.021444	0.000005
C	4.989599	0.616478	0.000004
H	3.618355	2.265238	-0.000001
C	4.013258	-1.576529	0.000007
H	1.879801	-1.685323	0.000006
C	5.164052	-0.774552	0.000007
H	5.861413	1.271489	0.000003
H	4.113832	-2.662305	0.000009
H	6.157502	-1.217199	0.000008
C	-2.530023	0.391977	-0.000003
C	-3.720815	1.180387	-0.000006
C	-2.740646	-1.021444	-0.000001

C	-4.989599	0.616478	-0.000006
H	-3.618355	2.265238	-0.000007
C	-4.013258	-1.576529	-0.000001
H	-1.879801	-1.685323	0.000001
C	-5.164052	-0.774552	-0.000004
H	-5.861413	1.271489	-0.000008
H	-4.113832	-2.662305	0.000000
H	-6.157502	-1.217199	-0.000004

***Z*,*E*-diphenylallyl**

C	0.810052	-0.053892	0.124321
H	0.297944	0.883789	0.323085
C	0.059298	-1.228873	0.052731
H	0.650638	-2.148546	0.010517
C	-1.316967	-1.476965	0.072010
H	-1.596701	-2.528969	0.148653
C	-2.433640	-0.579562	0.015223
C	-3.751023	-1.079415	0.247313
C	-2.359088	0.810328	-0.300843
C	-4.877335	-0.270234	0.198765
H	-3.865121	-2.138351	0.478655
C	-3.493995	1.613019	-0.352015
H	-1.397860	1.239855	-0.555267
C	-4.770155	1.097280	-0.094912
H	-5.856851	-0.708054	0.393279
H	-3.380191	2.667313	-0.605525
H	-5.650877	1.735059	-0.126438
C	2.238729	0.036274	0.057494
C	2.890924	1.279285	0.318971
C	3.111168	-1.045926	-0.271445
C	4.271120	1.422436	0.275427
H	2.272094	2.140742	0.568086
C	4.491736	-0.895898	-0.305826
H	2.683426	-2.014574	-0.517057
C	5.102509	0.336220	-0.033118
H	4.710655	2.397439	0.487955
H	5.108956	-1.757790	-0.561768
H	6.183889	0.447627	-0.068749

28

***Z*,*Z*-diphenylallyl**

C	1.258607	1.993531	-0.327354
H	1.955798	2.723085	-0.745906
C	-0.000134	2.516289	0.000226
H	0.000111	3.614488	0.000480
C	-1.259027	1.993873	0.327704
H	-1.955895	2.723291	0.747028
C	-1.854625	0.715615	0.067750
C	-3.134996	0.403668	0.615248
C	-1.290761	-0.280102	-0.783589
C	-3.782525	-0.796155	0.356791
H	-3.612180	1.140208	1.262015
C	-1.950106	-1.478257	-1.040688
H	-0.342778	-0.076793	-1.265606
C	-3.196864	-1.764573	-0.473413
H	-4.756883	-0.985522	0.808414
H	-1.476265	-2.205562	-1.699630
H	-3.698779	-2.709769	-0.670129
C	1.854166	0.715148	-0.067842
C	3.135492	0.404429	-0.613846
C	1.289661	-0.281862	0.781638
C	3.783597	-0.795059	-0.355322
H	3.613103	1.141815	-1.259336
C	1.949753	-1.479565	1.039070
H	0.340568	-0.079956	1.261989
C	3.197586	-1.764463	0.473497
H	4.758739	-0.983339	-0.805708
H	1.475484	-2.207705	1.696784
H	3.700035	-2.709341	0.670390

H_2O

O	-0.000000	0.000000	0.119736
H	0.000000	0.761619	-0.478942
H	-0.000000	-0.761619	-0.478942

Ia

C	2.167966	-0.501517	-0.494620
C	3.076700	0.519171	-1.000917
C	4.194362	-0.881356	0.478538
C	5.337824	-1.304906	1.224837
C	6.521328	-0.631419	1.083591
C	6.640355	0.498460	0.198830
C	5.572125	0.944633	-0.532071
C	4.311269	0.280214	-0.415708
H	5.259492	-2.159469	1.893884
H	7.397277	-0.948683	1.644648
H	7.602488	0.998440	0.117099

H	5.678840	1.798570	-1.197648
C	2.727164	1.605401	-1.974543
H	1.865375	2.199818	-1.639305
H	3.564950	2.299250	-2.098132
H	2.481025	1.216695	-2.973571
C	0.831307	-0.498253	-0.840030
H	0.462273	0.347592	-1.414429
C	2.897474	-1.358010	0.420070
H	2.488972	-2.159144	1.020499
C	-1.554482	-1.132480	-0.781681
C	0.084179	-2.759584	-0.064942
C	-2.215971	-2.535863	-0.763741
H	-1.669559	-0.623436	-1.744619
C	-1.043847	-3.533710	-0.755549
H	-0.006313	-2.791437	1.028491
H	1.083473	-3.095259	-0.347091
H	-2.811852	-2.642451	0.145805
H	-2.888068	-2.674078	-1.613786
H	-1.289049	-4.467844	-0.241976
H	-0.738343	-3.778971	-1.778944
N	-0.128281	-1.398142	-0.557334
C	-2.143427	-0.209020	0.331495
F	-1.889307	-0.902262	1.537041
C	-3.653985	-0.041789	0.214880
C	-6.434595	0.344782	0.090882
C	-4.410857	0.065765	1.388462
C	-4.306857	0.043734	-1.021499
C	-5.688372	0.237877	-1.082472
C	-5.790129	0.256459	1.326738
H	-3.911022	-0.006848	2.347844
H	-3.748565	-0.044047	-1.948621
H	-6.178723	0.300499	-2.050093
H	-6.362493	0.336021	2.246963
H	-7.509720	0.493771	0.043294
C	-1.412812	1.127081	0.405383
C	0.102060	3.488657	0.394551
C	-1.682310	2.137806	-0.526156
C	-0.387801	1.315355	1.338861
C	0.365318	2.489668	1.331915
C	-0.927610	3.310713	-0.532987
H	-2.486792	2.014833	-1.244985
H	-0.161177	0.530024	2.049174
H	1.171675	2.611647	2.048974
H	-1.145985	4.085961	-1.262395
H	0.695282	4.398699	0.384169

Ib

C	2.198565	-0.302765	-0.602657
C	2.950192	-0.797701	0.543073
C	4.367875	0.334296	-0.929856
C	5.639801	0.825731	-1.357943
C	6.737337	0.633686	-0.562223
C	6.634978	-0.056132	0.696778
C	5.439722	-0.556655	1.141947
C	4.267819	-0.399189	0.339211
H	5.723865	1.352459	-2.306285
H	7.709340	1.007091	-0.875642
H	7.533203	-0.182319	1.296698
H	5.382926	-1.086347	2.090518
C	2.406927	-1.373236	1.819900
H	1.393962	-1.005944	2.025001
H	2.358318	-2.472329	1.834958
H	3.033962	-1.077058	2.668886
C	0.857454	-0.395807	-0.924716
H	0.469957	0.316511	-1.652954
C	3.101868	0.421710	-1.474385
H	2.821080	0.875943	-2.417953
C	-1.500673	-1.081357	-0.776060
C	0.184085	-2.614788	0.022835
C	-2.091235	-2.514039	-0.741376
H	-1.597201	-0.612764	-1.761842
C	-0.863933	-3.441719	-0.729955
H	0.021932	-2.663698	1.104182
H	1.218610	-2.885704	-0.195352
H	-2.671995	-2.643584	0.175548
H	-2.760952	-2.694688	-1.585165
H	-1.064928	-4.403958	-0.249670
H	-0.513760	-3.635162	-1.750486
N	-0.074165	-1.261302	-0.480772
C	-2.164489	-0.143064	0.275708
F	-1.930400	-0.779457	1.516188
C	-3.674060	-0.032449	0.101894
C	-6.458859	0.261553	-0.130304
C	-4.473662	0.100248	1.244206
C	-4.286264	-0.019725	-1.157934
C	-5.670070	0.128623	-1.272714

C	-5.854910	0.244985	1.128935
H	-4.004800	0.084379	2.221688
H	-3.694368	-0.127895	-2.061869
H	-6.128641	0.135462	-2.257755
H	-6.460708	0.345272	2.025427
H	-7.535646	0.375140	-0.219731
C	-1.475575	1.216075	0.314203
C	-0.037398	3.623887	0.255221
C	-1.718922	2.162699	-0.688860
C	-0.516801	1.492306	1.294479
C	0.197270	2.690325	1.264327
C	-1.001516	3.358113	-0.719795
H	-2.474321	1.970657	-1.445145
H	-0.319657	0.761678	2.069246
H	0.950068	2.883550	2.023015
H	-1.198096	4.082534	-1.505512
H	0.527445	4.551324	0.226273

Ic

C	-1.831068	0.982258	-0.377696
C	-3.270787	1.189293	-0.226316
C	-2.848452	-1.008308	-0.839018
C	-3.233171	-2.349824	-1.148085
C	-4.559756	-2.686839	-1.142411
C	-5.578348	-1.716863	-0.832128
C	-5.257556	-0.421722	-0.526248
C	-3.884930	-0.019891	-0.511956
H	-2.470519	-3.089339	-1.384239
H	-4.863165	-3.704786	-1.375745
H	-6.619106	-2.031333	-0.843425
H	-6.039464	0.298084	-0.293803
C	-3.947040	2.474586	0.149946
H	-3.780489	3.273901	-0.587310
H	-5.029719	2.333596	0.225207
H	-3.607873	2.860109	1.122507
C	-0.963138	2.040473	-0.186236
H	-1.414320	2.984540	0.109567
C	-1.612016	-0.397836	-0.760615
H	-0.665062	-0.900499	-0.878374
C	1.065787	3.438778	-0.289037
C	1.287880	1.115187	-0.814825
C	1.893053	3.388295	-1.579369
H	1.711497	3.502810	0.593504
C	2.312112	1.904721	-1.687006
H	0.707174	0.414089	-1.410136
H	1.257943	3.671014	-2.425977
H	2.750175	4.067538	-1.555477
H	2.298436	1.548484	-2.719773
H	3.322483	1.756657	-1.302442
N	0.374719	2.142616	-0.305342
H	0.341064	4.255957	-0.258194
C	2.011160	0.345989	0.337957
C	2.973226	-0.693266	-0.223640
C	2.614066	-1.536682	-1.284117
C	4.241969	-0.832264	0.351391
C	3.506694	-2.499917	-1.757482
H	1.634107	-1.458047	-1.745591
C	5.133869	-1.792713	-0.124575
H	4.525282	-0.180863	1.169927
C	4.770229	-2.630317	-1.180564
H	3.211090	-3.146157	-2.579402
H	6.115453	-1.886139	0.332236
H	5.465492	-3.378695	-1.550715
C	1.067416	-0.205754	1.400686
C	0.579619	0.659108	2.389996
C	0.642542	-1.538115	1.399819
C	-0.341347	0.208266	3.332684
H	0.924329	1.686015	2.417800
C	-0.281725	-1.988556	2.342463
H	1.024028	-2.231287	0.658089
C	-0.782549	-1.115974	3.307068
H	-0.716177	0.893708	4.088130
H	-0.613566	-3.022455	2.315128
H	-1.508950	-1.464681	4.035802
F	2.796709	1.341864	0.984000

Id

C	-1.770177	1.375979	-0.408786
C	-1.951873	0.336664	-1.406875
C	-3.732478	0.318099	0.106133
C	-4.941355	-0.208348	0.652977
C	-5.553983	-1.267164	0.035155
C	-5.002858	-1.851922	-1.157284
C	-3.840241	-1.382686	-1.713794
C	-3.149506	-0.301019	-1.088152
H	-5.365043	0.234230	1.552130

H	-6.474970	-1.677648	0.441844
H	-5.524978	-2.687460	-1.617654
H	-3.432649	-1.845489	-2.610204
C	-1.186176	0.115191	-2.680410
H	-0.636246	1.011993	-2.990294
H	-0.459488	-0.714251	-2.630204
H	-1.868748	-0.136846	-3.501227
C	-0.724927	2.239467	-0.118589
H	-0.966064	3.087171	0.524057
C	-2.916088	1.368627	0.480652
H	-3.020049	1.997898	1.357004
C	1.440295	3.392747	-0.266278
C	1.371940	1.052477	-0.868612
C	2.749996	3.061649	-1.020282
H	1.618259	3.546222	0.804446
C	2.492729	1.695877	-1.699221
H	0.764412	0.372488	-1.450673
H	2.998085	3.835138	-1.752178
H	3.578619	2.984510	-0.315273
H	2.127013	1.836915	-2.722040
H	3.386266	1.069849	-1.744482
N	0.572452	2.217330	-0.475442
H	0.939259	4.279797	-0.665118
C	1.896651	0.266651	0.373781
C	2.763143	-0.925717	-0.021793
C	2.468509	-1.736494	-1.125820
C	3.871480	-1.245615	0.773305
C	3.262992	-2.844897	-1.424804
H	1.620810	-1.516307	-1.766771
C	4.664765	-2.351406	0.472671
H	4.107697	-0.621456	1.627497
C	4.363749	-3.156841	-0.627553
H	3.018502	-3.461413	-2.285497
H	5.520404	-2.583927	1.101002
H	4.982027	-4.019191	-0.861086
C	0.768851	-0.132963	1.319013
C	0.615369	0.511077	2.549874
C	-0.165739	-1.100993	0.931629
C	-0.465121	0.196452	3.377783
H	1.337875	1.257469	2.858333
C	-1.254016	-1.397002	1.747021
H	-0.067030	-1.605654	-0.023293
C	-1.406574	-0.748830	2.974671
H	-0.573476	0.703532	4.333123
H	-1.997627	-2.111777	1.409065
H	-2.262178	-0.974626	3.604863
F	2.736226	1.171463	1.072474

IIa

C	-1.341849	-2.460182	1.542344
C	1.107606	-2.628171	1.254483
H	1.290974	-2.351404	2.293343
Pd	0.044390	-0.760499	0.586506
P	-1.710615	0.673400	-0.210603
C	-2.731293	-0.348910	-1.344463
C	-4.131044	-0.354458	-1.333266
C	-2.756022	-1.899610	-3.216899
C	-4.835292	-1.122214	-2.262475
H	-4.676612	0.222669	-0.595217
C	-4.153295	-1.887300	-3.209571
H	-2.218591	-2.497826	-3.947537
H	-5.921134	-1.124456	-2.240023
H	-4.706666	-2.477796	-3.934110
C	-2.819718	1.340737	1.087692
C	-2.646588	0.940528	2.420047
C	-3.823626	2.276231	0.781875
C	-3.467298	1.449022	3.428407
H	-1.861680	0.233002	2.669142
C	-4.649460	2.776508	1.787941
C	-4.473679	2.363150	3.111884
H	-3.318551	1.134070	4.457475
H	-5.424540	3.495870	1.540154
H	-5.113661	2.760482	3.894362
C	-1.322175	2.172418	-1.212512
C	-0.995795	3.369878	-0.553098
C	-1.333892	2.150727	-2.614709
C	-0.693271	4.519278	-1.281459
C	-1.036725	3.305901	-3.338997
H	-1.584567	1.242256	-3.150000
C	-0.717416	4.492262	-2.677654
H	-0.448964	5.437175	-0.754259
H	-1.056237	3.273961	-4.424355
H	-0.491424	5.390422	-3.245393
H	-0.993797	3.416465	0.529442
H	-3.948667	2.622657	-0.239666
C	-2.048032	-1.142619	-2.284005

H	-0.960183	-1.150050	-2.296600
C	2.277741	-3.130826	0.514551
C	3.478918	-3.373989	1.198132
C	2.228444	-3.395694	-0.867841
C	4.591491	-3.880618	0.528281
H	3.540063	-3.163069	2.261478
C	3.340639	-3.897021	-1.537654
H	1.314595	-3.197276	-1.422567
C	4.527384	-4.145471	-0.840220
H	5.511192	-4.059884	1.076474
H	3.284533	-4.094273	-2.604579
H	5.395042	-4.537329	-1.362801
C	-0.219400	-2.987433	0.893213
H	-0.363751	-3.577959	-0.007535
H	-1.183828	-2.028861	2.531834
C	-2.740123	-2.731300	1.200353
C	-3.749167	-2.270146	2.066438
C	-3.121694	-3.454396	0.053941
C	-5.092789	-2.523205	1.801322
H	-3.474986	-1.710615	2.955785
C	-4.462208	-3.717910	-0.202719
H	-2.373202	-3.807020	-0.647081
C	-5.453827	-3.253424	0.667466
H	-5.854849	-2.158709	2.483855
H	-4.736755	-4.277945	-1.091042
H	-6.499979	-3.460097	0.461230
P	1.917743	0.642411	0.235292
C	3.599522	0.015073	0.629537
C	3.954665	-0.097466	1.985074
C	4.531782	-0.334309	-0.354911
C	5.230000	-0.526579	2.345857
H	3.242081	0.176347	2.759335
C	5.806857	-0.770095	0.012533
H	4.275510	-0.260491	-1.405946
C	6.161645	-0.860038	1.358333
H	5.498749	-0.594932	3.396264
H	6.522947	-1.035854	-0.759579
H	7.157790	-1.189941	1.638956
C	1.985339	1.097117	-1.539621
C	2.163162	2.403797	-2.008263
C	1.845482	0.047307	-2.465405
C	2.219613	2.652062	-3.381094
H	2.229972	3.233391	-1.314745
C	1.907207	0.300021	-3.834824
H	1.711003	-0.970635	-2.110158
C	2.097727	1.605604	-4.295483
H	2.347759	3.671118	-3.733289
H	1.808468	-0.520090	-4.540582
H	2.143751	1.805513	-5.362117
C	1.867592	2.180177	1.233756
C	2.916361	3.114706	1.172183
C	0.807857	2.403488	2.122862
C	2.872078	4.272083	1.948059
H	3.771508	2.930671	0.528318
C	0.770082	3.557465	2.908150
H	0.005744	1.677493	2.199407
C	1.796693	4.498098	2.813321
H	3.682482	4.992605	1.886673
H	-0.060702	3.716565	3.589834
H	1.769047	5.398047	3.420875

IIb

C	-1.646944	2.423612	-1.085102
C	0.823911	2.644298	-1.559617
Pd	-0.063598	0.710155	-0.723399
P	1.938543	-0.548527	-0.600850
C	2.464523	-0.728078	1.145089
C	2.931712	-1.924690	1.700846
C	2.873394	0.398719	3.257342
C	3.373873	-1.953649	3.025061
H	2.934198	-2.837772	1.116881
C	3.352909	-0.795333	3.802735
H	2.848829	1.306147	3.854014
H	3.727007	-2.888825	3.449495
H	3.701831	-0.823833	4.831079
C	1.831106	-2.208007	-1.369223
C	2.970007	-3.023030	-1.488952
C	0.611218	-2.653614	-1.897653
C	2.873007	-4.279926	-2.084631
H	3.931723	-2.669772	-1.127721
C	0.518414	-3.909115	-2.501140
C	1.646544	-4.726884	-2.586278
H	3.756246	-4.906624	-2.167286
H	-0.433873	-4.242283	-2.903985
H	1.576299	-5.704408	-3.054619
C	3.405760	0.174452	-1.435275

C	4.600831	0.457879	-0.762144
C	3.297269	0.474577	-2.803648
C	5.667431	1.047630	-1.447135
C	4.361669	1.062637	-3.483087
H	2.375462	0.247740	-3.334892
C	5.548919	1.357353	-2.802226
H	6.589841	1.266394	-0.916620
H	4.268528	1.291746	-4.540803
H	6.376992	1.821123	-3.330198
H	4.699264	0.233297	0.294331
H	-0.268870	-2.021600	-1.838008
C	2.426422	0.433662	1.938031
H	2.063888	1.369075	1.524285
C	-0.537964	2.547791	-1.942911
H	-0.695417	2.331865	-2.997351
H	-1.581177	2.788465	-0.067712
C	-3.023690	2.166309	-1.531507
C	-4.057024	2.266504	-0.582347
C	-3.362023	1.812036	-2.852148
C	-5.378915	1.997675	-0.931205
H	-3.817371	2.553306	0.436853
C	-4.683622	1.552712	-3.201216
H	-2.591985	1.725605	-3.613278
C	-5.697816	1.635325	-2.240708
H	-6.158733	2.075019	-0.179109
H	-4.926013	1.284869	-4.225800
H	-6.727427	1.426765	-2.515991
P	-1.466092	-0.654896	0.661380
C	-2.357105	0.465563	1.816116
C	-1.639862	1.535918	2.379097
C	-3.714983	0.315259	2.126806
C	-2.271243	2.439618	3.233190
H	-0.586028	1.668354	2.146061
C	-4.345020	1.224817	2.979864
H	-4.291532	-0.492038	1.689204
C	-3.629134	2.289082	3.530828
H	-1.705085	3.261860	3.662204
H	-5.400367	1.103332	3.206659
H	-4.124709	2.997443	4.188196
C	-2.741818	-1.604774	-0.245097
C	-3.502624	-2.606890	0.383146
C	-2.975432	-1.312298	-1.595621
C	-4.486801	-3.289434	-0.330510
H	-3.316858	-2.859946	1.422559
C	-3.963937	-1.994533	-2.306177
H	-2.391699	-0.542735	-2.088359
C	-4.720338	-2.982681	-1.674741
H	-5.069780	-4.062726	0.161336
H	-4.142701	-1.748203	-3.348487
H	-5.488397	-3.516561	-2.226994
C	-0.699445	-1.910704	1.768080
C	-0.368635	-1.604692	3.096548
C	-0.423564	-3.197317	1.275233
C	0.210291	-2.572154	3.917645
H	-0.570534	-0.619263	3.501017
C	0.157558	-4.159784	2.099943
H	-0.678277	-3.460374	0.255327
C	0.471178	-3.851598	3.425076
H	0.457093	-2.321936	4.945041
H	0.355869	-5.152418	1.705678
H	0.916681	-4.603840	4.069793
H	1.517793	2.495172	-2.383164
C	1.473336	3.248067	-0.380890
C	0.807635	3.737843	0.762906
C	2.882624	3.312904	-0.399367
C	1.526044	4.219579	1.855154
H	-0.272702	3.760871	0.805134
C	3.597825	3.790269	0.695303
H	3.418819	2.973767	-1.279211
C	2.924097	4.234387	1.835318
H	0.990231	4.593341	2.723369
H	4.682739	3.813837	0.655411
H	3.479542	4.606218	2.691250

IIc

C	-1.925320	1.189551	-2.020632
C	0.309375	2.438071	-2.019844
Pd	-0.027011	0.346901	-0.973296
P	2.116602	0.024769	-0.009637
C	1.879836	0.007610	1.804411
C	1.929658	-1.162068	2.574466
C	1.163897	1.252007	3.766417
C	1.607345	-1.118287	3.933784
H	2.190385	-2.111846	2.121727
C	1.232475	0.083826	4.533960
H	0.860892	2.189878	4.222992

H	1.642031	-2.034034	4.516100
H	0.986904	0.111955	5.591595
C	2.989566	-1.495929	-0.534982
C	4.049100	-2.039816	0.207422
C	2.671599	-2.047036	-1.784665
C	4.742313	-3.150208	-0.272789
H	4.335324	-1.598445	1.157317
C	3.379225	-3.147056	-2.271822
C	4.407397	-3.707100	-1.510624
H	5.552159	-3.573958	0.313898
H	3.123976	-3.568663	-3.239859
H	4.954882	-4.567170	-1.885199
C	3.423550	1.279608	-0.335918
C	4.145219	1.933352	0.671768
C	3.711959	1.563835	-1.681315
C	5.113508	2.883691	0.334932
C	4.682130	2.506442	-2.013044
H	3.179319	1.039287	-2.470938
C	5.377802	3.178325	-1.002844
H	5.662355	3.390682	1.123418
H	4.894245	2.719496	-3.056723
H	6.127024	3.921220	-1.259763
H	3.953079	1.715161	1.716645
H	1.856648	-1.625126	-2.368345
C	1.473584	1.214510	2.408616
H	1.392278	2.122460	1.815883
C	-0.713572	1.654642	-2.603372
H	-0.431553	1.128516	-3.515638
P	-1.232181	-1.527352	-0.066110
C	-2.410452	-0.872859	1.170721
C	-3.798930	-0.860937	0.984701
C	-1.866155	-0.298796	2.333382
C	-4.629244	-0.316432	1.965836
H	-4.236195	-1.265599	0.078931
C	-2.700692	0.228447	3.315554
H	-0.792102	-0.272202	2.475945
C	-4.086151	0.216747	3.135508
H	-5.703588	-0.304677	1.809320
H	-2.264773	0.653939	4.215016
H	-4.738877	0.633209	3.897291
C	-2.187682	-2.454029	-1.328456
C	-3.099760	-3.451686	-0.943079
C	-1.943479	-2.239666	-2.692012

C	-3.782403	-4.188879	-1.908803
H	-3.271214	-3.652170	0.110596
C	-2.620279	-2.987710	-3.658240
H	-1.212143	-1.496232	-2.996762
C	-3.546267	-3.955737	-3.267459
H	-4.492282	-4.951666	-1.602403
H	-2.422023	-2.815342	-4.712311
H	-4.076422	-4.535375	-4.017533
C	-0.362706	-2.905535	0.796958
C	-0.572619	-3.246279	2.140695
C	0.544458	-3.661195	0.038323
C	0.144031	-4.296718	2.718686
H	-1.284957	-2.696929	2.744291
C	1.269108	-4.699047	0.619504
H	0.687392	-3.440385	-1.012437
C	1.077157	-5.013782	1.967925
H	-0.030118	-4.551815	3.760348
H	1.982715	-5.255065	0.019198
H	1.640380	-5.822236	2.425190
H	1.267172	2.393291	-2.532964
C	0.155385	3.616116	-1.142037
C	1.200831	4.000949	-0.282757
C	-0.986529	4.432166	-1.217930
C	1.079591	5.130189	0.523188
H	2.109247	3.412707	-0.250284
C	-1.100111	5.570348	-0.421362
H	-1.782423	4.175175	-1.908085
C	-0.075955	5.915915	0.462596
H	1.894463	5.405693	1.186969
H	-1.991086	6.187055	-0.493608
H	-0.167488	6.801740	1.084501
H	-2.425611	0.461502	-2.655741
C	-2.860158	1.775801	-1.032096
C	-2.500122	2.304331	0.220019
C	-4.222065	1.778030	-1.388021
C	-3.461321	2.860439	1.057770
H	-1.467578	2.270113	0.547390
C	-5.182830	2.349363	-0.555249
H	-4.525470	1.347964	-2.339560
C	-4.803068	2.899624	0.669682
H	-3.161070	3.257122	2.022450
H	-6.224868	2.359002	-0.861627
H	-5.548223	3.340174	1.325793

TS-*endo*

C	0.519561	0.448842	0.515052
H	1.216877	0.083987	-0.237921
Pd	-1.497619	-0.283279	-0.032099
P	-1.161716	-2.615561	-0.221197
C	-1.870519	-3.397015	-1.730512
C	-1.462359	-4.665742	-2.177988
C	-3.420331	-3.265941	-3.600049
C	-2.040418	-5.231239	-3.314687
H	-0.686578	-5.206791	-1.643303
C	-3.024303	-4.535386	-4.024191
H	-4.172855	-2.708196	-4.150655
H	-1.720230	-6.213993	-3.649622
C	-1.829430	-3.549325	1.214148
C	-2.363547	-4.842374	1.122757
C	-1.798541	-2.905470	2.462563
C	-2.864401	-5.477241	2.260692
H	-2.408466	-5.349700	0.164489
C	-2.293764	-3.545641	3.597554
C	-2.832797	-4.830508	3.498056
H	-3.284400	-6.476044	2.178311
H	-2.267421	-3.035114	4.556037
C	0.595597	-3.167479	-0.316152
C	1.384434	-2.658299	-1.362347
C	1.186894	-4.020316	0.625153
C	2.727250	-3.013303	-1.474986
C	2.540897	-4.356672	0.524855
H	0.593397	-4.418389	1.441537
C	3.313781	-3.859106	-0.525079
H	3.314905	-2.648195	-2.312038
H	2.985399	-5.019454	1.262328
H	0.935590	-1.993698	-2.097773
H	-1.394608	-1.900131	2.544175
C	-2.841843	-2.698049	-2.463281
H	-3.139342	-1.702555	-2.154862
C	0.790896	-0.028828	1.875936
C	1.714209	-1.075915	2.060883
C	0.145811	0.490422	3.018327
C	1.944906	-1.618649	3.323118
H	2.226534	-1.485822	1.196874
C	0.387499	-0.044214	4.281387
H	-0.538766	1.324957	2.914534
C	1.279443	-1.110420	4.441621
H	2.640289	-2.447168	3.429137
H	-0.122741	0.371528	5.146024
H	1.456592	-1.533474	5.425975
C	-0.242966	1.604057	0.177417
H	-0.709323	2.212895	0.944531
C	-1.124897	2.980134	-1.813676
C	-1.064265	3.098592	-3.218769
C	-2.125444	3.692596	-1.124808
C	-1.978844	3.882526	-3.913659
H	-0.294977	2.555333	-3.764127
C	-3.044798	4.471733	-1.825350
H	-2.198626	3.623443	-0.046971
C	-2.978857	4.570856	-3.215795
H	-1.918673	3.955499	-4.995916
H	-3.817137	4.998273	-1.274709
H	-3.700260	5.177673	-3.755225
C	-0.165513	2.111332	-1.147956
C	0.986062	4.597673	-0.127810
C	1.275624	4.322385	1.257497
C	0.083708	5.625752	-0.463364
C	2.239317	3.286403	1.316844
C	0.625959	5.054356	2.278973
C	-0.534042	6.330392	0.557930
H	-0.133454	5.851278	-1.502460
C	-0.273522	6.041887	1.923233
H	0.832442	4.846490	3.325306
H	-1.237581	7.120796	0.311563
H	-0.781158	6.616731	2.692354
C	2.864883	2.739691	2.561978
H	2.472595	3.239159	3.451411
H	3.953212	2.876696	2.556070
H	2.679301	1.666429	2.681695
C	2.536023	2.895447	-0.032669
C	3.409489	1.826715	-0.240037
H	3.850735	1.403047	0.651425
C	1.681639	3.652237	-0.932077
N	3.784880	1.210290	-1.352731
C	4.700434	0.030439	-1.365101
C	4.863354	-0.269423	-2.867266
C	3.581321	0.273061	-3.503771
C	3.336583	1.557180	-2.712342
H	5.035134	-1.331580	-3.047953

H	5.732480	0.275481	-3.247950
H	3.678028	0.456105	-4.576833
H	2.745875	-0.420885	-3.348538
H	3.940063	2.393591	-3.086705
H	2.291665	1.862009	-2.693973
H	4.194169	-0.799548	-0.863623
C	6.068831	0.291203	-0.674510
F	6.536132	1.479931	-1.260827
C	7.043342	-0.834207	-1.025122
C	8.211956	-0.553682	-1.739369
C	6.770793	-2.157329	-0.651091
C	9.094352	-1.583012	-2.076446
H	8.431009	0.467213	-2.028630
C	7.649610	-3.183528	-0.994809
H	5.877545	-2.394983	-0.083542
C	8.815903	-2.899755	-1.710058
H	10.001104	-1.350710	-2.627641
H	7.426990	-4.204675	-0.697262
H	9.502663	-3.698513	-1.974286
C	6.008856	0.507986	0.837654
C	6.736510	1.560032	1.406361
C	5.262617	-0.332641	1.677471
C	6.721215	1.765389	2.787670
H	7.310311	2.218792	0.765762
C	5.242301	-0.120511	3.056296
H	4.666102	-1.141446	1.267410
C	5.975625	0.926556	3.617589
H	7.293003	2.585618	3.212265
H	4.636980	-0.764552	3.685880
H	5.959024	1.091193	4.690919
P	-3.848822	0.201384	0.101085
C	-4.832893	-0.981071	1.109778
C	-6.194423	-2.896583	2.646904
C	-4.883965	-2.323442	0.697402
C	-5.477543	-0.609980	2.298268
C	-6.149690	-1.566191	3.063841
C	-5.565690	-3.271160	1.456448
H	-4.389457	-2.632524	-0.218105
H	-5.458652	0.422606	2.630896
H	-6.642510	-1.265661	3.984435
H	-5.587194	-4.305703	1.128440
H	-6.715355	-3.639038	3.244864
C	-4.764503	0.340232	-1.484800
C	-6.034046	0.597031	-3.972070
C	-4.082301	0.902185	-2.577056
C	-6.088479	-0.092333	-1.650308
C	-6.716464	0.032155	-2.891694
C	-4.717152	1.037209	-3.810893
H	-3.055660	1.234792	-2.459637
H	-6.624426	-0.532824	-0.815201
H	-7.740254	-0.310711	-3.013183
H	-4.180048	1.483654	-4.642864
H	-6.526896	0.693301	-4.935586
C	-4.126266	1.824514	0.927309
C	-4.313746	4.340984	2.161788
C	-5.048785	2.771885	0.466097
C	-3.317856	2.142860	2.032120
C	-3.407002	3.391422	2.644712
C	-5.140664	4.022906	1.083089
H	-5.682237	2.543317	-0.385007
H	-2.610096	1.406034	2.401362
H	-2.763713	3.629918	3.487237
H	-5.857027	4.751847	0.713600
H	-4.377395	5.319916	2.628210
H	-3.230020	-5.324574	4.380322
H	4.358248	-4.141424	-0.621277
H	-3.471260	-4.979368	-4.909298
H	1.824751	3.766576	-1.997150
H	0.421299	1.499665	-1.829716

TS-*exo*

C	0.751443	0.844666	-2.053526
H	0.050460	1.271353	-2.765890
Pd	-1.679155	0.004314	-0.464825
P	-1.743394	-2.398518	-0.236348
C	-3.357724	-3.213845	0.147365
C	-4.372969	-3.098774	-0.814759
C	-4.885553	-4.471674	1.552145
C	-5.636281	-3.640628	-0.588300
H	-4.181060	-2.570825	-1.741733
C	-5.898127	-4.325375	0.602649
H	-5.077366	-5.013739	2.474362
H	-6.414462	-3.515054	-1.335464
C	-0.765282	-2.771386	1.285997
C	0.172583	-3.808533	1.392447
C	-1.044387	-1.987520	2.421722

C	0.796619	-4.074467	2.615709
H	0.409468	-4.419541	0.527563
C	-0.425345	-2.260212	3.641060
C	0.493699	-3.309217	3.744230
H	1.517453	-4.884602	2.685692
H	-0.673459	-1.655871	4.509544
C	-1.119285	-3.518149	-1.554432
C	-0.609451	-2.952129	-2.729139
C	-1.210552	-4.917728	-1.451496
C	-0.174715	-3.766957	-3.777074
C	-0.766095	-5.730139	-2.493138
H	-1.646361	-5.367798	-0.563755
C	-0.245678	-5.154994	-3.657409
H	0.220085	-3.313128	-4.680482
H	-0.838805	-6.810598	-2.404396
H	-0.557254	-1.872837	-2.820601
H	-1.763381	-1.175694	2.361081
C	-3.622179	-3.916496	1.330398
H	-2.854905	-4.022466	2.089389
C	1.550902	-0.282052	-2.572264
C	1.698934	-0.448325	-3.962667
C	2.186097	-1.207827	-1.726029
C	2.478424	-1.478743	-4.487294
H	1.203041	0.248609	-4.634775
C	2.967124	-2.236736	-2.248591
H	2.044228	-1.137745	-0.654977
C	3.126252	-2.374493	-3.630616
H	2.578991	-1.584554	-5.563916
H	3.432177	-2.952880	-1.575386
H	3.726580	-3.183466	-4.036166
C	0.396777	0.960383	-0.676221
H	1.076103	0.529687	0.052575
C	-0.652203	2.329940	1.191201
C	-1.275848	3.552607	1.504363
C	-0.100622	1.579726	2.249553
C	-1.337992	4.011933	2.820851
H	-1.719402	4.140442	0.709185
C	-0.159907	2.042156	3.561209
H	0.362867	0.619994	2.050187
C	-0.778023	3.261593	3.857983
H	-1.833416	4.955247	3.033421
H	0.273988	1.444756	4.358497
H	-0.826010	3.618704	4.882779
C	-0.584181	1.884592	-0.215347
H	-0.982156	2.593783	-0.936065
C	1.276122	3.615421	-2.401389
C	1.314131	4.187397	-1.088583
C	0.361729	4.103532	-3.351617
C	2.285764	3.486980	-0.315253
C	0.449467	5.247242	-0.743495
C	-0.480194	5.147319	-2.993091
H	0.320385	3.677828	-4.351111
C	-0.438661	5.716656	-1.695631
H	0.473659	5.675844	0.253330
H	-1.181616	5.544811	-3.721844
H	-1.115443	6.529488	-1.450698
C	2.563107	3.762714	1.128576
H	3.203677	4.647811	1.244016
H	3.052524	2.928439	1.639070
H	1.626439	3.962560	1.659389
C	2.912139	2.536746	-1.172559
C	3.943923	1.719979	-0.691916
H	4.132517	1.762761	0.370017
C	2.187806	2.508320	-2.442410
H	2.576789	2.110324	-3.367845
N	4.747077	0.911152	-1.359904
C	5.755004	-0.003044	-0.758616
C	6.931004	0.146749	-1.728722
C	6.225284	0.171269	-3.092934
H	7.648827	-0.668766	-1.629675
H	7.445950	1.090662	-1.521244
H	6.817653	0.659498	-3.870324
H	6.014105	-0.850815	-3.424492
H	5.354870	-1.019755	-0.859770
C	6.079161	0.229652	0.731118
F	6.464044	1.583159	0.828976
C	7.271463	-0.638800	1.128772
C	8.528068	-0.059610	1.331580
C	7.123034	-2.025800	1.254836
C	9.623793	-0.861624	1.657729
H	8.645888	1.013559	1.239827
C	8.221508	-2.824717	1.570904
H	6.147564	-2.485213	1.124763
C	9.475951	-2.244348	1.773818
H	10.594230	-0.401405	1.820182
H	8.094940	-3.899237	1.667633

H	10.330160	-2.865919	2.025879
C	4.892465	0.020486	1.673075
C	4.795228	0.809056	2.827404
C	3.899787	-0.933095	1.417650
C	3.713836	0.655795	3.696915
H	5.559981	1.550367	3.031368
C	2.814769	-1.081623	2.282108
H	3.952885	-1.560149	0.534692
C	2.719958	-0.287021	3.425163
H	3.648515	1.277738	4.585032
H	2.047824	-1.815589	2.063328
H	1.876048	-0.412087	4.096115
C	4.899730	0.914370	-2.832358
H	4.050173	0.418015	-3.301541
H	4.923198	1.957898	-3.160702
P	-3.845948	0.768299	0.116665
C	-5.257464	0.196478	-0.911680
C	-4.994595	-0.179911	-2.236291
C	-6.581833	0.197328	-0.449141
C	-6.034913	-0.570724	-3.081088
H	-3.967580	-0.185682	-2.594755
C	-7.618985	-0.208293	-1.288858
H	-6.802123	0.504909	0.568882
C	-7.347662	-0.594797	-2.604773
H	-5.819736	-0.864326	-4.104869
H	-8.640301	-0.216691	-0.918498
H	-8.158169	-0.906440	-3.257685
C	-4.113493	2.592287	0.078026
C	-4.746533	3.319146	1.094630
C	-3.658551	3.272557	-1.063165
C	-4.894864	4.703715	0.983004
H	-5.114382	2.812494	1.980721
C	-3.822941	4.651597	-1.181531
H	-3.161992	2.719701	-1.856740
C	-4.432054	5.373714	-0.151073
H	-5.377958	5.257407	1.783464
H	-3.462199	5.161390	-2.069776
H	-4.550707	6.450429	-0.234882
C	-4.179716	0.236986	1.840985
C	-3.504286	0.894190	2.886998
C	-4.905269	-0.929268	2.128867
C	-3.571421	0.404666	4.190284
H	-2.919466	1.783397	2.686010
C	-4.967738	-1.414203	3.437017
H	-5.405333	-1.476969	1.338069
C	-4.304362	-0.752227	4.470760
H	-3.044445	0.930226	4.981813
H	-5.528589	-2.322122	3.637230
H	-4.354523	-1.135690	5.486078
H	0.090379	-5.790329	-4.472369
H	-6.882888	-4.746304	0.785310
H	0.975282	-3.524157	4.693941

ent*1-TS-*exo

C	-0.735497	1.856510	-0.285069
H	-1.125120	2.552558	0.453164
Pd	-1.718572	-0.094902	0.054518
P	-3.982079	0.602828	0.085794
C	-4.822000	0.244731	1.686650
C	-5.997636	0.908100	2.078324
C	-4.888385	-1.031594	3.756550
C	-6.617067	0.591968	3.287852
H	-6.420557	1.681978	1.444218
C	-6.067399	-0.382061	4.126483
H	-4.443465	-1.779101	4.407711
H	-7.526950	1.109964	3.578121
C	-5.021394	-0.167713	-1.220129
C	-6.397910	-0.395680	-1.081721
C	-4.380485	-0.547990	-2.409948
C	-7.117340	-0.999091	-2.114023
H	-6.908378	-0.120735	-0.164503
C	-5.102397	-1.145880	-3.442127
C	-6.471667	-1.376586	-3.293628
H	-8.182341	-1.178487	-1.994433
H	-4.593714	-1.445625	-4.353860
C	-4.285448	2.406384	-0.114496
C	-3.822176	3.273963	0.890325
C	-4.889602	2.949134	-1.255916
C	-3.981767	4.652151	0.763732
C	-5.035772	4.333173	-1.386368
H	-5.248513	2.294962	-2.043886
C	-4.586984	5.187333	-0.378732
H	-3.629173	5.307253	1.554162
H	-5.507251	4.740183	-2.276658
H	-3.341727	2.869498	1.778034
H	-3.310715	-0.385928	-2.516545

C	-4.266732	-0.715633	2.547138
H	-3.338664	-1.209613	2.278583
C	-0.912105	2.269297	-1.687367
C	-1.605192	3.458569	-1.976959
C	-0.396157	1.523572	-2.769369
C	-1.769492	3.890273	-3.292622
H	-2.017603	4.042064	-1.161190
C	-0.557328	1.959745	-4.082907
H	0.117328	0.585423	-2.580703
C	-1.244925	3.147824	-4.353105
H	-2.315794	4.809119	-3.486871
H	-0.152355	1.367607	-4.899771
H	-1.374048	3.485085	-5.377423
C	0.293350	0.962871	0.124880
H	0.949343	0.542309	-0.630739
C	1.594501	-0.214013	1.967941
C	1.922404	-0.273271	3.336787
C	2.124639	-1.204129	1.120105
C	2.764335	-1.263561	3.836227
H	1.511611	0.475086	4.010800
C	2.965683	-2.198034	1.621069
H	1.840431	-1.234028	0.074681
C	3.297688	-2.230178	2.977417
H	3.000670	-1.286136	4.896341
H	3.332258	-2.967809	0.948025
H	3.947820	-3.008960	3.365239
C	0.707254	0.855732	1.480345
C	1.140192	3.709791	1.855235
C	1.049210	4.356547	0.577509
C	0.297541	4.110906	2.909604
C	1.988979	3.765547	-0.321659
C	0.118331	5.398169	0.373083
C	-0.609215	5.135622	2.686649
H	0.360989	3.629114	3.882150
C	-0.699962	5.774770	1.423281
H	0.031837	5.881786	-0.595107
H	-1.259100	5.464093	3.493177
H	-1.423170	6.572445	1.283581
C	2.202175	4.300257	-1.704939
H	2.100447	5.390918	-1.697459
H	1.448636	3.919465	-2.409016
H	3.195151	4.070837	-2.095335
C	2.661757	2.740252	0.397410

C	3.534013	1.686339	0.065162
H	3.923200	1.150380	0.922772
C	2.102748	2.665531	1.745608
N	3.882921	1.133180	-1.083753
C	4.719463	-0.100325	-1.118235
C	4.517276	-0.656380	-2.542947
C	3.252084	0.041353	-3.057463
C	3.318751	1.423154	-2.411547
H	4.426276	-1.744237	-2.531919
H	5.381014	-0.394582	-3.159882
H	3.203847	0.095775	-4.148458
H	2.358739	-0.481758	-2.702162
H	3.991331	2.094837	-2.955447
H	2.342134	1.890166	-2.302219
H	4.321766	-0.777768	-0.359928
C	6.208690	0.192162	-0.790088
F	6.609039	1.141947	-1.750067
C	7.072084	-1.051823	-0.964888
C	8.366573	-0.904686	-1.477331
C	6.626558	-2.328299	-0.594373
C	9.200713	-2.013649	-1.616003
H	8.713692	0.079736	-1.770258
C	7.464210	-3.436638	-0.732063
H	5.625263	-2.474866	-0.199374
C	8.753609	-3.283030	-1.243004
H	10.202051	-1.884539	-2.016611
H	7.105499	-4.420244	-0.441841
H	9.404562	-4.145661	-1.351018
C	6.361098	0.821515	0.590485
C	6.632388	2.187505	0.719675
C	6.097976	0.062673	1.739949
C	6.626291	2.789745	1.980540
H	6.832792	2.778477	-0.165915
C	6.074091	0.669646	2.994629
H	5.887569	-0.998541	1.659920
C	6.335993	2.037634	3.119056
H	6.837697	3.851570	2.067762
H	5.848344	0.072370	3.873116
H	6.319409	2.510276	4.096835
P	-1.592073	-2.484233	0.040099
C	-2.967088	-3.399740	-0.776558
C	-5.169559	-4.667928	-1.971922
C	-4.260553	-3.229477	-0.254780

C	-2.790715	-4.220279	-1.899183
C	-3.889225	-4.845982	-2.495157
C	-5.351144	-3.862774	-0.844234
H	-4.420516	-2.594626	0.610805
H	-1.800915	-4.377840	-2.314156
H	-3.738472	-5.476678	-3.367084
H	-6.345119	-3.709755	-0.435460
H	-6.022681	-5.151576	-2.439180
C	-1.442245	-3.317139	1.671399
C	-1.125537	-4.508306	4.190894
C	-0.910868	-2.584157	2.743418
C	-1.822695	-4.653167	1.876059
C	-1.665661	-5.242844	3.131153
C	-0.746343	-3.178455	3.994938
H	-0.624449	-1.548855	2.593163
H	-2.246702	-5.227241	1.057407
H	-1.966457	-6.275812	3.282408
H	-0.324047	-2.601281	4.812686
H	-1.005525	-4.971268	5.166464
C	-0.104709	-3.044883	-0.898132
C	2.143141	-3.780480	-2.422705
C	0.802429	-3.996861	-0.413974
C	0.130310	-2.461421	-2.156149
C	1.233691	-2.840617	-2.920751
C	1.924418	-4.353147	-1.168496
H	0.639343	-4.457146	0.554900
H	-0.564356	-1.717782	-2.540131
H	1.385009	-2.404929	-3.904518
H	2.619183	-5.092977	-0.779326
H	3.004804	-4.073776	-3.016130
H	-7.032893	-1.853778	-4.092100
H	-4.708971	6.262268	-0.478664
H	-6.551889	-0.624479	5.068182
H	2.630713	2.215525	2.576396
H	0.046093	1.286784	2.226173

ent2*-TS-*exo

C	-0.427787	1.705047	-0.256455
H	-0.323135	2.217577	0.696246
Pd	-1.779291	0.002692	0.092416
P	-3.741507	1.098991	0.785808
C	-4.600080	0.357234	2.230877
C	-5.804287	0.888635	2.721291
C	-4.640613	-1.308868	3.998120
C	-6.428623	0.312319	3.826771
H	-6.252275	1.751448	2.235958
C	-5.849314	-0.789453	4.464636
H	-4.180743	-2.158926	4.494832
H	-7.363560	0.725290	4.195039
C	-4.957232	1.156687	-0.585449
C	-6.183711	0.481398	-0.558276
C	-4.566589	1.824494	-1.761732
C	-7.013823	0.489556	-1.681826
H	-6.487567	-0.070317	0.323570
C	-5.402301	1.834880	-2.875934
C	-6.630533	1.167183	-2.839025
H	-7.957925	-0.045964	-1.649452
H	-5.089494	2.363803	-3.772102
C	-3.526854	2.848063	1.319982
C	-2.510828	3.105886	2.255711
C	-4.280199	3.918129	0.820939
C	-2.255611	4.406291	2.685599
C	-4.013947	5.223694	1.243210
H	-5.064082	3.739832	0.092258
C	-3.002918	5.471929	2.172708
H	-1.465409	4.588609	3.407733
H	-4.600733	6.046445	0.843806
H	-1.908546	2.284086	2.636166
H	-3.613882	2.340701	-1.804805
C	-4.018285	-0.737002	2.886035
H	-3.079970	-1.144900	2.520734
C	-0.932521	2.547625	-1.353505
C	-1.391369	3.848228	-1.067805
C	-0.987111	2.107289	-2.692178
C	-1.898503	4.667939	-2.075653
H	-1.360984	4.207788	-0.045199
C	-1.490489	2.929040	-3.697864
H	-0.645111	1.108915	-2.945803
C	-1.954019	4.214170	-3.396030
H	-2.259182	5.661132	-1.823106
H	-1.527585	2.564540	-4.721120
H	-2.352151	4.851609	-4.180128
C	0.371747	0.539860	-0.461185
H	0.574887	0.201947	-1.472673
C	1.696483	-1.385490	0.575058
C	2.318500	-1.878976	1.739939

C	1.587497	-2.244038	-0.533243
C	2.810641	-3.180529	1.798898
H	2.402009	-1.229665	2.609092
C	2.062634	-3.553495	-0.465482
H	1.079051	-1.913580	-1.431589
C	2.678953	-4.028336	0.694771
H	3.284659	-3.539304	2.708306
H	1.931849	-4.206543	-1.322919
H	3.044849	-5.049542	0.742729
C	1.125649	-0.030228	0.594473
C	2.533750	2.366334	1.248935
C	2.161966	3.323504	0.244809
C	2.253108	2.629450	2.604148
C	2.536038	2.812464	-1.025902
C	1.511121	4.524087	0.606728
C	1.621053	3.817262	2.936967
H	2.534208	1.916472	3.375565
C	1.245738	4.759732	1.942825
H	1.215343	5.241459	-0.152461
H	1.410425	4.039250	3.979825
H	0.744898	5.675231	2.242374
C	2.301970	3.530726	-2.316998
H	3.091185	4.272046	-2.504005
H	1.345634	4.062523	-2.287872
H	2.263832	2.859018	-3.179806
C	3.200266	1.562224	-0.808385
C	3.805672	0.897472	-1.880341
H	3.626513	1.328267	-2.862538
C	3.082607	1.221738	0.600547
N	4.594814	-0.166417	-1.938202
C	5.158657	-0.942628	-0.823558
C	5.329039	-2.340450	-1.446381
C	5.649120	-2.088345	-2.935071
C	5.048130	-0.702915	-3.246804
H	4.386121	-2.879020	-1.339327
H	6.105935	-2.914306	-0.938328
H	5.216255	-2.860762	-3.575965
H	6.726387	-2.074997	-3.099105
H	5.785546	-0.020826	-3.681895
H	4.185876	-0.757193	-3.917860
H	4.439035	-0.958236	-0.010393
C	6.517016	-0.345062	-0.320739
F	7.410525	-0.528498	-1.406846
C	7.058401	-1.145777	0.857069
C	8.438192	-1.371570	0.939838
C	6.227747	-1.629080	1.877398
C	8.976378	-2.067357	2.021828
H	9.085002	-1.002602	0.152204
C	6.769457	-2.323755	2.960820
H	5.153385	-1.481295	1.839986
C	8.144708	-2.545584	3.037011
H	10.048366	-2.235545	2.071191
H	6.112026	-2.693323	3.743197
H	8.565591	-3.087060	3.879235
C	6.465221	1.156321	-0.059446
C	6.676203	2.043394	-1.124683
C	6.158490	1.675441	1.202935
C	6.539232	3.418466	-0.938815
H	6.956664	1.654669	-2.097077
C	6.015416	3.051154	1.387191
H	6.026719	1.007298	2.047480
C	6.194544	3.926411	0.315618
H	6.707399	4.093371	-1.773646
H	5.758387	3.436344	2.369405
H	6.079307	4.996711	0.460006
P	-2.417081	-2.266138	-0.370152
C	-4.195664	-2.616228	-0.713394
C	-6.941673	-3.023941	-1.157462
C	-5.070924	-2.831167	0.365152
C	-4.717088	-2.597888	-2.014901
C	-6.080902	-2.799966	-2.232776
C	-6.432061	-3.036828	0.143137
H	-4.692115	-2.849936	1.381092
H	-4.063507	-2.434253	-2.865382
H	-6.468195	-2.784238	-3.247575
H	-7.091910	-3.208924	0.989122
H	-8.001699	-3.187380	-1.330776
C	-1.959059	-3.556431	0.852977
C	-1.134835	-5.463543	2.738364
C	-1.008186	-3.227006	1.829528
C	-2.503705	-4.851856	0.837676
C	-2.092139	-5.798619	1.775812
C	-0.593507	-4.177187	2.763902
H	-0.588976	-2.226189	1.848898
H	-3.256159	-5.114327	0.099770
H	-2.520070	-6.797085	1.757678

H	0.151423	-3.910146	3.507736
H	-0.817661	-6.203156	3.468348
C	-1.566010	-2.759532	-1.933720
C	-0.173612	-3.312924	-4.314126
C	-0.973517	-4.013207	-2.135013
C	-1.458194	-1.787491	-2.945021
C	-0.773566	-2.063539	-4.128583
C	-0.279234	-4.284843	-3.317192
H	-1.035691	-4.774774	-1.364875
H	-1.911096	-0.809774	-2.798988
H	-0.708766	-1.304873	-4.904285
H	0.176431	-5.261380	-3.459121
H	0.365068	-3.529260	-5.232512
H	-7.280677	1.171661	-3.709547
H	-2.798559	6.488134	2.498293
H	-6.336719	-1.234626	5.327555
H	3.675102	0.484353	1.115943
H	0.917016	0.354733	1.588218
